# Pd-catalyzed intramolecular addition of active methylene compounds to alkynes with subsequent cross-coupling with (hetero)aryl halides†

**DOI:** 10.1039/c9ra08002c

**Published:** 2019-12-03

**Authors:** Aleksandra Błocka, Paweł Woźnicki, Marek Stankevič, Wojciech Chaładaj

**Affiliations:** Institute of Organic Chemistry, Polish Academy of Sciences Kasprzaka 44/52 01-224 Warsaw Poland wojciech.chaladaj@icho.edu.pl; Department of Organic Chemistry, Faculty of Chemistry, Marie Curie-Skłodowska University in Lublin Gliniana 33 20-614 Lublin Poland

## Abstract

We report an efficient protocol for tandem Pd-catalyzed intramolecular addition of active methylene compounds to alkynes, followed by subsequent cross-coupling with (hetero)aryl bromides and chlorides. The reaction proceeds under mild conditions, providing excellent functional group tolerance, including unprotected OH, NH_2_ groups, enolizable ketones, or a variety of heterocycles. Mechanistic studies point towards a catalytic cycle involving oxidative addition, intramolecular nucleophilic addition to the Pd(ii)-activated alkyne, and reductive elimination, with 5-*exo-dig* cyclization being the rate limiting step.

## Abstract 2

A tandem cyclization/coupling of acetylenic active methylene compounds with aryl halides features broad scope and excellent functional group compatibility. Mechanistic studies identified 5-*exo-dig* cyclization as the rate limiting step.

## Graphical abstract

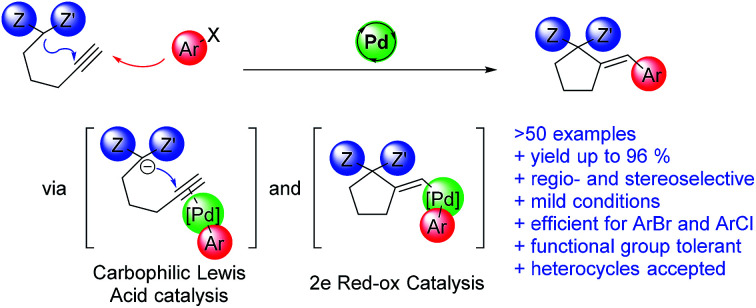

## Introduction

Palladium complexes emerge as some of the most versatile homogenous catalysts with a myriad of applications in both academic and industrial research. The most prominent area of palladium catalysis, awarded with the 2010 Nobel Price to R. Heck, A. Suzuki, and E. Negishi,^1^ covers cross-couplings of (hetero)aryl or vinyl(pseudo)halides with nucleophilic or organometallic partners. High efficiency of these and many other processes (*e.g.* Wacker oxidation) arises from the facile interconversion of palladium oxidation states through two-electron redox chemistry. Besides the most widespread Pd(0)/Pd(ii) cycle, palladium is also able to enter radical processes or to serve as a carbophilic Lewis acid in redox–neutral transformations. The ability to mediate mechanistically distinct transformations makes palladium the catalyst of choice for the design of tandem reactions in which a single metal complex catalyzes a sequence of transformations.^2^ In our research, we are focused on the development of tandem processes combining the nucleophilic addition to alkynes and subsequent cross-coupling, which give the access to a wide set of carbo- and heterocyclic systems.^3^ In contrast to cross-coupling reactions, these transformations are highly underdeveloped and suffer from harsh reaction conditions (*e.g.* the use of strong bases), narrow substrate scope (usually limited to active aryl iodides), and poor functional group tolerance, as well as insufficient mechanistic understanding.

In the late 1980s, Gore disclosed seminal works on a novel Pd-catalyzed dicarbofunctionalization of unsaturated C–C systems through arylation with iodobenzene and intramolecular nucleophilic additions of malonates to alkylidenecyclopropanes or alkenes.^4^ In subsequent accounts, the authors reported a sequential 5-*exo-dig* cyclization of malonates and β-ketoesters tethered to the alkyne moiety, followed by coupling with aryl iodides.^5^ The scope of the methodology was further extended to the use of haloalkynes,^6^ allyl halides and acetates^7^ as coupling partners. Recently, we have developed a protocol enabling the effective reaction of much less active aryl bromides with acetylenic β-ketoesters.^8^ A similar strategy, utilizing a 5-*endo-dig* cyclization has also been applied to the synthesis of cyclopentenes^9^ and indenes.^10^ Propargylmalonates led to substituted cyclopropanes *via* analogous cyclization/coupling protocol.^11^ On the other hand, propargyl-β-ketoesters underwent 5-*exo-dig* oxocyclization/coupling, leading to the formation of substituted furan systems due to ambident nature of enolates of β-ketoesters.^12^ Interestingly, the analogous transformation involving homopropargyl-β-ketoesters possessing an internal or terminal alkyne motif clearly led to either cyclopentenes^9^ or dihydropyranes,^13^ respectively.

The vast majority of the known methodologies utilizing sequential Pd-catalyzed nucleophilic cyclization and cross coupling are limited to aryl iodides. Moreover, the functional group compatibility appeared very narrow, which could possibly arise from the use of a strong base. Recently, we have addressed these challenges in a transformation involving acetylenic β-ketoesters which readily undergo cyclization. Extension of the scope with respect to activated methylene compounds still awaits investigation. Although there are examples of such transformations involving derivatives of ketoesters and malonates (with active aryl iodides), to the best of our knowledge, cyclization/coupling of haloarenes with acetylenic derivatives of malononitrile, cyanoacetates, diketones, as well as substrates bearing organophosphorus electron-withdrawing functions have not been reported.

Here, we report an efficient protocol for tandem Pd-catalyzed intramolecular addition of active methylene compounds to alkynes and subsequent cross-coupling with (hetero)aryl bromides and chlorides. The methodology features excellent tolerance for functionalities present in either reaction partner.

## Results and discussion

The reaction of dimethyl pent-4-yn-1-ylmalonate 1 with bromobenzene was chosen as a model transformation for the development of the reaction conditions. First, a range of Pd-complexes of mono- and diphosphine ligands were examined using 3rd-generation Buchwald-type palladacyclic system as a platform in order to identify an active catalyst system. Optimization revealed XPhos Pd G3 as the pre-catalyst of choice. Then, the benchmark reaction was evaluated against various reaction conditions, including base, solvent, catalyst loading, temperature, and time, among others (Table 1).^14^ A polar aprotic solvent appeared to be crucial for the efficiency of the cyclization. Reactions carried out in moderately polar, or nonpolar solvents (*e.g.* dioxane, THF, toluene) failed to proceed at all, or competitive Sonogashira coupling was observed. The best results were achieved for the reaction run for 24 h at 50 °C in DMF with potassium phosphate as the base. 2 mol% of palladium complex was necessary to achieve a high yield of desired product 2.

**Table 1:** Optimization of the reaction conditions for benchmark reaction

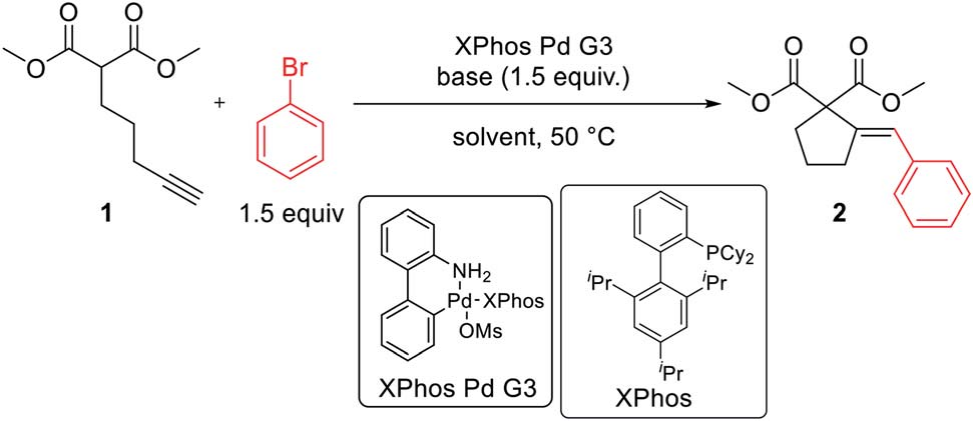					
Entry	Solvent	Base	Time	Cat. loading	Yield^a^
1	Toluene	K_3_PO_4_	4 h	1 mol%	1%
2	Dioxane	K_3_PO_4_	4 h	1 mol%	3%
3	THF	K_3_PO_4_	4 h	1 mol%	2%
4	MeCN	K_3_PO_4_	4 h	1 mol%	8%
5	DMSO	K_3_PO_4_	4 h	1 mol%	47%
6	DMF	*t*-BuOK	4 h	1 mol%	0%
7	DMF	KHMDS	4 h	1 mol%	0%
8	DMF	K_2_CO_3_	4 h	1 mol%	22%
9	DMF	K_3_PO_4_	4h	1 mol%	22%
10	DMF	K_3_PO_4_	4 h	1 mol%	61%
11	DMF	K_3_PO_4_	24 h	2 mol%	90%

a Determined by GC with mesitilene as an internal standard.

With satisfactory conditions developed for the model substrate, we proceeded to investigate the scope of the reaction. First, we examined the performance of various aryl and heteroaryl bromides in the reaction with malonate 1 (Table 2).

**Table 2:** Substrate scope: aryl bromides^a^

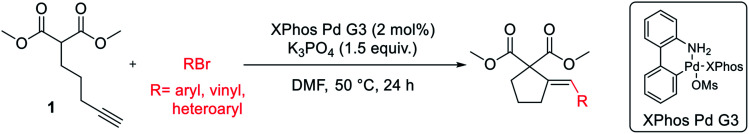
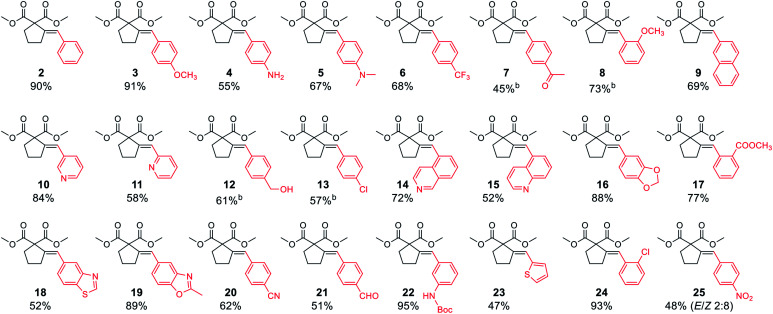

a Reaction conditions: dimethyl pent-4-yn-1-ylmalonate 1 (0.400 mmol), aryl bromide (0.500 mmol), K_3_PO_4_ (0.600 mmol), XPhos Pd G3 (8.0 μmol, 2 mol%), DMF (1 ml), 50 °C, 24 h.

b Run for 4 h.

Both electron-rich and electron-poor bromoarenes smoothly underwent the reaction, affording the expected products with good to excellent yields and complete stereoselectivity on the olefinic bond. A range of functional groups including, *inter alia*, unprotected amines (4), alcohols (12), aldehydes (21), nitriles (20), nitro (25), carbamates (22), or enolizable ketones (7) were well tolerated. Furthermore, sterically hindered *o*-substituted bromo(hetero)arenes also proved to be complementary reaction partners (8, 17, 24). The use of various heteroaryl bromides enabled the introduction of the heterocyclic moiety to the product (10–11, 14–16, 18–19, 23), including pharmaceutically relevant N-heterocyclic motifs (10–11, 14–16, 18–19).

Next, we proceeded to examine the scope and limitations with respect to various acetylenic active methylene compounds (Table 3). Selected derivatives of malonates, cyanoacetates, cyanomalonates, β-ketoesters, and 1,3-diketones were subjected to the reaction with both electron-poor and electron-rich bromoarenes – bromobenzene, *p*-bromoanisole, and *p*-bromobenzonitrile. Transformations with more sterically hindered *i*-propyl and *t*-butyl malonates delivered the expected products (26–31), although with diminished yields, compared to the less sterically demanding methyl malonate 1.

**Table 3:** Substrate scope: acetylenic active methylene compounds^a^

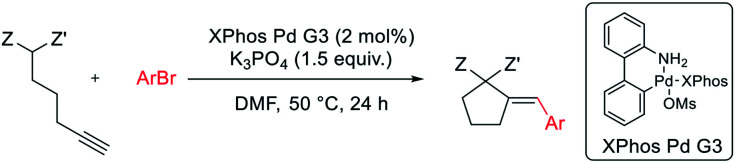
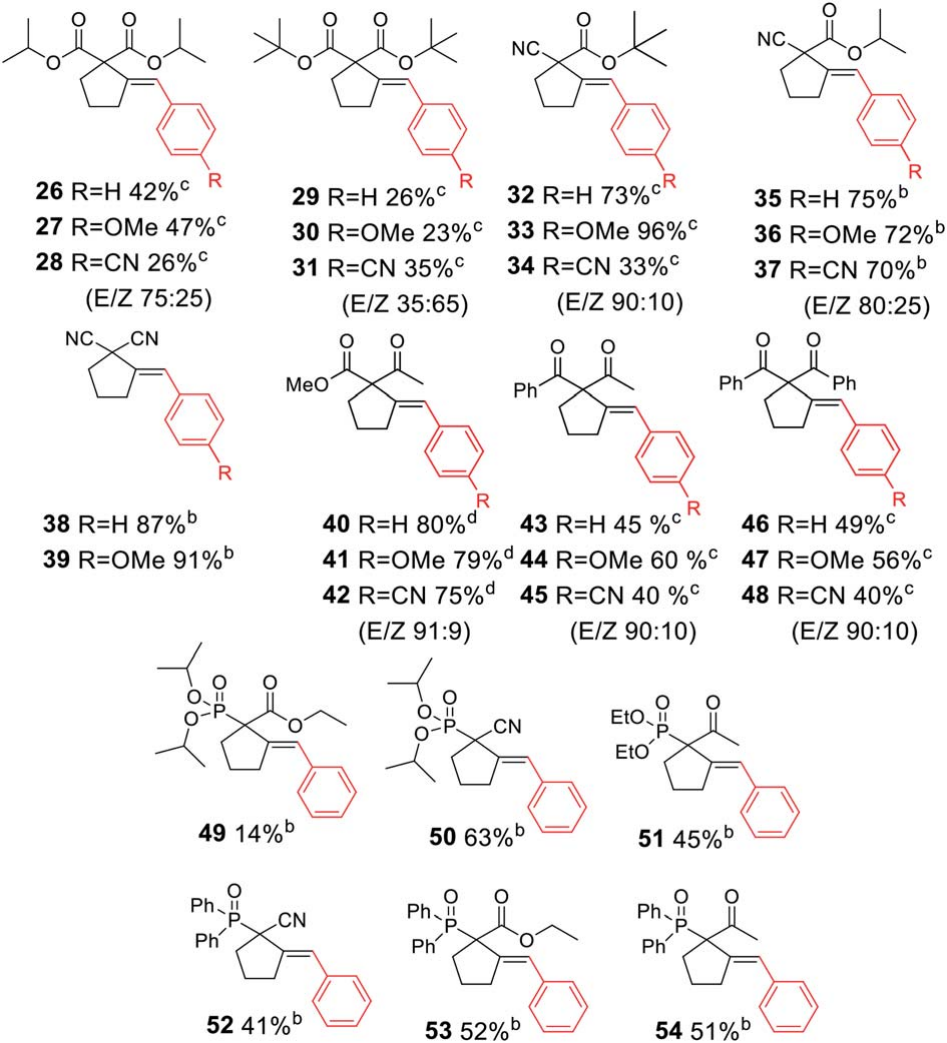

a Reaction conditions: acetylenic active methylene compound (0.400 mmol), aryl bromide (0.500 mmol), K_3_PO_4_ (0.600 mmol), XPhos Pd G3 (8.0 μmol, 2 mol%), DMF (1 ml), 50 °C, 24 h.

b Run for 4 h.

c Run at 80 °C for 24 h.

d Run at 50 °C for 2 h.

The considerably more C–H acidic cyanoacetates, cyanomalonates, and β-ketoesters appeared to be the more reactive substrates, usually providing the appropriate products (32–43) with very good yields (70–96%). The only exception was a reaction of electron-deficient bromoarenes with cyanomalonate and *t*-butyl cyanoacetate, which afforded products (34 and 40) with moderate yields (33–40%). Notably, reactions involving electron-deficient bromoarenes and all of the above-mentioned acetylenic substrates proceeded with high, but not complete diastereoselectivity (*E*/*Z* selectivity). All reactions involving electronically neutral, or electron-rich bromoarenes provided complete selectivity.

Next, we investigated various phosphorus-substituted acetylenes as potential reaction partners. We were pleased to find that esters, ketones, and nitriles bearing phosphoryl or phosphinoyl functions entered the reaction with bromobenzene, affording the target cyclopentanes (49–54) with moderate to good yields and complete diastereoselectivity. Compound 49 was isolated with a low yield due to difficulties in the isolation and purification.

Finally, we were pleased to find that the developed protocol is also applicable to the remarkably less active aryl chlorides (Table 4). Both electron-rich and electron-deficient chloroarenes, as well as heteroaryl chlorides (2-chloropyridine) entered the reaction, yielding the expected products in moderate to good yields (39–69%). Interestingly, electron-deficient chloroarenes gave products with low diastereoselectivity, in contrast to their corresponding aryl bromides which provided the products as single isomers (except 4-nitrobromobenzene).

**Table 4:** Substrate scope: aryl chlorides^a^

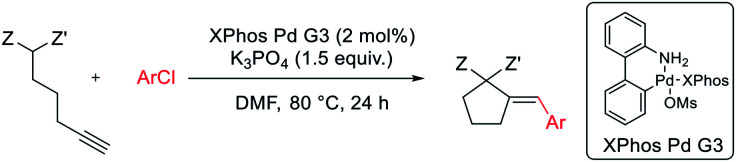
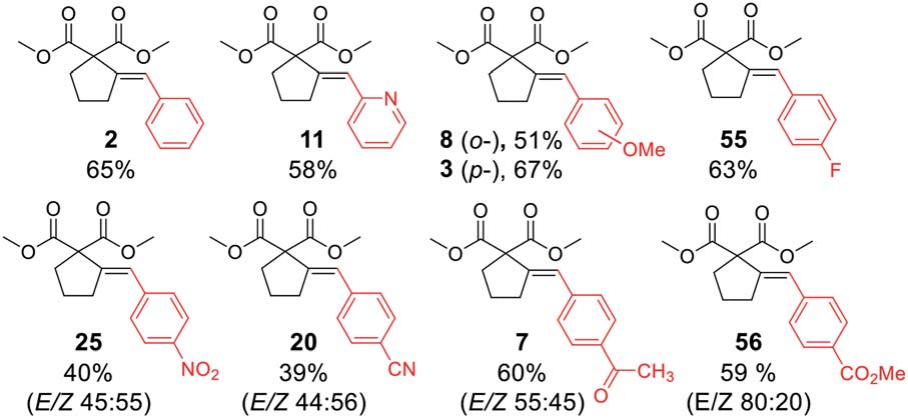

a Reaction conditions: dimethyl pent-4-yn-1-ylmalonate 1 (0.400 mmol), aryl chloride (0.500 mmol), K_3_PO_4_ (0.600 mmol), XPhos Pd G3 (8.0 μmol, 2 mol%), DMF (1 ml), 80 °C, 24 h.

**Scheme 1:**
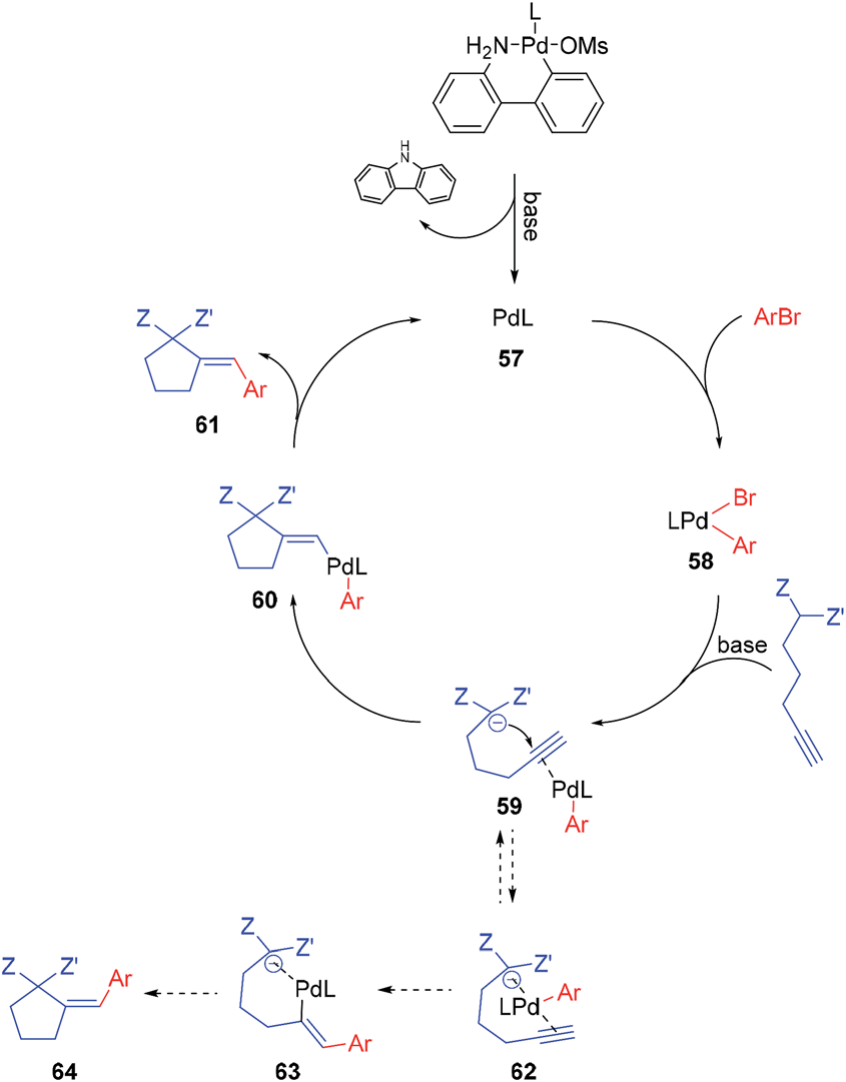
Plausible mechanism.

The postulated mechanism, based on the observations of the reaction outcome, several control experiments, and literature data, is depicted in Scheme 1. First, the bromoarene undergoes fast oxidative addition to Pd(0) complex 57 (formed upon the activation of the precatalyst with a base)^15^ leading to the formation of aryl–Pd(ii) species 58 which coordinates to the alkyne moiety. Then, intramolecular nucleophilic addition to the activated unsaturated system occurs, providing vinyl–Pd(ii) species 60 which undergoes facile reductive elimination affording the expected product 61 and reconstituting the Pd(0) complex 57. Although the above mechanism seems viable for the majority of the investigated reactions, for some specific combinations of substrates, alternative scenarios should also be considered. For instance, the formation of chelate 62 (possibly being in equilibrium with 59), in which palladium is bound by both alkyne and active methylene moieties, could facilitate the insertion of the Pd–arene to the alkyne (*syn*-carbometallation), and thus rationalize the formation of some amount of another diastereoisomer of the product with altered configuration at the exocyclic double bond (64).

**Scheme 2:**
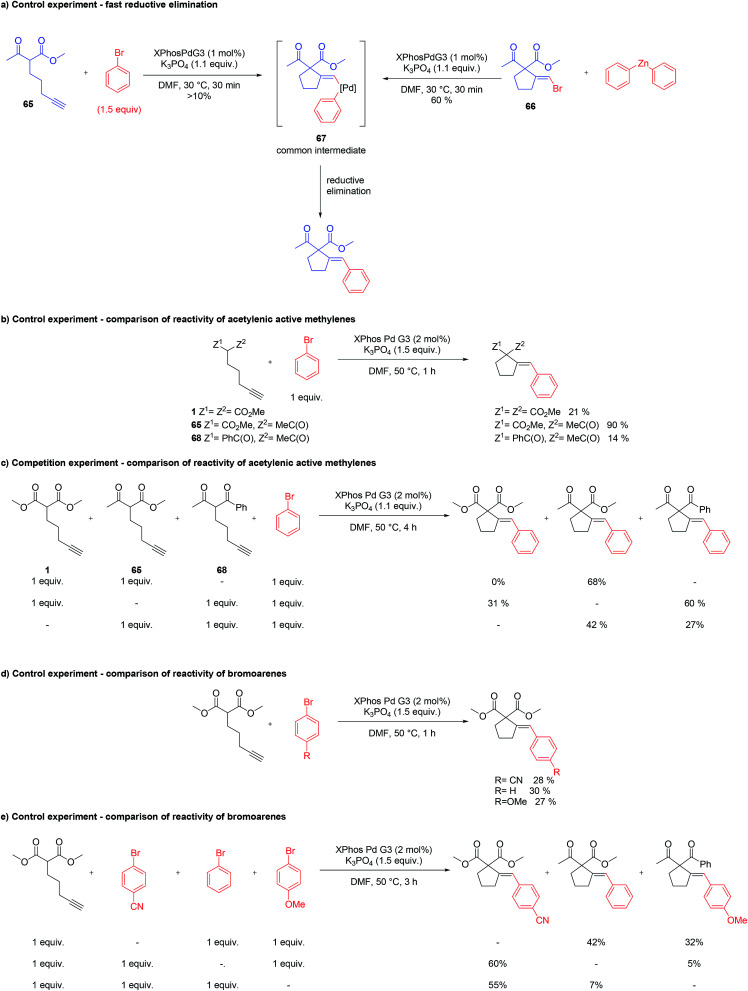
Control experiments.

Oxidative addition to Pd(0) ligated to a single electron-rich monophosphine is fast. In fact, oxidative addition of bromoarene to XPhos–Pd(0) complex proceeds within minutes at room temperature, as observed by ^31^P NMR spectroscopy. Reductive elimination from Pd complexes of sterically demanding ligands is also facile. In particular, we have recently shown that the reductive elimination is not a rate-limiting step in the XPhos–Pd-catalyzed tandem cyclization/coupling of ε-acetylenic β-ketoesters with aryl bromides (Scheme 2a).^8^ The tandem reaction of ketoester 65 with bromobenzene is much slower than Negishi coupling of compound 66 with diphenylzinc, both proceeding through reductive elimination from a common intermediate 67. This points towards the conclusion that the cyclization step is a bottleneck of the transformation. In order to shed more light on the influence of the structure of reagents on the reaction outcome, we compared the rate of reactions of bromobenzene with three acetylenic substrates – derivatives of malonate 1, β-ketoester 65, and β-diketone 68 (Scheme 2b). As expected, malonate 1 reacted significantly slower than ketoester 65, providing the corresponding product in only 21% yield after 1 h, compared to 90% for 65. This is due to considerably lower C–H acidity of the malonate. Surprisingly, under identical conditions, the more C–H acidic β-diketone 68 delivered the product with only 14% yield. Competition experiments, involving pairs of acetylenic substrates (1 equiv. of each) and bromobenzene (1 equiv.) were also conducted (Scheme 2c). A reaction involving ketoester 65 and malonate 1 delivered only the product of the cyclization/coupling of 65, demonstrating the huge difference in their reactivity. Despite diketone 68 reacting slower than malonate in a parallel experiment (see: Scheme 2b), in the competition experiment it provided higher yield of the corresponding product (60% and 31%, respectively). Similarly, the cyclization of ketoester and diketone occurred at comparable rates under the competition conditions (42% and 27%, respectively), in contrast to the parallel experiment (90% *vs.* 14%). The remarkably slow reaction of diketone 68 could be attributed either to the lower nucleophilicity of its enolate due to extended resonance stabilization, or the capability for the formation of stable complexes with palladium.^16^ The relatively stable palladium complex with diketone (or its anion) could possibly be in tautomeric equilibrium with Pd–alkyne complex suitable for intramolecular nucleophilic addition leading to 61. Thus, the involvement of arylpalladium 58 in complexation with diketone 68 could make it less available for the catalytic transformation of the more reactive ketoester 65 in the competition experiment.

Competition experiments of malonate 1 with pairs of electronically divergent bromoarenes revealed the preference for the reaction with the more electron-deficient substrate (Scheme 2e). This stays in contrast with the outcome of the parallel experiments of 1 with each of the above bromoarenes showing comparable rates (Scheme 2d). Apparently, oxidative addition is not a rate limiting step, although in control experiments it determines the ratio of aryl–Pd(ii) intermediates, which in turn dictates the final product distribution.

Another factor used for better understanding the reaction mechanism is the stereochemical outcome of the transformation. All of the reactions with malonates proceeded with complete diastereoselectivity, arising from *anti*-carbopalladation of the alkyne moiety. Similarly, other acetylenic active methylene compounds delivered the corresponding products as single isomers, unless electron-deficient bromoarenes (*e.g. p*-bromobenzonitrile) were used as coupling partners. In this case, the isomer with the alternate configuration on the double bond was formed to some extent, suggesting an alternative pathway for these sets of substrates (Scheme 1, dashed lines).

## Experimental

All manipulations were performed in a nitrogen-filled glovebox or under an argon atmosphere using Schlenk techniques, unless mentioned otherwise. Flash chromatography was performed using Merck silica gel 60 (230–400 mesh). TLC analysis of reaction mixtures was performed on Merck silica gel 60 F254 TLC plates and visualized with cerium molybdate stain (Hanessian's stain). ^1^H, ^13^C{1H}, and ^19^F NMR spectra were recorded with a Bruker AV 400 spectrometer. ^1^H and ^13^C chemical shifts are given in ppm relative to TMS. Solvent signals were used as references (CDCl_3_*δ*_H_ = 7.26 ppm, *δ*_C_ = 77.0 ppm) and the chemical shift converted to the TMS scale. Coupling constants (*J*) are reported in Hz, and the following abbreviations were used to denote multiplets: s = singlet, d = doublet, t = triplet, q = quartet, quint = quintet, m = multiplet (denotes a complex pattern), dd = doublet of doublets, dt = doublet of triplets and br = broad signal. Infrared spectra were recorded with a Jasco FTIR-6200 spectrometer. Electron ionization high-resolution mass spectra (EI-HR) were recorded with an Autospec Premier (Waters Inc) mass spectrometer using the narrow-range high-voltage scan technique with low-boiling perfluorokerosene (PFK) as internal standard. Samples were introduced by using a heated direct insertion probe. Electrospray ionization high-resolution mass spectra (ESI-HR) were recorded with MALDISynapt G2-S HDMS (Waters Inc) mass spectrometer equipped with an electrospray ion source and q-TOF type mass analyzer. ESI-MS spectra were recorded in the positive ion mode (source parameters: capillary voltage 3.15 kV, sampling cone 25 V, source temperature 120 °C, desolvation temperature 150 °C).

Unless otherwise noted, all commercially available compounds (ABCR, Acros, Fluorochem, TCI, Sigma-Aldrich, Strem) were used as received. Phosphine ligands were purchased from Aldrich or Fluorochem, Pd(OAc)_2_ was purchased from Strem. Buchwald-type 3rd-generation palladacyclic precatalysts (Ligand Pd G3) were prepared following literature procedures,^15^ and showed similar reactivity to the commercial samples (XPhos Pd G3 was compared with commercial samples). Dimethyl pent-4-yn-1-ylmalonate 1 and other acetylenic active methylene compounds were synthesized by alkylation of dimethyl malonate or other C–H acids with 1-iodo-pentyne, according to typical literature procedures.

### General procedure A for Pd-catalyzed carbocyclization-coupling of aryl bromides with acetylenic active methylene compounds

In a drybox, a 4 ml screw-cap vial was charged with XPhos Pd G3 (6.8 mg, 8 μmol), aryl halide (0.5 mmol), K_3_PO_4_ (127.2 mg, 0.6 mmol), DMF (1 ml), and a magnetic stirring bar. Then, acetylenic active methylene compound (*e.g.* dimethyl pent-4-yn-1-ylmalonate 1) was added (0.4 mmol), the vial was tightly sealed and removed from drybox. The reaction mixture was stirred for 24 h at 50 °C in a heating block, then cooled to room temperature, quenched with 20 ml of an NH_4_Cl solution, added to 10 ml of water, and extracted with MTBE (3 × 10 ml). The combined organic phases were dried with Na_2_SO_4_, filtered, and concentrated. The crude product was purified by column chromatography on silica gel.

### Dimethyl (2*E*)-2-benzylidenecyclopentane-1,1-dicarboxylate (2)

Prepared in reaction of dimethyl 4-pentenylmalonate and bromobenzene following general procedure (105 mg, 90%) or in reaction with chlorobenzene following modified general procedure (run at 80 °C) (71 mg, yield 65%). Product was isolated as oil after column chromatography on silica gel (15 g, hex/AcOEt 95 : 5). ^1^H NMR (400 MHz, CDCl_3_) *δ* 7.37–7.30 (m, 4H), 7.24–7.19 (m, 1H), 6.71 (t, *J* = 2.4 Hz, 1H), 3.78 (s, 6H), 2.72 (td, *J* = 7.2, 2.5 Hz, 2H), 2.40 (t, *J* = 6.9 Hz, 2H), 1.84 (p, *J* = 7.1 Hz, 2H); ^13^C NMR (101 MHz, CDCl_3_) *δ* 171.4, 141.0, 137.6, 128.7, 128.2, 127.4, 126.8, 65.4, 52.8, 35.7, 32.0, 24.8; IR (CH_2_Cl_2_): 3053, 3024, 2953, 2878, 2842, 1733, 1431, 1263, 1152, 773, 696 cm^−1^; HRMS (ESI) *m*/*z* calcd for C_16_H_18_O_4_Na 297.1103; found 297.1097.

### Dimethyl (2*E*)-2-(4-methoxybenzylidene)cyclopentane-1,1-dicarboxylate (3)

Prepared in reaction of dimethyl 4-pentenylmalonate and 4-bromoanisole following general procedure (110 mg, yield 91%) or in reaction with 4-chloroanisole following modified general procedure (run at 80 °C) (81 mg, yield 67%). Product was isolated as oil after column chromatography on silica gel (15 g, hex/AcOEt 80 : 20). ^1^H NMR (400 MHz, CDCl_3_) *δ* 7.31–7.26 (m, 2H), 6.89–6.84 (m, 2H), 6.63 (t, *J* = 2.6 Hz, 1H), 3.80 (s, 3H), 3.76 (s, 6H), 2.69 (td, *J* = 7.2, 2.6 Hz, 2H), 2.38 (t, *J* = 6.9 Hz, 2H), 1.83 (p, *J* = 7.0 Hz, 2H); ^13^C NMR (101 MHz, CDCl_3_) *δ* 171.5, 158.4, 138.7, 130.4, 129.9, 126.8, 113.6, 65.3, 55.2, 52.7, 35.7, 31.9, 24.8; IR (CH_2_Cl_2_): 2954, 2838, 1732, 1606, 1512, 1435, 1251, 1177, 1033, 826 cm^−1^; HRMS (ESI): *m*/*z* calcd for C_17_H_20_O_5_Na 327.1208; found 327.1196.

### Dimethyl (2*E*)-2-(4-aminobenzylidene)cyclopentane-1,1-dicarboxylate (4)

Prepared in reaction of dimethyl 4-pentenylmalonate and 4-bromoaniline following general procedure (63 mg, yield 55%). Product was isolated as oil after column chromatography on silica gel (15 g, hex/AcOEt 80 : 20 → 70 : 30) ^1^H NMR (400 MHz, CDCl_3_) *δ* 7.16 (d, *J* = 8.4 Hz, 2H), 6.64 (d, *J* = 8.4 Hz, 2H), 6.57 (t, *J* = 2.3 Hz, 1H), 3.75 (s, 6H), 2.68 (td, *J* = 7.2, 2.4 Hz, 2H), 2.36 (t, *J* = 7.0 Hz, 2H), 1.82 (p, *J* = 7.1 Hz, 2H); ^13^C NMR (101 MHz, CDCl_3_) *δ* 171.7, 145.3, 137.2, 129.9, 128.2, 127.2, 114.7, 65.3, 52.7, 35.8, 31.9, 24.9; IR (CH_2_Cl_2_): 3467, 3378, 2953, 1728, 1623, 1516, 1264, 1180, 1153, 825, 526 cm^−1^; MS (EI): *m*/*z* (%) = 290(21), 289(67)[M^+^], 231(29), 230(100), 229(21), 202(19), 171(30), 170(94), 143(21), 106(22), 73(38), 57(28), 55(17), 43(23); HRMS (EI): *m*/*z* calcd for C_16_H_19_NO_4_ 289.1314; found 289.1316.

### Dimethyl (2*E*)-2-(4-(dimethylamino)benzylidene)cyclopentane-1,1-dicarboxylate (5)

Prepared in reaction of dimethyl 4-pentenylmalonate and 4-bromo-*N*,*N*-dimethylaniline following general procedure (85 mg, yield 67%). Product was isolated as oil after column chromatography on silica gel (15 g, hex/AcOEt 90 : 10 → 70 : 30). ^1^H NMR (400 MHz, CDCl_3_) *δ* 7.29–7.24 (m, 2H), 6.70 (d, *J* = 8.8 Hz, 2H), 6.60 (t, *J* = 2.3 Hz, 1H), 3.76 (s, 6H), 2.96 (s, 6H), 2.72 (td, *J* = 7.2, 2.4 Hz, 2H), 2.38 (t, *J* = 7.0 Hz, 2H), 1.83 (p, *J* = 7.1 Hz, 2H); ^13^C NMR (101 MHz, CDCl_3_) *δ* 171.8, 149.3, 136.5, 129.7, 127.2, 126.2, 112.1, 65.3, 52.6, 40.4, 35.8, 31.9, 24.9; IR (CH_2_Cl_2_): 2952, 2881, 2804, 1730, 1608, 1522, 1434, 1355, 1247, 1162, 1064, 813, 530 cm^−1^; MS (EI): *m*/*z* (%) = 318(21), 317(69)[M^+^], 259(29), 258(100), 199(20), 198(49), 171(12), 153(9), 134(13), 77(5), 59(7); HRMS (EI): *m*/*z* calcd for C_18_H_23_NO_4_ 317.1627; found 317.1636.

### Dimethyl (2*E*)-2-(4-(trifluoromethyl)benzylidene)cyclopentane-1,1-dicarboxylate (6)

Prepared in reaction of dimethyl 4-pentenylmalonate and 4-bromobenzotrifluoride following general procedure (91 mg, yield 67%) or in reaction with 4-chlorobenzotrifluoride following modified general procedure (run at 80 °C) (76 mg, yield 56%). Product was isolated as oil after column chromatography on silica gel (15 g, hex/AcOEt/DCM 86 : 9.5 : 0.5). ^1^H NMR (400 MHz, CDCl_3_*δ* 7.58 (d, *J* = 8.2 Hz, 2H), 7.43 (d, *J* = 8.2 Hz, 2H), 6.74 (s, 1H), 3.78 (s, 6H), 2.71 (td, *J* = 7.2, 2.5 Hz, 2H), 2.41 (t, *J* = 7.0 Hz, 2H), 1.85 (p, *J* = 7.1 Hz, 2H); ^13^C NMR (101 MHz, CDCl_3_) *δ* 171.1, 143.8, 141.1, 128.8, 128.6 (q, *J* = 32.5 Hz), 126.3, 125.0 (q, *J* = 3.6 Hz), 124.2 (q, *J* = 271.8 Hz) 65.5, 52.9, 35.6, 32.1, 24.7; ^19^F NMR (376 MHz, CDCl_3_) *δ* −62.52; IR (CH_2_Cl_2_): 2956, 1735, 1615, 1435, 1327, 1265, 1125, 1068, 830, 598 cm^−1^; MS (EI): *m*/*z* (%) = 343(12), 342(39)[M^+^], 323(18), 310(24), 283(40), 282(61), 252(25), 251(83), 250(41), 224(27), 223(100), 159(37), 77(14), 59(38); HRMS (EI): *m*/*z* calcd for C_17_H_17_F_3_O_4_ 342.1079; found 342.1084.

### Dimethyl (2*E*)-2-(4-acetylbenzylidene)cyclopentane-1,1-dicarboxylate (7)

Prepared in reaction of dimethyl 4-pentenylmalonate and 4-bromoacetophenone following general procedure (57 mg, yield 45%) or in reaction with 4-chloroacetophenone following modified general procedure (run at 80 °C) (75 mg, yield 60%, isomer *E*/*Z* 55 : 45). Product was isolated as oil after column chromatography on silica gel (15 g, hex/AcOEt 90 : 10 → 80 : 20). ^1^H NMR (400 MHz, CDCl_3_): *δ* 7.92–7.88 (m, 2H), 7.43–7.38 (m, 2H), 6.73 (t, *J* = 2.6 Hz, 1H), 3.76 (s, 6H), 2.71 (td, *J* = 7.2, 2.6 Hz, 2H), 2.57 (s, 3H), 2.39 (t, *J* = 6.9 Hz, 2H), 1.84 (p, *J* = 7.1 Hz, 2H); ^13^C NMR (101 MHz, CDCl_3_): *δ* 197.5, 171.0, 144.0, 142.2, 135.2, 128.7, 128.2, 126.6, 65.6, 52.8, 35.6, 32.2, 26.5, 24.7; IR (CH_2_Cl_2_): 2954, 1732, 1682, 1602, 1435, 1360, 1268, 590 cm^−1^; HRMS (ESI): *m*/*z* calcd for C_18_H_20_O_5_Na 339.1208; found: 339.1201.

### Dimethyl (2*E*)-2-(2-methoxybenzylidene)cyclopentane-1,1-dicarboxylate (8)

Prepared in reaction of dimethyl 4-pentenylmalonate and 2-bromoanisole following general procedure (88 mg, yield 73%) or in reaction with 2-chloroanisole following modified general procedure (run at 80 °C) (63 mg, yield 51%). Product was isolated as oil after column chromatography on silica gel (15 g, hex/AcOEt 85 : 15). ^1^H NMR (400 MHz, CDCl_3_) *δ* 7.34 (dd, *J* = 7.6, 1.7 Hz, 1H), 7.23–7.17 (m, 1H), 6.97 (t, *J* = 2.6 Hz, 1H), 6.92 (td, *J* = 7.5, 1.2 Hz, 1H), 6.85 (dd, *J* = 8.3, 1.1 Hz, 1H), 3.81 (s, 3H), 3.78 (s, 6H), 2.64 (td, *J* = 7.2, 2.6 Hz, 2H), 2.39 (t, *J* = 6.9 Hz, 2H), 1.80 (p, *J* = 7.1 Hz, 2H); ^13^C NMR (101 MHz, CDCl_3_) *δ* 171.5, 157.0, 140.5, 128.9, 128.1, 126.7, 122.3, 120.0, 110.5, 64.9, 55.5, 52.6, 35.7, 31.8, 24.7; IR (CH_2_Cl_2_): 2953, 2839, 1732, 1597, 1487, 1461, 1436, 1248, 1136, 755 cm^−1^; HRMS (ESI): *m*/*z* calcd for C_17_H_20_O_5_Na 327.1208; found 327.1203.

### Dimethyl (2*E*)-2-(naphthalen-2-ylmethylidene)cyclopentane-1,1-dicarboxylate (9)

Prepared in reaction of dimethyl 4-pentenylmalonate and 2-bromonaphthalene following general procedure (90 mg, yield 69%). Product was isolated as oil after column chromatography on silica gel (15 g, hex/AcOEt 90 : 10). ^1^H NMR (400 MHz, CDCl_3_) *δ* 7.84–7.79 (m, 4H), 7.52 (dd, *J* = 8.5, 1.4 Hz, 1H), 7.48–7.44 (m, 2H), 6.90 (t, *J* = 2.3 Hz, 1H), 3.82 (s, 6H), 2.84 (td, *J* = 7.2, 2.5 Hz, 2H), 2.46 (t, *J* = 7.0 Hz, 2H), 1.89 (p, *J* = 7.0 Hz, 2H); ^13^C NMR (101 MHz, CDCl_3_) *δ* 171.4, 141.5, 135.1, 133.3, 132.23, 128.0, 127.6, 127.6, 127.5, 126.8, 126.0, 125.8, 65.5, 52.8, 35.7, 32.1, 24.8; IR (CH_2_Cl_2_): 3053, 2953, 2879, 1732, 1434, 1262, 1065, 1016, 817, 748, 477 cm^−1^; HRMS (ESI): *m*/*z* calcd for C_20_H_20_O_4_Na 347.1248; found 347.1259.

### Dimethyl (2*E*)-2-(pyridin-3-ylmethylidene)cyclopentane-1,1-dicarboxylate (10)

Prepared in reaction of dimethyl 4-pentenylmalonate and 3-bromopyridine following general procedure (92 mg, yield 84%). Product was isolated as oil after column chromatography on silica gel (15 g, hex/AcOEt 80 : 20 → 70 : 30). ^1^H NMR (400 MHz, CDCl_3_) *δ* 8.56 (d, *J* = 2.0 Hz, 1H), 8.41 (dd, *J* = 4.8, 1.5 Hz, 1H), 7.61 (dt, *J* = 7.9, 1.7 Hz, 1H), 7.24–7.19 (m, 1H), 6.64 (t, *J* = 2.4 Hz, 1H), 3.75 (s, 6H), 2.67 (td, *J* = 7.2, 2.6 Hz, 2H), 2.37 (t, *J* = 7.0 Hz, 2H), 1.82 (p, *J* = 7.0 Hz, 2H); ^13^C NMR (101 MHz, CDCl_3_) *δ* 171.0, 150.0, 147.7, 143.6, 135.1, 133.2, 124.0, 123.0, 65.4, 52.8, 35.6, 32.0, 24.7; IR (CH_2_Cl_2_): 3027, 2953, 2879, 1732, 1567, 1434, 1266, 1065, 1021, 804, 710 cm^−1^; HRMS (ESI): *m*/*z* calcd for C_15_H_17_NO_4_ 276.1212; found 276.1232.

### Dimethyl (2*E*)-2-(pyridin-2-ylmethylidene)cyclopentane-1,1-dicarboxylate (11)

Prepared in reaction of dimethyl 4-pentenylmalonate and 2-bromopyridine following general procedure (64 mg, yield 58%) or in reaction with 2-chloropyridine following modified general procedure (run at 80 °C) (60 mg, yield 55%). Product was isolated as oil after column chromatography on silica gel (15 g, hex/AcOEt 80 : 20 → 70 : 30). ^1^H NMR (400 MHz, CDCl_3_) *δ* 8.57 (dd, *J* = 4.8, 1.9, 0.9 Hz, 1H), 7.59 (td, *J* = 7.7, 1.9 Hz, 1H), 7.28–7.20 (m, 1H), 7.07–7.02 (m, 1H), 6.74 (t, *J* = 2.7 Hz, 1H), 3.74 (s, 6H), 2.94 (td, *J* = 7.3, 2.6 Hz, 2H), 2.37 (t, *J* = 6.9 Hz, 2H), 1.82 (p, *J* = 7.1 Hz, 2H); ^13^C NMR (101 MHz, CDCl_3_) *δ* 171.1, 156.4, 149.1, 146.1, 135.8, 126.6, 124.07, 121.0, 65.8, 52.8, 35.6, 32.7, 24.6; IR (CH_2_Cl_2_): 3050, 2954, 2280, 1732, 1584, 1438, 1433, 1263, 1151, 738, 747 cm^−1^; HRMS (ESI): *m*/*z* calcd for C_15_H_17_NO_4_ 276.1236; found 276.1223.

### Dimethyl (2*E*)-2-(4-(hydroxymethyl)benzylidene)cyclopentane-1,1-dicarboxylate (12)

Prepared in reaction of dimethyl 4-pentenylmalonate and 4-bromobenzyl alcohol following general procedure (72 mg, yield 60%). Product was isolated as oil after column chromatography on silica gel (15 g, hex/AcOEt 60 : 40). ^1^H NMR (400 MHz, CDCl_3_) *δ* 7.34–7.27 (m, 4H), 6.67 (t, *J* = 2.5 Hz, 1H), 4.64 (s, 2H), 3.75 (s, 6H), 2.68 (td, *J* = 7.2, 2.6 Hz, 2H), 2.37 (t, *J* = 6.9 Hz, 2H), 2.11 (s, 1H), 1.82 (p, *J* = 7.1 Hz, 2H); ^13^C NMR (101 MHz, CDCl_3_) *δ* 171.4, 141.0, 139.5, 136.9, 128.8, 127.1, 126.7, 65.4, 64.8, 52.8, 35.7, 32.0, 24.7; IR (CH_2_Cl_2_): 3426, 2953, 2877, 1730, 1435, 1265, 1163, 1013 cm^−1^; HRMS (ESI): *m*/*z* calcd for C_17_H_20_O_5_Na 327.1208; found 327.1205.

### Dimethyl (2*E*)-2-(4-chlorobenzylidene)cyclopentane-1,1-dicarboxylate (13)

Prepared in reaction of dimethyl 4-pentenylmalonate and 1-bromo-4-chlorobenzene following general procedure (70 mg, 57%). Product was isolated as oil after column chromatography on silica gel (15 g, hex/AcOEt 90 : 10). ^1^H NMR (400 MHz, CDCl_3_): *δ* 7.31–7.23 (m, 4H), 6.65 (t, *J* = 2.5 Hz, 1H), 3.76 (s, 6H), 2.66 (td, *J* = 7.2, 2.6 Hz, 2H), 2.38 (t, *J* = 6.9 Hz, 2H), 1.88–1.80 (m, 2H); ^13^C NMR (101 MHz, CDCl_3_): *δ* 171.2, 141.7, 136.0, 132.5, 129.9, 128.3, 126.3, 65.4, 52.8, 35.7, 32.0, 24.7; IR (CH_2_Cl_2_): 2953, 1733, 1491, 1434, 1265, 821, 519 cm^−1^; HRMS (ESI): *m*/*z* calcd for C_16_H_17_O_4_ClNa 331.0713; found 331.0706.

### Dimethyl (2*E*)-2-(isoquinolin-5-ylmethylidene)cyclopentane-1,1-dicarboxylate (14)

Prepared in reaction of dimethyl 4-pentenylmalonate and 5-bromoisoquinoline following general procedure (93 mg, yield 72%). Product was isolated as oil after column chromatography on silica gel (15 g, hex/AcOEt 70 : 30 → 60 : 40). ^1^H NMR (400 MHz, CDCl_3_) *δ* 9.21 (s, 1H), 8.53 (d, *J* = 6.0 Hz, 1H), 7.82 (dd, *J* = 15.3, 6.9 Hz, 2H), 7.61–7.52 (m, 2H), 7.19 (s, 1H), 3.82 (s, 6H), 2.49–2.42 (m, 4H), 1.75 (p, *J* = 7.0 Hz, 2H); ^13^C NMR (101 MHz, CDCl_3_) *δ* 171.2, 152.7, 144.2, 143.2, 134.3, 134.1, 129.5, 128.6, 126.7, 126.5, 123.7, 117.5, 64.6, 52.9, 35.9, 31.6, 24.4; IR (CH_2_Cl_2_): 2953, 1732, 1617, 1584, 1434, 1261, 1152, 832, 762, 475 cm^−1^; MS (EI): *m*/*z* (%) = 326(12), 325(57)[M^+^], 275(9), 267(4), 234(75), 207(30), 206(100), 204(26), 156(13), 142(14), 98(2), 77(6), 43(12); HRMS (EI): *m*/*z* calcd for C_19_H_19_NO_4_ 325.1314, found: 325.1317.

### Dimethyl (2*E*)-2-(quinolin-5-ylmethylidene)cyclopentane-1,1-dicarboxylate (15)

Prepared in reaction of dimethyl 4-pentenylmalonate and 5-bromoquinoline following general procedure (68 mg, yield 52%). Product was isolated as oil after column chromatography on silica gel (15 g, hex/AcOEt 70 : 30 → 60 : 40). ^1^H NMR (400 MHz, CDCl_3_) *δ* 8.88 (dd, *J* = 4.2, 1.6 Hz, 1H), 8.41–8.36 (m, 1H), 7.99 (d, *J* = 8.4 Hz, 1H), 7.67–7.62 (m, 1H), 7.44–7.37 (m, 2H), 7.18 (s, 1H), 3.81 (s, 6H), 2.44 (td, *J* = 7.0, 2.1 Hz, 4H), 1.73 (p, *J* = 7.0 Hz, 2H); ^13^C NMR (101 MHz, CDCl_3_) *δ* 171.3, 150.1, 148.3, 144.1, 135.4, 133.2, 128.7, 128.6, 126.8, 126.0, 124.1, 120.9, 64.5, 52.9, 35.9, 31.5, 24.4; IR (CH_2_Cl_2_): 2952, 1731, 1593, 1572, 1434, 1254, 1148, 806 cm^−1^; MS (EI): *m*/*z* (%) = 326(10), 325(41)[M^+^], 265(23), 248(20), 235(27), 234(73), 207(35), 206(100), 204(36), 152(9), 142(24), 59(11); HRMS (EI): *m*/*z* calcd for C_19_H_19_NO_4_ 325.1314; found 325.1312.

### Dimethyl (2*E*)-2-(1,3-benzodioxol-5-ylmethylidene)cyclopentane-1,1-dicarboxylate (16)

Prepared in reaction of dimethyl 4-pentenylmalonate and 4-bromo-1,2-methylenedioxybenzene following general procedure (112 mg, yield 88%). Product was isolated as oil after column chromatography on silica gel (15 g, hex/AcOEt 70 : 30 → 50 : 50). ^1^H NMR (400 MHz, CDCl_3_) *δ* 6.87 (s, 1H), 6.83–6.74 (m, 2H), 6.59 (t, *J* = 2.3 Hz, 1H), 5.93 (s, 2H), 3.76 (s, 6H), 2.67 (td, *J* = 7.1, 2.4 Hz, 2H), 2.37 (t, *J* = 6.9 Hz, 2H), 1.83 (p, *J* = 7.0 Hz, 2H); ^13^C NMR (101 MHz, CDCl_3_) *δ* 171.4, 147.5, 146.4, 139.3, 131.9, 127.1, 123.0, 108.6, 108.1, 100.9, 65.3, 52.7, 35.7, 31.9, 24.8; IR (CH_2_Cl_2_): 2963, 2890, 1730, 1491, 1442, 1254, 1038, 930, 809 cm^−1^; MS (EI): *m*/*z* (%) = 319(25), 318(81)[M^+^], 260(18), 259(63), 258(38), 231(29), 227(23), 200(31), 199(100), 169(36), 141(34), 135(30), 115(27), 77(13), 59(21); HRMS (EI): *m*/*z* calcd for C_17_H_18_O_6_ 318.1103; found 318.1095.

### Dimethyl (2*E*)-2-(2-(methoxycarbonyl)benzylidene)cyclopentane-1,1-dicarboxylate (17)

Prepared in reaction of dimethyl 4-pentenylmalonate and methyl 2-bromobenzoate following general procedure (102 mg, yield 77%). Product was isolated as oil after column chromatography on silica gel (15 g, hex/AcOEt 90 : 10 → 70 : 30).^1^H NMR (400 MHz, CDCl_3_) *δ* 7.89 (dd, *J* = 7.8, 1.4 Hz, 1H), 7.48–7.39 (m, 2H), 7.30–7.25 (m, 1H), 7.19 (t, *J* = 2.6 Hz, 1H), 3.85 (s, 3H), 3.80 (s, 6H), 2.49 (td, *J* = 7.2, 2.6 Hz, 2H), 2.38 (t, *J* = 6.9 Hz, 2H), 1.77 (p, *J* = 7.1 Hz, 2H); ^13^C NMR (101 MHz, CDCl_3_) *δ* 171.3, 167.5, 141.1, 138.9, 131.4, 130.3, 129.8, 129.3, 126.9, 126.7, 64.4, 52.7, 51.9, 35.8, 31.3, 24.6; IR (CH_2_Cl_2_): 2953, 1729, 1598, 1569, 1434, 1257, 1127, 1078, 777, 740 cm^−1^; MS (EI): *m*/*z* (%) = 332(8)[M^+^], 301(12), 300(24), 273(23), 268(12), 241(42), 240(100), 213(38), 182(27), 181(72), 153(32), 128(17), 115(18), 91(12), 77(14), 59(23); HRMS (EI): *m*/*z* calcd for C_18_H_20_O_6_ 332.1260; found 332.1255.

### Dimethyl (2*E*)-2-(1,3-benzothiazol-5-ylmethylidene)cyclopentane-1,1-dicarboxylate (18)

Prepared in reaction of dimethyl 4-pentenylmalonate and 5-bromobenzothiazole following general procedure (69 mg, yield 52%). Product was isolated as orange solid after column chromatography on silica gel (15 g, hex/AcOEt 75 : 25). ^1^H NMR (400 MHz, CDCl_3_) *δ* 8.97 (s, 1H), 8.11 (d, *J* = 1.7 Hz, 1H), 7.88 (d, *J* = 8.4 Hz, 1H), 7.41 (dd, *J* = 8.4, 1.7 Hz, 1H), 6.85 (t, *J* = 2.6 Hz, 1H), 3.78 (s, 6H), 2.78 (td, *J* = 7.2, 2.6 Hz, 2H), 2.41 (t, *J* = 6.9 Hz, 2H), 1.86 (p, *J* = 7.0 Hz, 2H); ^13^C NMR (101 MHz, CDCl_3_) *δ* 171.3, 154.3, 153.6, 141.9, 136.1, 132.0, 126.8, 126.8, 123.1, 121.3, 65.4, 52.8, 35.7, 32.1, 24.8; IR (CH_2_Cl_2_): 2952, 1731, 1540, 1438, 1264, 1153, 1065, 849 cm^−1^; MS (EI): *m*/*z* (%) = 332(21), 331(62)[M^+^], 272(10), 241(20), 240(62), 213(29), 212(100), 186(18), 152(14), 148(28), 59(14); HRMS (EI): *m*/*z* calcd for C_17_H_17_NO_4_S: 331.0878; found 331.0885.

### Dimethyl (2*E*)-2-((2-methyl-1,3-benzoxazol-5-yl)methylidene)cyclopentane-1,1-dicarboxylate(19)

Prepared in reaction of dimethyl 4-pentenylmalonate and 5-bromo-2-methyl-1,3-benzoxazole following general procedure (118 mg, yield 89%). Product was isolated as oil after column chromatography on silica gel (15 g, hex/AcOEt 70 : 30). ^1^H NMR (400 MHz, CDCl_3_) *δ* 7.61 (d, *J* = 2.0 Hz, 1H), 7.37 (d, *J* = 8.4 Hz, 1H), 7.23 (dd, *J* = 8.5, 1.8 Hz, 1H), 6.77 (t, *J* = 2.6 Hz, 1H), 3.75 (s, 6H), 2.70 (td, *J* = 7.2, 2.6 Hz, 2H), 2.59 (s, 3H), 2.37 (t, *J* = 6.9 Hz, 2H), 1.82 (p, *J* = 7.1 Hz, 2H); ^13^C NMR (101 MHz, CDCl_3_) *δ* 171.3, 164.2, 149.8, 141.6, 140.6, 134.1, 127.1, 125.9, 119.0, 109.6, 65.3, 52.7, 35.7, 31.9, 24.8, 14.4; IR (CH_2_Cl_2_): 3456, 2954, 1732, 1578, 1434, 1265, 919, 812 cm^−1^; MS (EI): *m*/*z* (%) = 330(15), 329(45)[M^+^], 269(37), 252(23), 238(59), 211(30), 210(100), 169(33), 146(28), 141(31), 115(23), 91(5), 77(9), 59(19); HRMS (EI): *m*/*z* calcd for C_18_H_19_NO_5_ 329.1263; found 329.1274.

### Dimethyl (2*E*)-2-(4-cyanobenzylidene)cyclopentane-1,1-dicarboxylate (20)

Prepared in reaction of dimethyl 4-pentenylmalonate and 4-bromobenzonitrile following general procedure (73 mg, yield 62%) or in reaction with 4-chlorobenzonitrile following modified general procedure (run at 80 °C) (47 mg, yield 39%, isomer *E*/*Z* 56 : 44). Product was isolated as oil after column chromatography on silica gel (15 g, hex/AcOEt 98 : 2 → 90 : 10). ^1^H NMR (400 MHz, CDCl_3_) *δ* 7.60 (d, *J* = 8.3 Hz, 2H), 7.41 (d, *J* = 8.3 Hz, 2H), 6.71 (t, *J* = 2.4 Hz, 1H), 3.76 (s, 6H), 2.68 (td, *J* = 7.2, 2.5 Hz, 2H), 2.39 (t, *J* = 6.9 Hz, 2H), 1.89–1.80 (m, 2H); ^13^C NMR (101 MHz, CDCl_3_) *δ* 170.8, 145.1, 142.0, 131.9, 129.1, 126.1, 118.9, 110.1, 65.6, 52.9, 35.5, 32.2, 24.7; IR (CH_2_Cl_2_): 2955, 2225, 1732, 1604, 1435, 1264, 1115, 826, 555 cm^−1^; HRMS (ESI): *m*/*z* calcd for C_17_H_17_NO_4_Na 322.1055; found: 322.1045.

### Dimethyl (2*E*)-2-(4-formylbenzylidene)cyclopentane-1,1-dicarboxylate (21)

Prepared in reaction of dimethyl 4-pentenylmalonate and 4-bromobenzaldehyde following general procedure (62 mg, yield 51%). Product was isolated as oil after column chromatography on silica gel (15 g, hex/AcOEt 90 : 10 → 80 : 20). ^1^H NMR (400 MHz, CDCl_3_) *δ* 9.97 (s, 1H), 7.83 (d, *J* = 7.9 Hz, 2H), 7.48 (d, *J* = 7.9 Hz, 2H), 6.75 (t, *J* = 2.8 Hz, 1H), 3.77 (d, *J* = 1.1 Hz, 6H), 2.72 (td, *J* = 7.3, 2.6 Hz, 2H), 2.42–2.37 (m, 2H), 1.85 (p, *J* = 7.1 Hz, 2H); ^13^C NMR (101 MHz, CDCl_3_) *δ* 191.7, 171.0, 144.8, 143.7, 134.6, 129.6, 129.1, 126.6, 65.6, 52.9, 35.6, 32.3, 24.8; IR (CH_2_Cl_2_): 2954, 2840, 1731, 1696, 1602, 1565, 1434, 1264, 1168, 822, 792, 523 cm^−1^; MS (EI): *m*/*z* (%) = 303(17), 302(63)[M^+^], 270(41), 243(42), 242(50), 213(21), 211(99), 210(54), 183(73), 156(27), 155(100), 153(49), 128(33), 115(31), 91(37), 77(27), 59(33); HRMS (EI): *m*/*z* calcd for C_17_H_18_O_5_ 302.1154; found 302.1159.

### Dimethyl (2*E*)-2-(3-((*tert*-butoxycarbonyl)amino)benzylidene)cyclopentane-1,1-dicarboxylate (22)

Prepared in reaction of dimethyl 4-pentenylmalonate and *N*-(*tert*-butoxycarbonyl)-3-bromoaniline following general procedure (148 mg, yield 95%). Product was isolated as oil after column chromatography on silica gel (15 g, hex/AcOEt 85 : 15 → 80 : 20). ^1^H NMR (400 MHz, CDCl_3_) *δ* 7.39 (s, 1H), 7.24–7.16 (m, 2H), 7.00 (dt, *J* = 7.2, 1.7 Hz, 1H), 6.65 (t, *J* = 2.7 Hz, 1H), 6.60 (s, 1H), 3.75 (s, 6H), 2.70 (td, *J* = 7.2, 2.6 Hz, 2H), 2.37 (t, *J* = 6.9 Hz, 2H), 1.81 (p, *J* = 7.1 Hz, 2H), 1.50 (s, 9H); ^13^C NMR (101 MHz, CDCl_3_) *δ* 171.3, 152.7, 141.4, 138.4, 138.3, 128.6, 127.2, 123.4, 118.7, 117.1, 80.3, 65.4, 52.7, 35.7, 32.0, 28.3, 24.8; IR (CH_2_Cl_2_): 3361, 2976, 2955, 1729, 1538, 1435, 1237, 1160, 1065, 888, 737, 693, 463 cm^−1^; HRMS (EI): *m*/*z* calcd for C_21_H_27_NO_6_ 389.1736; found 389.1736.

### Dimethyl (2*E*)-2-(thiophen-2-ylmethylidene)cyclopentane-1,1-dicarboxylate (23)

Prepared in reaction of dimethyl 4-pentenylmalonate and 2-bromothiophene following general procedure (53 mg, yield 47%). Product was isolated as oil after column chromatography on silica gel (15 g, hex/AcOEt 98 : 2 → 90 : 10). ^1^H NMR (400 MHz, CDCl_3_) *δ* 7.31–7.27 (m, 1H), 7.04–7.01 (m, 2H), 6.93 (t, *J* = 2.7 Hz, 1H), 3.76 (s, 6H), 2.69 (td, *J* = 7.3, 2.6 Hz, 2H), 2.40 (t, *J* = 6.9 Hz, 2H), 1.90 (p, *J* = 7.1 Hz, 2H); ^13^C NMR (101 MHz, CDCl_3_) *δ* 171.1, 141.3, 138.7, 127.4, 126.9, 125.6, 120.8, 65.3, 52.8, 36.1, 32.2, 24.5; IR (CH_2_Cl_2_): 2952, 1731, 1433, 1261, 1148, 701 cm^−1^; MS (EI): *m*/*z* (%) = 280(53)[M^+^], 222(36), 221(100), 220(84), 190(21), 189(62), 167(30), 161(87), 128(32), 115(20), 97(52), 77(23), 59(32); HRMS (EI): *m*/*z* calcd for C_14_H_16_O_4_S 280.0769; found 280.0761.

### Dimethyl (2*E*)-2-(2-chlorobenzylidene)cyclopentane-1,1-dicarboxylate (24)

Prepared in reaction of dimethyl 4-pentenylmalonate and 1-chloro-2-bromobenzene following general procedure (114 mg, yield 93%). Product was isolated as oil after column chromatography on silica gel (15 g, hex/AcOEt 90 : 10). ^1^H NMR (400 MHz, CDCl_3_) *δ* 7.40–7.34 (m, 2H), 7.24–7.19 (m, 1H), 7.18–7.13 (m, 1H), 6.95 (t, *J* = 2.6 Hz, 1H), 3.79 (s, 6H), 2.58 (td, *J* = 7.2, 2.6 Hz, 2H), 2.39 (t, *J* = 6.9 Hz, 2H), 1.81 (p, *J* = 7.0 Hz, 2H); ^13^C NMR (101 MHz, CDCl_3_) *δ* 171.1, 142.9, 135.7, 133.7, 129.6, 129.3, 128.1, 126.2, 124.5, 64.7, 52.8, 35.7, 31.6, 24.7; IR (CH_2_Cl_2_): 2953, 1733, 1590, 1436, 1258, 1138, 751, 606 cm^−1^; MS (EI): *m*/*z* (%) = 310(22), 308(48)[M^+^], 276(27), 249(42), 248(48), 219(42), 217(83), 213(94), 191(48), 189(100), 153(65), 125(62), 115(26), 77(27); HRMS (EI) *m*/*z* calcd for C_16_H_17_O_4_Cl 308.0815; found 308.0819.

### Dimethyl (2*E*)-2-(4-nitrobenzylidene)cyclopentane-1,1-dicarboxylate (25)

Prepared in reaction of dimethyl 4-pentenylmalonate and 4-bromonitrobenzene following modified general procedure (run at 80 °C) (61 mg, yield 48%, isomer *E*/*Z* 20 : 80) or with 4-chloronitrobenzene following modified general procedure (run at 80 °C) (50 mg, yield 40%, isomer *E*/*Z* 45 : 55). Product was isolated as oil after column chromatography on silica gel (15 g, hex/AcOEt 75 : 25). ^1^H NMR (400 MHz, CDCl_3_) *δ* 8.14–8.08 (m, 4H), 5.41–5.38 (m, 1H), 3.67 (s, 6H), 3.64 (q, *J* = 2.3 Hz, 2H), 2.57–2.51 (m, 2H), 2.40–2.33 (m, 2H); ^13^C NMR (101 MHz, CDCl_3_) *δ* 171.2, 170.5, 147.5, 146.5, 146.4, 145.3, 143.5, 139.9, 134.1, 123.0, 129.2, 124.9, 123.4, 122.9, 67.6, 64.0, 52.6, 52.5, 39.4, 35.0, 35.0, 33.9, 30.4, 22.6. Indicative signals of minor isomer: ^1^H NMR (400 MHz, CDCl_3_) *δ* 7.49–7.45 (m, 2H), 7.37–7.32 (m, 2H), 6.67 (t, *J* = 2.4 Hz, 1H), 3.47 (s, 6H), 2.68 (td, *J* = 7.6, 2.2 Hz, 2H), 2.44 (t, *J* = 7.0 Hz, 2H), 1.76 (p, *J* = 7.3 Hz, 2H); MS (EI): *m*/*z* (%) = 319(36)[M^+^], 287(33), 260(48), 259(100), 229(28), 228(99), 227(48), 200(73), 154(66), 128(31), 115(30), 106(14), 90(19), 77(25), 59(49), 39(16); IR (CH_2_Cl_2_): 2953, 2854, 1732, 1597, 1519, 1434, 1346, 1266, 1156, 1066, 857 cm^−1^; HRMS (EI): *m*/*z* calcd for C_16_H_17_NO_6_ 319.1056; found 319.1057.

### Di(propan-2-yl) (2*E*)-2-benzylidenecyclopentane-1,1-dicarboxylate (26)

Prepared in reaction of dipropan-2-yl 2-pent-4-ynylpropanedioate and bromobenzene following modified general procedure (run at 80 °C) (56 mg, yield 42%). Product was isolated as oil after column chromatography on silica gel (15 g, hex/AcOEt 95 : 5). ^1^H NMR (400 MHz, CDCl_3_) *δ* 7.34–7.32 (m, 4H), 7.23–7.18 (m, 1H), 6.76 (t, *J* = 2.6 Hz, 1H), 5.10 (hept, *J* = 6.3 Hz, 2H), 2.70 (td, *J* = 7.2, 2.6 Hz, 2H), 2.36 (t, *J* = 6.9 Hz, 2H), 1.82 (p, *J* = 7.1 Hz, 2H), 1.28 (dd, *J* = 6.3, 4.1 Hz, 12H); ^13^C NMR (101 MHz, CDCl_3_) *δ* 170.3, 141.2, 137.9, 128.6, 128.1, 127.2, 126.6, 68.8, 65.2, 35.6, 32.1, 24.7, 21.6, 21.5; IR (CH_2_Cl_2_): 3450, 2980, 2875, 1722, 1449, 1374, 1251, 1104, 909, 777, 699, 517 cm^−1^. MS (EI): *m*/*z* (%) = 330(27)[M^+^], 244(35), 202(59), 201(72), 184(54), 183(57), 173(44), 155(100), 129(43), 115(29), 91(56), 77(25), 43(95); HRMS (EI): *m*/*z* calcd for C_20_H_26_O_4_ 330.1831; found 330.1822.

### Di(propan-2-yl) (2*E*)-2-(4-methoxybenzylidene)cyclopentane-1,1-dicarboxylate (27)

Prepared in reaction of dipropan-2-yl 2-pent-4-ynylpropanedioate and 4-bromoanisole following modified general procedure (run at 80 °C) (68 mg, yield 47%). Product was isolated as oil after column chromatography on silica gel (15 g, hex/AcOEt 90 : 10). ^1^H NMR (400 MHz, CDCl_3_) *δ* 7.29–7.24 (m, 2H), 6.88–6.84 (m, 2H), 6.68 (t, *J* = 2.6 Hz, 1H), 5.08 (hept, *J* = 6.2 Hz, 2H), 3.80 (s, 3H), 2.67 (td, *J* = 7.2, 2.6 Hz, 2H), 2.33 (t, *J* = 6.9 Hz, 2H), 1.81 (p, *J* = 7.1 Hz, 2H), 1.26 (dd, *J* = 6.3, 4.0 Hz, 12H); ^13^C NMR (101 MHz, CDCl_3_) *δ* 170.4, 158.3, 139.0, 130.7, 129.9, 126.7, 113.6, 68.7, 65.1, 55.2, 35.6, 32.0, 24.7, 21.6, 21.5; IR (CH_2_Cl_2_): 2979, 2936, 1724, 1607, 1511, 1466, 1250, 1103, 827, 530 cm^−1^; MS (EI): *m*/*z* (%) = 361(14), 360(43) [M^+^], 317(11), 273(36), 232(32), 231(100), 214(33), 213(30), 185(53), 171(12), 159(13), 135(17), 121(28), 115(21), 43(50), 41(24); HRMS (EI): *m*/*z* calcd for C_21_H_28_O_5_ 360.1937; found 360.1936.

### Di(propan-2-yl) (2*E*)-2-(4-cyanobenzylidene)cyclopentane-1,1-dicarboxylate (28)

Prepared in reaction of dipropan-2-yl 2-pent-4-ynylpropanedioate and 4-bromobenzonitrile following modified general procedure (run at 80 °C) (37 mg, yield 26%, isomer *E*/*Z* 75 : 25). Product was isolated as oil after column chromatography on silica gel (25 g, hex/AcOEt 95 : 5). ^1^H NMR (400 MHz, CDCl_3_) *δ* 7.55–7.49 (m, 4H), 6.62 (t, *J* = 2.2 Hz, 1H), 4.81 (hept, *J* = 6.3 Hz, 2H), 2.68 (td, *J* = 7.6, 2.2 Hz, 2H), 2.43 (t, *J* = 7.0 Hz, 2H), 1.76 (p, *J* = 7.2 Hz, 2H), 1.13 (d, *J* = 6.2 Hz, 6H), 1.04 (d, *J* = 6.3 Hz, 6H); ^13^C NMR (101 MHz, CDCl_3_) *δ* 169.6, 144.7, 132.1, 131.5, 130.2, 129.3, 125.2, 110.1, 69.5, 64.3, 39.8, 35.8, 22.7, 21.4, 21.3; IR (CH_2_Cl_2_): 2981, 2937, 2226, 1724, 1604, 1375, 1265, 1102, 1128, 845, 696, 515 cm^−1^; Indicative signals of minor isomer (*Z*): ^1^H NMR (400 MHz, CDCl3) *δ* 7.59–7.55 (m, 2H), 7.35–7.30 (m, 2H), 5.23 (p, *J* = 2.1 Hz, 1H), 5.05 (hept, *J* = 6.3 Hz, 2H), 3.61–3.55 (m, 2H), 2.55–2.48 (m, 2H), 2.37–2.30 (m, 2H), 1.25 (dd, *J* = 6.3, 5.1 Hz, 12H); ^13^C NMR (101 MHz, CDCl_3_) *δ* 170.5, 145.8, 141.7, 140.6, 133.3, 119.1, 110.1, 69.0, 68.0, 35.3, 33.8, 30.3, 21.6, 21.6; MS (EI): *m*/*z* (%) = 355(11)[M^+^], 313(7), 269(20), 227(54), 209(30), 180(40), 154(19), 116(29), 77(10), 57(11); HRMS (EI): *m*/*z* calcd for C_21_H_25_NO_4_ 355.1784; found 355.1781.

### Di-*tert*-butyl (2*E*)-2-benzylidenecyclopentane-1,1-dicarboxyl-ate (29)

Prepared in reaction of di-*tert*-butyl pent-4-yn-1-ylpropanedioate and bromobenzene following modified general procedure (run at 80 °C) (37 mg, yield 26%). Product was isolated as oil after column chromatography on silica gel (25 g, hex/AcOEt 98 : 2). ^1^H NMR (400 MHz, CDCl_3_) *δ* 7.36–7.29 (m, 4H), 7.23–7.17 (m, 1H), 6.77 (t, *J* = 2.6 Hz, 1H), 2.68 (td, *J* = 7.2, 2.6 Hz, 2H), 2.30 (t, *J* = 6.9 Hz, 2H), 1.79 (p, *J* = 7.0 Hz, 2H), 1.50 (s, 18H); ^13^C NMR (101 MHz, CDCl_3_) *δ* 169.9, 141.5, 138.1, 128.6, 128.1, 127.0, 126.5, 81.2, 66.51, 35.7, 32.1, 27.9, 24.6; MS (EI): *m*/*z* (%) = 247(3), 246(12), 202(34), 185(14), 184(21), 183(11), 155(21), 142(7), 129(14), 115(13), 106(9), 91(16), 79(16), 57(100), 41(34); IR (CH_2_Cl_2_): 3054, 2977, 2930, 1725, 1599, 1368, 1270, 1166, 1128, 845, 696, 515 cm^−1^; HRMS (EI): *m*/*z* calcd for C_22_H_30_O_4_ 358.2144; found 358.2115.

### Di-*tert*-butyl (2*E*)-2-(4-methoxybenzylidene)cyclopentane-1,1-dicarboxylate (30)

Prepared in reaction of di-*tert*-butyl pent-4-yn-1-ylpropanedioate and 4-bromoanisole following modified general procedure (run at 80 °C) (35 mg, yield 23%). Product was isolated as oil after column chromatography on silica gel (25 g, hex/AcOEt 90 : 10). ^1^H NMR (400 MHz, CDCl_3_) *δ* 7.29–7.25 (m, 2H), 6.88–6.84 (m, 2H), 6.69 (t, *J* = 2.5 Hz, 1H), 3.80 (s, 3H), 2.65 (td, *J* = 7.2, 2.6 Hz, 2H), 2.27 (t, *J* = 6.9 Hz, 2H), 1.78 (p, *J* = 7.1 Hz, 2H), 1.48 (s, 18H); ^13^C NMR (101 MHz, CDCl_3_) *δ* 170.1, 158.3, 139.3, 130.9, 129.9, 126.4, 113.6, 81.2, 66.5, 55.2, 35.7, 32.1, 27.9, 27.7, 24.6; IR (CH_2_Cl_2_) 3449, 2977, 2934, 1725, 1607, 1511, 1456, 1368, 1251, 1167, 1129, 1036, 848, 828 cm^−1^; MS (EI): *m*/*z* (%) = 388(7)[M^+^], 276(22), 232(40), 231(100), 214(25), 203(11), 185(36), 171(12), 121(21), 115(15), 91(4), 77(8), 57(96), 43(12), 41(33); HRMS (EI): *m*/*z* calcd for C_23_H_32_O_5_ 388.2250; found 388.2242.

### Di-*tert*-butyl (2*E*)-2-(4-cyanobenzylidene)cyclopentane-1,1-dicarboxylate (31)

Prepared in reaction of di-*tert*-butyl pent-4-yn-1-ylpropanedioate and 4-bromobenzonitrile following modified general procedure (run at 80 °C) (54 mg, yield 35%, isomer *E*/*Z* 35 : 65). Product was isolated as oil after column chromatography on silica gel (25 g, hex/AcOEt 95 : 5). ^1^H NMR (400 MHz, CDCl_3_) *δ* 7.55–7.50 (m, 4H), 6.57 (t, 1H), 2.65 (td, *J* = 7.5, 2.3 Hz, 3H), 2.37 (t, *J* = 6.9 Hz, 2H), 1.76–1.69 (m, 2H), 1.28 (s, 18H). ^13^C NMR (101 MHz, CDCl_3_) *δ* 169.2, 145.0, 141.8, 131.5, 129.5, 124.7, 109.9, 109.8, 81.9, 65.3, 40.5, 36.5, 27.6, 22.7; indicative signals of minor isomer: ^1^H NMR (400 MHz, CDCl_3_) *δ* 7.61–7.56 (m, 2H), 7.42–7.37 (m, 2H), 6.76 (t, *J* = 2.6 Hz, 1H), 2.29 (t, *J* = 6.9 Hz, 2H), 1.84–1.76 (m, 2H), 1.47 (s, 18H); ^13^C NMR (101 MHz, CDCl_3_) *δ* 169.36, 145.66, 142.49, 131.90, 129.00, 125.56, 119.06, 81.62, 66.83, 35.46, 32.37, 27.84, 24.48; IR (CH_2_Cl_2_): 3434, 2978, 2933, 2226, 1724, 1604, 1456, 1368, 1128, 1065, 844, 555 cm^−1^; MS (EI): *m*/*z* (%) = 384(1)[M^+^], 327(4), 283(10), 271(13), 254(9), 227(42), 210(15), 180(13), 153(11), 116(12), 77(3), 57(100), 43(6), 41(23).

### *tert*-Butyl (2*E*)-2-benzylidene-1-cyanocyclopentanecarboxylate (32)

Prepared in reaction of *tert*-butyl 2-cyanohept-6-ynoate and bromobenzene following general procedure (82 mg, yield 73%). Product was isolated as oil after column chromatography on silica gel (15 g, hex/AcOEt 90 : 10). ^1^H NMR (400 MHz, CDCl_3_) *δ* 7.39–7.31 (m, 4H), 7.29–7.24 (m, 1H), 6.85 (t, *J* = 2.6 Hz, 1H), 2.78–2.71 (m, 2H), 2.59–2.50 (m, 1H), 2.32–2.24 (m, 1H), 2.14–2.03 (m, 1H), 2.01–1.90 (m, 1H), 1.52 (s, 9H); ^13^C NMR (101 MHz, CDCl_3_) *δ* 166.7, 141.3, 136.4, 128.6, 128.5, 128.3, 127.5, 127.1, 120.1, 83.8, 53.6, 36.4, 30.8, 27.7, 25.1; MS (EI): *m*/*z* (%) = 283(1)[M^+^], 182(37), 153(10), 128(12), 115(15), 102(5), 91(17), 77(12), 57(100), 43(12), 41(26); IR (CH_2_Cl_2_): 3447, 2978, 2877, 2240, 2214, 1737, 1449, 1370, 1256, 1150, 840, 695, 513 cm^−1^; HRMS (EI): *m*/*z* calcd for C_18_H_21_NO_2_ 283.1572; found 283.1564.

### *tert*-Butyl (2*E*)-1-cyano-2-(4-methoxybenzylidene)cyclopentanecarboxylate (33)

Prepared in reaction of *tert*-butyl 2-cyanohept-6-ynoate and 4-bromoanisole following modified general procedure (run at 80 °C) (121 mg, yield 96%). Product was isolated as oil after column chromatography on silica gel (15 g, hex/AcOEt 80 : 20).^1^H NMR (400 MHz, CDCl_3_) *δ* 7.32–7.23 (m, 2H), 6.93–6.85 (m, 2H), 6.77 (t, *J* = 2.6 Hz, 1H), 3.80 (s, 3H), 2.78–2.64 (m, 2H), 2.58–2.46 (m, 1H), 2.31–2.20 (m, 1H), 2.13–2.01 (m, 1H), 2.00–1.88 (m, 1H), 1.51 (s, 9H); ^13^C NMR (101 MHz, CDCl_3_) *δ* 166.9, 158.9, 138.9, 129.9, 129.2, 126.6, 120.3, 113.8, 83.7, 55.17, 53.60, 36.5, 30.7, 27.7, 25.1; IR (CH_2_Cl_2_): 2978, 2935, 2838, 2240, 1735, 1607, 1512, 1462, 1370, 1253, 1152, 1034, 836, 513 cm^−1^; MS (EI): *m*/*z* (%) = 313(10)[M^+^], 213(65), 212(75), 198(28), 167(32), 121(16), 115(14), 91(8), 77(13), 57(100), 43(11), 41(27); HRMS (EI): *m*/*z* calcd for C_19_H_23_NO_3_ 313.1678; found 313.1680.

### *tert*-Butyl (2*E*)-1-cyano-2-(4-cyanobenzylidene)cyclopentane-carboxylate (34)

Prepared in reaction of *tert*-butyl 2-cyanohept-6-ynoate and 4-bromobenzonitrile following modified general procedure (run at 80 °C) (40 mg, yield 33%, isomer *E*/*Z* 23 : 77). Product was isolated as oil after column chromatography on silica gel (15 g, hex/AcOEt 90 : 10). ^1^H NMR (400 MHz, CDCl_3_) *δ* 7.61–7.56 (m, 2H), 7.35–7.29 (m, 2H), 5.49–5.45 (m, 1H), 3.58 (dq, *J* = 16.6, 2.1 Hz, 1H), 3.46 (dq, *J* = 16.5, 2.2 Hz, 1H), 2.66–2.57 (m, 1H), 2.54–2.44 (m, 3H), 1.45 (s, 9H); ^13^C NMR (101 MHz, CDCl_3_) *δ* 166.7, 143.3, 138.5, 134.0, 132.2, 132.1, 130.1, 129.0, 110.6, 84.1, 56.7, 36.1, 34.61, 31.0, 27.6; Indicative signals of minor isomer (*Z*): ^1^H NMR (400 MHz, CDCl_3_) *δ* 7.66–7.60 (m, 2H), 7.41 (d, *J* = 8.3 Hz, 2H), 6.82 (t, *J* = 2.7 Hz, 1H), 2.73 (td, *J* = 7.3, 2.6 Hz, 2H), 2.32–2.24 (m, 1H), 2.14–2.03 (m, 1H), 2.02–1.91 (m, 1H), 1.50 (s, 9H); ^13^C NMR (101 MHz, CDCl_3_) *δ* 166.2, 145.3, 140.7, 119.6, 118.5, 110.9, 84.3, 53.9, 36.4, 25.0; IR (CH_2_Cl_2_): 3059, 2879, 2935, 2228, 1736, 1606, 1370, 1254, 1152 cm^−1^; MS (EI): *m*/*z* (%) = 308(1)[M^+^], 252(10), 208(26), 207(30), 153(6), 116(28), 77(8), 57(100), 43(16), 41(28); HRMS (ESI): *m*/*z* calcd for C_19_H_20_N_2_O_2_Na 331.1525; found 331.1397.

### Propan-2-yl (2*E*)-2-benzylidene-1-cyanocyclopentane-carboxylate (35)

Prepared in reaction of propan-2-yl 2-cyanohept-6-ynoate and bromobenzene following modified general procedure (run for 4 h) (80 mg, yield 75%). Product was isolated as oil after column chromatography on silica gel (15 g, hex/AcOEt 95 : 5). ^1^H NMR (400 MHz, CDCl_3_) *δ* 7.39–7.30 (m, 4H), 7.29–7.24 (m, 1H), 6.84 (t, *J* = 2.6 Hz, 1H), 5.10 (hept, *J* = 6.3 Hz, 1H), 2.82–2.68 (m, 2H), 2.61–2.53 (m, 1H), 2.35–2.26 (m, 1H), 2.16–2.05 (m, 1H), 2.03–1.91 (m, 1H), 1.32 (t, *J* = 6.5 Hz, 6H); ^13^C NMR (101 MHz, CDCl_3_) *δ* 167.4, 141.0, 136.3, 128.6, 128.4, 127.6, 127.4, 119.9, 70.9, 52.9, 36.6, 30.8, 25.1, 21.4, 21.4; IR (CH_2_Cl_2_): 2981, 2241, 1737, 1450, 1376, 1237, 1103, 762, 695, 513 cm^−1^; MS (EI): *m*/*z* (%) = 270(3), 269(14), 184(5), 183(62), 182(82), 155(5), 129(22), 115(20), 102(8), 91(24), 77(18), 52(13), 43(100); HRMS (EI) *m*/*z* calcd for C_17_H_19_NO_2_ 269.1416; found 269.1422.

### Propan-2-yl (2*E*)-1-cyano-2-(4-methoxybenzylidene)cyclopentanecarboxylate (36)

Prepared in reaction of propan-2-yl 2-cyanohept-6-ynoate and 4-bromoanisole following modified general procedure (run for 4 h) (86 mg, yield 72%). Product was isolated as oil after column chromatography on silica gel (15 g, hex/AcOEt 90 : 10). ^1^H NMR (400 MHz, CDCl_3_) *δ* 7.31–7.23 (m, 2H), 6.92–6.84 (m, 2H), 6.76 (t, *J* = 2.6 Hz, 1H), 5.08 (hept, *J* = 6.3 Hz, 1H), 3.80 (s, 3H), 2.80–2.64 (m, 2H), 2.59–2.49 (m, 1H), 2.33–2.22 (m, 1H), 2.15–2.04 (m, 1H), 2.01–1.89 (m, 1H), 1.31 (t, *J* = 6.3 Hz, 6H); ^13^C NMR (101 MHz, CDCl_3_) *δ* 167.5, 159.0, 138.6, 129.9, 129.0, 126.8, 120.0, 113.8, 70.7, 55.2, 52.9, 36.5, 30.7, 25.1, 21.4, 21.3. IR (CH_2_Cl_2_): 2981, 2937, 2240, 1736, 1606, 1512, 1465, 1253, 1178, 1103, 1034, 831, 531 cm^−1^; MS (EI): *m*/*z* (%) = 299(19)[M^+^], 256(4), 212(100), 198(10), 170(12), 121(13), 115(12), 91(6), 77(10), 43(52); HRMS (EI): *m*/*z* calcd for C_18_H_21_NO_3_ 299.1521; found 299.1527.

### Propan-2-yl (2*E*)-1-cyano-2-(4-cyanobenzylidene)cyclopentanecarboxylate (37)

Prepared in reaction of propan-2-yl 2-cyanohept-6-ynoate and 4-bromobenzonitrile following modified general procedure (run for 4 h) (83 mg, yield 70%, isomer *E*/*Z* 20 : 80). Product was isolated as oil after column chromatography on silica gel (15 g, hex/AcOEt 90 : 10). ^1^H NMR (400 MHz, CDCl_3_) *δ* 7.60–7.55 (m, 2H), 7.32–7.27 (m, 2H), 5.50 (p, *J* = 1.9 Hz, 1H), 4.95 (hept, *J* = 6.3 Hz, 1H), 3.61–3.52 (m, 1H), 3.48–3.40 (m, 1H), 2.67–2.58 (m, 1H), 2.56–2.46 (m, 2H), 1.26–1.21 (m, 6H); ^13^C NMR (101 MHz, CDCl_3_) *δ* 167.3, 143.1, 138.2, 134.3, 132.7, 132.4, 132.2, 132.1, 130.0, 129.4, 129.0, 127.8, 118.6, 118.5, 110.6, 71.0, 56.0, 36.1, 34.5, 31.0, 21.4, 21.3, 21.3, 21.3; indicative signals of minor isomer (*Z*): ^1^H NMR (400 MHz, CDCl_3_) *δ* 7.78–7.72 (m, 1H), 7.71–7.65 (m, 1H), 7.65–7.57 (m, 1H), 7.40 (d, *J* = 8.4 Hz, 1H), 6.81 (t, *J* = 2.7 Hz, 1H), 5.08 (p, *J* = 6.2 Hz, 1H), 2.77–2.70 (m, 1H), 2.36–2.26 (m, 2H), 2.16–2.05 (m, 1H), 2.03–1.95 (m, 1H), 1.95–1.88 (m, 1H), 1.32–1.27 (m, 6H); ^13^C NMR (101 MHz, CDCl_3_) *δ* 166.8, 160.8, 145.0, 143.3, 142.5, 140.6, 125.8, 119.3, 118.2, 112.2, 110.9, 110.81, 110.2, 53.2, 37.1, 36.4, 35.5, 34.1, 25.0, 22.3; IR (CH_2_Cl_2_): 3452, 3060, 2983, 2938, 2228, 1738, 1606, 1326, 1248, 1178, 1104, 834, 553 cm^−1^; MS (EI): *m*/*z* (%) = 294(7)[M^+^], 252(10), 208(63), 204(34), 180(18), 153(15), 140(19), 116(45), 104(16), 89(20), 77(14), 43(100); HRMS (EI): *m*/*z* calcd for C_18_H_18_N_2_O_2_ 294.1368; found 294.1363.

### (2*E*)-2-Benzylidenecyclopentane-1,1-dicarbonitrile (38)

Prepared in reaction of pent-4-yn-1-ylpropanedinitrile and bromobenzene following modified general procedure (run for 4 h) (72 mg, yield 87%). Product was isolated as oil after column chromatography on silica gel (15 g, hex/AcOEt 90 : 10). ^1^H NMR (400 MHz, CDCl_3_) *δ* 7.44–7.31 (m, 5H), 6.99 (t, *J* = 2.7 Hz, 1H), 2.83 (td, *J* = 7.3, 2.7 Hz, 2H), 2.50 (t, *J* = 6.9 Hz, 2H), 2.15 (p, *J* = 7.1 Hz, 2H); ^13^C NMR (101 MHz, CDCl_3_) *δ* 136.5, 135.0, 129.9, 128.8, 128.7, 128.6, 128.4, 115.4, 40.3, 38.8, 29.3, 24.3; IR (CH_2_Cl_2_): 3058, 3029, 2953, 2246, 1492, 1449, 1194, 921, 760, 694, 512 cm^−1^; MS (EI): *m*/*z* (%) = 208(100)[M^+^], 207(55), 180(47), 153(30), 115(69), 102(21), 91(33), 77(26), 51(28), 39(26); HRMS (EI): *m*/*z* calcd for C_14_H_12_N_2_ 208.1000; found 208.1006.

### (2*E*)-2-(4-Methoxybenzylidene)cyclopentane-1,1-dicarbo-nitrile (39)

Prepared in reaction of pent-4-yn-1-ylpropanedinitrile and 4-bromoanisole following modified general procedure (run for 4 h) (86 mg, yield 91%). Product was isolated as oil after column chromatography on silica gel (15 g, hex/AcOEt 80 : 20). ^1^H NMR (400 MHz, CDCl_3_) *δ* 7.34–7.28 (m, 2H), 6.95–6.89 (m, 3H), 3.83 (s, 3H), 2.79 (td, *J* = 7.3, 2.7 Hz, 2H), 2.47 (t, *J* = 6.9 Hz, 2H), 2.13 (p, *J* = 7.1 Hz, 2H); ^13^C NMR (101 MHz, CDCl_3_) *δ* 159.6, 133.8, 130.2, 129.3, 127.7, 115.6, 114.0, 55.2, 40.3, 38.8, 29.2, 24.4; IR (CH_2_Cl_2_): 2956, 2839, 2246, 1606, 1513, 1463, 1254, 1179, 1032, 890, 829, 531 cm^−1^; MS (EI): *m*/*z* (%) = 239(29), 238(100)[M^+^], 237(27), 223(16), 210(29), 195(21), 170(19), 160(40), 145(40), 129(25), 115(27), 91(17), 77(20), 51(17), 43(13), 39(18); HRMS (EI): *m*/*z* calcd for C_15_H_14_N_2_O 238.1106; found 238.1111.

### (*E*)-Methyl 1-acetyl-2-benzylidenecyclopentanecarboxylate (40)

Prepared in reaction of methyl 2-acetylhept-6-ynoate and bromobenzene following modified general procedure (run for 2 h) (yield: 80%) product was isolated as oil after column chromatography on silica gel (15 g, 95 : 5 → 90 : 10 hexanes/EtOAc). ^1^H NMR (400 MHz, CDCl_3_) *δ* 7.37–7.32 (m, 4H), 7.27–7.20 (m, 1H), 6.60 (t, *J* = 2.5 Hz, 1H), 3.78 (s, 3H), 2.80–2.63 (m, 2H), 2.50–2.41 (m, 1H), 2.26 (s, 3H), 2.25–2.16 (m, 1H), 1.89–1.75 (m, 2H); ^13^C NMR (101 MHz, CDCl_3_) *δ* 204.0, 171.8, 141.5, 137.4, 128.6, 128.2, 127.6, 126.9, 72.2, 52.6, 34.4, 32.0, 26.8, 24.8; IR (CH_2_Cl_2_): 3410, 2953, 1737, 1714, 1493, 1447, 1433, 1356, 1238, 697 cm^−1^; MS (EI), *m*/*z* (%): 258 (7, M^+^), 216 (80), 184 (100), 167 (13), 155 (86), 141 (19), 128 (34), 115 (29), 105 (14), 91 (35), 77 (23), 43 (46); HRMS (EI): *m*/*z* calcd for C_16_H_18_O_3_: 258.1256. Found 258.1255.

### (*E*)-Methyl 1-acetyl-2-(4-methoxybenzylidene)cyclopentane-carboxylate (41)

Prepared in reaction of methyl 2-acetylhept-6-ynoate and 4-bromoanisole following modified general procedure (run for 2 h) (yield: 79%). Product was isolated as oil after column chromatography on silica gel (15 g column, 90 : 10 → 80 : 20 hexanes/EtOAc). ^1^H NMR (400 MHz, CDCl_3_) *δ* 7.33–7.26 (m, *J* = 8.7 Hz, 2H), 6.91–6.85 (m, 2H), 6.53 (t, *J* = 2.4 Hz, 1H), 3.81 (s, 3H), 3.77 (s, 3H), 2.79–2.59 (m, 2H), 2.44 (dt, *J* = 13.5, 6.9 Hz, 1H), 2.25 (s, 3H), 2.24–2.13 (m, 1H), 1.90–1.76 (m, 2H); ^13^C NMR (101 MHz, CDCl_3_) *δ* 204.3, 172.0, 158.5, 139.2, 130.2, 129.9, 127.1, 113.7, 72.2, 55.2, 52.6, 34.4, 31.9, 26.7, 24.8; IR (CH_2_Cl_2_): 2954, 1737, 1712, 1606, 1512, 1461, 1435, 1355, 1251, 1177, 1034, 826, 531 cm^−1^; MS (EI), *m*/*z* (%): 288 (24, M^+^), 245 (79), 229 (16), 214 (50), 185 (100), 171 (16), 159 (14), 141 (13), 128 (14), 121 (24), 115 (23), 77 (10), 43 (32); HRMS (EI): *m*/*z* calcd for C_17_H_20_O_4_: 288.1362. Found 288.1364.

### (*E*)-Methyl 1-acetyl-2-(4-cyanobenzylidene)cyclopentane-carboxylate (42)

Prepared in reaction of methyl 2-acetylhept-6-ynoate and 4-bromobenzonitrile following modified general procedure (run for 2 h) (yield: 75%, *E*/*Z* 91 : 9) Product was isolated as oil after column chromatography on silica gel (15 g column, 9 : 1 → 8 : 2 hexanes/EtOAc). ^1^H NMR (400 MHz, CDCl_3_) *δ* 7.63–7.56 (m, 2H), 7.40 (d, *J* = 8.3 Hz, 2H), 6.57 (t, *J* = 2.5 Hz, 1H), 3.78 (s, 3H), 2.76–2.59 (m, 2H), 2.53–2.43 (m, 1H), 2.24 (s, 3H), 2.22–2.14 (m, 1H), 1.91–1.75 (m, 2H); ^13^C NMR (101 MHz, CDCl_3_) *δ* 202.9, 171.3, 145.4, 141.9, 132.0, 129.1, 126.2, 118.9, 110.2, 72.4, 52.9, 34.3, 32.3, 26.8, 24.7; indicative signals of *Z* isomer: ^1^H NMR (400 MHz, CDCl_3_) *δ* 7.52–7.48 (m, 2H), 7.28 (d, *J* = 8.1 Hz, 2H), 6.68 (s, 1H), 3.40 (s, 3H), 2.06 (s, 3H); ^13^C NMR (101 MHz, CDCl_3_) *δ* 204.3, 145.2, 141.3, 131.6, 125.4, 110.5, 70.3, 52.3, 38.4, 35.8, 23.1; IR (CH_2_Cl_2_): 2954, 2880, 2842, 2226, 1738, 1713, 1604, 1503, 1433, 1357, 1239, 1177, 1153, 1129, 886, 827, 555; HRMS (ESI): *m*/*z* calcd for C_17_H_17_NO_3_Na ([M + Na]^+^): 306.1106. Found 306.1107.

### 1-((2*E*)-1-Benzoyl-2-benzylidenecyclopentyl)ethanone (43)

Prepared in reaction of 2-(pent-4-ynyl)-1-phenylbutane-1,3-dione and bromobenzene following modified general procedure (run at 80 °C) (55 mg, yield 45%). Product was isolated as oil after column chromatography on silica gel (15 g, hex/AcOEt 90 : 10). ^1^H NMR (400 MHz, CDCl_3_) *δ* 7.83–7.78 (m, 2H), 7.54–7.49 (m, 1H), 7.44–7.39 (m, 2H), 7.36–7.31 (m, 4H), 7.26–7.21 (m, 1H), 2.90–2.80 (m, 2H), 2.79–2.69 (m, 1H), 2.34 (s, 3H), 2.32–2.24 (m, 1H), 1.92–1.84 (m, 2H); ^13^C NMR (101 MHz, CDCl_3_) *δ* 204.8, 199.2, 142.2, 137.4, 135.5, 132.5, 129.3, 128.8, 128.6, 128.3, 128.2, 127.0, 77.2, 34.9, 31.8, 27.4, 24.8; IR (CH_2_Cl_2_): 3056, 3025, 2959, 2876, 1683, 1597, 1446, 1258, 1231, 735, 696, 516 cm^−1^; MS (EI): *m*/*z* (%) = 304(5)[M^+^], 262(13), 233(12), 199(11), 182(13), 155(10), 128(15), 105(100), 91(19), 77(48), 51(15), 43(32); HRMS (EI) *m*/*z* calcd for C_21_H_20_O_2_ 304.1463; found 304.1462.

### 1-((2*E*)-1-Benzoyl-2-(4-methoxybenzylidene)cyclopentyl)ethanone (44)

Prepared in reaction of 2-(pent-4-ynyl)-1-phenylbutane-1,3-dione and 4-bromoanisole following modified general procedure (run at 80 °C) (78 mg, yield 60%). Product was isolated as oil after column chromatography on silica gel (15 g, hex/AcOEt 80 : 20). ^1^H NMR (400 MHz, CDCl_3_) *δ* 7.81–7.76 (m, 2H), 7.52–7.47 (m, 1H), 7.42–7.36 (m, 2H), 7.29–7.24 (m, 2H), 6.90–6.85 (m, 2H), 6.40 (t, *J* = 2.5 Hz, 1H), 3.80 (s, 3H), 2.87–2.74 (m, 2H), 2.73–2.66 (m, 1H), 2.32 (s, 3H), 2.29–2.22 (m, 1H), 1.92–1.82 (m, 2H); ^13^C NMR (101 MHz, CDCl_3_) *δ* 204.9, 199.4, 158.6, 139.9, 135.6, 132.4, 130.2, 129.9, 129.2, 128.3, 128.2, 113.7, 77.2, 55.2, 34.9, 31.7, 27.3, 24.9; IR (CH_2_Cl_2_): 3059, 2968, 2837, 1684, 1605, 1511, 1446, 1251, 1177, 1032, 880, 829, 701 cm^−1^; MS (EI): *m*/*z* (%) = 334(25)[M^+^], 292(50), 291(34), 229(100), 187(33), 135(26), 121(31), 105(89), 77(57), 43(51); HRMS (EI) *m*/*z* calcd for C_22_H_22_O_3_ 334.1569; found 334.1574.

### 4-(((1*E*)-2-Acetyl-2-benzoylcyclopentylidene)methyl)benzo-nitrile (45)

Prepared in reaction of 2-(pent-4-ynyl)-1-phenylbutane-1,3-dione and 4-brombenzonitrile following modified general procedure (run at 80 °C) (51 mg, yield 40%, isomer *E*/*Z* 70 : 30). Product was isolated as oil after column chromatography on silica gel (25 g, hex/AcOEt 90 : 10). ^1^H NMR (400 MHz, CDCl_3_) *δ* 7.77–7.73 (m, 2H), 7.65–7.58 (m, 2H), 7.46–7.38 (m, 5H), 6.45 (t, *J* = 2.6 Hz, 1H), 2.88–2.69 (m, 4H), 2.31 (s, 3H), 1.95–1.82 (m, 2H); ^13^C NMR (101 MHz, CDCl_3_) *δ* 204.3, 198.2, 145.9, 141.9, 135.3, 132.8, 132.1, 132.0, 130.3, 129.2, 129.1, 128.9, 128.6, 128.5, 127.1, 35.0, 32.1, 27.4, 24.8; indicative signals of minor isomer (*Z*): ^1^H NMR (400 MHz, CDCl_3_) *δ* 7.57 (d, *J* = 1.9 Hz, 1H), 7.56–7.50 (m, 5H), 7.36–7.30 (m, 3H), 5.41 (p, *J* = 2.0 Hz, 1H), 2.58–2.35 (m, 6H), 2.24 (s, 3H); ^13^C NMR (101 MHz, CDCl_3_) *δ* 206.5, 199.4, 145.3, 135.2, 133.6, 133.0, 118.8, 110.3, 79.3, 35.4, 33.3, 31.1, 27.3; IR (CH_2_Cl_2_): 3058, 2962, 2226, 1695, 1692, 1603, 1446, 1357, 1232, 700, 553 cm^−1^; MS (EI): *m*/*z* (%) = 329(1)[M^+^], 287(39), 286(16), 258(8), 153(9), 127(6), 116(12), 105(100), 77(48), 51(15), 43(30); HRMS (EI): *m*/*z* calcd for C_22_H_19_NO_2_ 329.1416; found 329.1404.

### ((2*E*)-1-Benzoyl-2-benzylidenecyclopentyl)(phenyl)methanone (46)

Prepared in reaction of 2-(pent-4-ynyl)-1,3-diphenylpropane-1,3-dione and bromobenzene following modified general procedure (run at 80 °C) (71 mg, yield 49%). Product was isolated as oil after column chromatography on silica gel (25 g, hex/AcOEt 95 : 5). ^1^H NMR (400 MHz, CDCl_3_) *δ* 7.85–7.81 (m, 4H), 7.50–7.45 (m, 2H), 7.41–7.36 (m, 4H), 7.34–7.28 (m, 4H), 7.24–7.20 (m, 1H), 6.41 (t, *J* = 2.5 Hz, 1H), 2.92 (td, *J* = 7.4, 2.5 Hz, 2H), 2.68 (t, *J* = 7.2 Hz, 2H), 1.89 (p, *J* = 7.3 Hz, 2H); ^13^C NMR (101 MHz, CDCl_3_) *δ* 199.0, 143.1, 137.6, 136.2, 132.4, 129.3, 128.9, 128.8, 128.4, 128.1, 126.8, 75.5, 36.7, 32.0, 24.2; IR (CH_2_Cl_2_): 3059, 3026, 2959, 1689, 1659, 1597, 1447, 1264, 1125, 697 cm^−1^; MS (EI): *m*/*z* (%) = 366(2)[M^+^], 262(7), 261(20), 245(12), 244(21), 183(4), 155(6), 128(7), 115(7), 105(100), 91(13), 77(44), 51(11); HRMS (EI): *m*/*z* calcd for C_26_H_22_O_2_ 366.1620; found 366.1620.

### ((2*E*)-1-Benzoyl-2-(4-methoxybenzylidene)cyclopentyl)(phenyl)methanone (47)

Prepared in reaction of 2-(pent-4-ynyl)-1,3-diphenylpropane-1,3-dione and 4-bromoanisole following modified general procedure (run at 80 °C) (88 mg, yield 56%). Product was isolated as oil after column chromatography on silica gel (25 g, hex/AcOEt/dioxane 85 : 10 : 5). ^1^H NMR (400 MHz, CDCl_3_) *δ* 7.84–7.80 (m, 4H), 7.49–7.43 (m, 2H), 7.40–7.34 (m, 4H), 7.27–7.22 (m, 2H), 6.89–6.84 (m, 2H), 6.34 (t, *J* = 2.5 Hz, 1H), 3.78 (s, 3H), 2.92–2.87 (m, 2H), 2.65 (t, *J* = 7.2 Hz, 2H), 1.88 (p, *J* = 7.3 Hz, 2H); ^13^C NMR (101 MHz, CDCl_3_) *δ* 199.2, 158.4, 140.8, 136.2, 132.3, 130.3, 130.0, 129.3, 128.3, 128.3, 113.5, 75.5, 55.1, 36.7, 31.9, 24.3; IR (CH_2_Cl_2_): 3058, 2956, 2836, 1687, 1659, 1606, 1510, 1251, 1177, 1033, 828, 701, 531 cm^−1^; MS (EI): *m*/*z* (%) = 397(5)[M^+^], 369(15), 292(33), 291(100), 274(25), 263(19), 155(11), 135(19), 105(75), 91(13), 77(54), 51(15); HRMS (EI): *m*/*z* calcd for C_27_H_24_O_3_ 396.1725; found 396.1719.

### 4-(((1*E*)-2,2-Dibenzoylcyclopentylidene)methyl)benzonitrile (48)

Prepared in reaction of 2-(pent-4-ynyl)-1,3-diphenylpropane-1,3-dione and 4-bromobenzonitrile following modified general procedure (run at 80 °C) (63 mg, yield 40%). Product was isolated as oil after column chromatography on silica gel (25 g, hex/AcOEt 90 : 10). ^1^H NMR (400 MHz, CDCl_3_) *δ* 7.81–7.76 (m, 4H), 7.59–7.56 (m, 2H), 7.50–7.45 (m, 2H), 7.40–7.34 (m, 6H), 6.38 (t, *J* = 2.6 Hz, 1H), 2.88 (td, *J* = 7.4, 2.6 Hz, 2H), 2.69 (t, *J* = 7.2 Hz, 2H), 1.90 (p, *J* = 7.3 Hz, 2H); ^13^C NMR (101 MHz, CDCl_3_) *δ* 198.5, 147.3, 142.0, 135.8, 132.6, 131.9, 129.3, 129.2, 128.5, 128.5, 127.2, 118.9, 110.1, 75.7, 36.6, 32.2, 24.2; IR (CH_2_Cl_2_): 3361, 3060, 2961, 2226, 1659, 1601, 1446, 1265, 1225, 1178, 879, 832, 736, 701, 554 cm^−1^; MS (EI): *m*/*z* (%) = 391(1)[M^+^], 287(2), 285(7), 269(4), 201(6), 153(3), 130(4), 105(100), 77(40), 51(10); HRMS (EI): *m*/*z* calcd for C_27_H_21_NO_2_ 391.1572; found 391.1586.

### Ethyl (2*E*)-2-benzylidene-1-(diethylphosphono)cyclopentanecarboxylate (49)

Prepared in reaction of ethyl 2-(diethylphosphono)hept-6-ynoate and bromobenzene following general procedure (20 mg, yield 14%). Product was isolated as oil after HPLC chromatography (DCM/MeOH 99,5 : 0,5 → 99 : 1). ^1^H NMR (400 MHz, CDCl_3_) *δ* 7.36–7.29 (m, 4H), 7.27–7.17 (m, 1H), 6.98 (7.01–6.94 (m, 1H), 4.28–4.10 (m, 6H), 2.79–2.68 (m, 1H), 2.69–2.59 (m, 1H), 2.58–2.45 (m, 1H), 2.45–2.33 (m, 1H), 2.01–1.87 (m, 1H), 1.84–1.73 (m, 1H), 1.34–1.25 (m, 9H); ^13^C NMR (101 MHz, CDCl_3_) *δ* 170.5, 140.3 (d, *J* = 7.7 Hz), 137.9 (d, *J* = 3.9 Hz), 128.7 (d, *J* = 1.8 Hz), 128.2, 127.5 (d, *J* = 7.5 Hz), 126.7, 63.3 (d, *J* = 6.9 Hz), 62.9 (d, *J* = 7.3 Hz), 61.6, 58.9 (d, *J* = 143.8 Hz), 33.7 (d, *J* = 3.4 Hz), 32.7 (d, *J* = 5.3 Hz), 29.7, 25.4 (d, *J* = 6.3 Hz), 16.5 (d, *J* = 5.7 Hz), 14.0; ^31^P NMR (162 MHz, CDCl_3_) *δ* 23.4; IR (CH_2_Cl_2_): 3233, 2979, 2928, 1728, 1446, 1248, 1025, 966, 759, 698, 572 cm^−1^; MS (EI): *m*/*z* (%) = 367(16), 366(41)[M^+^], 293(33), 229(42), 184(33), 183(82), 156(30), 155(100), 129(26), 115(29), 105(23), 91(38), 77(25), 43(8); HRMS (EI): *m*/*z* calcd for C_19_H_27_O_5_P 366.1596; found 366.1604.

### Dipropan-2-yl ((2*E*)-2-benzylidene-1-cyanocyclopentyl) phosphonate (50)

Prepared in reaction of dipropan-2-yl (1-cyanohex-5-yn-1-yl)phosphonate and bromobenzene following general procedure (88 mg, yield 63%). Product was isolated as oil after column chromatography on silica gel (15 g, hex/AcOEt 60 : 40). ^1^H NMR (400 MHz, CDCl_3_) *δ* 7.37–7.28 (m, 4H), 7.27–7.18 (m, 1H), 6.98–6.91 (m, 1H), 4.86–4.73 (m, 2H), 2.80–2.61 (m, 2H), 2.54–2.41 (m, 1H), 2.37–2.25 (m, 1H), 2.14–2.03 (m, 1H), 1.89–1.77 (m, 1H), 1.39–1.27 (m, 12H); ^13^C NMR (101 MHz, CDCl_3_) *δ* 138.1 (d, *J* = 8.1 Hz), 136.5 (d, *J* = 3.7 Hz), 128.5 (d, *J* = 2.0 Hz), 128.3 (d, *J* = 7.7 Hz), 128.2, 127.2, 119.8 (d, *J* = 6.4 Hz), 73.0 (d, *J* = 7.2 Hz), 73.0 (d, *J* = 7.2 Hz), 46.1 (d, *J* = 148.1 Hz), 34.6 (d, *J* = 4.6 Hz), 31.3 (d, *J* = 3.6 Hz), 24.8 (d, *J* = 4.1 Hz), 24.0 (d, *J* = 3.3 Hz), 24.0 (d, *J* = 3.3 Hz), 23.6 (d, *J* = 3.9 Hz), 23.5 (d, *J* = 3.8 Hz); ^31^P NMR (162 MHz, CDCl_3_) *δ* 16.7; IR (CH_2_Cl_2_): 3458, 3253, 2981, 2936, 2235, 1450, 1387, 1255, 1103, 989, 762, 696, 585 cm^−1^; MS (EI): *m*/*z* (%) = 348(3), 347(10)[M^+^], 305(9), 264(20), 263(68), 210(10), 183(50), 182(100), 181(12), 166(32), 155(22), 129(19), 115(22), 91(25), 77(15), 51(7); HRMS (EI): *m*/*z* calcd for C_19_H_26_NO_3_P 347.1650; found 347.1647.

### Diethyl ((2*E*)-1-acetyl-2-benzylidenecyclopentyl)phosphonate (51)

Prepared in reaction of diethyl (2-oxooct-7-yn-3-yl)phosphonate and bromobenzene following general procedure (60 mg, yield 45%). Product was isolated as oil after column chromatography on silica gel (15 g, hex/AcOEt/dioxane 45 : 45 : 10). ^1^H NMR (400 MHz, CDCl_3_) *δ* 7.36–7.31 (m, 4H), 7.26–7.18 (m, 1H), 7.01–6.96 (m, 1H), 4.22–4.05 (m, 4H), 2.74–2.64 (m, 2H), 2.51–2.38 (m, 1H), 2.37 (s, 3H), 2.35–2.23 (m, 1H), 1.96–1.83 (m, 1H), 1.77–1.65 (m, 1H), 1.33–1.29 (m, 3H), 1.26 (td, *J* = 7.1, 0.6 Hz, 3H); ^13^C NMR (101 MHz, CDCl_3_) *δ* 203.8, 140.5 (d, *J* = 6.6 Hz), 137.6 (d, *J* = 3.7 Hz), 128.6 (d, *J* = 1.8 Hz), 128.2, 128.2 (d, *J* = 7.4 Hz), 126.8, 66.7 (d, *J* = 139.8 Hz), 63.3 (d, *J* = 7.0 Hz), 62.5 (d, *J* = 7.3 Hz), 32.7 (d, *J* = 0.9 Hz), 32.6 (d, *J* = 4.2 Hz), 27.5, 25.0, 24.9 (d, *J* = 6.9 Hz), 16.4 (d, *J* = 5.8 Hz), 16.3 (d, *J* = 5.8 Hz); ^31^P NMR (162 MHz, CDCl_3_) *δ* 23.8, 23.8; IR (CH_2_Cl_2_): 3455, 3230, 2980, 1706, 1445, 1227, 1049, 1024, 956, 760, 698, 599 cm^−1^; MS (EI): *m*/*z* (%) = 336(4)[M^+^], 295(29), 294(100), 266(15), 237(13), 220(5), 156(31), 155(84), 128(22), 115(19), 105(11), 91(29), 77(16), 43(29); HRMS (EI): *m*/*z* calcd for C_18_H_25_O_4_P 336.1490; found 336.1502.

### (2*E*)-2-Benzylidene-1-(diphenylphosphoryl)cyclopentane-carbonitrile (52)

Prepared in reaction of 2-(diphenylphosphoryl)hept-6-ynenitrile and bromobenzene following general procedure (62 mg, yield 41%). Product was isolated as oil after column chromatography on silica gel (15 g, hex/AcOEt 50 : 50). ^1^H NMR (400 MHz, CDCl_3_) *δ* 8.28–8.18 (m, 2H), 8.05–7.96 (m, 2H), 7.67–7.52 (m, 4H), 7.51–7.41 (m, 2H), 7.35–7.26 (m, 2H), 7.26–7.18 (m, 1H), 7.15 (d, *J* = 7.2 Hz, 2H), 5.97–5.91 (m, 1H), 2.89–2.70 (m, 2H), 2.65–2.51 (m, 1H), 2.44–2.29 (m, 1H), 2.23–2.11 (m, 1H), 1.90–1.75 (m, 1H); ^13^C NMR (101 MHz, CDCl_3_) *δ* 138.6 (d, *J* = 6.6 Hz), 136.3 (d, *J* = 3.4 Hz), 132.8 (d, *J* = 8.4 Hz), 132.7 (d, *J* = 2.9 Hz), 132.6 (d, *J* = 2.6 Hz), 131.9 (d, *J* = 8.5 Hz), 129.9 (d, *J* = 97.2 Hz), 128.85 (d, *J* = 11.7 Hz), 128.78 (d, *J* = 6.6 Hz), 128.5 (d, *J* = 1.9 Hz), 128.4 (d, *J* = 101.2 Hz), 128.21 (d, *J* = 12.4 Hz), 128.15, 127.3, 121.6 (d, *J* = 2.8 Hz), 47.5 (d, *J* = 64.7 Hz), 34.4, 31.8, 25.3 (d, *J* = 2.2 Hz); ^31^P NMR (162 MHz, CDCl_3_) *δ* 30.4; IR (CH_2_Cl_2_): 3057, 2961, 2871, 2230, 1438, 1203, 1115, 725, 695, 607, 548, 526 cm^−1^; MS (EI): *m*/*z* (%) = 384(12), 383(28)[M^+^], 382(6), 258(4), 202(25), 201(100), 182(15), 154(9), 115(7), 91(8), 77(25), 51(13); HRMS (EI): *m*/*z* calcd for C_25_H_22_NOP 383.1439; found 383.1428.

### Ethyl (2*E*)-2-benzylidene-1-(diphenylphosphoryl)cyclopentanecarboxylate (53)

Prepared in reaction of ethyl 2-(diphenylphosphoryl)hept-6-ynoate and bromobenzene following general procedure (83 mg, yield 52%). Product was isolated as oil after column chromatography on silica gel (15 g, hex/AcOEt 60 : 40 → 50 : 50). ^1^H NMR (400 MHz, CDCl_3_) *δ* 7.97–7.89 (m, 2H), 7.88–7.80 (m, 2H), 7.54–7.47 (m, 2H), 7.46–7.38 (m, 4H), 7.34–7.28 (m, 2H), 7.24–7.18 (m, 3H), 6.50 (p, *J* = 7.0 Hz, 1H), 4.22–4.06 (m, 2H), 2.69–2.51 (m, 2H), 2.47–2.34 (m, 1H), 1.85–1.68 (m, 2H), 1.28–1.22 (m, 1H), 1.11 (t, *J* = 7.1 Hz, 3H); ^13^C NMR (101 MHz, CDCl_3_) *δ* 171.0 (d, *J* = 2.0 Hz), 140.5 (d, *J* = 6.5 Hz), 137.6 (d, *J* = 3.2 Hz), 133.1 (d, *J* = 8.7 Hz), 132.2 (d, *J* = 8.7 Hz), 131.9 (d, *J* = 97.4 Hz), 131.68 (d, *J* = 2.8 Hz), 131.65 (d, *J* = 2.8 Hz), 131.1 (d, *J* = 100.3 Hz), 128.63, 128.61, 128.56 (d, *J* = 7.0 Hz), 128.2 (d, *J* = 11.7 Hz), 128.1, 127.7 (d, *J* = 11.7 Hz), 61.6, 61.2 (d, *J* = 65.9 Hz), 33.4, 33.1 (d, *J* = 2.9 Hz), 25.7 (d, *J* = 5.1 Hz), 13.7; ^31^P NMR (162 MHz, CDCl_3_) *δ* 34.1, 31.2; IR (CH_2_Cl_2_): 3431, 3057, 2959, 1721, 1438, 1228, 1113, 724, 697, 549 cm^−1^; MS (EI): *m*/*z* (%) = 431(18), 430(36)[M^+^], 357(10), 301(7), 288(6), 229(12), 219(32), 202(59), 201(100), 184(68), 183(46), 155(59), 129(24), 105(23), 91(32), 77(25), 43(8); HRMS (EI): *m*/*z* calcd for C_27_H_27_O_3_P 430.1698; found 430.1705.

### 1-((2*E*)-2-Benzylidene-1-(diphenylphosphoryl)cyclopentyl) ethanone (54)

Prepared in reaction of 3-(diphenylphosphoryl)oct-7-yn-2-one and bromobenzene following general procedure (81 mg, yield 51%). Product was isolated as oil after column chromatography on silica gel (15 g, hex/AcOEt/dioxane 45 : 45 : 10). ^1^H NMR (400 MHz, CDCl_3_) *δ* 7.94–7.79 (m, 4H), 7.54–7.46 (m, 2H), 7.46–7.38 (m, 4H), 7.36–7.30 (m, 2H), 7.27–7.19 (m, 3H), 6.68 (s, 1H), 2.75–2.65 (m, 1H), 2.62–2.52 (m, 2H), 2.42 (s, 3H), 2.38–2.28 (m, 1H), 1.76–1.63 (m, 2H); ^13^C NMR (101 MHz, CDCl_3_) *δ* 205.3, 140.8 (d, *J* = 5.7 Hz), 137.5 (d, *J* = 2.8 Hz), 133.0 (d, *J* = 9.2 Hz), 132.6 (d, *J* = 8.6 Hz), 131.8 (d, *J* = 2.7 Hz), 131.7 (d, *J* = 2.7 Hz), 131.6 (d, *J* = 97.8 Hz), 131.0 (d, *J* = 97.8 Hz), 129.3 (d, *J* = 6.5 Hz), 128.6, 128.21, 128.17 (d, *J* = 11.4 Hz), 128.0 (d, *J* = 11.9 Hz), 127.0, 68.8 (d, *J* = 64.4 Hz), 32.8 (d, *J* = 3.7 Hz), 32.7, 28.3, 25.4 (d, *J* = 5.5 Hz); ^31^P NMR (162 MHz, CDCl_3_) *δ* 35.80, 34.60; IR (CH_2_Cl_2_): 3378, 3058, 2960, 2925, 2854, 1699, 1437, 1179, 1112, 750, 722, 696, 543 cm^−1^; MS (EI): *m*/*z* (%) = 400(24)[M^+^], 359(42), 358(100), 281(16), 219(24), 202(47), 201(98), 182(45), 167(53), 155(40), 128(34), 115(25), 105(21), 91(36), 77(63), 51(33), 43(65); HRMS (EI): *m*/*z* calcd for C_26_H_25_O_2_P 400.1592; found 400.1587.

### Dimethyl (2*E*)-2-(4-fluorobenzylidene)cyclopentane-1,1-dicarboxylate (55)

Prepared in reaction of dimethyl 4-pentenylmalonate and 1-chloro-4-fluorobenzene following modified general procedure (run at 80 °C) (74 mg, yield 63%). Product was isolated as oil after column chromatography on silica gel (15 g, hex/AcOEt 90 : 10). ^1^H NMR (400 MHz, CDCl_3_) *δ* 7.33–7.27 (m, 2H), 7.04–6.97 (m, 2H), 6.66 (t, *J* = 2.7 Hz, 1H), 3.76 (s, 6H), 2.66 (td, *J* = 7.2, 2.6 Hz, 2H), 2.38 (t, *J* = 6.9 Hz, 2H), 1.83 (p, *J* = 7.1 Hz, 2H); ^13^C NMR (101 MHz, CDCl_3_) *δ* 171.3, 162.8, 161.5 (d, *J* = 246.8 Hz), 140.6 (d, *J* = 2.1 Hz), 133.7 (d, *J* = 3.3 Hz), 130.2 (d, *J* = 8.0 Hz), 126.3, 115.0 (d, *J* = 21.5 Hz), 65.3, 52.7, 35.7, 31.8, 24.7; ^19^F NMR (376 MHz, CDCl_3_) *δ* −114.96; IR (CH_2_Cl_2_): 2954, 2879, 2842, 1733, 1603, 1508, 1434, 1227, 1190, 1159, 1098, 1065, 1014, 929, 885, 827, 773, 523 cm^−1^. MS (EI): *m*/*z* (%) = 293(6)[M^+^], 292(32), 260(13), 233(32), 232(44), 201(65), 200(21), 173(100), 146(23), 133(18), 109(44), 77(7), 59(16), 43(4); HRMS (EI): *m*/*z* calcd for C_16_H_17_O_4_F 292.1111; found 292.1117.

### Dimethyl (2*E*)-2-(4-(methoxycarbonyl)benzylidene)cyclopentane-1,1-dicarboxylate (56)

Prepared in reaction of dimethyl 4-pentenylmalonate and methyl 4-chlorobenzoate following modified general procedure (run at 80 °C) (78 mg, yield 59%, isomer *E*/*Z* 80 : 20). Product was isolated as oil after column chromatography on silica gel (25 g, hex/AcOEt 90 : 10 → 85 : 15). ^1^H NMR (400 MHz, CDCl_3_) *δ* 7.99–7.95 (m, 2H), 7.40–7.36 (m, 2H), 6.72 (t, *J* = 2.6 Hz, 1H), 3.88 (s, 3H), 3.76 (s, 6H), 2.70 (td, *J* = 7.2, 2.6 Hz, 2H), 2.38 (t, *J* = 7.0 Hz, 2H), 1.83 (p, *J* = 7.1 Hz, 2H); ^13^C NMR (101 MHz, CDCl_3_) *δ* 171.0, 166.8, 143.7, 142.0, 129.4, 128.5, 128.3, 126.7, 65.5, 52.8, 51.9, 35.6, 32.1, 24.7; indicative signals of minor isomer (*Z*): ^1^H NMR (400 MHz, CDCl_3_) *δ* 7.95–7.89 (m, 2H), 7.35 (s, 1H), 6.65 (d, *J* = 2.3 Hz, 1H), 3.87 (s, 3H), 3.43 (s, 5H), 2.67–2.62 (m, 2H), 2.42 (t, *J* = 7.0 Hz, 2H), 1.78–1.70 (m, 2H); ^13^C NMR (101 MHz, CDCl_3_) *δ* 170.7, 143.5, 141.4, 129.0, 128.3, 128.2, 125.8, 63.8, 52.4, 39.4, 34.8, 22.5, 14.0; IR (CH_2_Cl_2_): 2963, 2843, 1724, 1606, 1565, 1435, 1279, 1183, 1156, 1111, 1066, 1017, 966, 890, 777, 700, 522 cm^−1^; MS (EI): *m*/*z* (%) = 333(9)[M^+^], 332(39), 301(24), 273(24), 272(43), 242(32), 241(100), 240(48), 214(30), 213(98), 181(32), 155(35), 154(39), 153(48), 129(44), 128(19), 115(16), 105(6), 91(9), 77(12), 59(41), 41(4); HRMS (EI): *m*/*z* calcd for C_18_H_20_O_6_ 332.1260; found 332.1266.

## Conclusions

In summary, we developed an efficient protocol for tandem Pd-catalyzed intramolecular addition of active methylene compounds to alkynes, followed by subsequent cross-coupling with (hetero)aryl bromides and chlorides. The methodology features exceptional tolerance to functional groups (including unprotected OH, NH_2_, or enolizable ketones), broad applicability of aryl and heteroaryl bromides of different electronic properties, as well as a range of active methylene partners, including acetylenic derivatives of malonates, cyanomalonates, β-ketoesters, β-diketones, cyanoacetates, and organophosphorus compounds. Mechanistic studies revealed a plausible mechanism comprising oxidative addition of haloarene, nucleophilic addition to alkyne activated by coordination to aryl–Pd(ii), and reductive elimination. However, for the transformations of less C–H acidic substrates (*e.g.* β-ketoesters, β-diketones) and electron-deficient haloarenes, an alternative path involving *syn*-carbometallation may operate in parallel.

## Conflicts of interest

There are no conflicts to declare.

## Supplementary Material

RA-009-C9RA08002C-s001
