# Supplementary material for: Pd-catalyzed intramolecular addition of active methylene compounds to alkynes with subsequent cross-coupling with (hetero)aryl halides
Source: RSC Adv. 2019 Dec 3;9(68):40152–67. doi: 10.1039/c9ra08002c (PMC9076196; doi:10.1039/c9ra08002c)
Supplement: RA-009-C9RA08002C-s001 [file RA-009-C9RA08002C-s001.pdf]

Electronic Supplementary Material (ESI) for RSC Advances.
This journal is © The Royal Society of Chemistry 2019

Supporting information

Pd-catalyzed Intramolecular Addition of Active Methylene Compounds to Alkynes with Subsequent Cross-coupling with (Hetero)aryl Halides

Aleksandra Błocka,[a] Paweł Woźnicki,[b] Marek Stankevič,[b] and Wojciech Chaładaj[a,*]

[a]*Institute of Organic Chemistry, Polish Academy of Sciences, Kasprzaka 44/52, 01-224 Warsaw, Poland*
[b]*Department of Organic Chemistry, Faculty of Chemistry, Marie Curie-Skłodowska University in Lublin, Gliniana 33, 20-614 Lublin, Poland*
**wojciech.chaladaj@icho.edu.pl*

Table of contents

Table of contents 1
General Information 1
Evaluation of reaction conditions for Pd-catalyzed cyclization/coupling of dimethyl pent-4-yn-1-ylmalonate with bromobenzene. 2
Synthesis and analytical data of acetylenic active methylene compounds 6
Copies of ^{1}H and $^{13}C\{^{1}H\}$ NMR spectra of isolated compounds 12
References 107

General Information

All the manipulations were performed in a nitrogen-filled glovebox or under an argon atmosphere using Schlenk techniques, unless mentioned otherwise. Flash chromatography was performed using Merck silica gel 60 (230-400 mesh). TLC analysis of reaction mixtures was performed on Merck silica gel 60 F254 TLC plates and visualized with cerium molybdate stain (Hanessian's stain). ^{1}H, $^{13}C\{1H\}$, and ^{19}F NMR spectra were recorded with a Bruker AV 400 spectrometer. ^{1}H and ^{13}C chemical shifts are given in ppm relative to TMS. The solvent signals were used as references ($CDCl_3 \delta_H$ = 7.26 ppm, δ_C = 77.0 ppm) and the chemical shift converted to the TMS scale. Coupling constants (J) are reported in Hz, and the following abbreviations were used to denote multiplets: s = singlet, d = doublet, t = triplet, q = quartet, quint = quintet, m = multiplet (denotes complex pattern), dd = doublet of doublets, dt = doublet of triplets and br = broad signal. Infrared spectra were recorded with a Jasco FTIR-6200 spectometer. Electron ionization high-resolution mass spectra (EI-HR) were recorded with an Autospec Premier (Waters Inc) mass spectrometer using the narrow-range high-voltage scan technique with low-boiling perfluorokerosene (PFK) as internal standard. Samples were introduced by using a heated direct insertion probe. Electrospray ionization high-resolution mass spectra (ESI-HR) were recorded with MALDISynapt G2-S HDMS (Waters Inc) mass spectrometer equipped with an electrospray ion source and q-TOF type mass analyzer. ESI-MS spectra were recorded in the positive ion mode (the source parameters: capillary voltage 3.15 kV, sampling cone 25 V, source temperature 120 °C, desolvation temperature 150 °C). GC analyses were performed on Agilent 7890B Gas Chromatograph equipped with FID detector and HP-5 column (30m, 0.32 mmID, 0.25 µm). The following temperature program was used: 100 °C (2 min), 20 °C/min to 310 °C (2 min). Unless otherwise noted, all commercially available compounds (ABCR, Acros, Fluorochem, TCI, Sigma-Aldrich, Strem) were used as received. Phosphine ligands were purchased from Aldrich or Fluorochem, $Pd(OAc)_2$ was purchased Strem. Precatalysts L Pd G3 were prepared following Buchwald's procedure,[1] and showed similar reactivity to the commercial sample purchased from Strem (as tested for XPhos Pd G3). Substrates for cyclization-coupling were prepared by alkylation of appropriate active methylenes following standard procedures.

Evaluation of reaction conditions for Pd-catalyzed cyclization/coupling of dimethyl pent-4-yn-1-ylmalonate with bromobenzene.

General procedure for evaluation of reaction conditions: In a glovebox, to a 4-mL screw-capped vial containing catalyst (typically 2 mol%) following reagents were added: dimethyl pent-4-yn-1-ylmalonate (19.8 mg, 0.100 mmol), bromobenzene (23.6 mg, 0.150 mmol), base (0.150 mmol) and solvent (0.5 ml). Then, magnetic stirring bar was placed and the vial was sealed with a cap containing a PTFE septum. The reaction mixture was stirred at 50 °C for 4 h and then cooled to room temperature. The mixture was diluted with MTBE (2 mL) quenched with sat. aqueous NH_4Cl (0.5 mL) and mesitylene (25 µl) was added as a internal standard.

Table S1. Effect of catalyst[a]

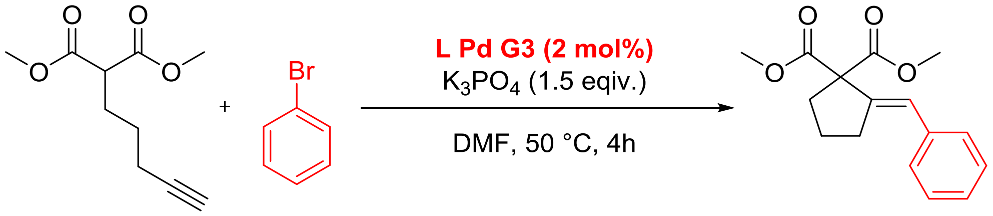

Entry	Catalyst	Yield[b]
1	XPhos Pd G3	61 %
2	*t*-BuXPhos Pd G3	5 %
3	SPhos Pd G3	25 %
4	MonoPhos Pd G3	41 %
5	RuPhos Pd G3	61 %
6	DPPF Pd G3	9 %
7	DCyPF Pd G3	13 %
8	DPPB Pd G3	28 %
9	BINAP Pd G3	24 %
10	DPPE Pd G3	21 %
11	PPh_3 Pd G3	29 %
12	$(tol)_3P$ Pd G3	17 %
13	XantPhos Pd G3	13 %
14	$P(Cy)_3$ Pd G3	50 %
15	Tol-BINAP Pd G3	26 %
16	CataCXium A Pd G3	3 %

[a]Conditions: L Pd G3 (2 mol%), dimethyl pent-4-yn-1-ylmalonate (0.100 mmol, 1 equiv.), bromobenzene (0.150 mmol, 1.5 equiv.), K_3PO_4 (0.150 mmol, 1.5 equiv.), DMF (0.5 mL), 50 ^oC, 4h. [b]Yield was determined by GC with mesitylene as an internal standard.

BINAP Pd G3 was chosen as a catalyst due to lower amount of by-product and easier isolation of expected product from the reaction mixture than in case when PAd_2(*n*-Bu) Pd G3 was used.

Table S2. Effect of solvent[a]

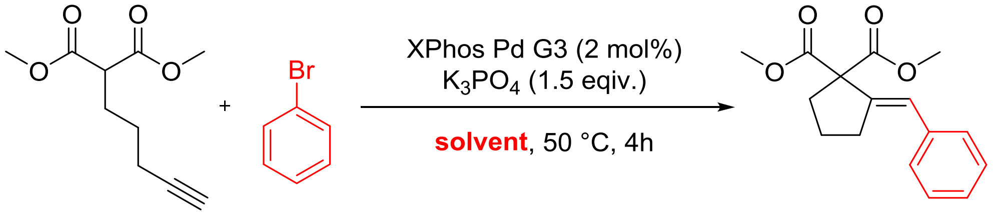

Entry	Solvent	Yield[b]
1	DMF	61%
2	THF	2%
3	Toluene	1%
4	Dioksane	3%
5	CH_2Cl_2	1%
6	DMF	23%
7	DMSO	27%
8	NMP	19%
9	AcCN	8%
10	MeOH	1%

[a]Conditions: XPhos Pd G3 (2 mol%), dimethyl pent-4-yn-1-ylmalonate (0.100 mmol, 1 equiv.), bromobenzene (0.150 mmol, 1.5 equiv.), K_3PO_4 (0.150 mmol, 1.5 equiv.), solvent (0.5 mL), 50 °C, 4h.
[b]Yield was determined by GC with mesitylene as an internal standard.

Table S3. Effect of base[a]

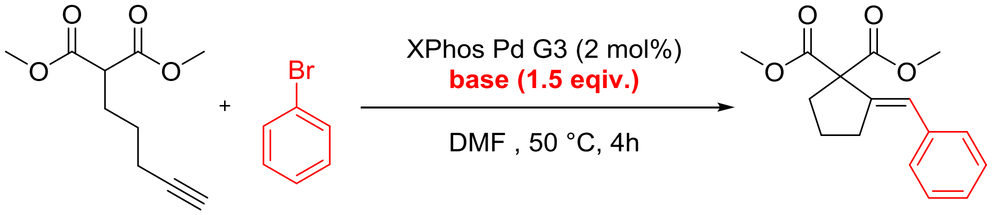

Entry	Base	Yield[b]
1	t-BuOLi	4%
2	t-BuONa	61%
3	t-BuOK	0%
4	LiHMDS	6%
5	KHMDS	0%
6	KOMe	0%
7	AcONa	0%
8	AcOCs	0%
9	KOH	77%
10	NaOH	5%
11	NaHMDS	0%
12	Cs_2CO_3	76%
13	K_2CO_3	22%
14	K_3PO_4	61%

[a]Conditions: XPhos Pd G3 (2 mol%), dimethyl pent-4-yn-1-ylmalonate (0.100 mmol, 1 equiv.), bromobenzene (0.150 mmol, 1.5 equiv.), base (0.150 mmol, 1.5 equiv.), DMF (0.5 mL), 50 °C, 4h.
[b]Yield was determined by GC with mesitylene as an internal standard.

Table S4. Effect of stoichiometry of reagents[a]

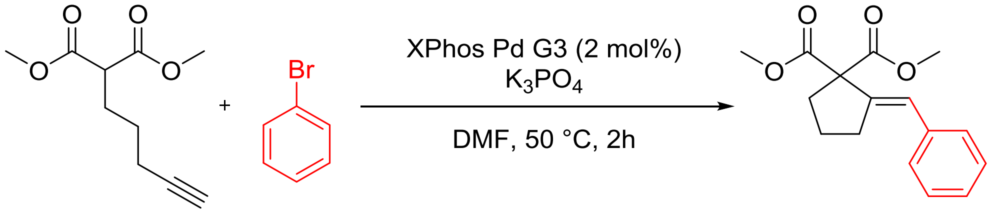

Entry	Amount of malonate	Amount of ArBr	Amount of K_3PO_4	Yield[b]
1	1 eqiv.	1 eqiv.	1 eqiv.	14%
2	1 eqiv.	2 eqiv.	1 eqiv.	23%
3	2 eqiv.	1 eqiv.	1 eqiv.	15%
4	1 eqiv.	1 eqiv.	2 eqiv.	26%

[a]Conditions: XPhos Pd G3 (2 mol%), DMF (0.5 mL), 50 °C, 2h.
[b]Yield was determined by GC with mesitylene as an internal standard.

Table S5. Effect of catalyst loading[a]

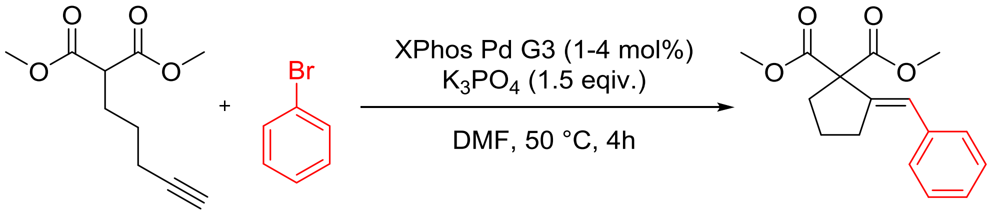

Entry	Catalyst loading	Yield[b]
1	4 mol %	79%
2	2 mol%	61%
3	1 mol%	22%

[a]Conditions: XPhos Pd G3 (1-4 mol%), dimethyl pent-4-yn-1-ylmalonate (0.100 mmol, 1 equiv.), bromobenzene (0.150 mmol, 1.5 equiv.), K_3PO_4 (0.150 mmol, 1.5 equiv.), DMF (0.5 mL), 50 °C, 4h.
[b]Yield was determined by GC with mesitylene as an internal standard.

Table S6. Effect of temperature and reaction time[a]

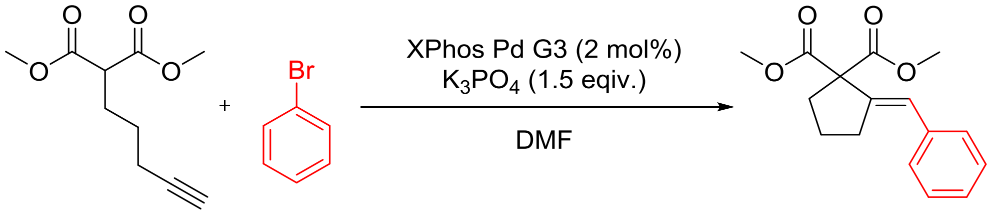

Entry	Rxn. time	Temperature	Yield[b]
1	0.5 h	50°C	16%
2	1 h	50°C	21%
3	2 h	50°C	23%
4	4 h	50°C	60%
5	24 h	50°C	89%
6	4 h	80°C	34%
7	24 h	80°C	68%

[a]Standard conditions: XPhos Pd G3 (2 mol%), dimethyl pent-4-yn-1-ylmalonate (0.100 mmol, 1 equiv.), bromobenzene (0.150 mmol, 1.5 equiv.), K_3PO_4 (0.150 mmol, 1.5 equiv.), DMF (0.5 mL).
[b]Yield was determined by GC with mesitylene as an internal standard.

Table S6. Effect of concentration[a]

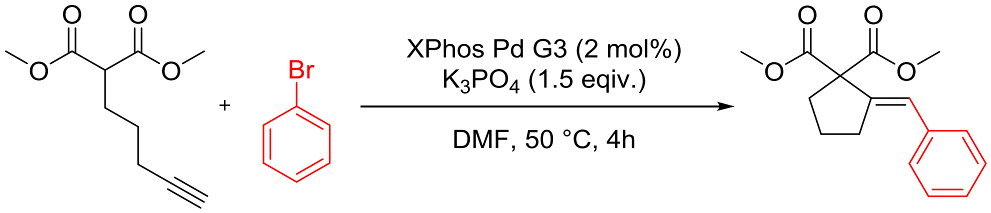

Entry	Solvent amount	Yield[b]
1	0.25 ml	59%
2	**0.5 ml**	61%
3	1.0 ml	26%
4	2.0 ml	8%

[a]Standard conditions: XPhos Pd G3 (2 mol%), dimethyl pent-4-yn-1-ylmalonate (0.100 mmol, 1 equiv.), bromobenzene (0.150 mmol, 1.5 equiv.), K_3PO_4 (0.150 mmol, 1.5 equiv.), DMF, 50 °C, 4h.
[b]Yield was determined by GC with mesitylene as an internal standard.

Synthesis and analytical data of acetylenic active methylene compounds

The list of acetylenic active methylene compounds used in the work is depicted in Figure 1. Compounds **1**, **65**, **S5** are known and were obtained according to literature procedure. Their spectra were in accordance with reported data.[2–4]

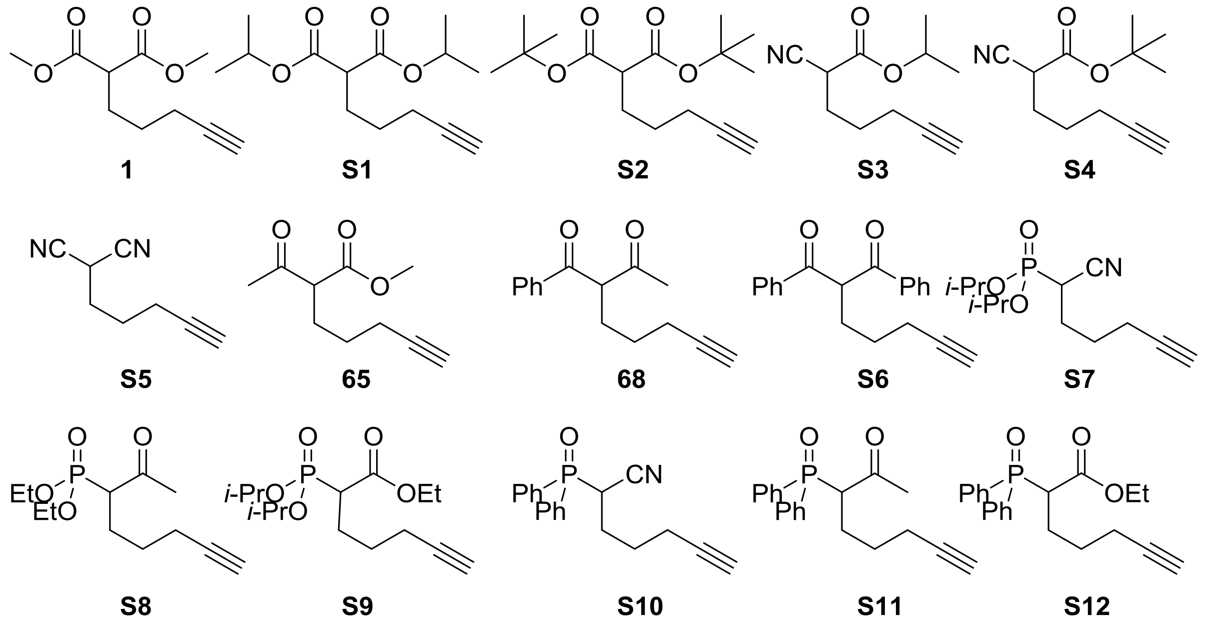

Figure 1. Acetylenic Active methylene compounds

Compounds **S1**, **S2**, **S3**, **S4** were obtained by alkylation of commercial active methylene with 5-iodopent-1-yne according to General Procedure A.

General procedure A. To a suspension of sodium hydride (15 mmol, 1.5 eqiv., 60% in mineral oil) in dry DMF (15 ml), malonate ester (cyanoacetate or malononitrile) (12 mmol, 1.2 equiv.) was added dropwise at 0°C. Mixture was stirred for 30 minutes, next 5-iodo-1-pentyne was added dropwise (10 mmol. 1 equiv.). Mixture was heated at 60 °C for 16 hours, then cooled down and quenched with diethyl ether/water/NH_4Cl mixture. Aqueous layer was washed with diethyl ether (3x30 ml), then combined organic phases was dried over sodium sulfate. Product was isolated as colorless oil after column chromatography (250g of silica, hexanes:AcOEt 90:10). The isolated product was further purified by distillation under reduced pressure.

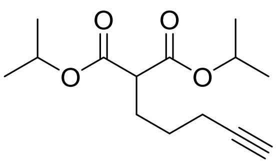

S1

Dipropan-2-yl pent-4-yn-1-ylpropanedioate (S1) Prepared in reaction of di-*iso*-propyl malonate (2.26 g, 12 mmol) following General procedure A (1.51 g, 5.9 mmol, yield: 59 %). ^{1}H NMR (400 MHz, $CDCl_3$) δ 5.05 (p, J = 6.3 Hz, 2H), 3.26 (t, J = 7.5 Hz, 1H), 2.22 (td, J = 7.0, 2.7 Hz, 2H), 2.02 – 1.96 (m, 2H), 1.95 (m, 1H), 1.61 – 1.53 (m, 2H), 1.24 (m, 6H), 1.24 – 1.22 (m, 6H). ^{13}C NMR (101 MHz, $CDCl_3$) δ 168.8, 83.5, 68.8, 51.9, 27.6, 26.1, 21.6, 21.6, 18.2; IR (CH_2Cl_2): 3455, 3286, 2981, 2875, 2118, 1727, 1468, 1455, 1375, 1105, 909, 822, 635 cm^{-1}; MS (EI): m/z (%) = 195(4), 188(12), 170(7), 153(17), 146(21), 126(26), 125(30), 104(28), 97(12), 81(41), 79(33), 55(16), 43(100); HRMS (EI): m/z calcd for $C_{14}H_{22}O_4$ 254.1518; found 254.1511.

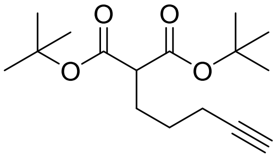

S2

Di-*tert*-butyl pent-4-yn-1-ylpropanedioate (S2). Prepared in reaction of di-*tert*-butyl malonate (2.60 g, 12 mmol) following General procedure A (1.13 g, 4.0 mmol, yield: 40 %). ^{1}H NMR (400 MHz, $CDCl_3$) δ 3.13 (t, *J* = 7.5 Hz, 1H), 2.21 (td, *J* = 7.1, 2.7 Hz, 2H), 1.95 – 1.87 (m, 3H), 1.61 – 1.51 (m, 2H), 1.45 (s, 18H); ^{13}C NMR (101 MHz, $CDCl_3$) δ 168.7, 83.7, 81.4, 68.7, 53.5, 27.9, 27.7, 26.1, 18.2; IR (CH_2Cl_2): 3293, 2979, 2934, 2119, 1727, 1457, 1369, 1140 cm^{-1}; HRMS (ESI): m/z calcd for $C_{16}H_{26}O_4Na$ 305.1729; found 305.1715.

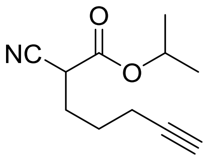

S3

Propan-2-yl 2-cyanohept-6-ynoate (S3). Prepared in reaction of *iso*-propyl cyanoacetate (1.53 g, 12 mmol) following General procedure A (0.78 g, 4.1 mmol, yield: 41 %). ^{1}H NMR (400 MHz, $CDCl_3$) δ 5.08 – 4.97 (m, 1H), 3.51 – 3.44 (m, 1H), 2.27 – 2.17 (m, 2H), 2.08 – 1.98 (m, 2H), 1.96 – 1.91 (m, 1H), 1.71 – 1.60 (m, 2H), 1.28 – 1.19 (m, 6H); ^{13}C NMR (101 MHz, $CDCl_3$) δ 165.2, 116.2, 82.5, 70.7, 69.4, 37.2, 28.5, 25.2, 21.3, 17.6; IR (CH_2Cl_2): 3290, 2984, 2251, 2119, 1740 cm^{-1}; MS (EI): 151(4), 134(8), 127(8), 106(40), 80(33), 79(48), 77(14), 67(24), 54(20), 43(100), 41(46); HRMS (ESI) m/z calcd for $C_{11}H_{14}NO_2$ 192.1025; found 192.1026.

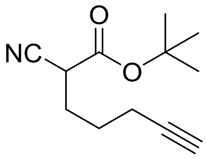

S4

***tert*-butyl 2-cyanohept-6-ynoate (S4).** Prepared in reaction of *tert*-butyl cyanoacetate (1.63g, 12 mmol) following General procedure A (0.41 g, 1.98 mmol, yield: 20 %). ^{1}H NMR (400 MHz, $CDCl_3$) δ 3.43 (dd, *J* = 7.6, 6.4 Hz, 1H), 2.27 (td, *J* = 6.8, 2.6 Hz, 2H), 2.10 – 1.99 (m, 2H), 1.98 (t, *J* = 2.7 Hz, 1H), 1.78 – 1.65 (m, 2H), 1.50 (s, 9H); ^{13}C NMR (101 MHz, $CDCl_3$) δ 164.8, 116.6, 84.1, 82.7, 69.5, 38.1, 28.7, 27.8, 25.4, 17.8; IR (CH_2Cl_2): 3292, 2981, 2938, 2872, 2250, 2118, 1739, 1458, 1371, 1284, 1259,1152, 840, 644 cm^{-1}; HRMS (ESI): m/z calcd for $C_{12}H_{16}NO_2$ 206.1181; found 206.1176

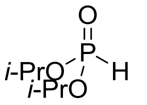

Di-*iso*-propyl phosphonate. In an oxygen and moisture-free one-necked round-bottom flask (50 mL) equipped with magnetic stirrer and inert gas inlet triisopropyl phosphite (10 mL, 8.44 g, 40.53 mmol) was dissolved in THF (15 mL). After cooling the solution in an ice bath degassed distilled water was added (0.73 mL, 40.53 mmol) and the mixture was stirred at room temperature. After 24 h due to incomplete conversion of the substrate as evidenced by a ^{31}P NMR experiment another equivalent of water was added (0.73 mL, 40.53 mmol) and the mixture was stirred for further 24 h. Then the solvent was evaporated under reduced pressure and the residue was dried by azeotropic distillation with 10 mL of toluene. The crude oil was purified by Kugelrohr distillation (65-68 ^{o}C/4 mmHg) yielding diisopropyl phosphonate as a colorless oil (6.42 g, 95%). ^{1}H NMR (500 MHz, $CDCl_3$) δ 6.85 (d, *J* = 687.6 Hz, 1H), 4.68-4.78 (m, 2H), 1.36 (dd, J_1 = 6.0 Hz, J_2 = 3.2 Hz, 12H); ^{13}C NMR (126 MHz, $CDCl_3$) δ 70.8 (d, *J* = 6.4 Hz), 23.9 (dd, J_1 = 25.4 Hz, J_2 = 4.5 Hz); ^{31}P NMR (202 MHz, CDCl3) δ 4.47. Analytical data are in accordance with the literature.[5,6]

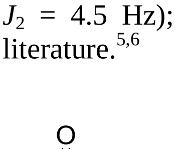

2-(Diphenylphosphoryl)acetonitrile (S13). In an oxygen and moisture-free round-bottom Schlenk flask (25 mL) equipped with magnetic stirrer and inert gas inlet was placed methyl diphenylphosphinite (3.3 g, 1.53 mmol) (prepared from Ph_2PCl, MeOH and NEt_3) followed by chloroacetonitrile (1.26 mL, 1.98 mmol). The mixture was heated at 135 ^{o}C with stirring for one hour. After cooling to room temperature, the residue was dissolved in chloroform and purified using column chromatography with $CHCl_3$:MeOH 50:1 (v/v) as an eluent affording **1a** (1.84 g, 45%) as a white solid; mp = 143.7-145.3 ^{o}C; R_f = 0.60 ($CHCl_3$/MeOH 50:1); ^{1}H NMR (500 MHz, $CDCl_3$) δ 7.81-7.88 (m, 4H), 7.62-7.68 (m, 2H), 7.54-7.59 (m, 4H), 3.35 (d, *J* = 15.1 Hz, 2H); ^{13}C NMR (126 MHz, $CDCl_3$) δ 133.2 (d, *J* = 2.8 Hz), 131.1 (d, *J* = 10.0 Hz), 129.6 (d, *J* = 106.1 Hz), 129.0 (d, *J* = 12.7 Hz), 113.4 (d, *J* = 7.3 Hz), 21.3 (d, *J* = 61.8 Hz); ^{31}P NMR (202 MHz, CDCl3) δ 24.50; GC-MS (EI, 70 eV) *m/z* = 202 (13), 201 (100), 77 (27), 51 (17); HRMS (ESI): m/z calcd for $C_{28}H_{24}N_2O_2P_2Na$ ($[2M+Na]^+$) 505.1205; found 505.1201

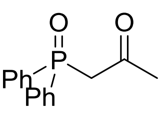

1-(Diphenylphosphoryl)propan-2-one (S14). In an oxygen and moisture-free round-bottom Schlenk flask (25 mL) equipped with magnetic stirrer and inert gas inlet

was placed methyl diphenylphosphinite (3.3 g, 1.53 mmol) (prepared from Ph_2PCl, MeOH and NEt_3) followed by chloroacetone (1.6 mL, 1.98 mmol). The mixture was heated at 135 °C with stirring for 1.5 hour. After cooling to room temperature, the residue was dissolved in chloroform and purified using column chromatography with $CHCl_3$:MeOH 50:1 (v/v) as an eluent affording **1b** (2.54 g, 64%) as a white solid; mp = 143.7-145.3 °C; R_f = 0.43 ($CHCl_3$/MeOH 50:1); ^1H NMR (500 MHz, $CDCl_3$) δ 7.55-7.60 (m, 4H), 7.18-7.31 (m, 6H), 2.07 (s, 3H), 1.78 (d, J = 13.1 Hz, 2H); ^{13}C NMR (126 MHz, $CDCl_3$) δ 200.9 (d, J = 5.0 Hz), 132.2 (d, J = 2.9 Hz), 131.8 (d, J = 102.7 Hz), 130.8 (d, J = 9.8 Hz), 128.7 (d, J = 12.7 Hz), 47.9 (d, J = 56.7 Hz), 32.6; ^{31}P NMR (202 MHz, CDCl3) δ 26.23; GC-MS (EI, 70 eV) *m/z* = 258 (M) (28), 257 (13), 216 (19), 215 (45), 202 (13), 201 (100), 143 (16), 91 (15), 77 (39), 51 (19), 47 (11); HRMS (ESI): m/z calcd for $C_{30}H_{30}O_4P_2Na$ ($[2M+Na]^+$) 539.1510; found 539.1512.

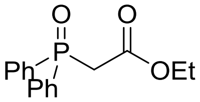

Ethyl 2-(diphenylphosphoryl)acetate (S15). In an oxygen and moisture-free round-bottom Schlenk flask (25 mL) equipped with magnetic stirrer and inert gas inlet was placed methyl diphenylphosphinite (5.5 g, 2.54 mmol) (prepared from Ph_2PCl, MeOH and NEt_3) followed by ethyl chloroacetate (2.72 mL, 2.54 mmol). The mixture was heated at 120 °C with stirring for 24 hours. After cooling to room temperature, the residue was dissolved in chloroform and purified using column chromatography with $CHCl_3$:MeOH 50:1 (v/v) as an eluent affording **1b** (4.24 g, 58%) as a white solid; mp = 72.0-73.1 °C; R_f = 0.73 ($CHCl_3$/MeOH 50:1); ^1H NMR (500 MHz, $CDCl_3$) δ 7.74-7.85 (m, 4H), 7.52-7.59 (m, 2H), 7.43-7.52 (m, 4H), 4.00 (q, J = 7.3 Hz, 2H), 3.49 (d, J = 14.8 Hz, 2H), 1.03 (t, J = 7.3 Hz, 3H); ^{13}C NMR (126 MHz, $CDCl_3$) δ 166.1 (d, J = 4.5 Hz), 132.2, 131.8 (d, J = 104.5 Hz), 131.1 (d, J = 10.0 Hz), 128.6 (d, J = 11.8 Hz), 61.5, 39.2 (d, J = 60.0 Hz), 13.8; ^{31}P NMR (202 MHz, CDCl3) δ 26.69; GC-MS (EI, 70 eV) *m/z* = 288 (M) (7), 216 (10), 215 (10), 202 (14), 201 (100), 199 (11), 91 (10), 77 (22), 51 (10); HRMS (ESI): m/z calcd for $C_{16}H_{17}O_3PNa$ ($[M+H]^+$) 289.0988; found 289.0988.

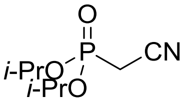

Diisopropyl (cyanomethyl)phosphonate (S16). In an oxygen and moisture-free round-bottom Schlenk flask (25 mL) equipped with magnetic stirrer and inert gas inlet was placed triisopropyl phosphite (2.11 g, 10.13 mmol) and chloroacetonitrile (0.956 g, 12.66 mmol). The mixture was heated neat to 135°C over 1 h and then stirred at this temperature for 2.5 h. The desired product was obtained as a slightly yellow oil (1.77 g, 85%) after removal of volatiles under reduced pressure (65°C/1 mmHg). ^1H NMR (500 MHz, $CDCl_3$) δ 4.77-4.87 (m, 2H), 2.82 (d, J = 20.7 Hz, 2H), 1.38-1.44 (m, 12H); ^{13}C NMR (126 MHz, $CDCl_3$) δ 112.9 (d, J = 11.8 Hz), 73.0 (d, J = 6.4 Hz), 23.9 (d, J = 4.5 Hz), 23.8 (d, J = 5.0 Hz), 17.4 (d, J = 144.4 Hz); ^{31}P NMR (202 MHz, CDCl3) δ 12.11; GC-MS (EI, 70 eV) *m/z* = 148 (83), 123 (26), 122 (100), 131 (10), 104 (11), 45 (20); HRMS (ESI): m/z calcd for $C_8H_{16}NO_3PNa$ ($[M+Na]^+$) 228.0760; found 228.0769. Analytical data are in accordance with the literature.[7]

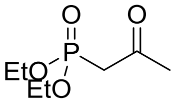

Diethyl (2-oxopropyl)phosphonate (S17). In an oxygen and moisture-free round-bottom Schlenk flask (25 mL) equipped with magnetic stirrer and inert gas inlet was placed methyl chlroacetate (4.64 mL, 5.76 mmol) and KI (9.38 g, 5.65 mmol) in acetone (20 mL). The mixture was vigorously stirred at room temperature for 2 h. Then, triethyl phosphite (9.5 mL, 5.54 mmol) in diethyl ether (20 mL) was added and the mixture was heated to reflux for 2.5 h. The mixture was allowed to cool to room temperature and then filtered through a Celite. The filtrate was evaporated to dryness and the residue was distilled under reduced pressure affording **2b** (5.64 g, 52%) as a colorless liquid; bp = 104-110 °C/2 mm Hg; ^1H NMR (500 MHz, $CDCl_3$) δ 4.09-4.19 (m, 4H), 3.07 (d, J = 22.9 Hz, 2H), 2.31 (s, 3H), 1.33 (d, J = 7.1 Hz, 6H); ^{13}C NMR (126 MHz, $CDCl_3$) δ 199.9 (d, J = 6.3 Hz), 62.5 (d, J = 6.9 Hz), 43.3 (d, J = 128.4 Hz), 31.3, 16.2 (d, J = 6.1 Hz); ^{31}P NMR (202 MHz, CDCl3) δ 19.66; GC-MS (EI, 70 eV) *m/z* = 194 (M) (9), 179 (16), 167 (15), 152 (49), 151 (23), 149 (21), 139 (19), 125 (100), 124 (10), 123 (48), 121 (56), 109 (45), 108 (31), 105 (12), 97 (87), 96 (27), 91 (14), 81 (41), 80 (32), 79 (14), 78 (15), 65 (22), 58 (26), 47 (12), 45 (12); HRMS (ESI): m/z calcd for $C_7H_5O_4PNa$ ($[M+H]^+$) 195.0781; found 195.0774.

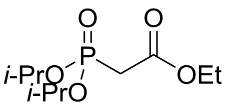

Ethyl 2-(diisopropoxyphosphoryl)acetate (S18). In an oxygen and moisture-free two-necked round-bottom flask (100 mL) equipped with magnetic stirrer and inert gas inlet was placed triisopropyl phosphite (8.44 g, 40.53 mmol) and ethyl chloroacetate (5.15 g, 42.04 mmol). The mixture was heated neat to 135°C over 1 h and then stirred at this temperature for 2.5 h. The desired product was obtained as a colorless oil (8.1 g, 79%) after removal of volatiles under reduced pressure (65 °C/1 mmHg). ^{1}H NMR (500 MHz, $CDCl_3$) δ 4.71–4.81 (m, 2H), 4.20 (q, J = 7.3 Hz, 2H), 2.92 (d, J = 21.8 Hz, 2H), 1.35 (d, J = 6.3 Hz, 12H), 1.29 (t, J = 7.2 Hz, 3H); ^{13}C NMR (126 MHz, $CDCl_3$) δ 166.0 (d, J = 6.4 Hz), 71.4 (d, J = 7.3 Hz), 61.4, 35.5 (d, J = 134.4 Hz), 24.0 (d, J = 3.8 Hz), 23.8 (d, J = 5.0 Hz), 14.1; ^{31}P NMR (202 MHz, CDCl3) δ 17.62; GC-MS (EI, 70 eV) *m/z* = 169 (51), 165 (10), 151 (40), 141 (9), 123 (100), 105 (15), 96 (12), 45 (10); HRMS (ESI): m/z calcd for $C_{10}H_{21}O_5PNa$ ([M+Na]$^+$) 275.1019; found 275.1023.

General procedurę B (for alkylation of phosphine oxides and phosphonates). In an oxygen and moisture-free Schlenk tube (50 mL) equipped with a magnetic stirrer and an inert gas inlet was placed phosphine oxide or phosphonate in THF (0.15–0.20 M solution). After cooling to 0°C NaH (1.05–1.10 eq.) was added, after 15 min. the cooling bath was removed and a solution of 5-iodopent-1-yne (1.25–1.50 eq.) in THF (2 mL) was added. The mixture was then heated to 65°C and stirred for 16 h. Then saturated NH_4Cl solution (10 mL) and water (10 mL) were added. The aqueous phase was extracted with DCM (3x12mL), combined organic phases were dried over $MgSO_4$, filtered, and evaporated under reduced pressure. The residue was purified using column chromatography.

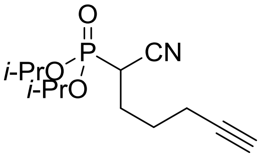

Diisopropyl (1-cyanohex-5-yn-1-yl)phosphonate (S7). This compound was prepared according to the general procedure using **S16** (0.970 g, 4.72 mmol), 60% NaH dispersion in mineral oil (0.198 g, 4.96 mmol), 5-iodopent-1-yne (1.287 g, 6.61 mmol) as a colorless oil; yield: 0.575 g (45%); *Rf* = 0.60 (hexane/EtOAc 1:1); ^{1}H NMR (500 MHz, $CDCl_3$) δ 4.77-4.87 (m, 2H), 2.86-2.95 (m, 1H), 2.28-2.33 (m, 2H), 2.02-2.12 (m, 1H), 1.99 (t, J = 2.5 Hz, 1H), 1.86-2.00 (m, 2H), 1.66-1.75 (m, 1H), 1.36-1.41 (m, 12H); ^{13}C NMR (126 MHz, $CDCl_3$) δ 116.3 (d, J = 9.1 Hz), 82.6, 73.0 (d, J = 7.7 Hz), 72.7 (d, J = 7.1 Hz), 69.5, 30.9, 29.8, 26.4 (d, J = 12.7 Hz), 26.0 (d, J = 4.5 Hz), 24.01 (d, J = 4.1 Hz), 23.98 (d, J = 3.6 Hz), 23.85 (d, J = 4.4 Hz), 23.82 (d, J = 5.4 Hz), 17.7; ^{31}P NMR (202 MHz, CDCl3) δ 15.60; GC-MS (EI, 70 eV) *m/z* = 188 (20), 187 (44), 170 (15), 134 (11), 132 (37), 123 (32), 122 (12), 121 (31), 108 (18), 107 (100), 106 (71) 105 (11), 80 (38), 79 (34), 77 (15), 67 (17), 65 (13), 59 (11), 54 (23), 45 (11); HRMS (ESI): m/z calcd for $C_{13}H_{22}NO_3PNa$ ([M+Na]$^+$) 294.1230; found 294.1235.

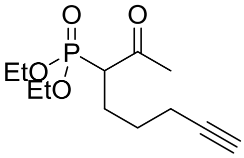

Diethyl (2-oxooct-7-yn-3-yl)phosphonate (S8). This compound was prepared according to the general procedure using **S17** (0.830 g, 4.27 mmol), 60% NaH dispersion in mineral oil (0.188 g, 4.70 mmol), 5-iodopent-1-yne (0.994 g, 5.12 mmol) as a colorless oil; yield: 0.366 g (33%); *Rf* = 0.39 (hexane/EtOAc/MeOH 8:3:1); ^{1}H NMR (500 MHz, $CDCl_3$) δ 4.09-4.18 (m, 4H), 3.15-3.25 (m, 1H), 2.34 (s, 3H), 2.20-2.25 (m, 2H), 2.07-2.16 (m, 1H), 1.97 (t, J = 2.5 Hz, 1H), 1.88-1.95 (m, 1H), 1.46-1.56 (m, 2H), 1.34 (dt, J_1 = 7.2 Hz, J_2 = 2.8 Hz, 6H); ^{13}C NMR (126 MHz, $CDCl_3$) δ 203.5 (d, J = 3.6 Hz), 83.3, 69.0, 62.7 (d, J = 6.4 Hz), 62.5 (d, J = 7.3 Hz), 53.2 (d, J = 124.9 Hz), 31.1, 27.0 (d, J = 14.5 Hz), 25.4 (d, J = 5.4 Hz), 18.1, 16.3 (d, J = 5.4 Hz); ^{31}P NMR (202 MHz, CDCl3) δ 21.98. GC[a] t_R = 8.24 min; GC-MS (EI, 70 eV) *m/z* = 218 (26), 217 (10), 194 (27), 190 (13), 189 (17), 179 (11), 177 (13), 167 (9), 165 (11), 162 (22), 161 (35), 151 (15), 139 (17), 138 (32), 137 (16), 123 (24), 122 (25), 121 (9), 111 (43), 110 (14), 109 (96), 108 (64), 107 (18), 105 (22), 97 (9), 93 (27), 91 (31), 83 (17), 82 (30), 81 (82), 80 (100), 79 (96), 78 (14), 77 (32), 67 (18), 65 (29), 55 (10), 53 (11); HRMS (ESI): m/z calcd for $C_{12}H_{21}O_4PNa$ ([M+Na]$^+$) 283.1070; found 283.1067.

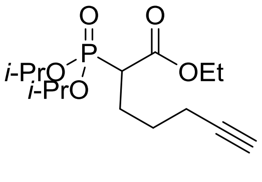

Ethyl 2-(diisopropoxyphosphoryl)hept-6-ynoate (S9). This compound was prepared according to the general procedure using **S18** (0.697 g, 2.76 mmol), 60% NaH dispersion in mineral oil (0.116 g, 2.90 mmol), 5-iodopent-1-yne (0.804 g, 4.14 mmol) as a colorless oil; yield: 0.398 g (45%); *Rf* = 0.38 (hexane/EtOAc 1:1); ^{1}H NMR (500 MHz, $CDCl_3$) δ 4.67-4.77 (m, 2H), 4.20 (q, J = 7.3 Hz, 2H), 2.84-2.95 (m, 1H), 2.18-2.23 (m, 2H), 2.19-2.26 (m, 2H), 1.90-2.11 (m, 2H), 1.48-1.65 (m, 2H), 1.30-1.36 (m, 12H), 1.28 (t, J = 7.3 Hz, 3H); ^{13}C NMR (126 MHz, $CDCl_3$) δ 169.1 (d, J = 5.5 Hz), 83.4, 71.5 (d, J = 6.2 Hz), 71.2 (d, J = 7.1 Hz), 68.8, 61.2, 45.2 (d, J = 133.4 Hz), 27.1 (d, J = 15.4 Hz), 26.2 (d, J = 5.5 Hz), 24.1 (d, J = 3.6 Hz), 24.0 (d, J = 3.5 Hz), 23.8 (d, J = 5.4 Hz), 23.7 (d, J = 5.5 Hz), 18.0, 14.2; ^{31}P NMR (202 MHz, CDCl3) δ 20.31. GC[a] t_R = 8.63 min; GC-MS (EI, 70 eV) *m/z* = 235 (28), 217 (24), 211 (17), 195 (9), 189 (54), 171 (13), 169 (33), 168 (25), 165 (14), 161 (24), 153 (11), 141 (13), 123 (24), 109 (46), 108 (21), 107 (27), 99 (14), 97 (10), 96 (20), 91 (12), 82 (10), 81 (27), 80 (30), 79 (100), 78 (16), 77 (27) 65 (16), 55 (22); HRMS (ESI): m/z calcd for $C_{15}H_{27}O_5P$ Na ($[M+Na]^+$) 341.1488; found 341.1486.

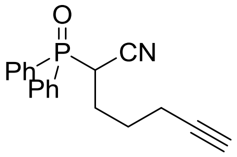

2-(Diphenylphosphoryl)hept-6-ynenitrile (S10). This compound was prepared according to the general procedure using **S13** (0.715 g, 2.96 mmol), 60% NaH dispersion in mineral oil (0.142 g, 3.56 mmol), and 5-iodopent-1-yne (1.014 g, 5.23 mmol) as a white solid; yield: 0.448 g (49%); mp = 115.9–117.1 °C; R_f = 0.33 (hexane/EtOAc 1:1); ^{1}H NMR (500 MHz, $CDCl_3$) δ 7.94-8.01 (m, 2H), 7.84-7.91 (m, 2H), 7.61-7.68 (m, 2H), 7.52-7.60 (m, 4H), 3.42-3.54 (m, 1H), 2.25 (td, J_1 = 6.6 Hz, J_2 = 2.5 Hz, 2H), 2.16-2.26 (m, 1H), 1.94 (t, J = 2.5 Hz, 1H), 1.78-1.95 (m, 3H), 1.68–1.78 (m, 1H); ^{13}C NMR (126 MHz, $CDCl_3$) δ 133.1 (d, J = 2.7 Hz), 133.0 (d, J = 2.7 Hz), 131.9 (d, J = 9.1 Hz), 131.2 (d, J = 10.0 Hz), 129.7 (d, J = 103.5 Hz), 129.0 (d, J = 11.8 Hz), 128.9 (d, J = 11.8 Hz), 128.0 (d, J = 101.7 Hz), 117.1 (d, J = 3.6 Hz), 82.5, 69.6, 32.8 (d, J = 63.6 Hz), 26.6 (d, J = 10.0 Hz), 24.9, 17.6; ^{31}P NMR (202 MHz, CDCl3) δ 27.69; GC-MS (EI, 70 eV) *m/z* = 307 [M] (3), 306 (10), 202 (15), 201 (100), 77 (26), 51 (13); HRMS (ESI): m/z calcd for $C_{19}H_{18}NOPNa$ ($[M+Na]^+$) 330.1018; found 330.1019.

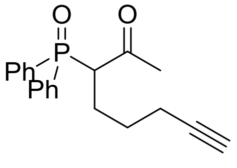

3-(Diphenylphosphoryl)oct-7-yn-2-one (S11). This compound was prepared according to the general procedure using **S14** (0.504 g, 1.95 mmol), 60% NaH dispersion in mineral oil (0.086 g, 2.15 mmol), 5-iodopent-1-yne (0.561 g, 2.89 mmol) as a white solid; yield: 0.392 g (62%); mp = 160.4–161.5 °C; *Rf* = 0.32 (chloroform/MeOH 50:1); ^{1}H NMR (500 MHz, $CDCl_3$) δ 7.76-7.85 (m, 4H), 7.52-7.57 (m, 2H), 7.46-7.52 (m, 4H), 3.60 (dt, J_1 = 11.7 Hz, J_2 = 2.8 Hz, 1H), 2.21-2.35 (m, 1H), 2.21 (s, 3H), 2.12-2.17 (m, 2H), 1.87 (t, J = 2.8 Hz, 1H), 1.77-1.86 (m, 1H), 1.48-1.59 (m, 1H), 1.38-1.48 (m, 1H); ^{13}C NMR (126 MHz, $CDCl_3$) δ 205.1, 132.25 (d, J = 2.7 Hz), 132.17 (d, J = 2.7 Hz), 131.4, 131.1 (d, J = 9.1 Hz), 131.0 (d, J = 99.4 Hz), 130.9 (d, J = 97.5 Hz), 128.8 (d, J = 4.5 Hz), 128.7 (d, J = 5.4 Hz), 83.0, 69.2, 56.9 (d, J = 56.3 Hz), 30.6, 27.1 (d, J = 12.7 Hz), 25.3 (d, J = 2.7 Hz), 17.8; ^{31}P NMR (202 MHz, CDCl3) δ 28.48; GC-MS (EI, 70 eV) *m/z* = 323 [M-H] (15), 282 (11), 281 (35), 258 (25), 243 (17), 220 (10), 219 (56), 202 (48), 201 (100), 183 (12), 155 (15), 141 (12), 129 (13), 91 (11), 78 (9), 77 (48), 51 (15), 47 (20); HRMS (ESI): m/z calcd for $C_{20}H_{21}O_2PNa$ ($[M+Na]^+$) 347.1171; found 347.1162.

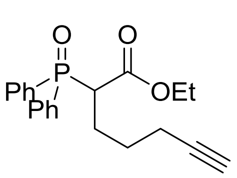

Ethyl 2-(diphenylphosphoryl)hept-6-ynoate (S12). This compound was prepared according to the general procedure using **S15** (0.584 g, 2.02 mmol), 60% NaH dispersion in mineral oil (0.086 g, 2.12 mmol), 5-iodopent-1-yne (0.494 g, 2.54 mmol) as a white solid; yield: 0.188 g (26%); mp = 102.2–103.5 °C; *Rf* = 0.35 (hexane/EtOAc 1:2); ^{1}H NMR (500 MHz, $CDCl_3$) δ 0.91 (t, J = 7.25 Hz, 3H), 1.49-1.59 (m, 1H), 1.59-1.69 (m, 1H), 1.88 (t, J = 2.5 Hz, 1H), 1.89-1.98 (m, 1H), 2.11-2.21 (m, 3H), 3.45-3.52 (m, 1H), 3.81-3.96 (m, 2H), 7.45-7.51 (m, 4H), 7.42-7.57 (m, 2H), 7.79-7.85 (m, 2H), 7.85-7.91 (m, 2H); ^{13}C NMR (126 MHz, $CDCl_3$) δ 169.6, 132.12 (d, J = 2.7 Hz), 132.09 (d, J = 3.6 Hz), 131.6 (d, J = 9.1 Hz), 131.4 (d, J = 100.6 Hz), 131.2 (d, J = 9.1 Hz), 130.4 (d, J = 99.9 Hz), 128.6 (d, J = 11.8 Hz), 128.4 (d, J = 11.8 Hz), 83.2, 68.9, 61.3, 48.8 (d, J = 59.0 Hz), 27.3 (d, J = 12.7 Hz), 25.5, 17.9, 13.6; ^{31}P NMR (202 MHz, CDCl3) δ 29.36. GC-MS (EI,

70 eV) *m/z* = 353 [M-H] (19), 288 (16), 281 (11), 219 (28), 216 (20), 215 (20), 202 (32), 201 (100), 183 (10), 155 (11), 141 (9), 77 (45), 51 (13), 47 (12); HRMS (ESI): m/z calcd for $C_{21}H_{23}O_3PNa$ ($[M+Na]^+$) 377.1277; found 377.1277.

Copies of ^{1}H and $^{13}C\{^{1}H\}$ NMR spectra of isolated compounds

Dimethyl (2*E*)-2-benzylidenecyclopentane-1,1-dicarboxylate (2).

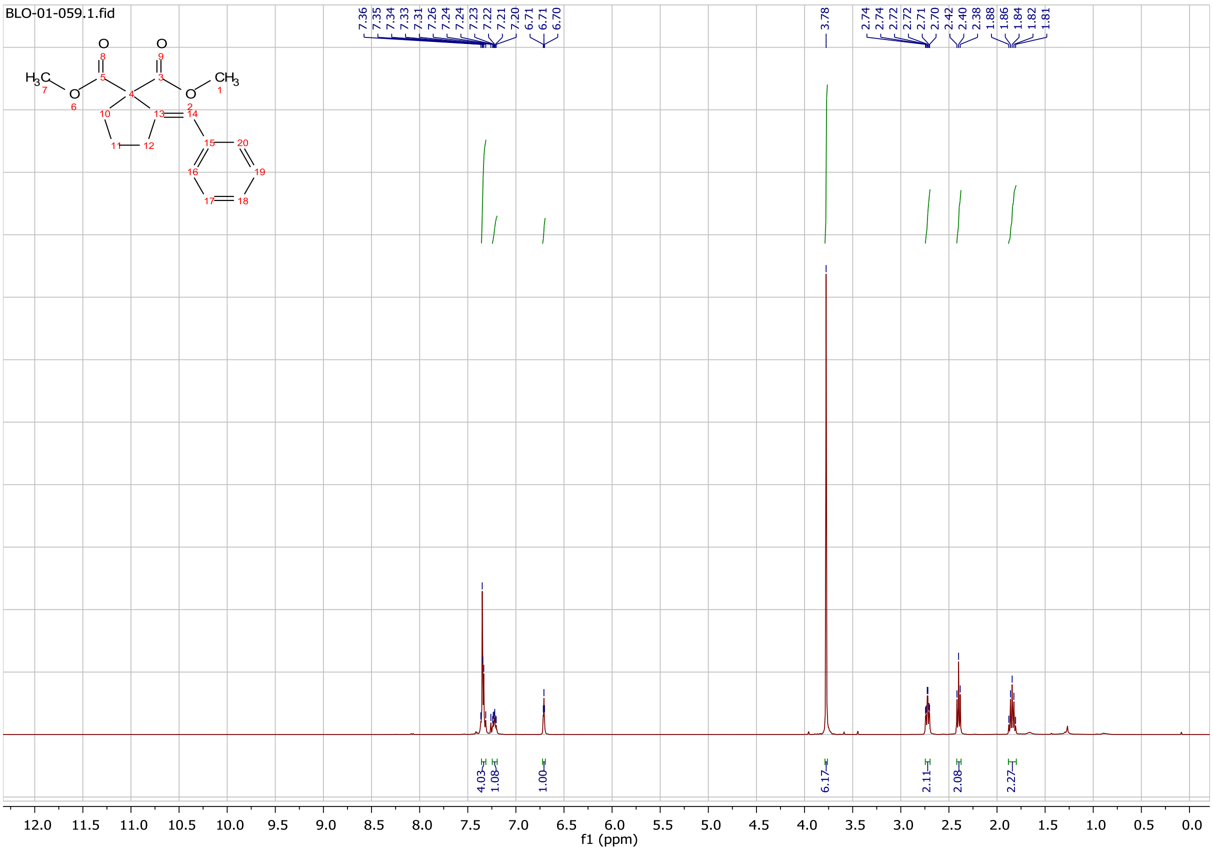

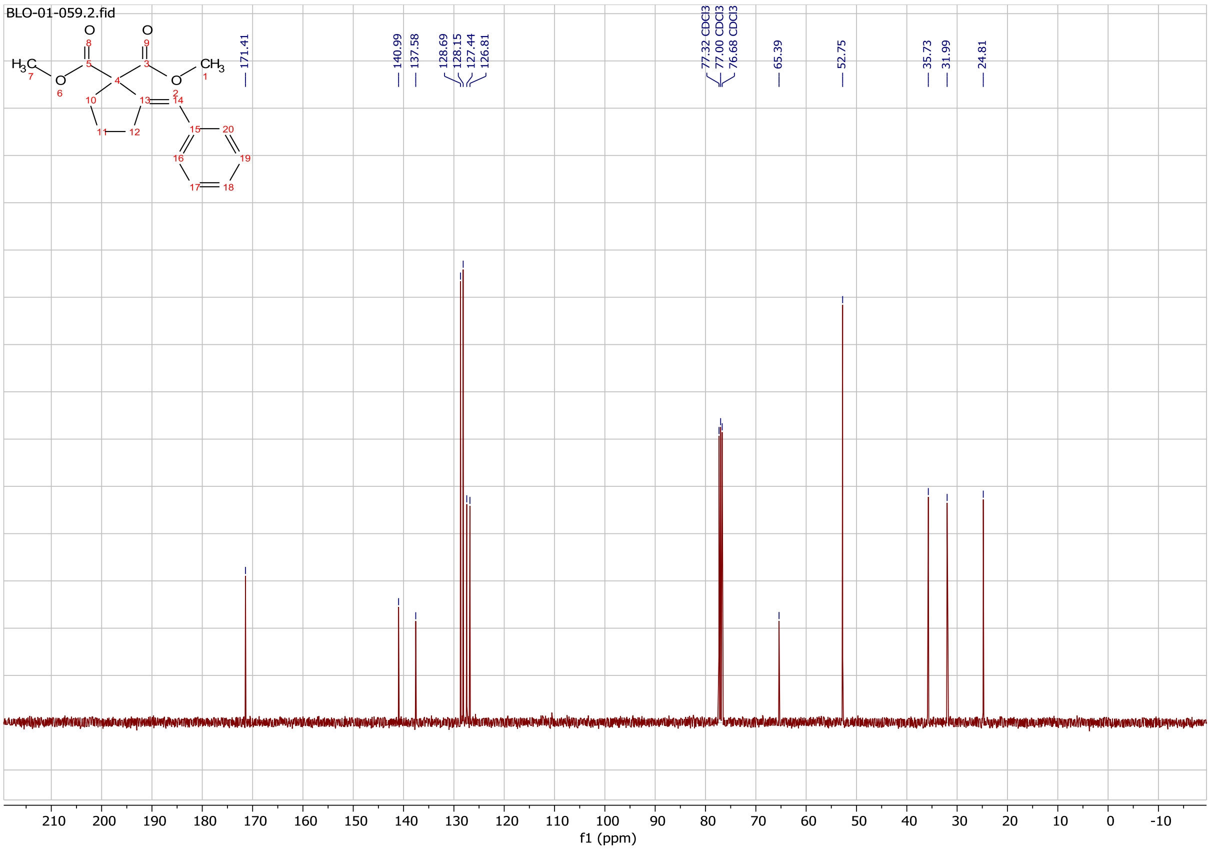

Dimethyl (2*E*)-2-(4-methoxybenzylidene)cyclopentane-1,1-dicarboxylate (3).

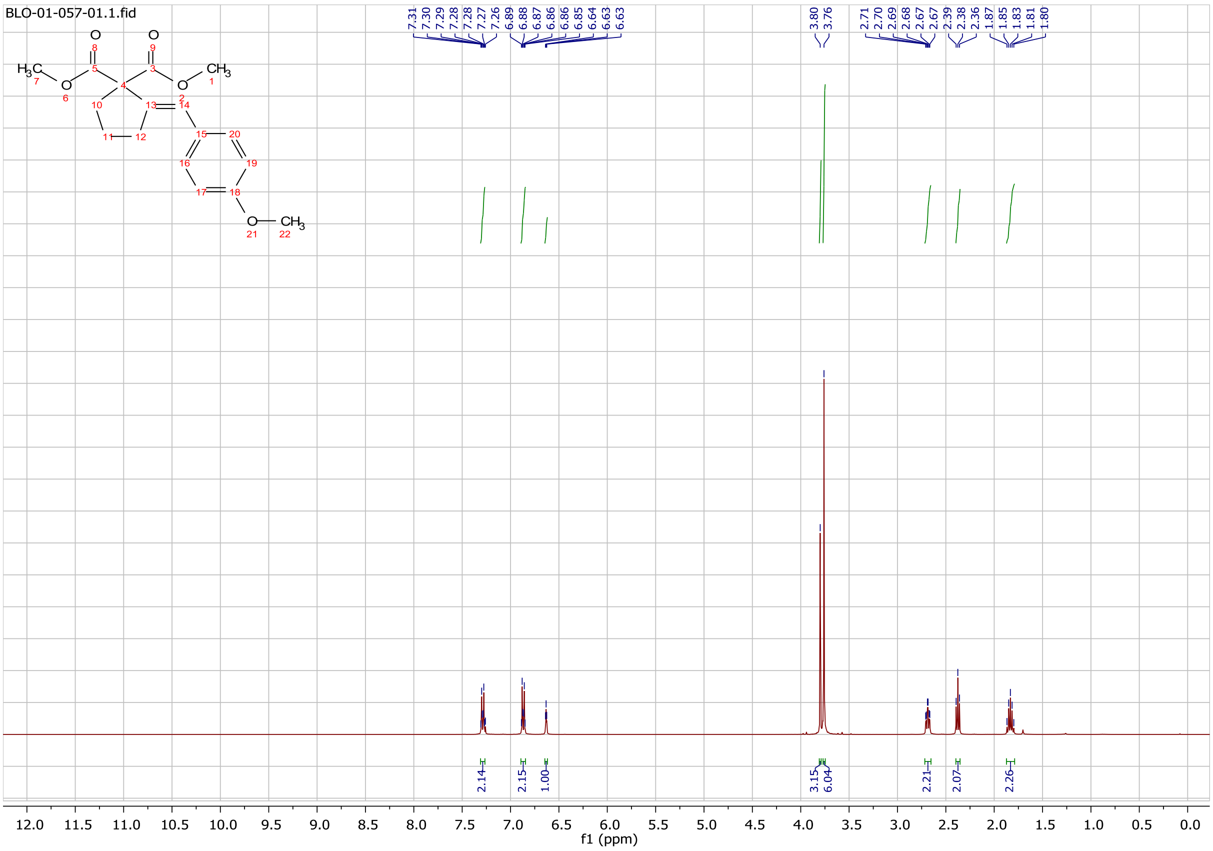

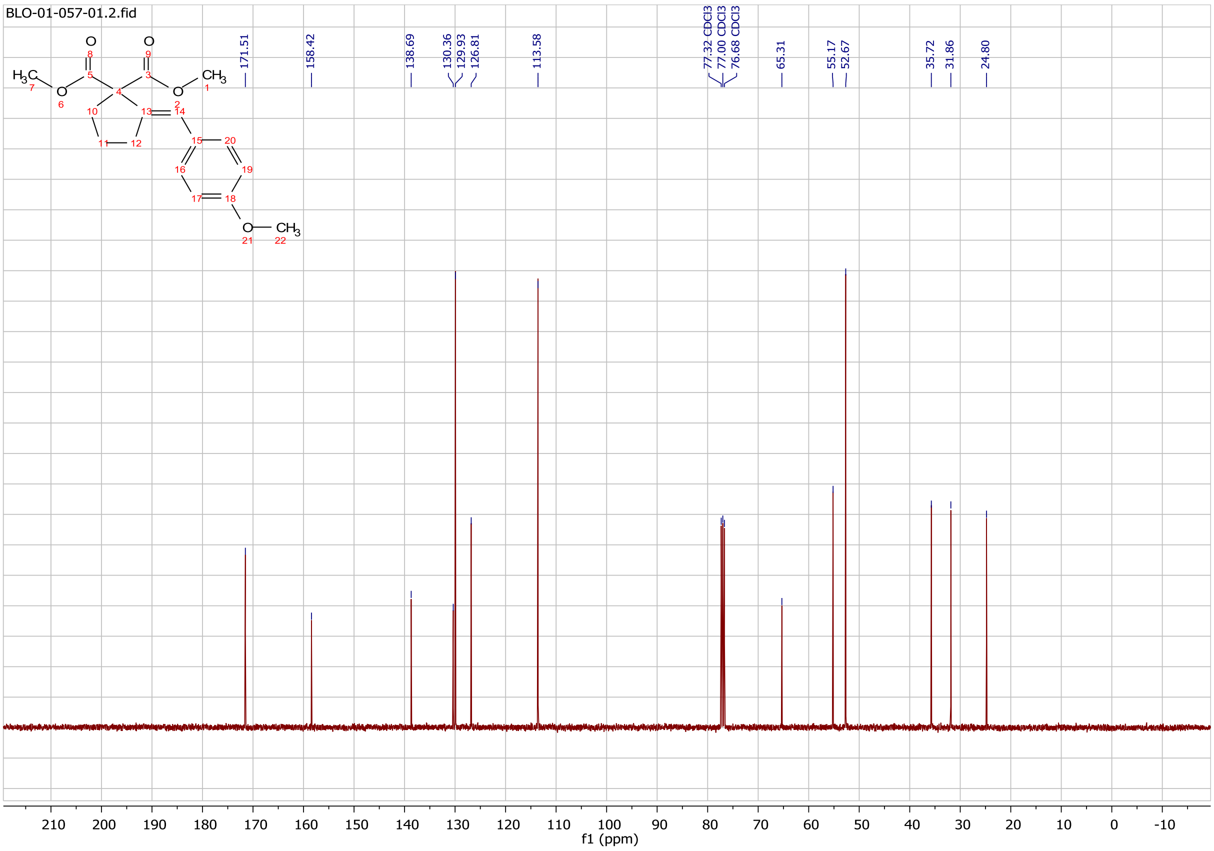

Dimethyl (2*E*)-2-(4-aminobenzylidene)cyclopentane-1,1-dicarboxylate (4).

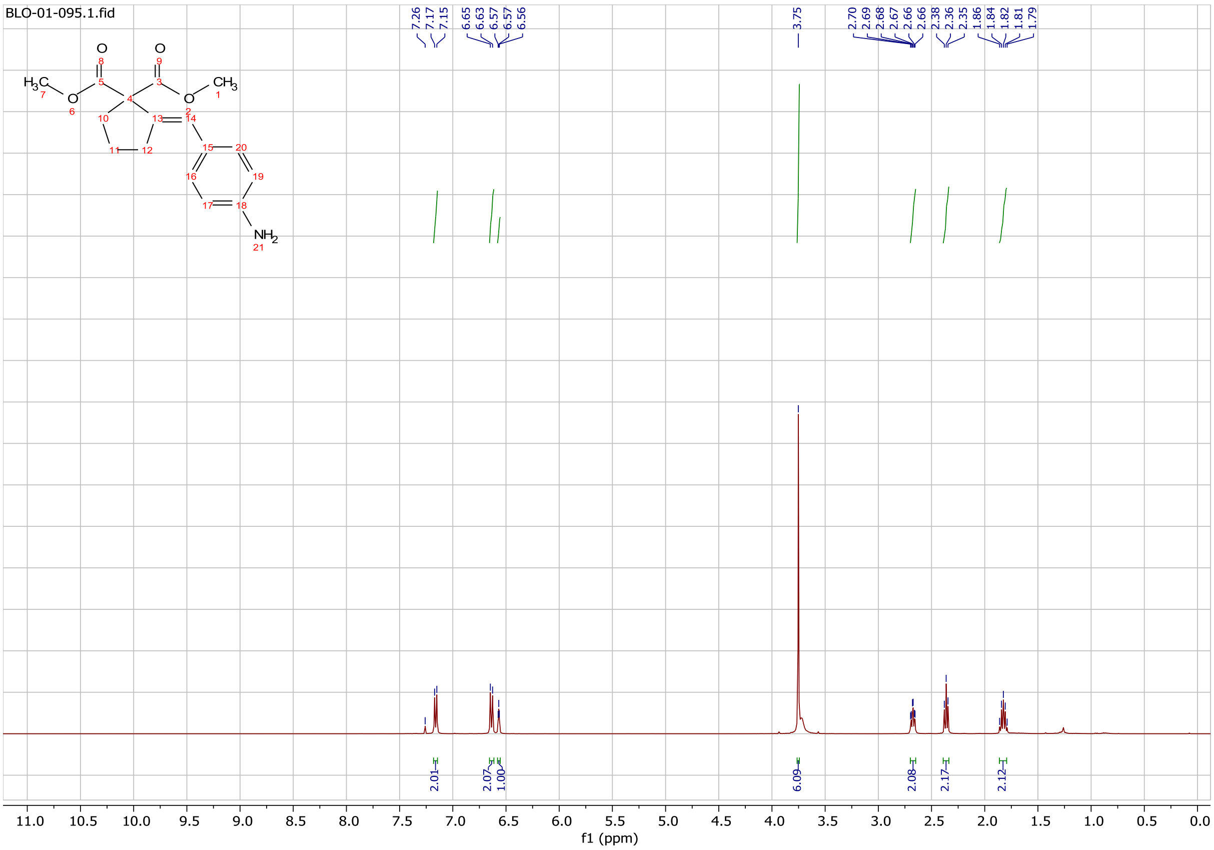

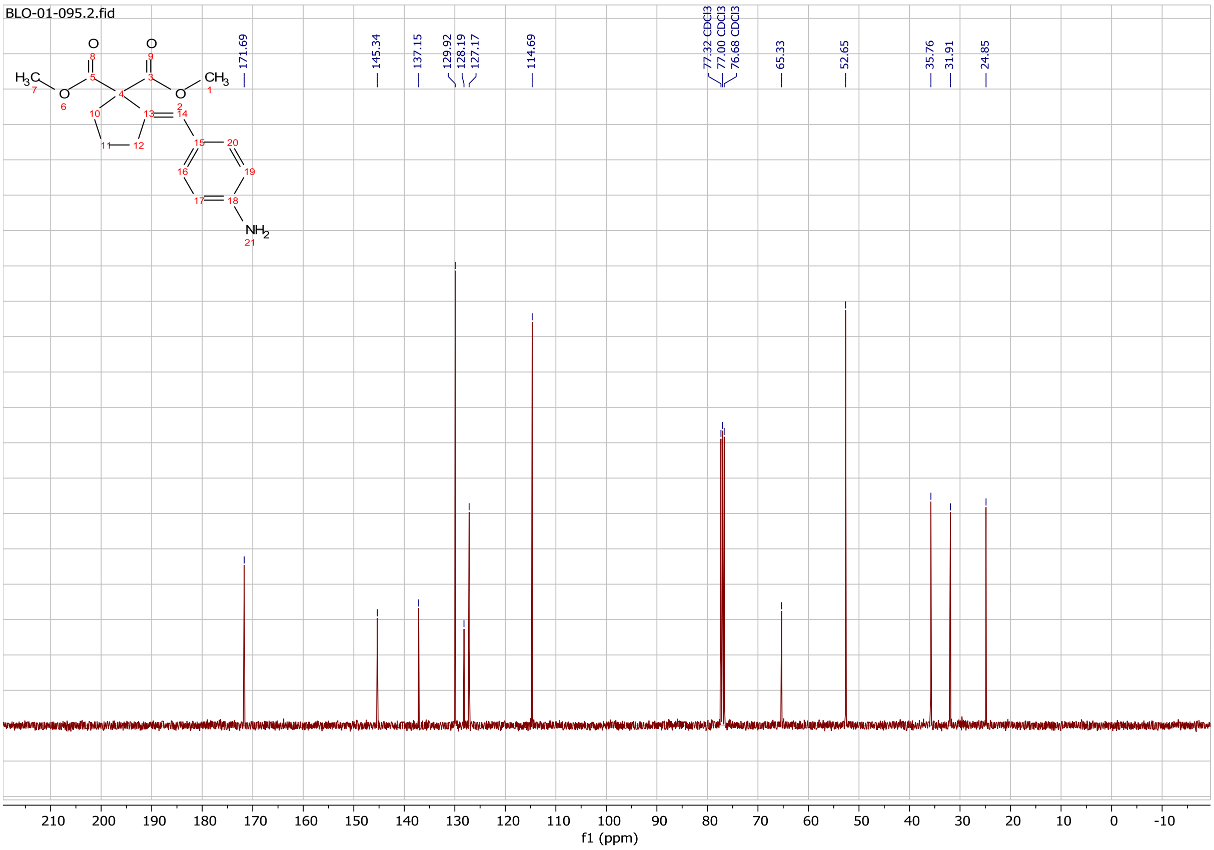

Dimethyl (2*E*)-2-(4-(dimethylamino)benzylidene)cyclopentane-1,1-dicarboxylate (5).

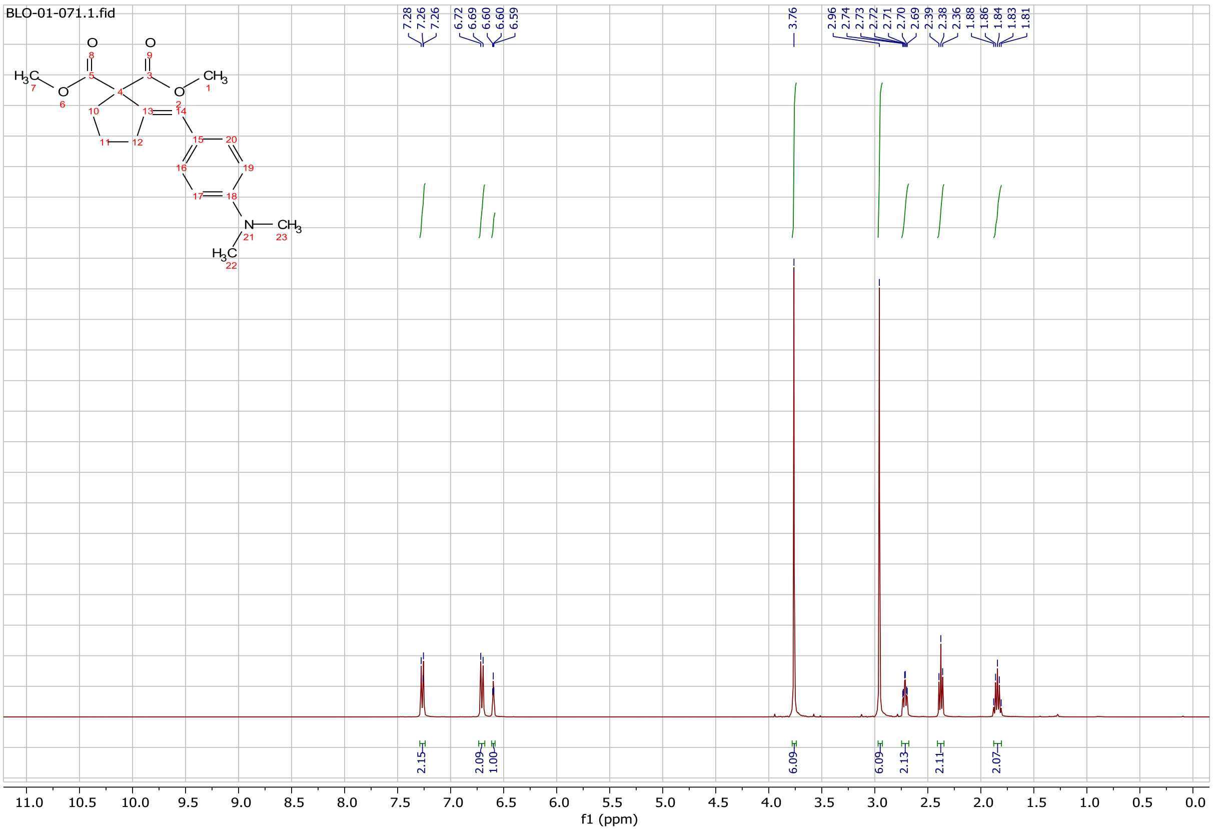

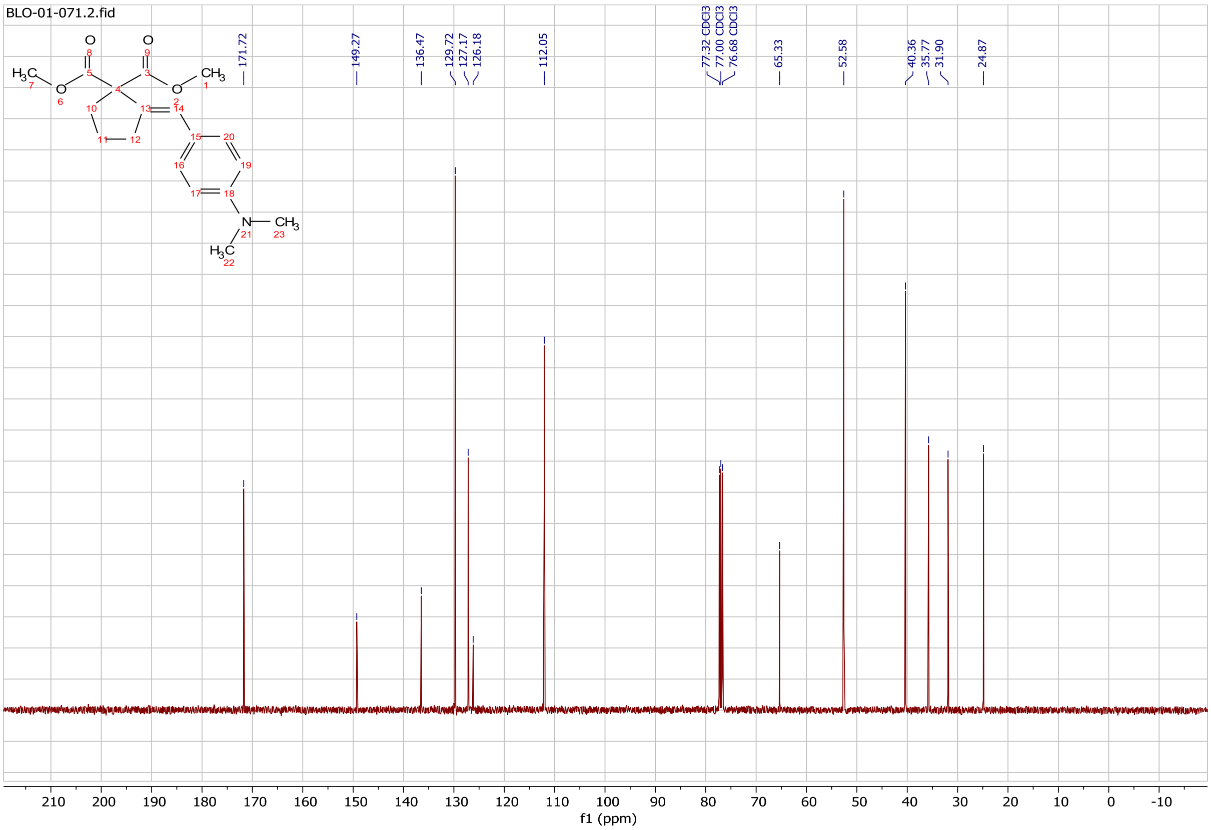

Dimethyl (2*E*)-2-(4-(trifluoromethyl)benzylidene)cyclopentane-1,1-dicarboxylate (6).

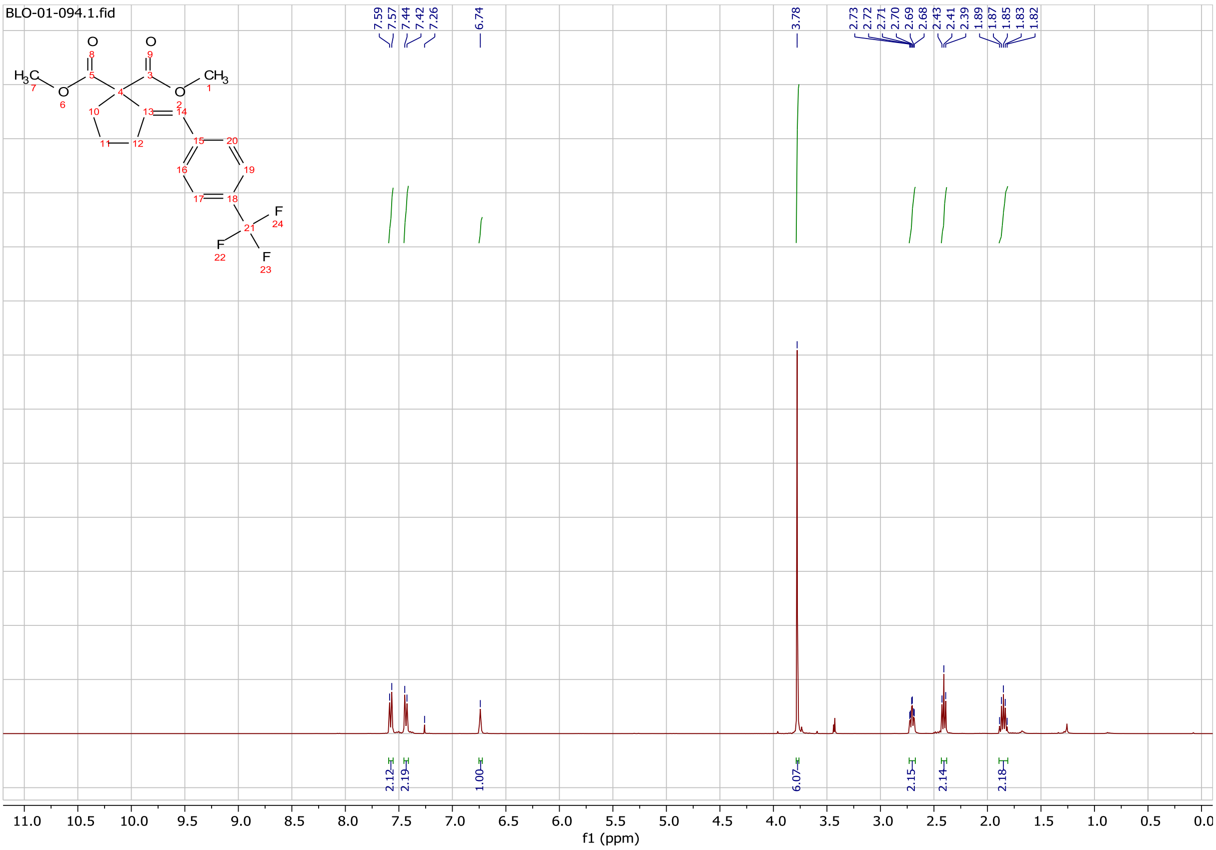

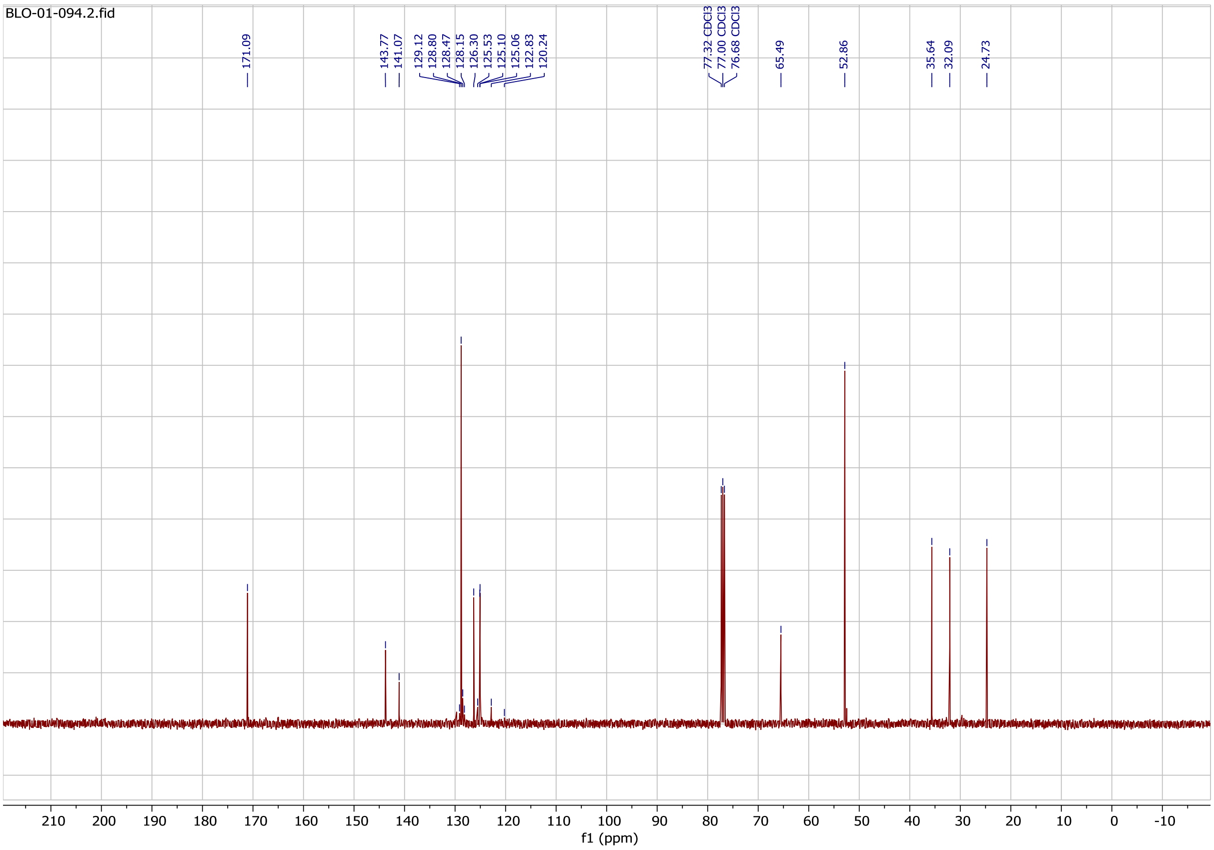

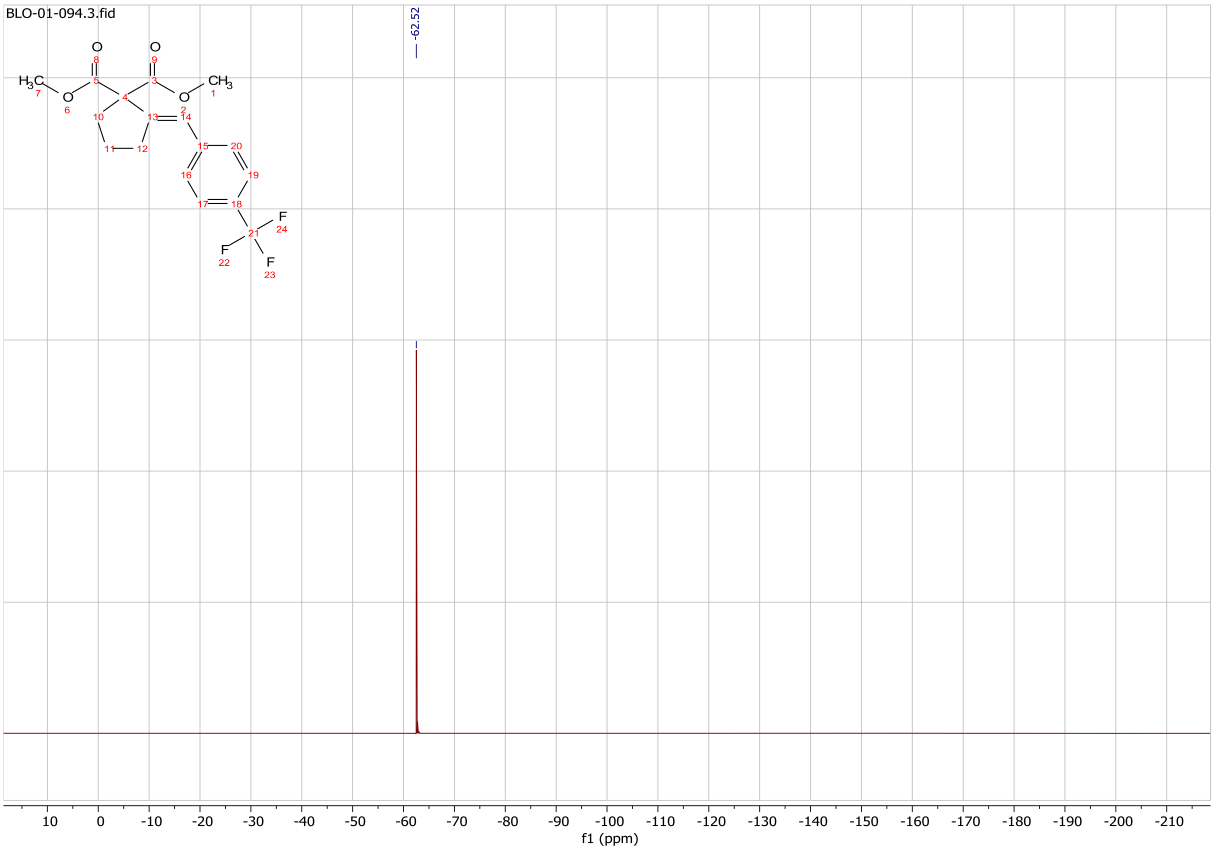
BLO-01-094.3.fid
-62.52
f1 (ppm)

Dimethyl (2*E*)-2-(4-acetylbenzylidene)cyclopentane-1,1-dicarboxylate (7).

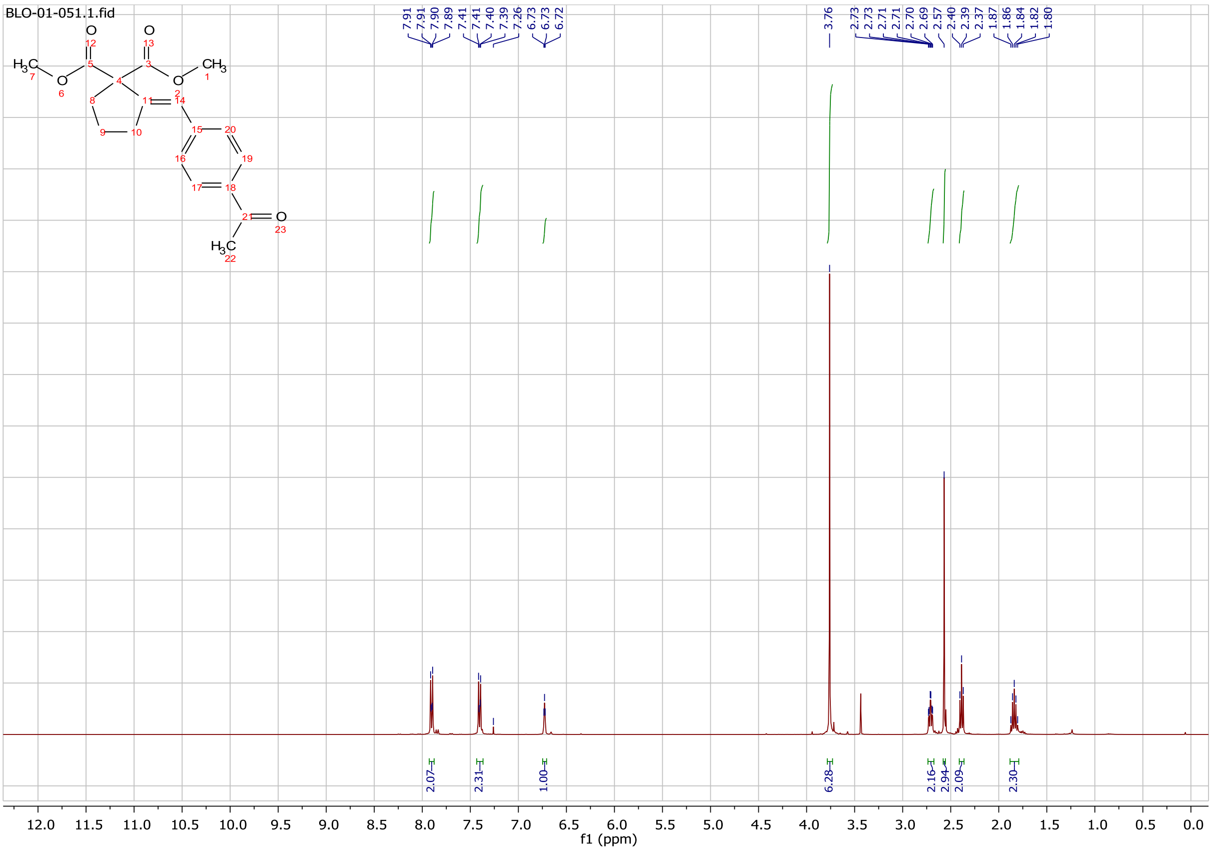

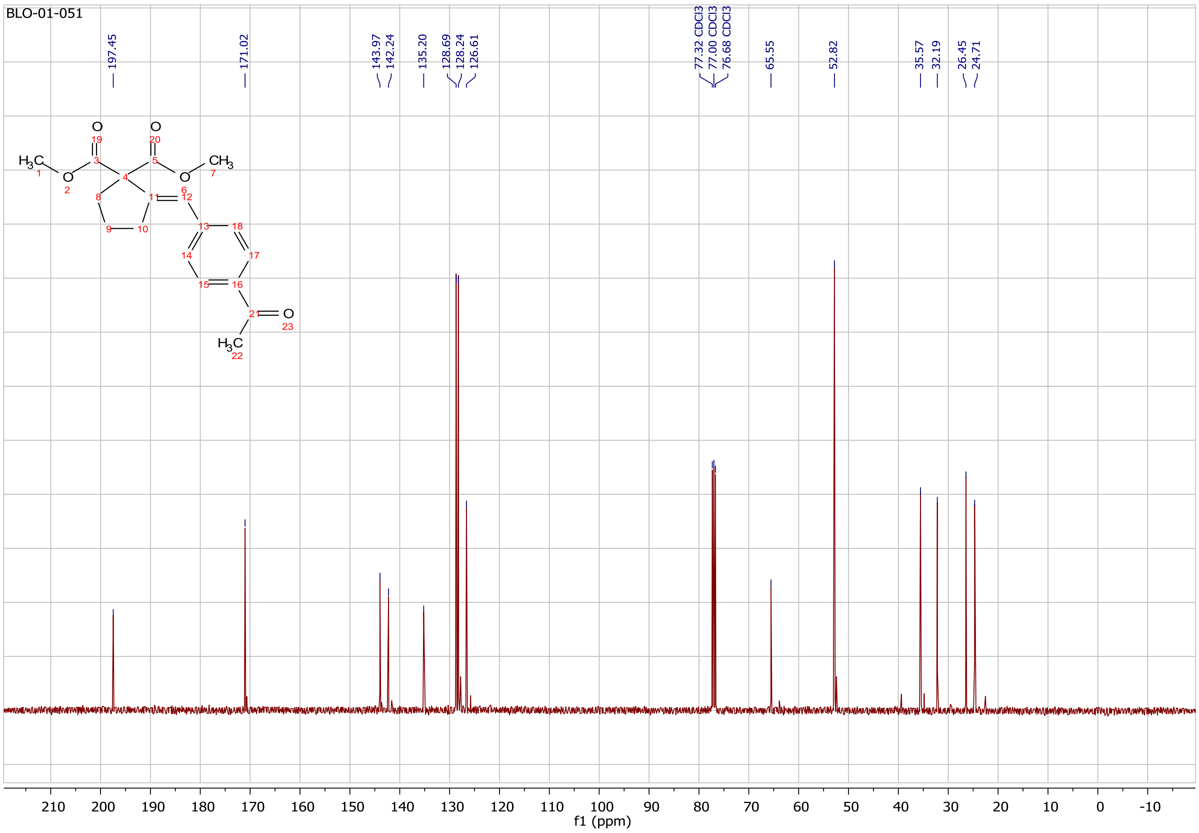

Dimethyl (2*E*)-2-(2-methoxybenzylidene)cyclopentane-1,1-dicarboxylate (8).

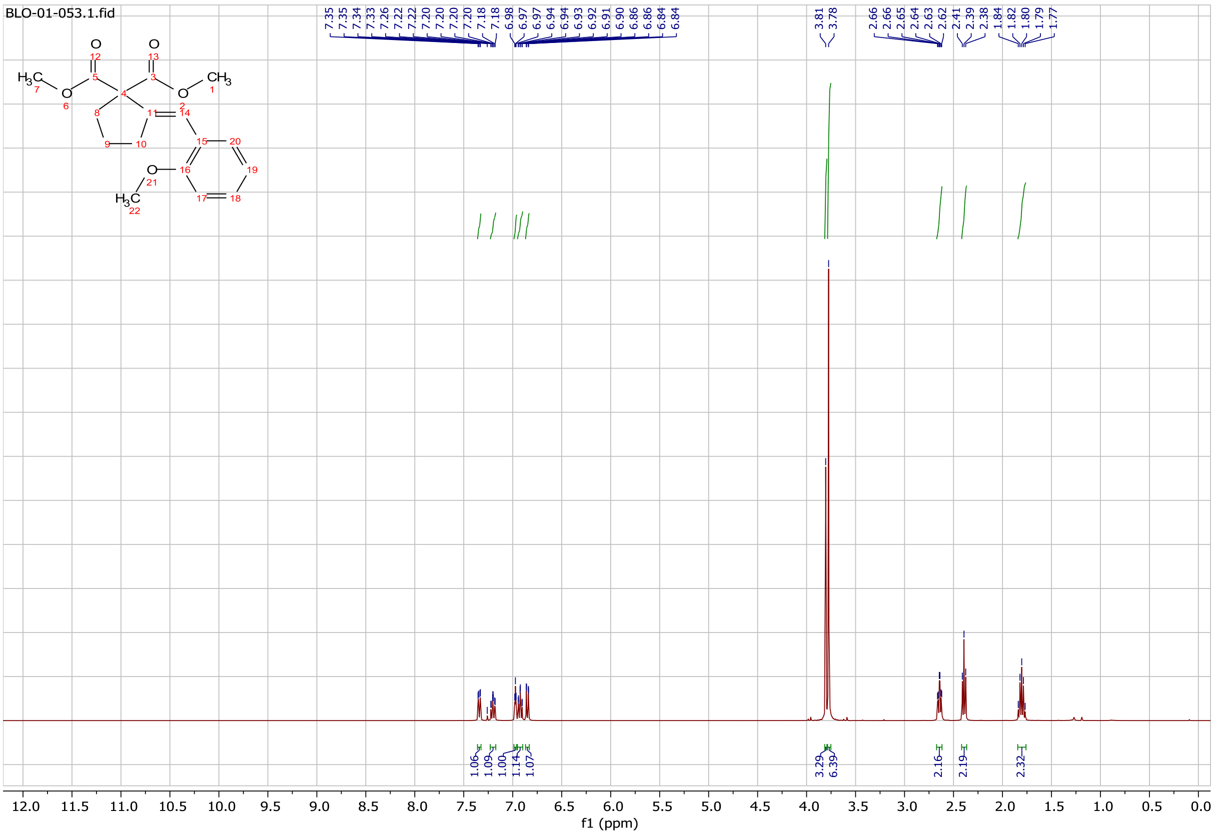

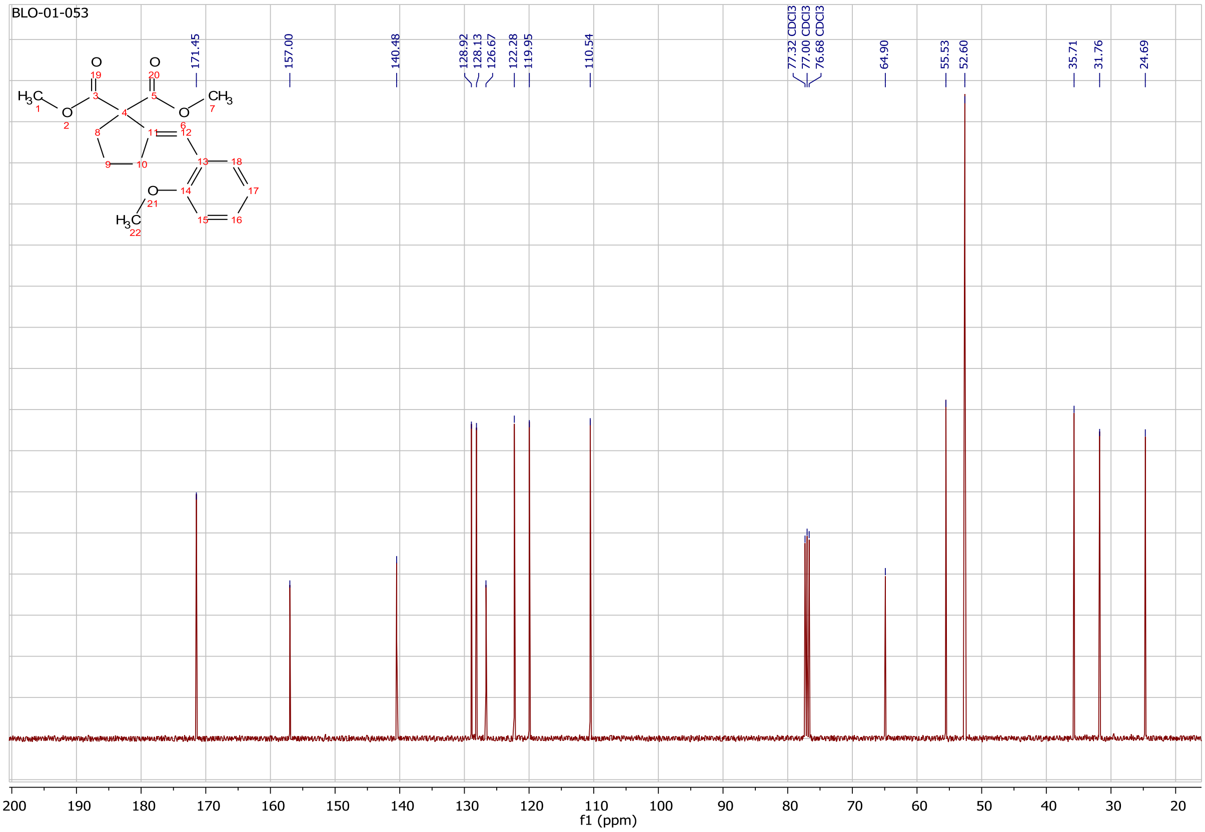

Dimethyl (2*E*)-2-(naphthalen-2-ylmethylidene)cyclopentane-1,1-dicarboxylate (9).

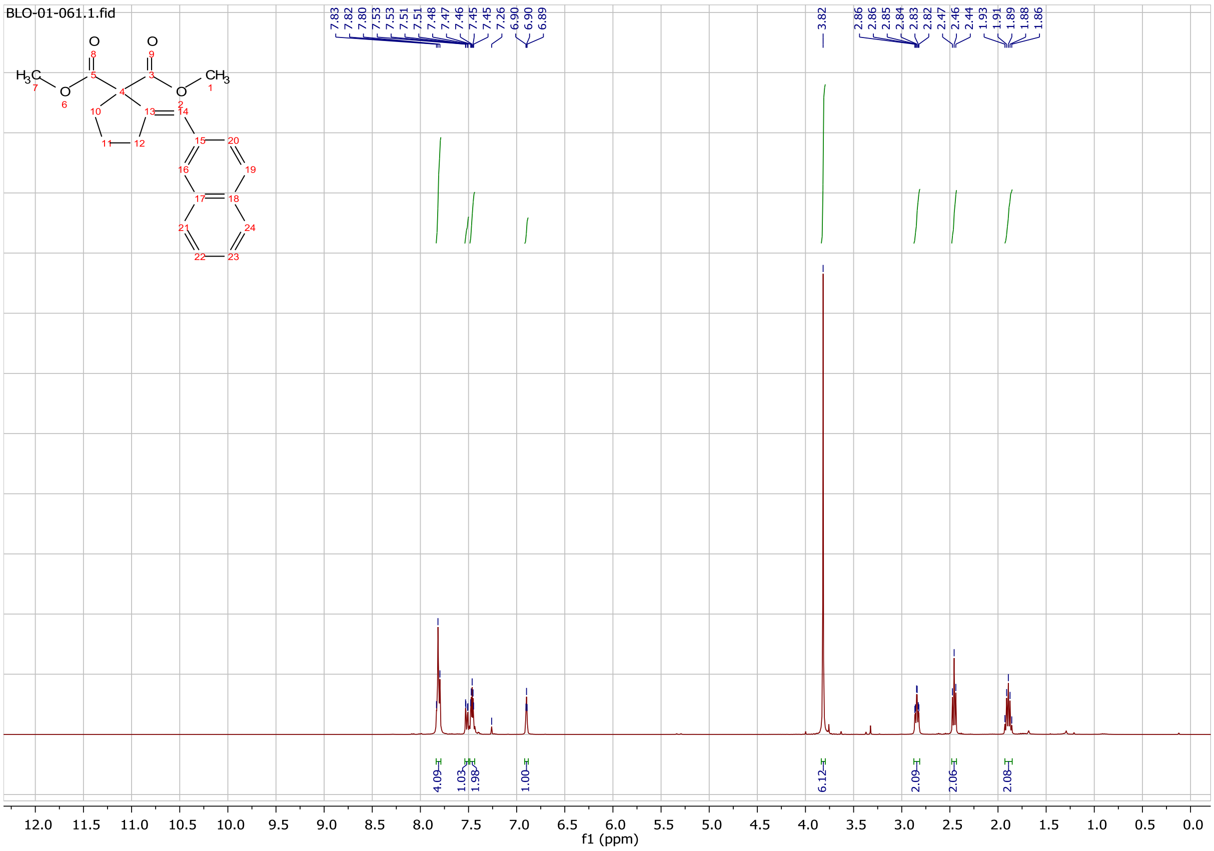

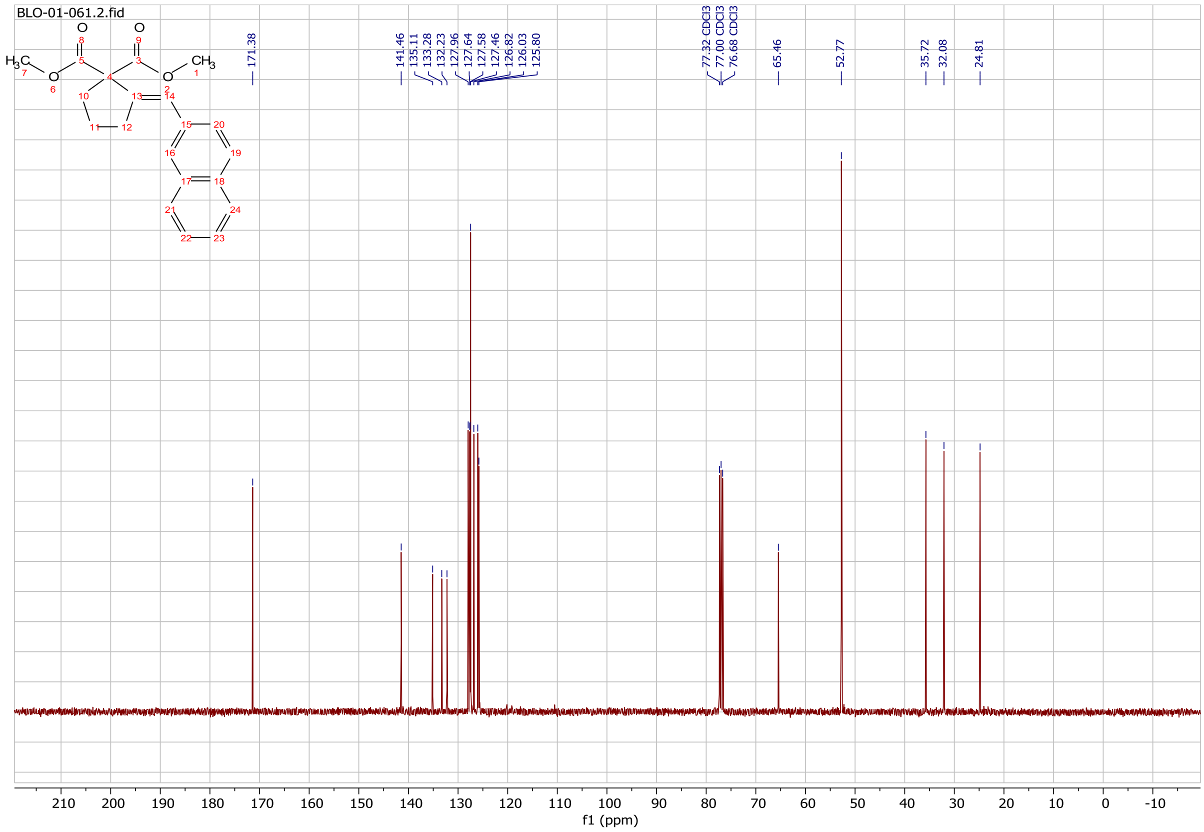

Dimethyl (2*E*)-2-(pyridin-3-ylmethylidene)cyclopentane-1,1-dicarboxylate (10).

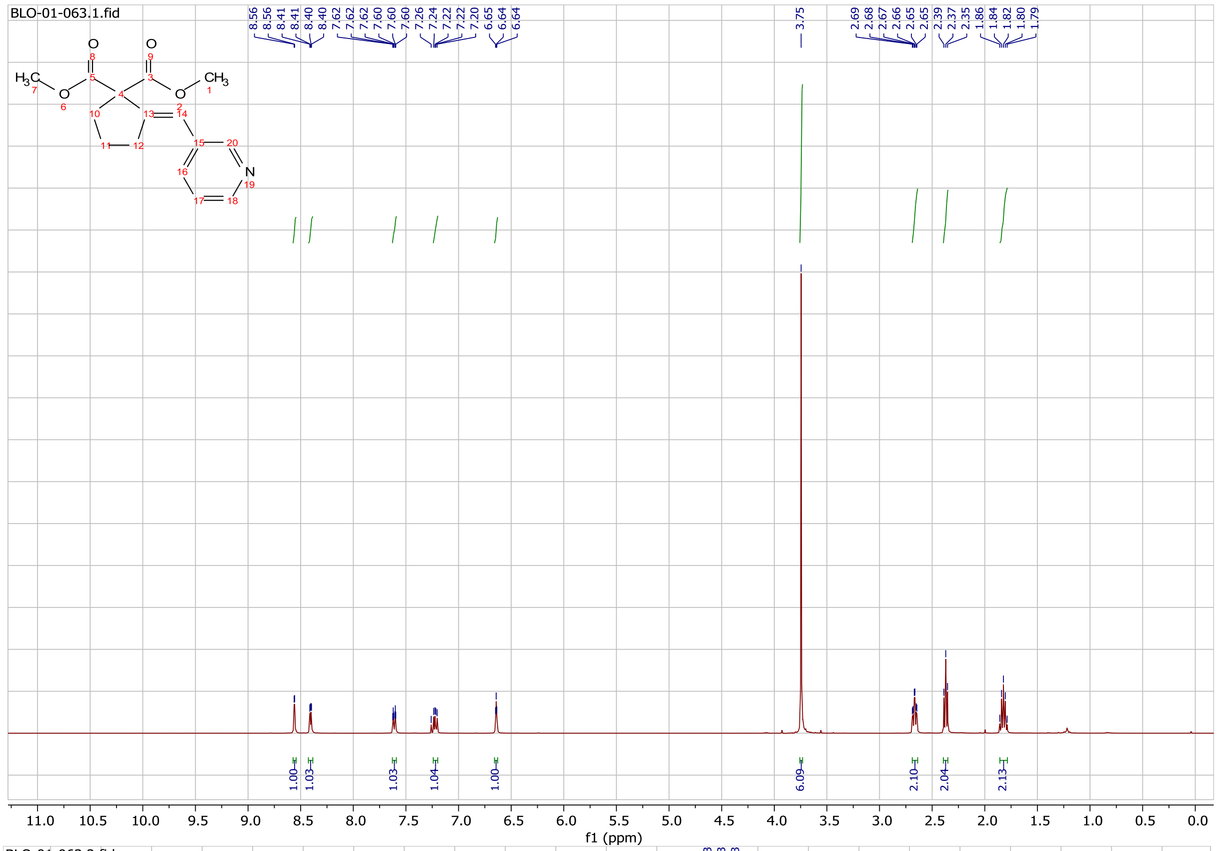

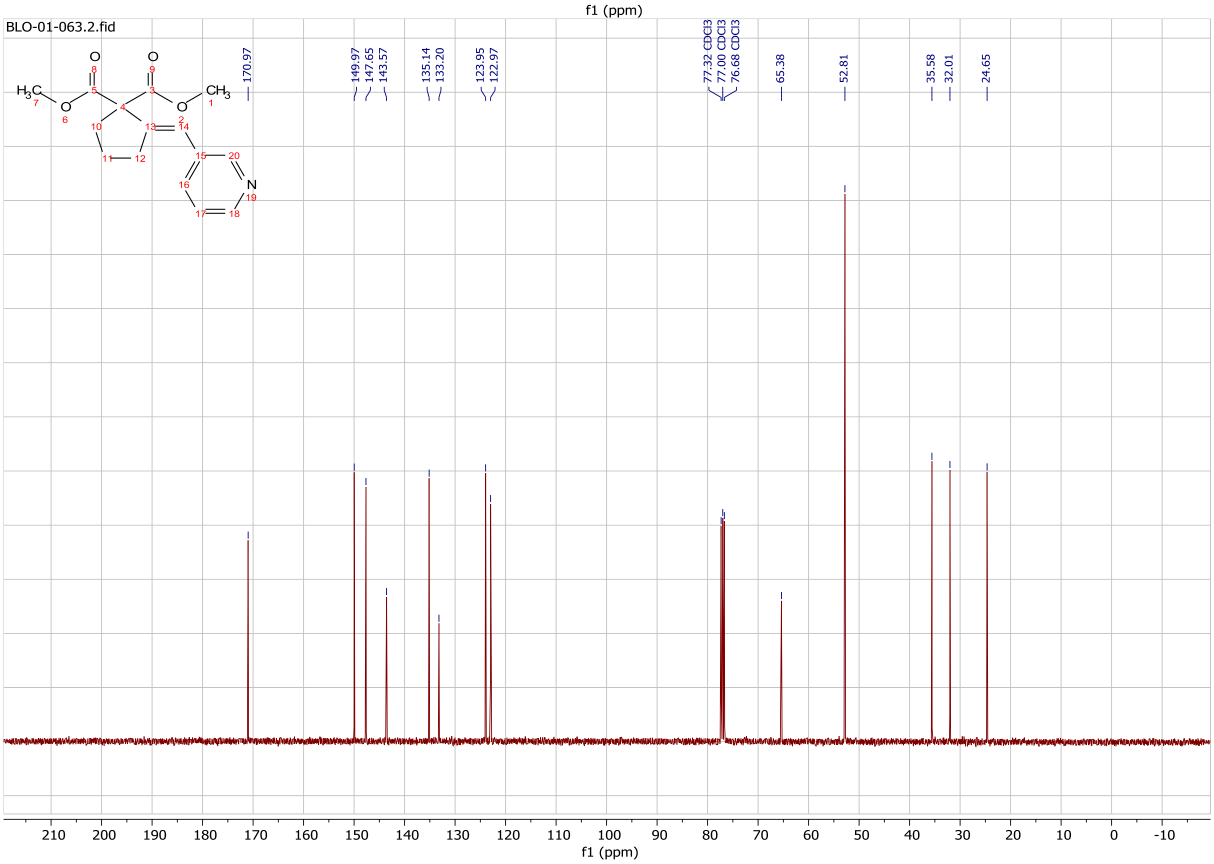

Dimethyl (2*E*)-2-(pyridin-2-ylmethylidene)cyclopentane-1,1-dicarboxylate (11).

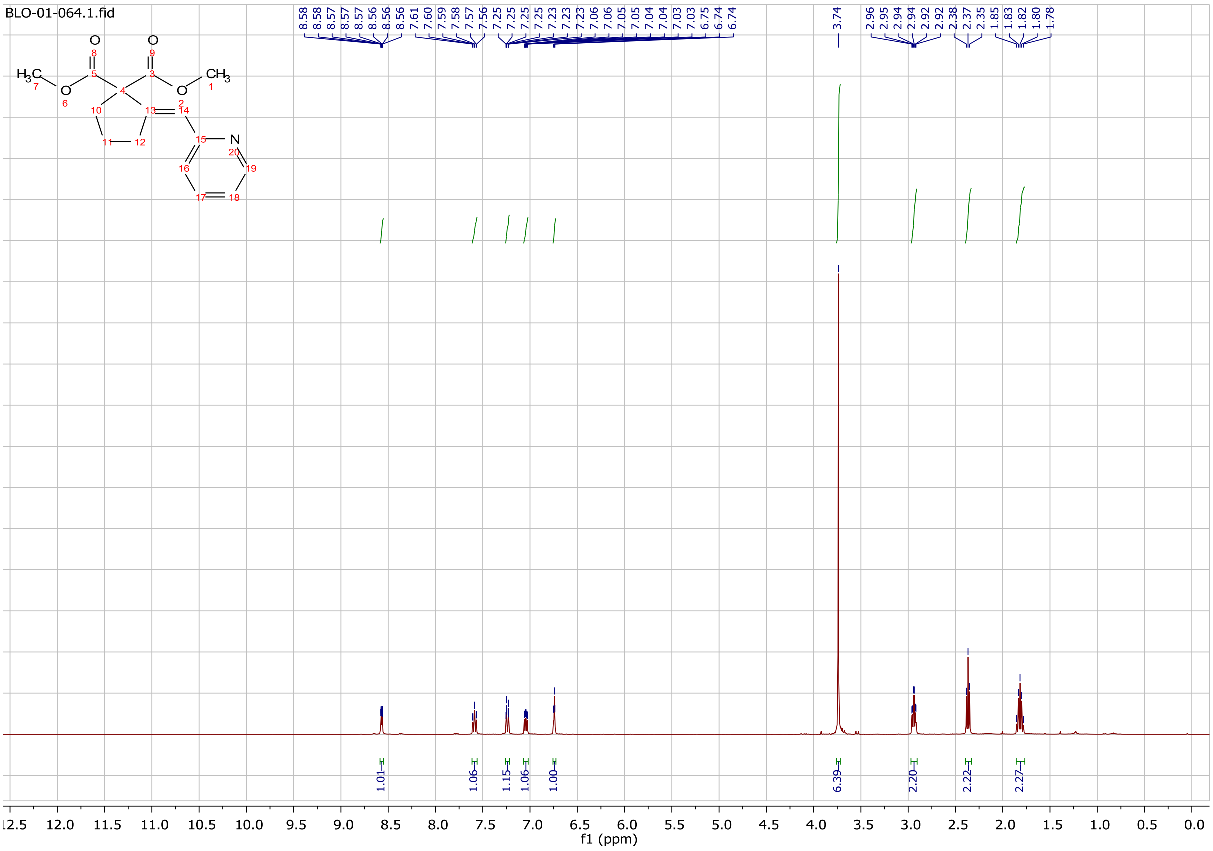

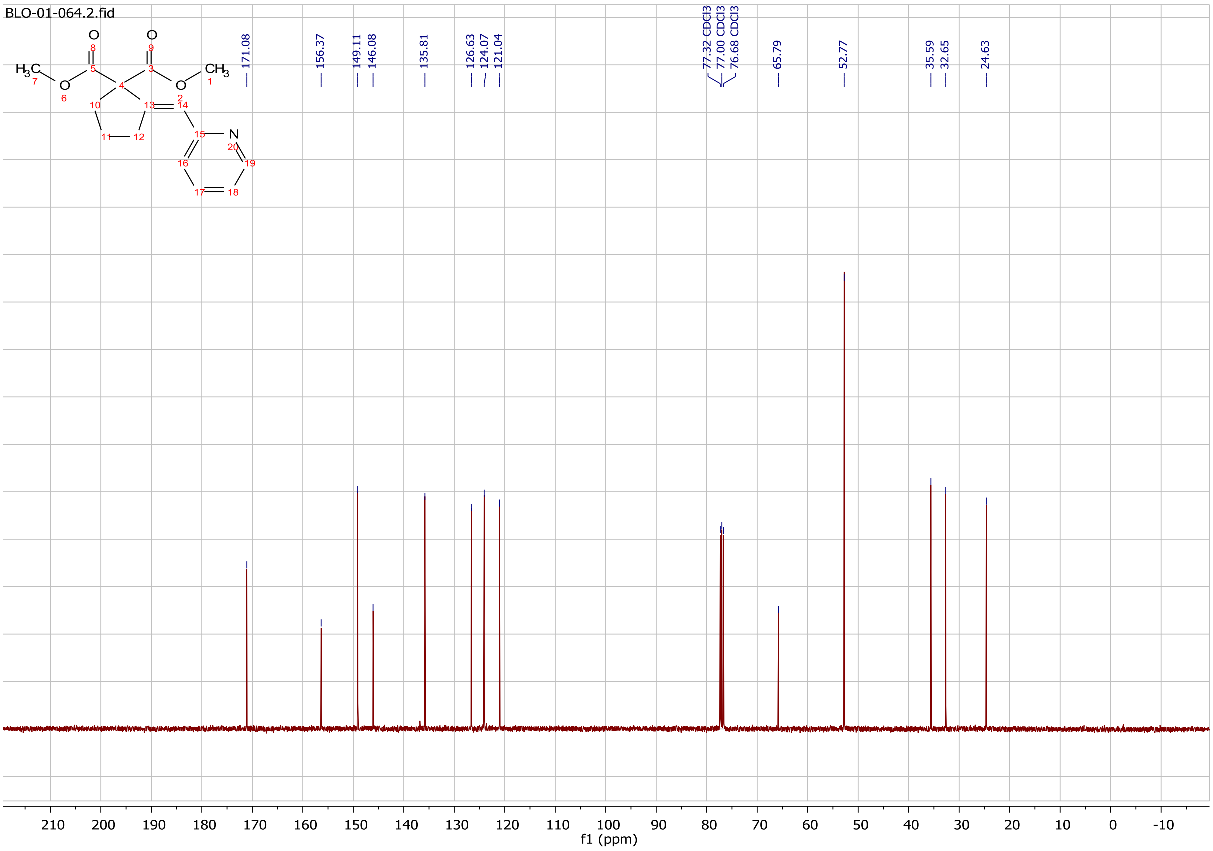

Dimethyl (2*E*)-2-(4-(hydroxymethyl)benzylidene)cyclopentane-1,1-dicarboxylate (12).

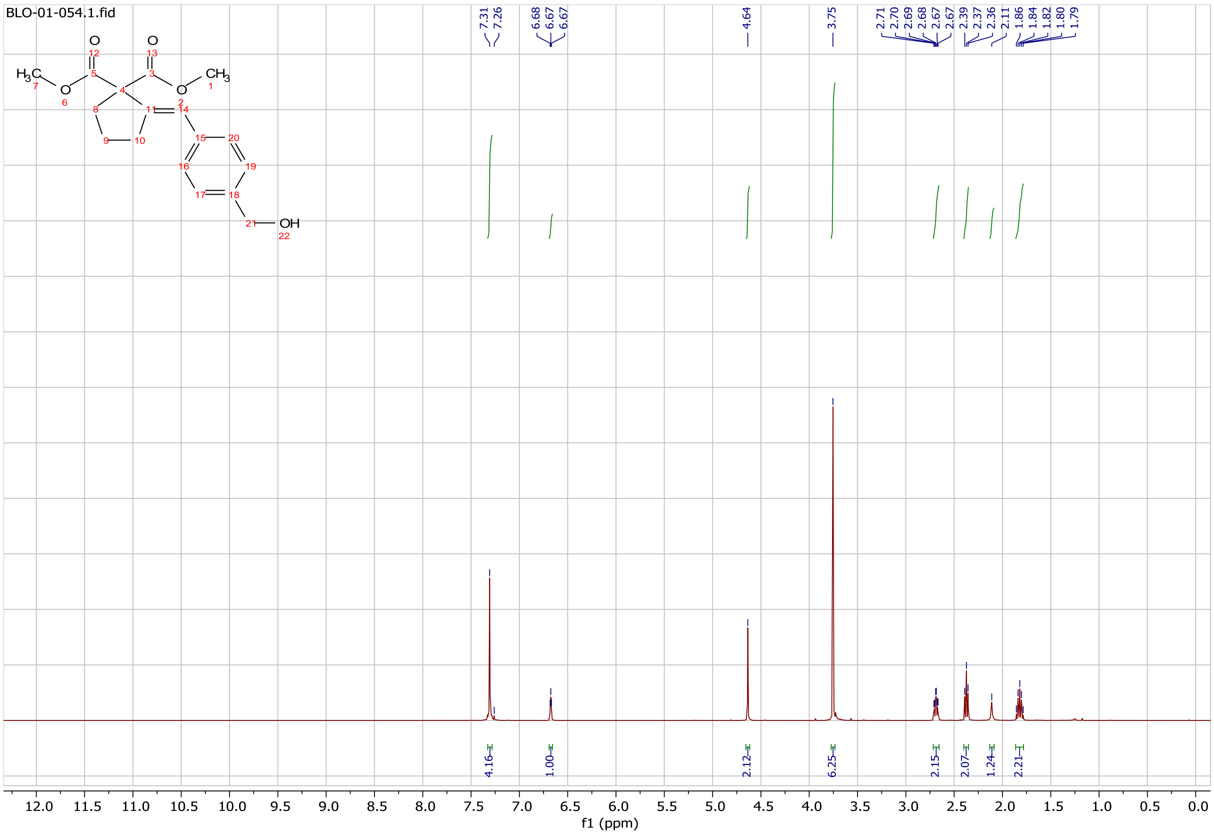

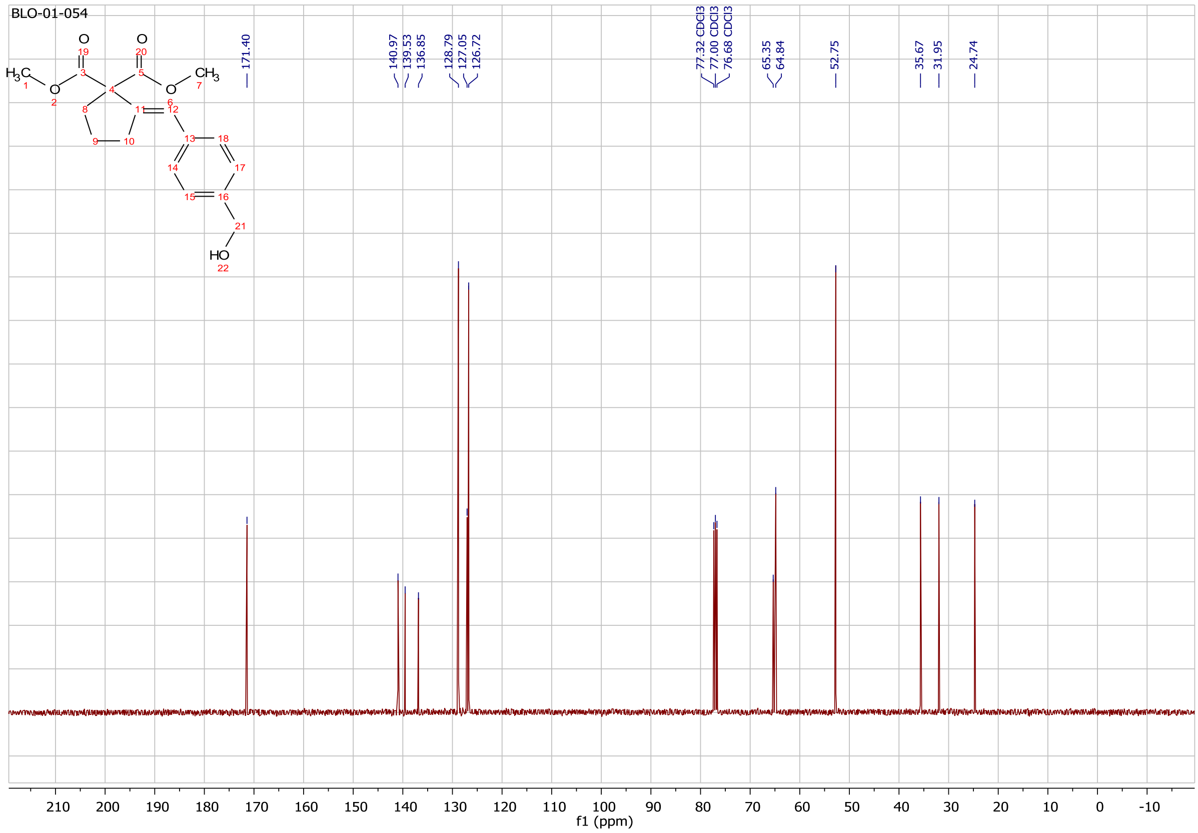

Dimethyl(2*E*)-2-(4-chlorobenzylidene)cyclopentane-1,1-dicarboxylate (13).

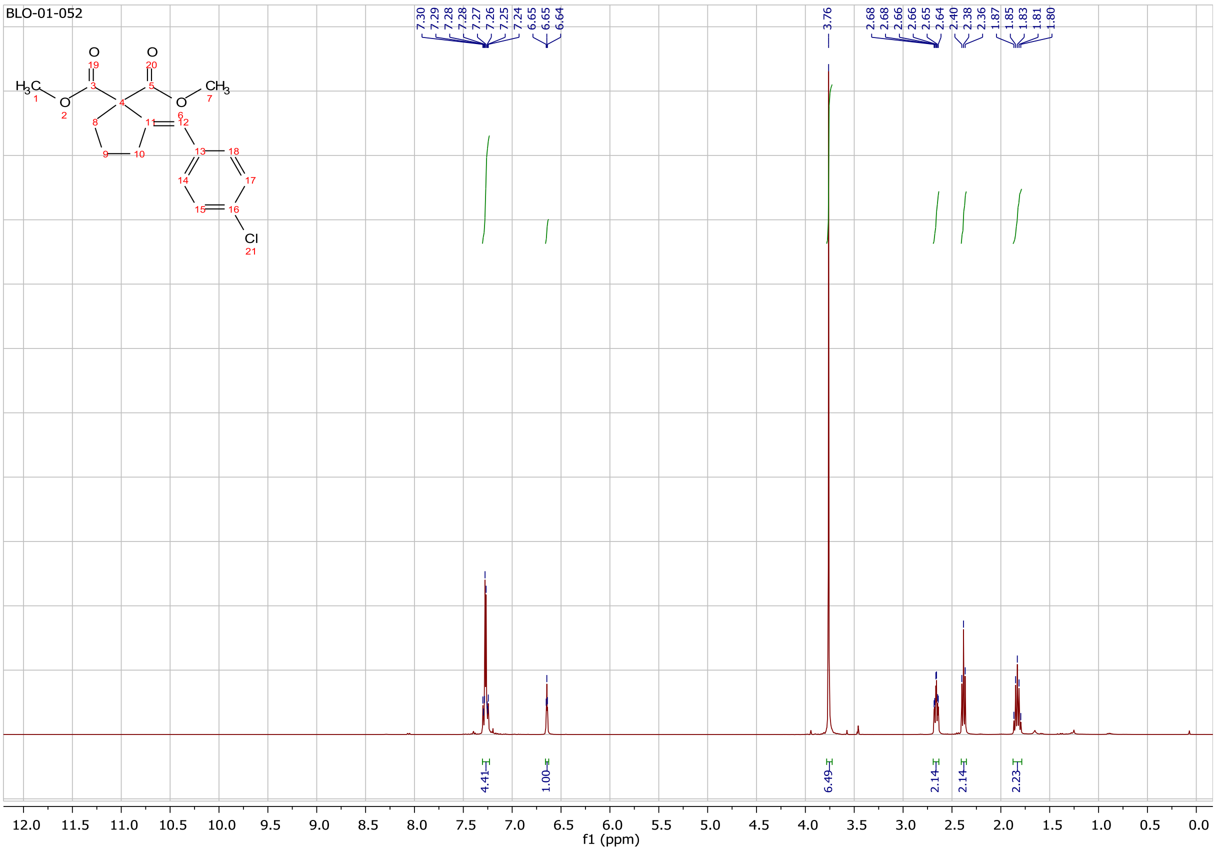

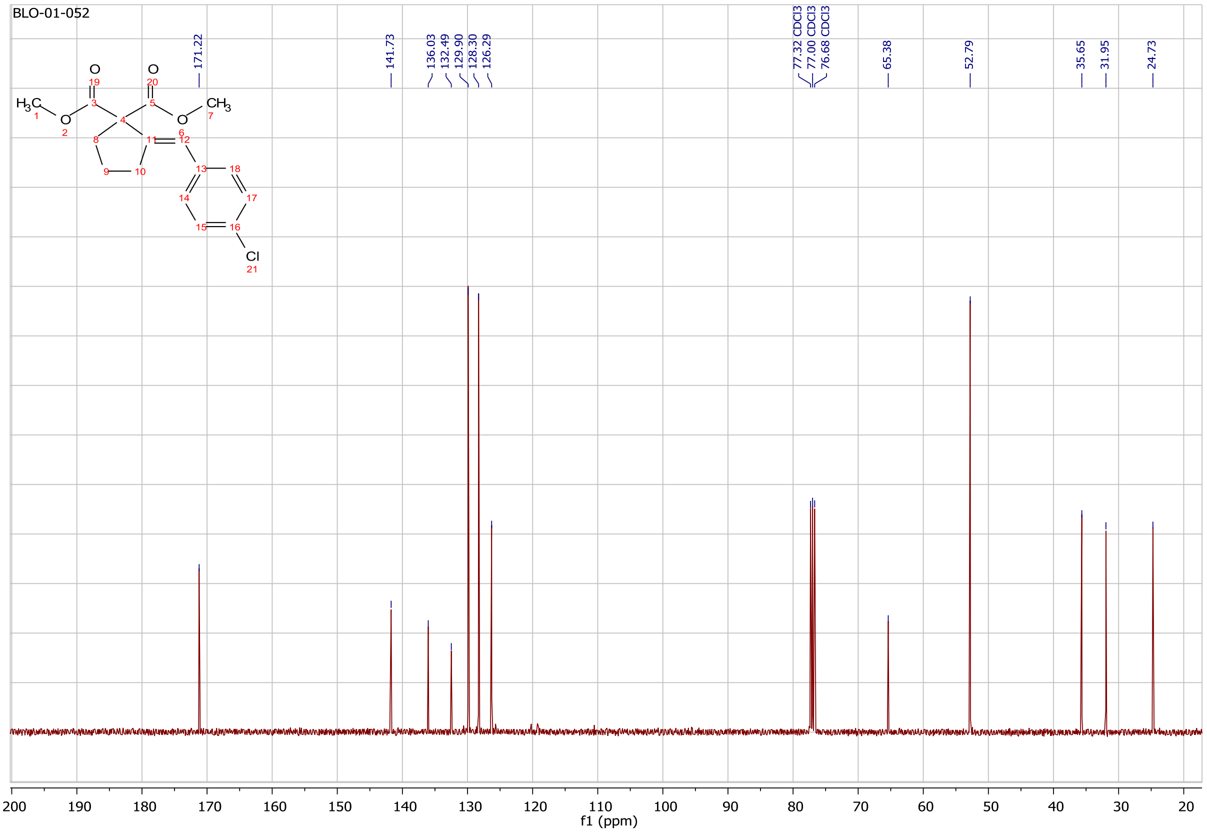

Dimethyl (2*E*)-2-(isoquinolin-5-ylmethylidene)cyclopentane-1,1-dicarboxylate (14).

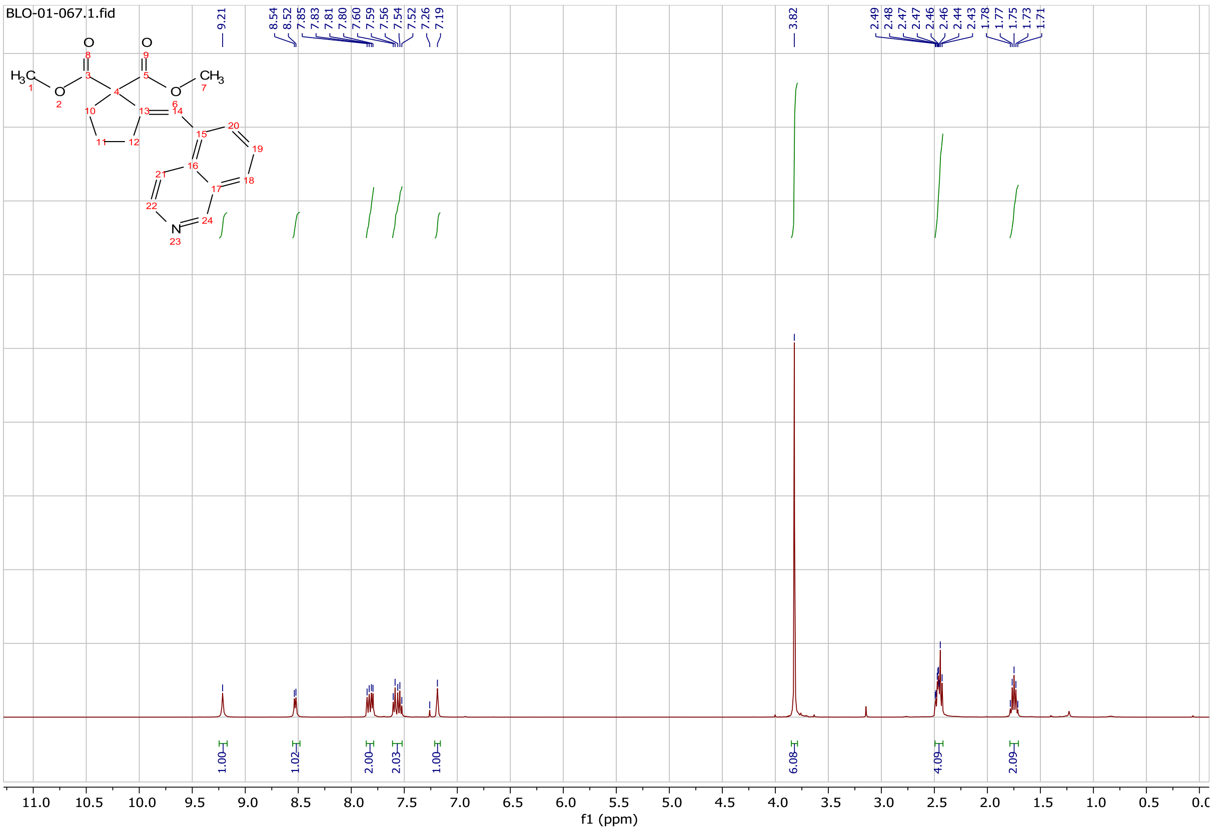

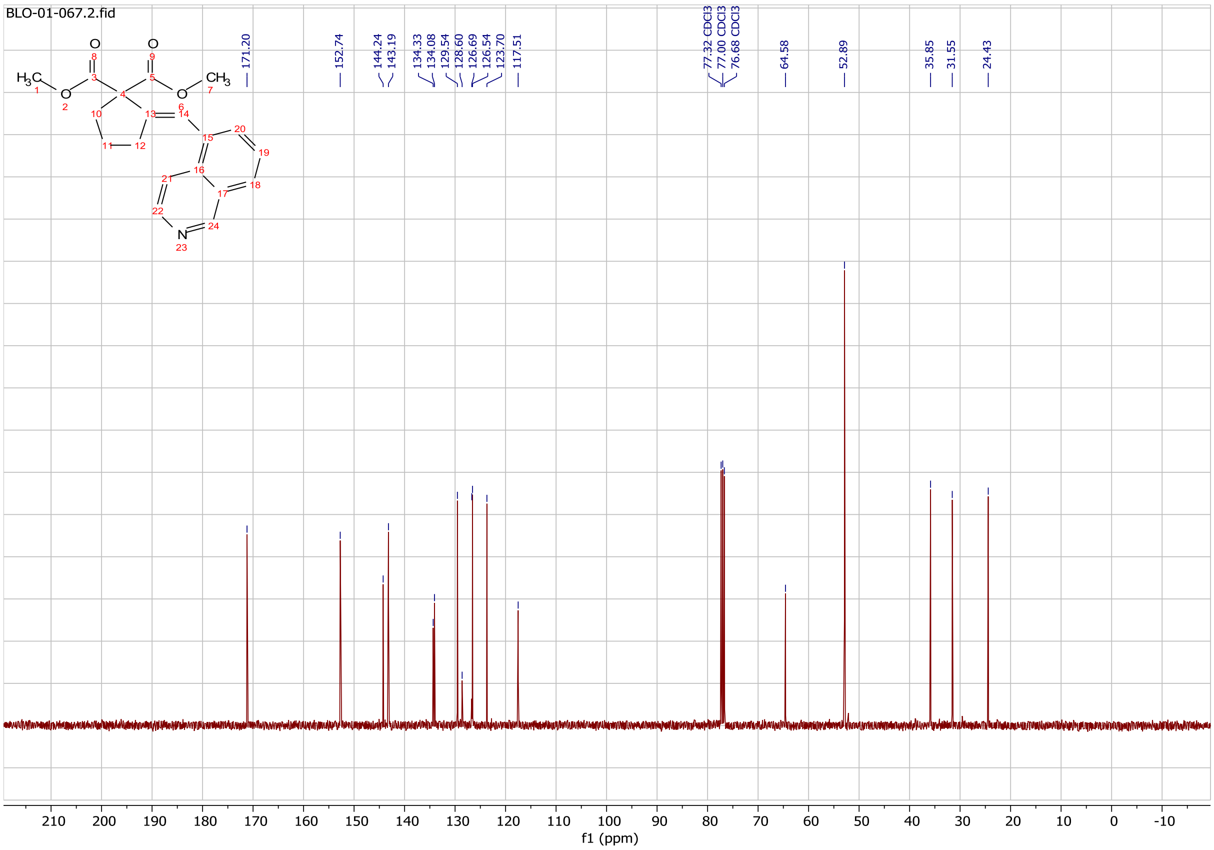

Dimethyl (2*E*)-2-(quinolin-5-ylmethylidene)cyclopentane-1,1-dicarboxylate (15).

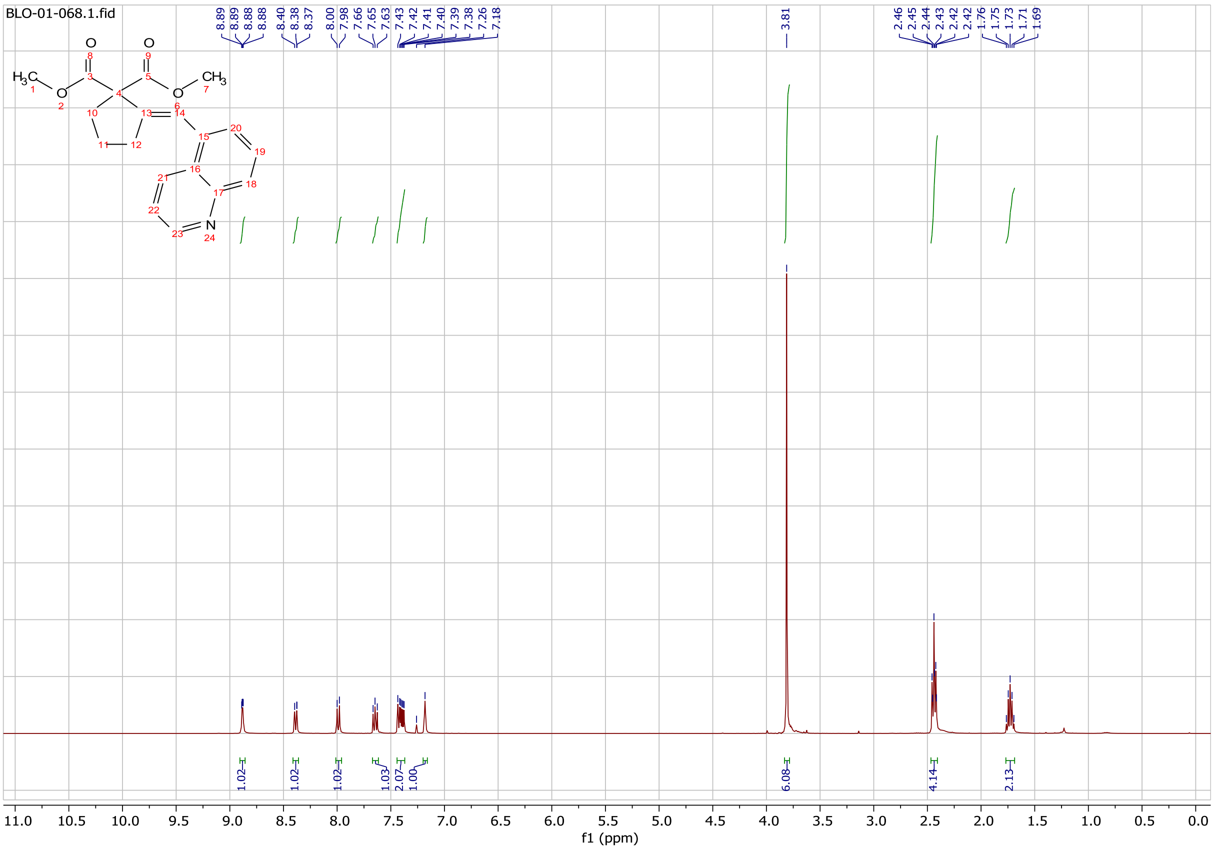

BLO-01-068.1.fid
8.89
8.89
8.88
8.88
8.40
8.38
8.37
8.00
7.98
7.66
7.65
7.63
7.43
7.42
7.41
7.40
7.39
7.38
7.26
7.18
3.81
2.46
2.45
2.44
2.43
2.42
2.42
1.76
1.75
1.73
1.71
1.69
1.02
1.02
1.02
1.03
2.07
1.00
6.08
4.14
2.13
f1 (ppm)

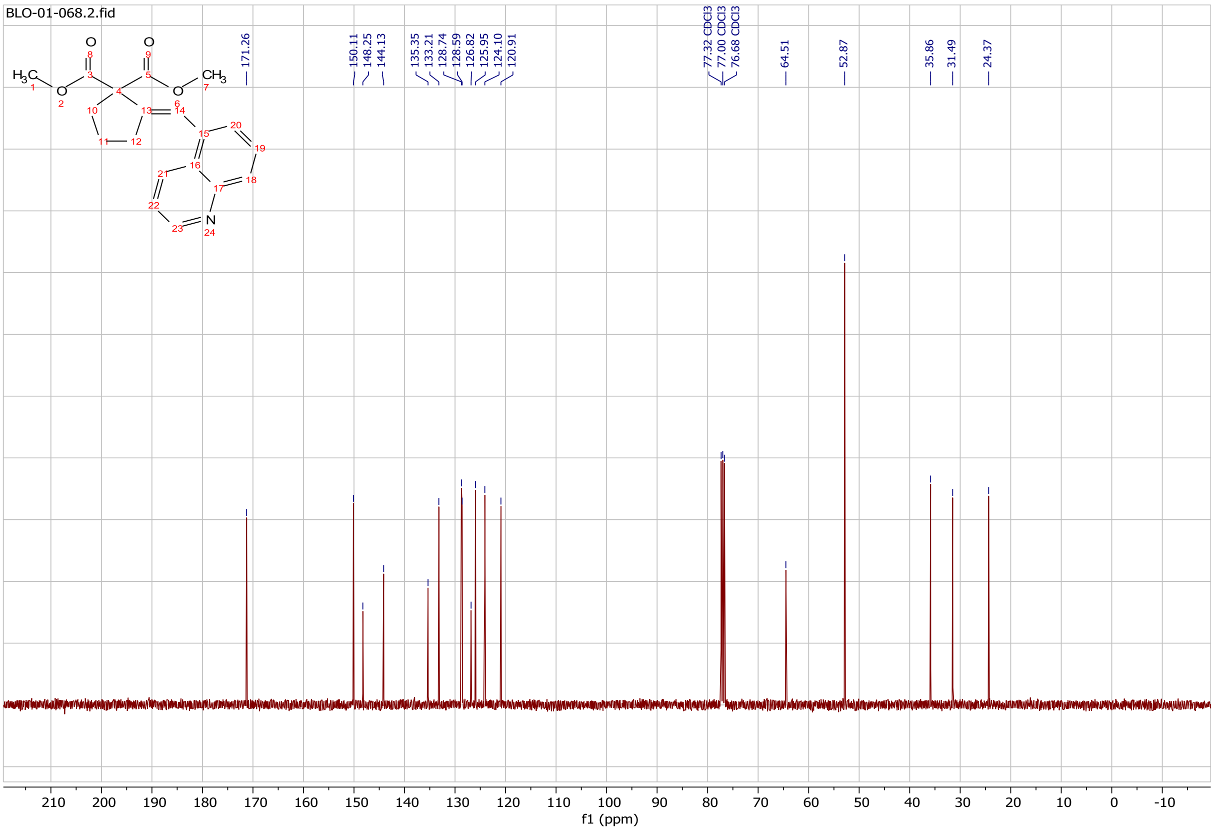

BLO-01-068.2.fid
171.26
150.11
148.25
144.13
135.35
133.21
128.74
128.59
126.82
125.95
124.10
120.91
77.32 CDCl3
77.00 CDCl3
76.68 CDCl3
64.51
52.87
35.86
31.49
24.37
f1 (ppm)

Dimethyl (2*E*)-2-(1,3-benzodioxol-5-ylmethylidene)cyclopentane-1,1-dicarboxylate (16).

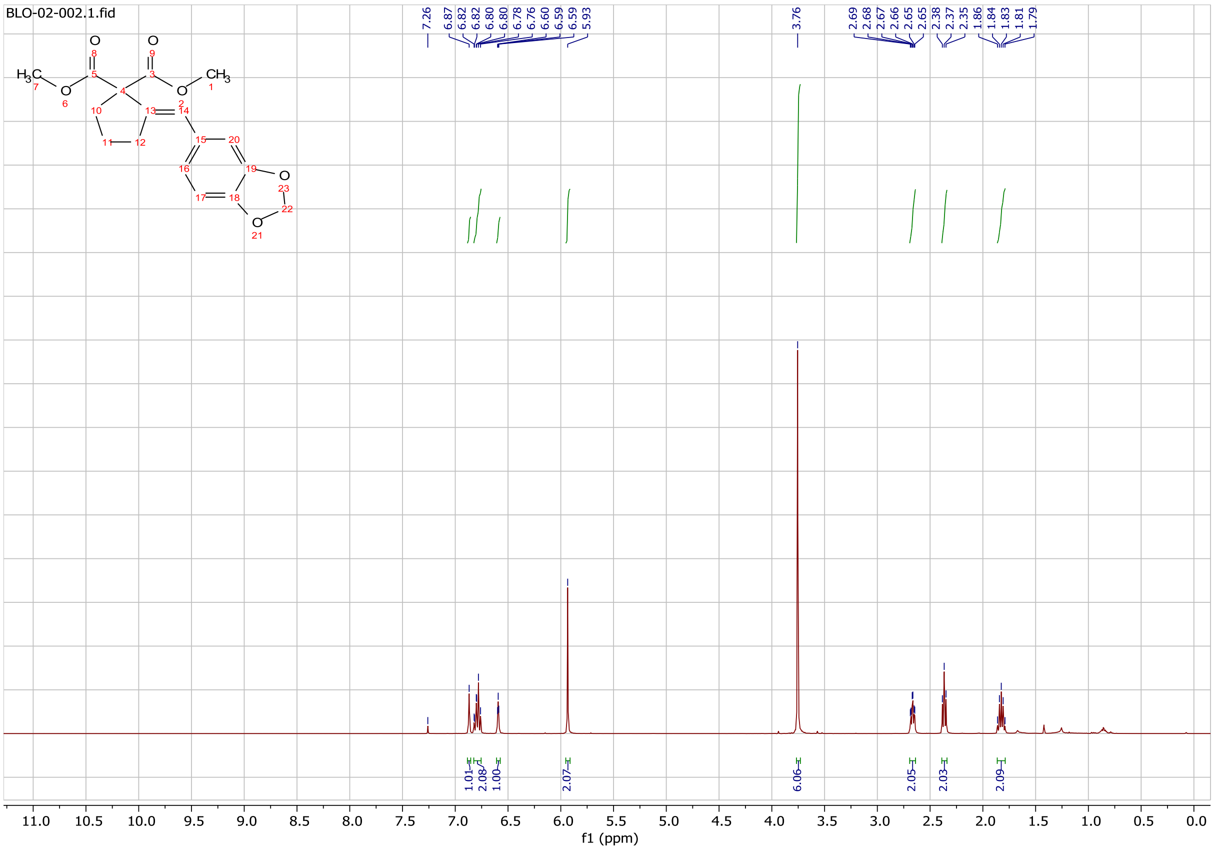

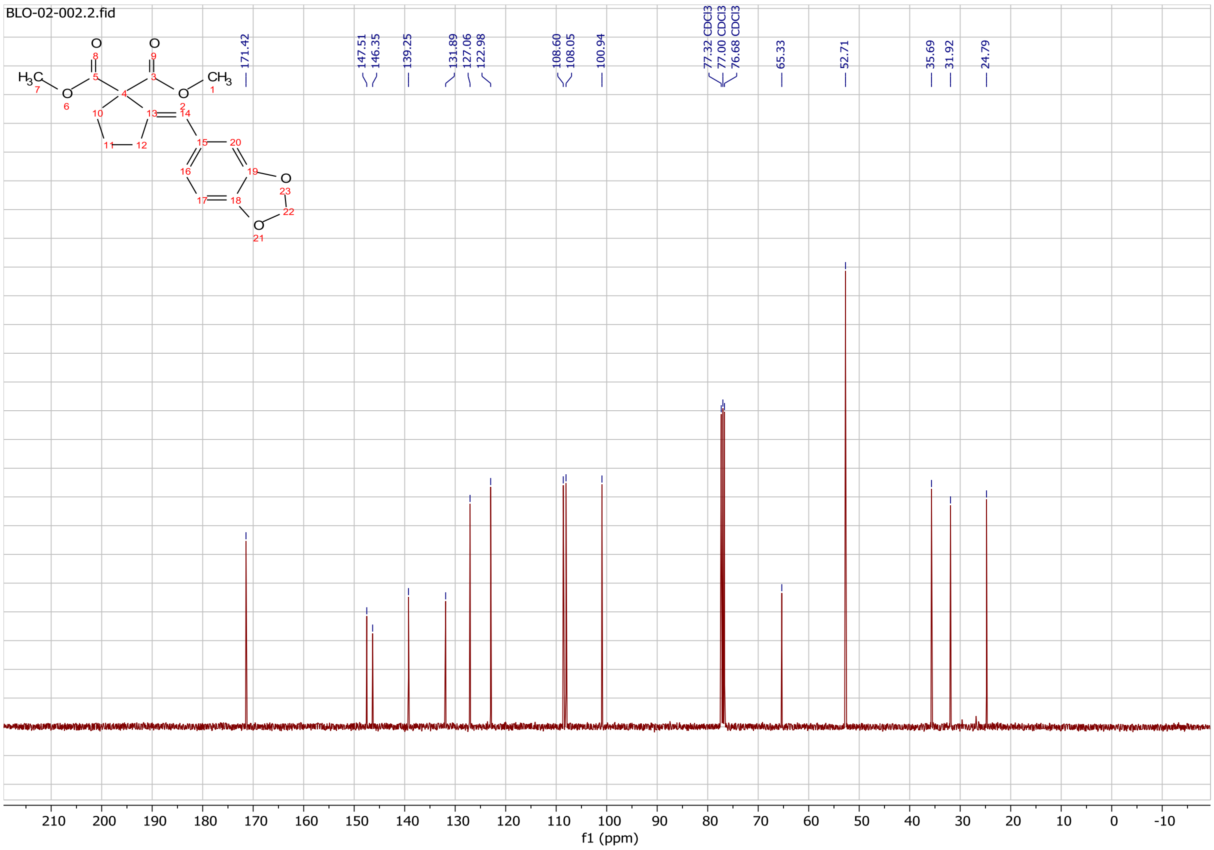

Dimethyl (2*E*)-2-(2-(methoxycarbonyl)benzylidene)cyclopentane-1,1-dicarboxylate (17).

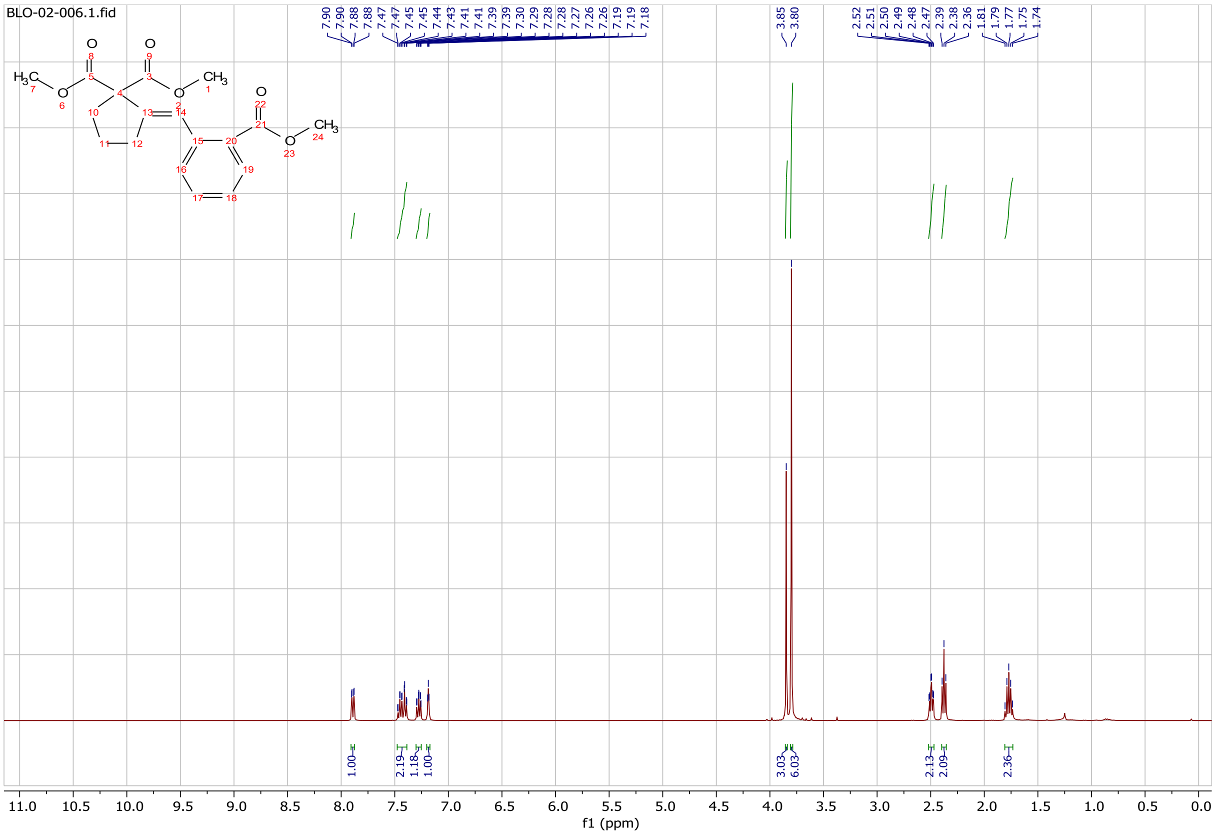

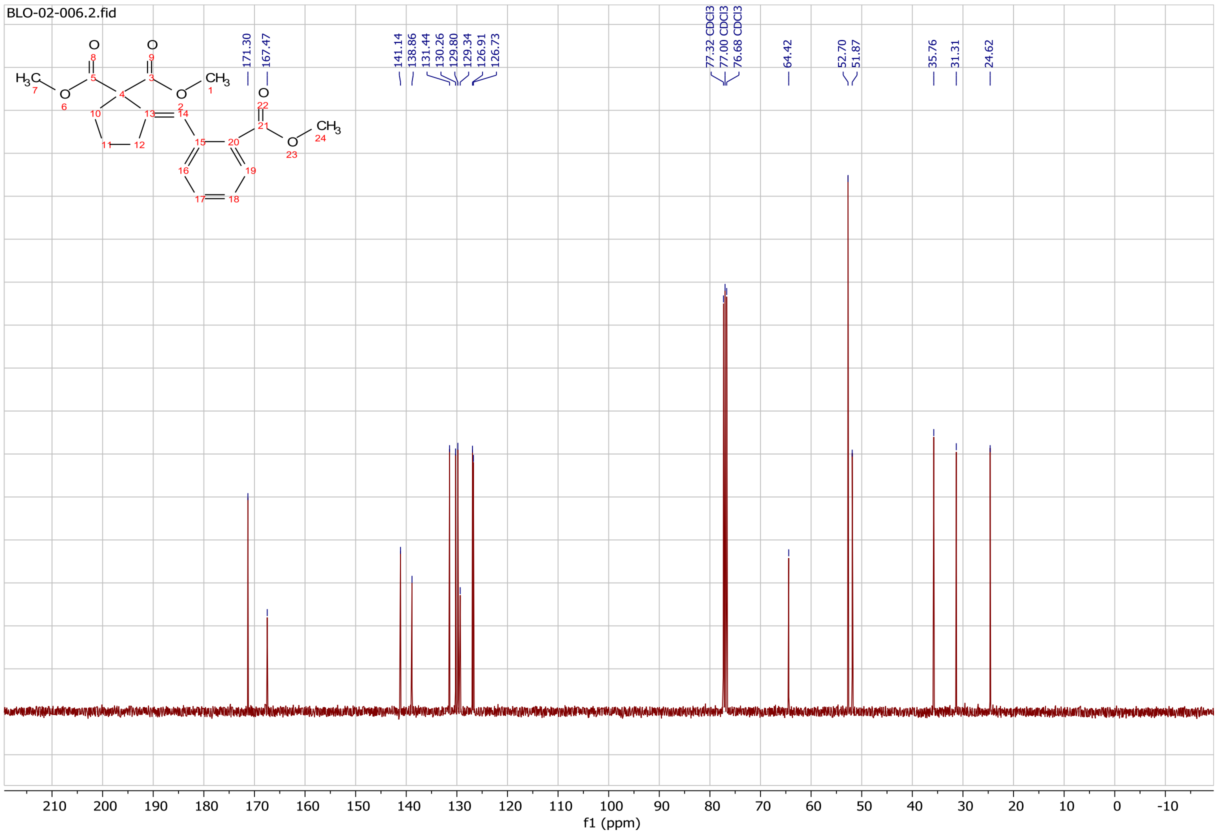

Dimethyl (2*E*)-2-(1,3-benzothiazol-5-ylmethylidene)cyclopentane-1,1-dicarboxylate (18).

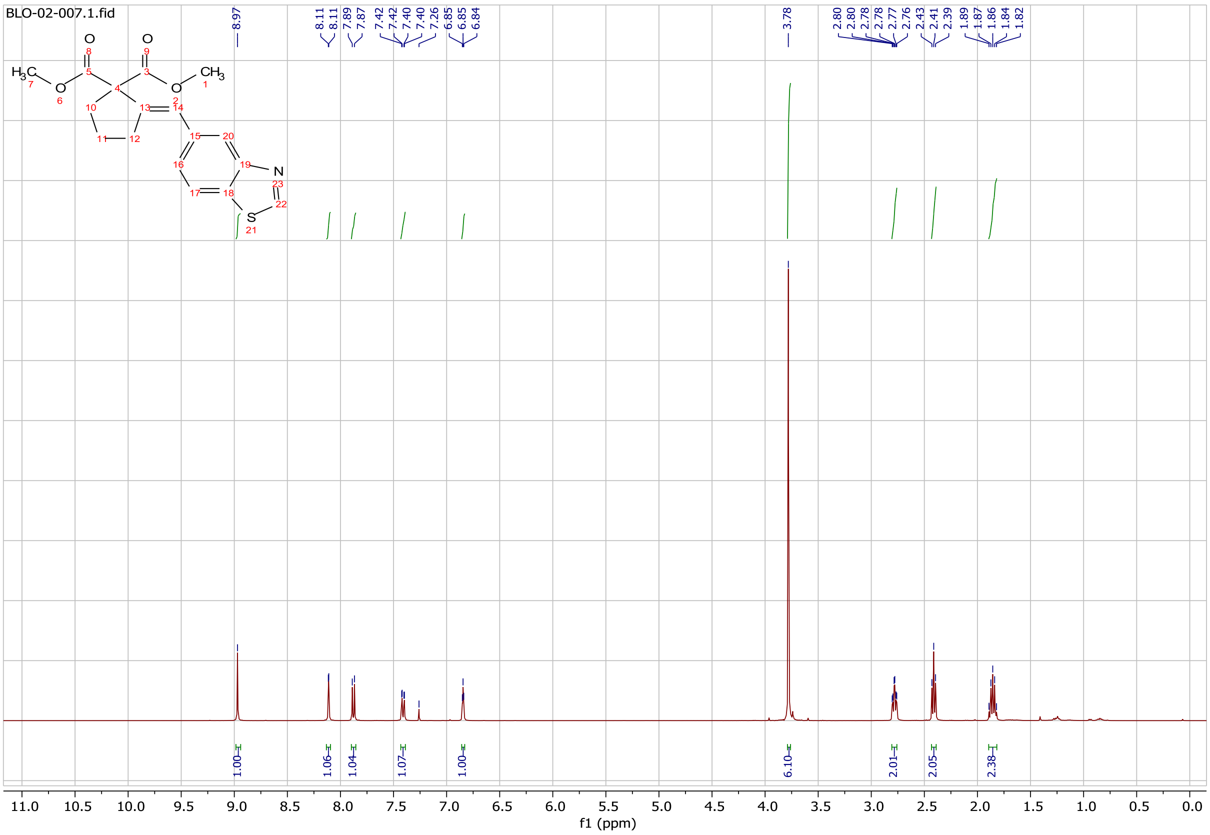

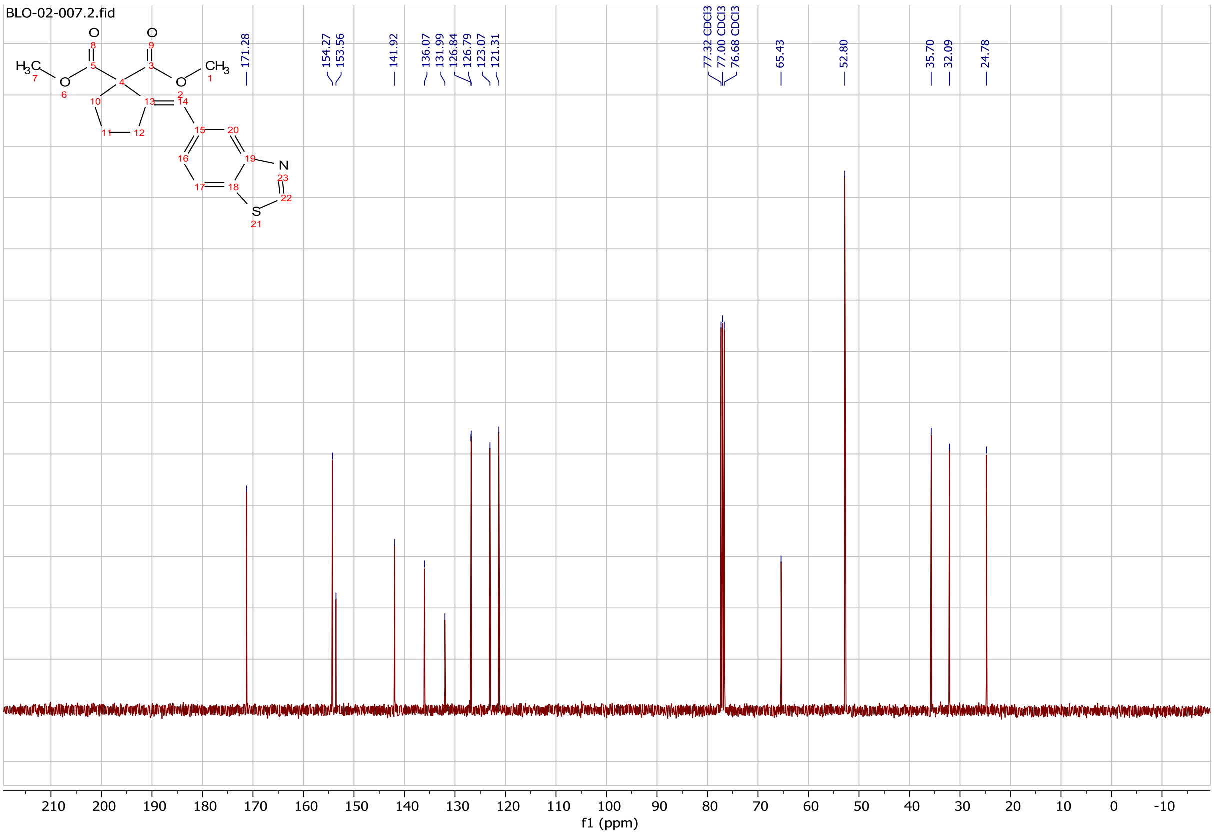

Dimethyl (2*E*)-2-((2-methyl-1,3-benzoxazol-5-yl)methylidene)cyclopentane-1,1-dicarboxylate (19).

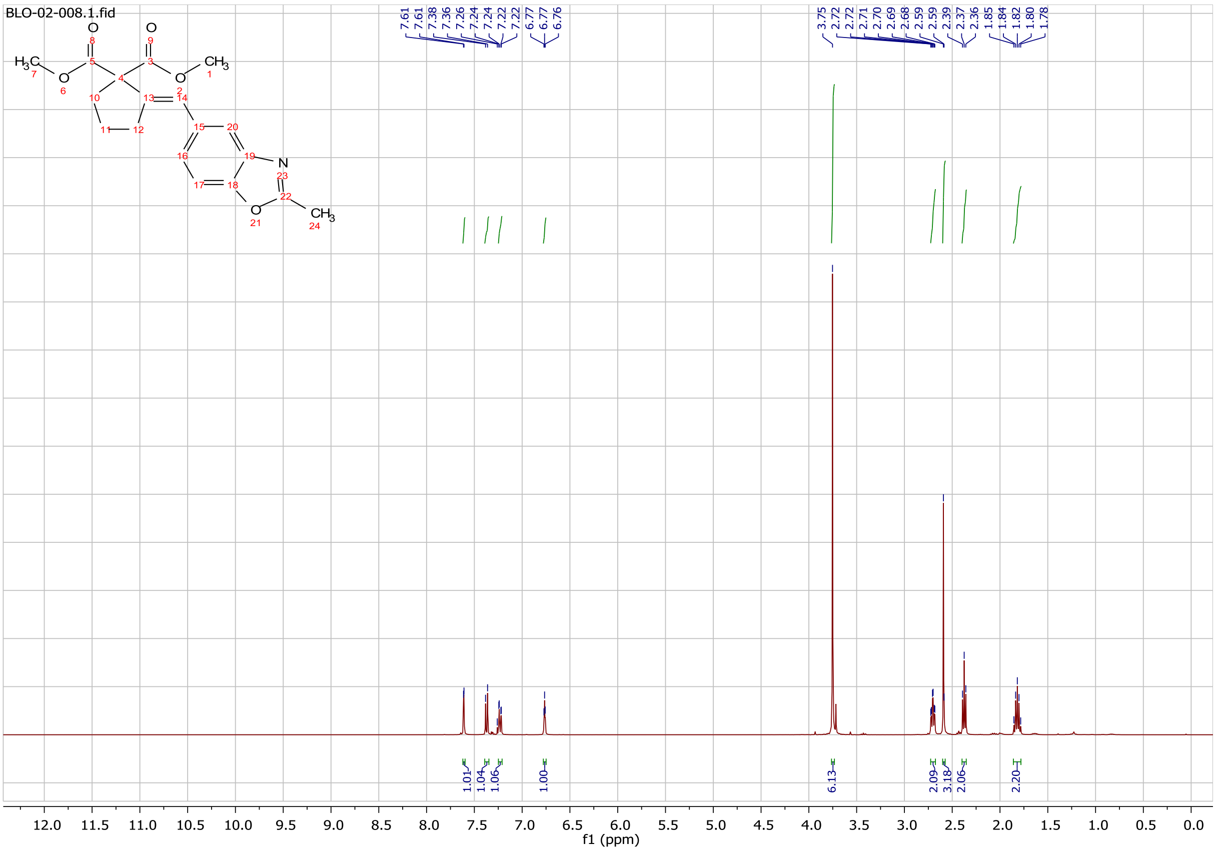

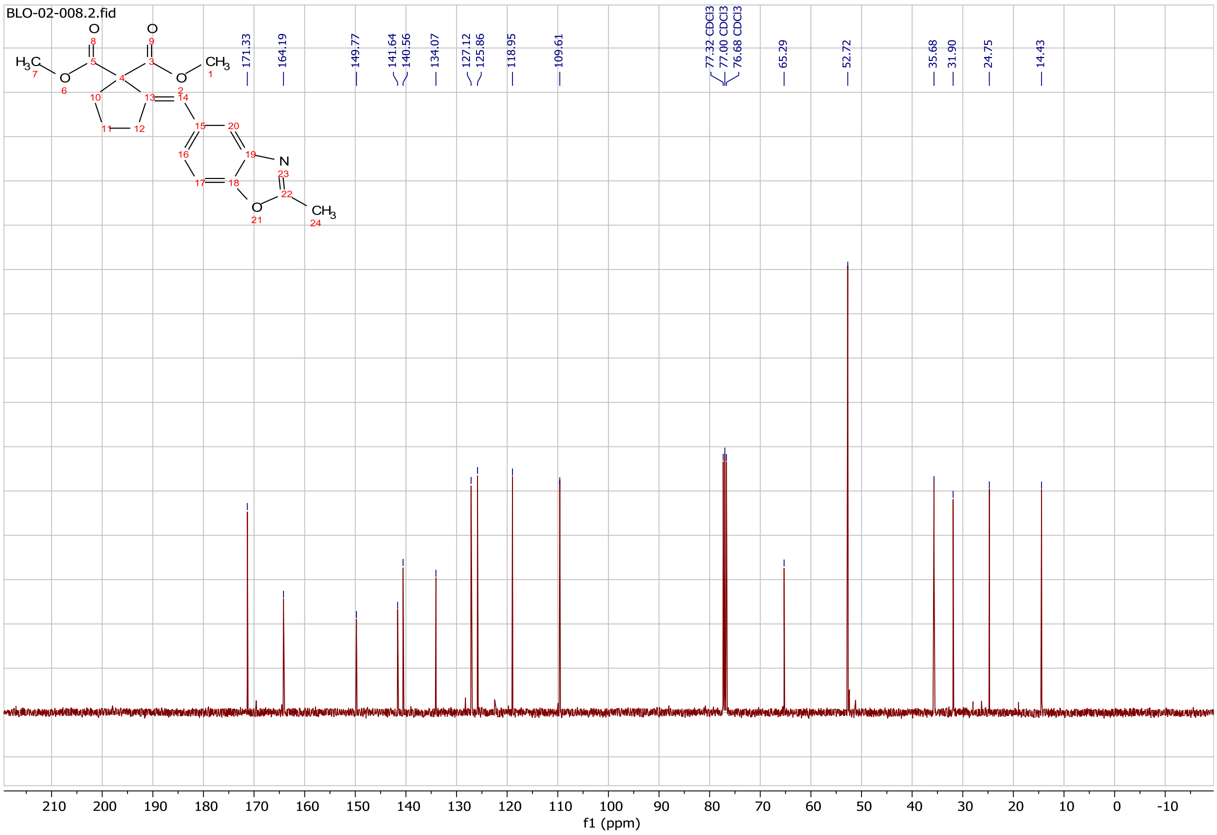

Dimethyl (2*E*)-2-(4-cyanobenzylidene)cyclopentane-1,1-dicarboxylate (20).

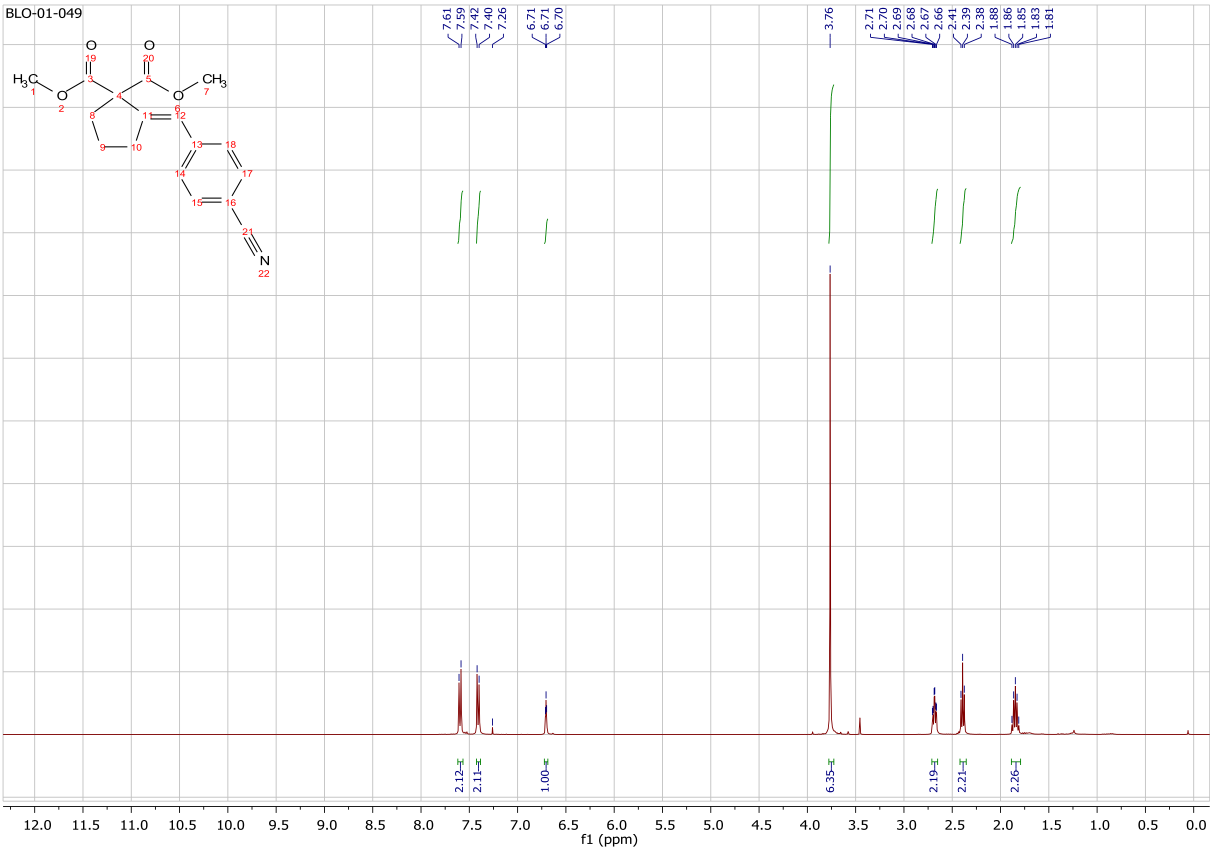
BLO-01-049
7.61
7.59
7.42
7.40
7.26
6.71
6.71
6.70
3.76
2.71
2.70
2.69
2.68
2.67
2.66
2.41
2.39
2.38
1.88
1.86
1.85
1.83
1.81
2.12
2.11
1.00
6.35
2.19
2.21
2.26
f1 (ppm)

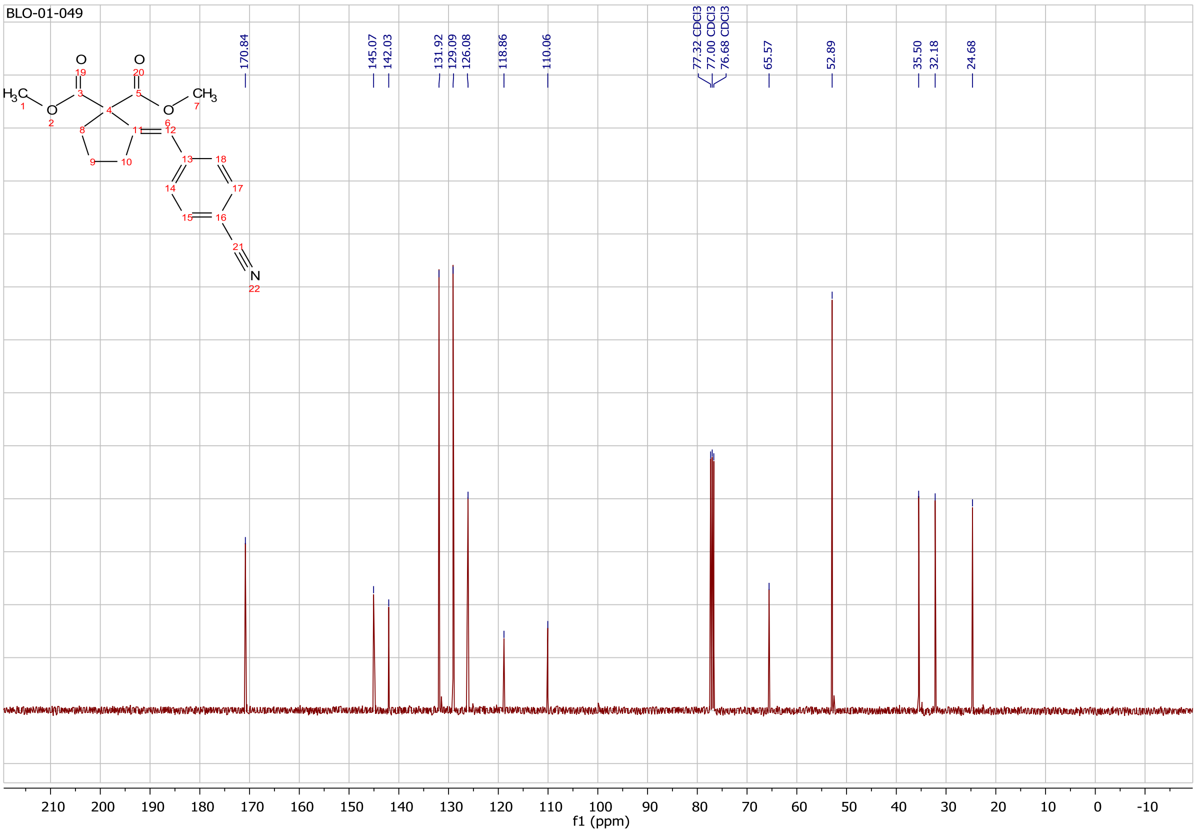
BLO-01-049
170.84
145.07
142.03
131.92
129.09
126.08
118.86
110.06
77.32 CDCl3
77.00 CDCl3
76.68 CDCl3
65.57
52.89
35.50
32.18
24.68
f1 (ppm)

Dimethyl (2*E*)-2-(4-formylbenzylidene)cyclopentane-1,1-dicarboxylate (21).

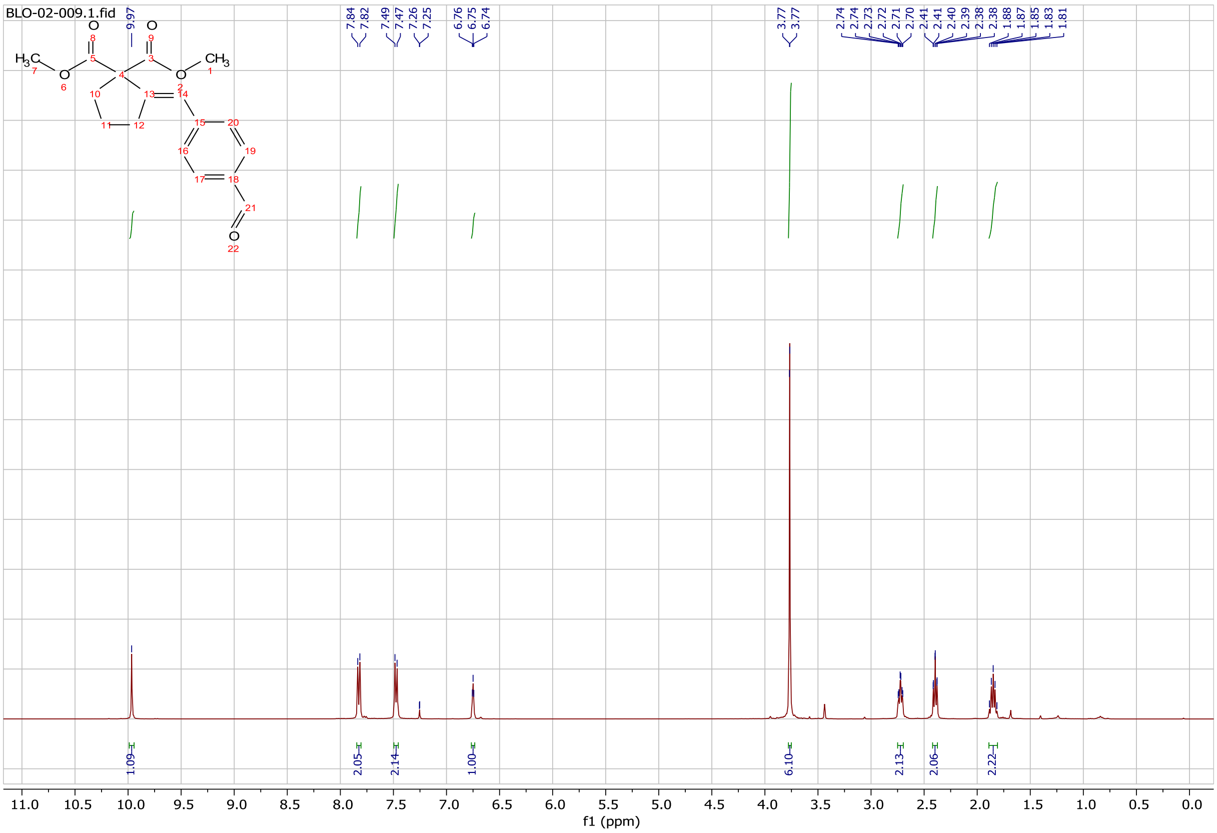

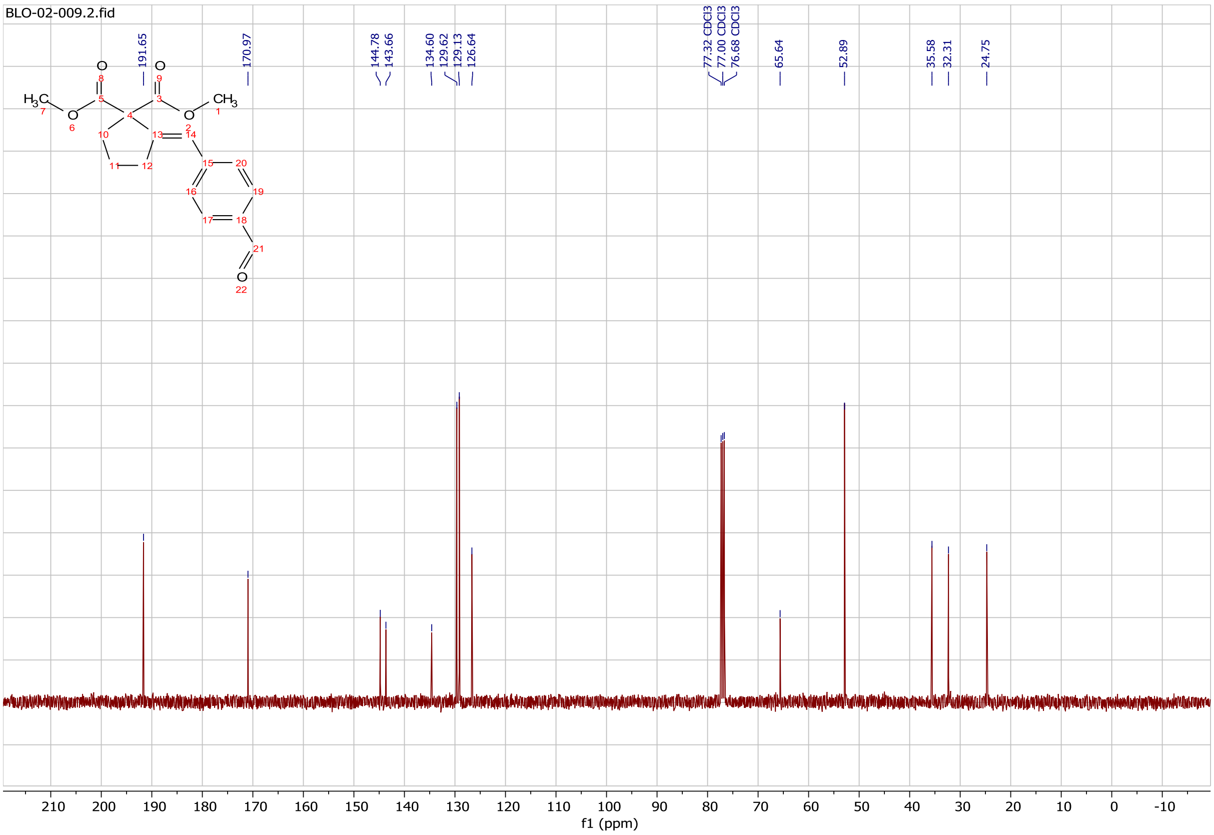

Dimethyl (2*E*)-2-(3-((*tert*-butoxycarbonyl)amino)benzylidene)cyclopentane-1,1-dicarboxylate (22).

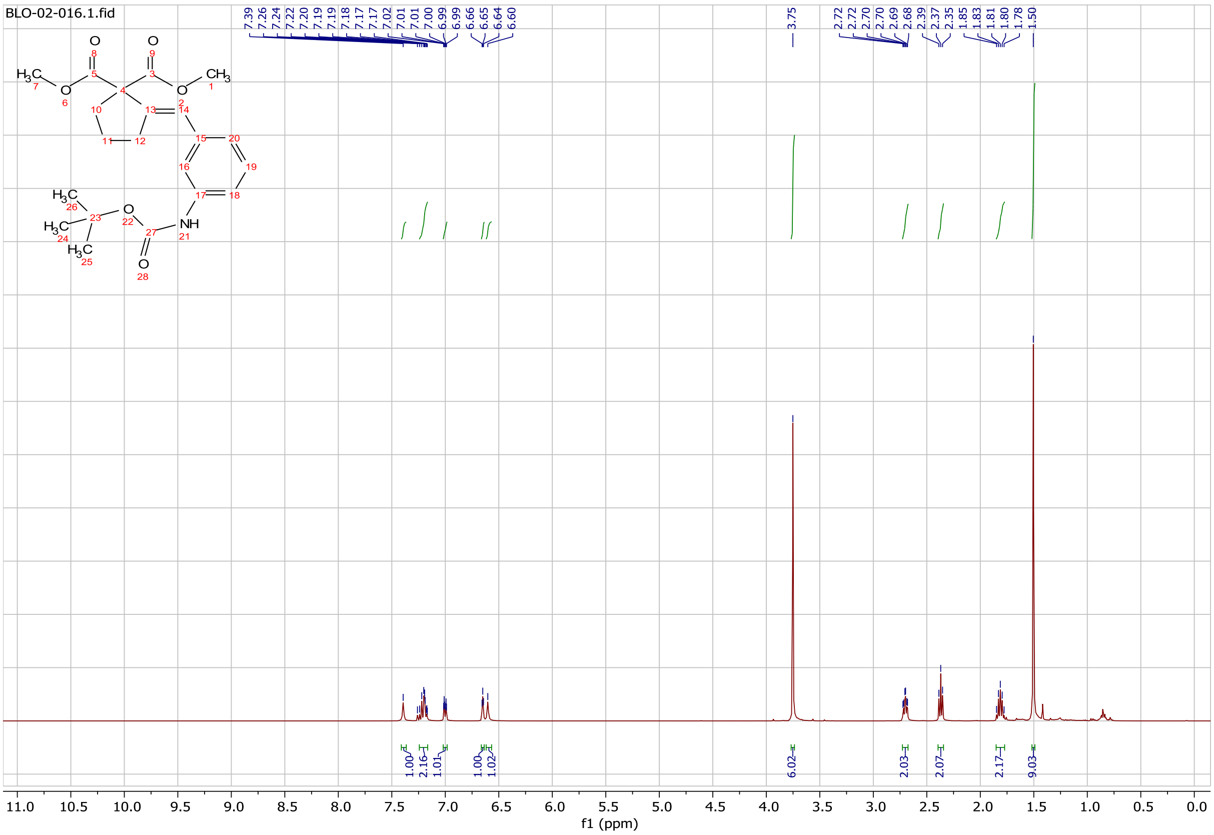

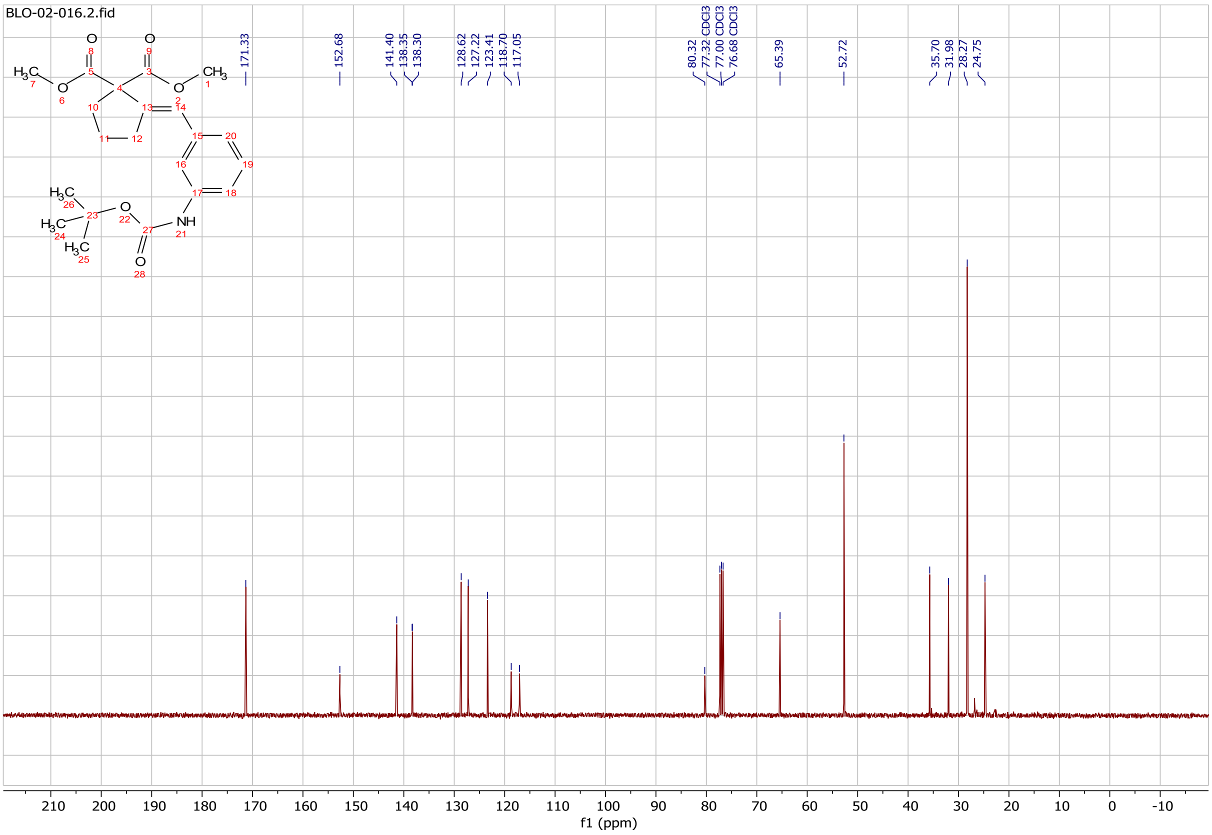

Dimethyl (2*E*)-2-(thiophen-2-ylmethylidene)cyclopentane-1,1-dicarboxylate (23).

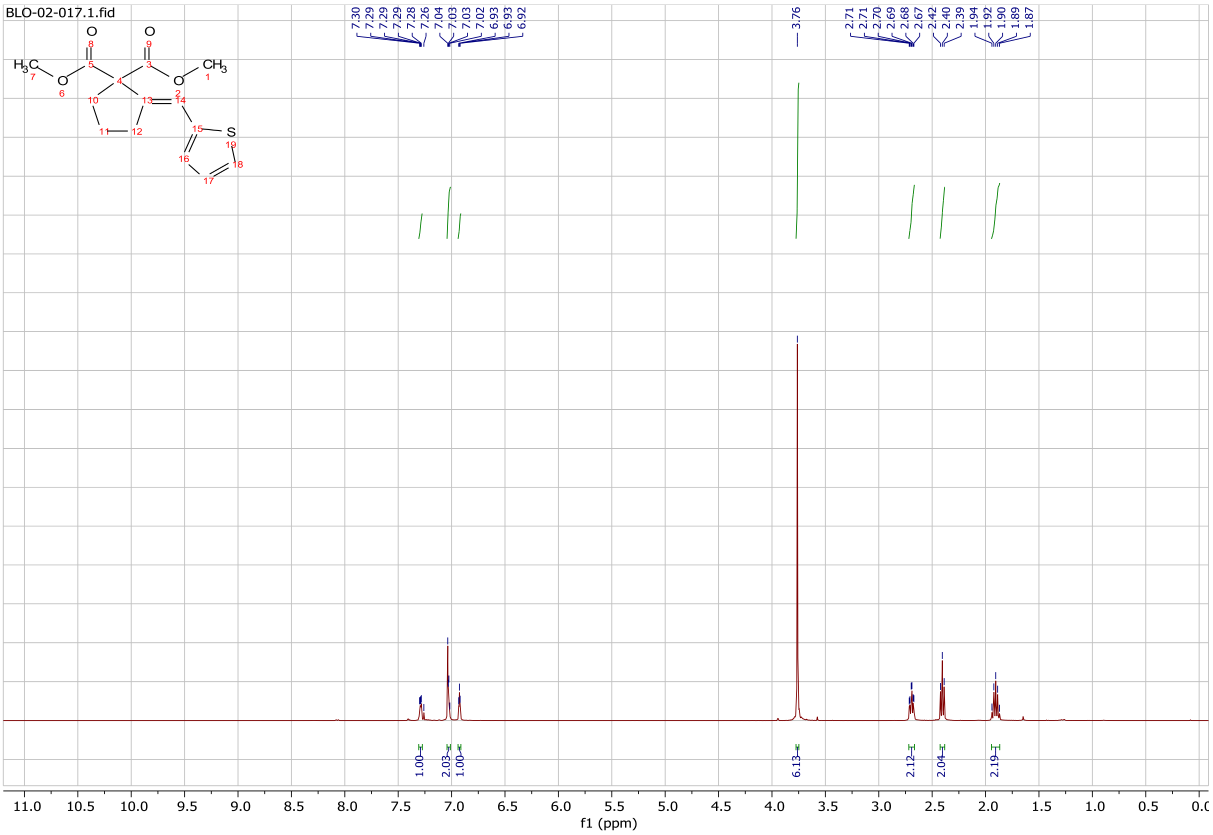

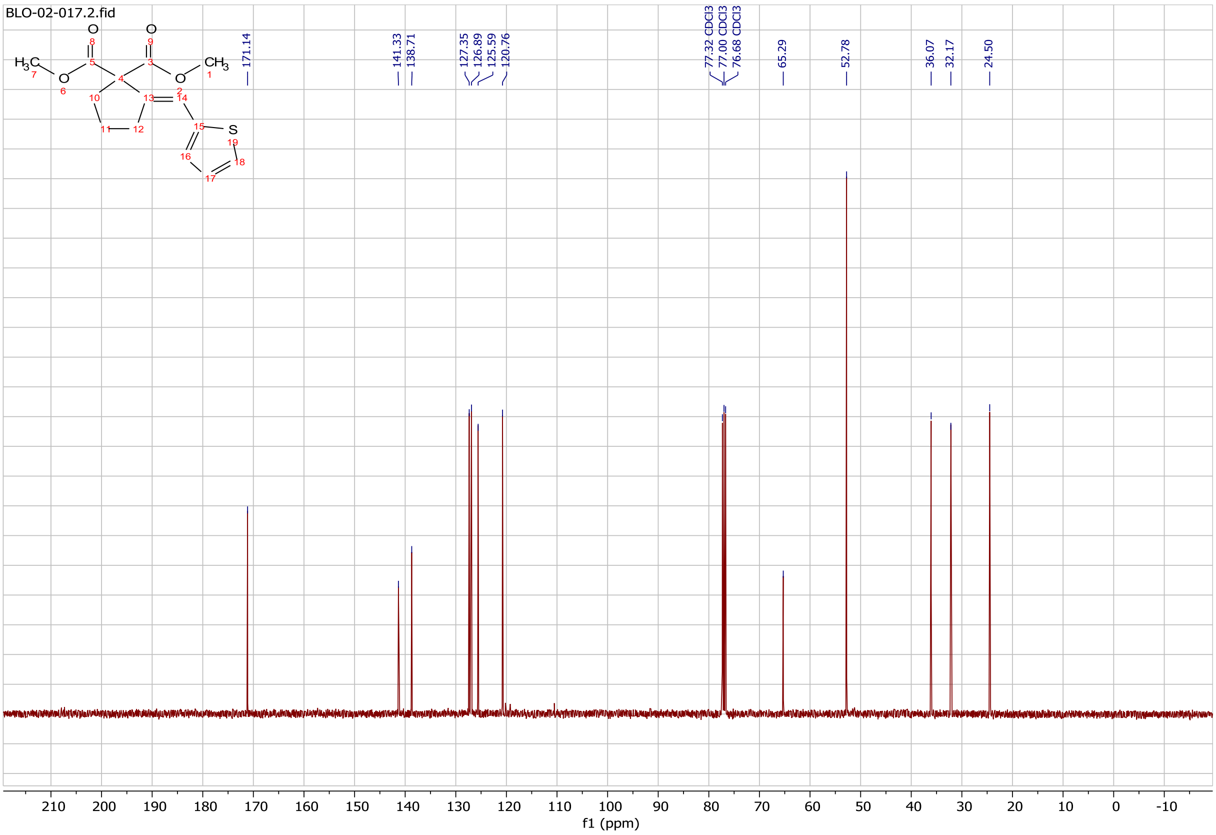

Dimethyl (2*E*)-2-(2-chlorobenzylidene)cyclopentane-1,1-dicarboxylate (24).

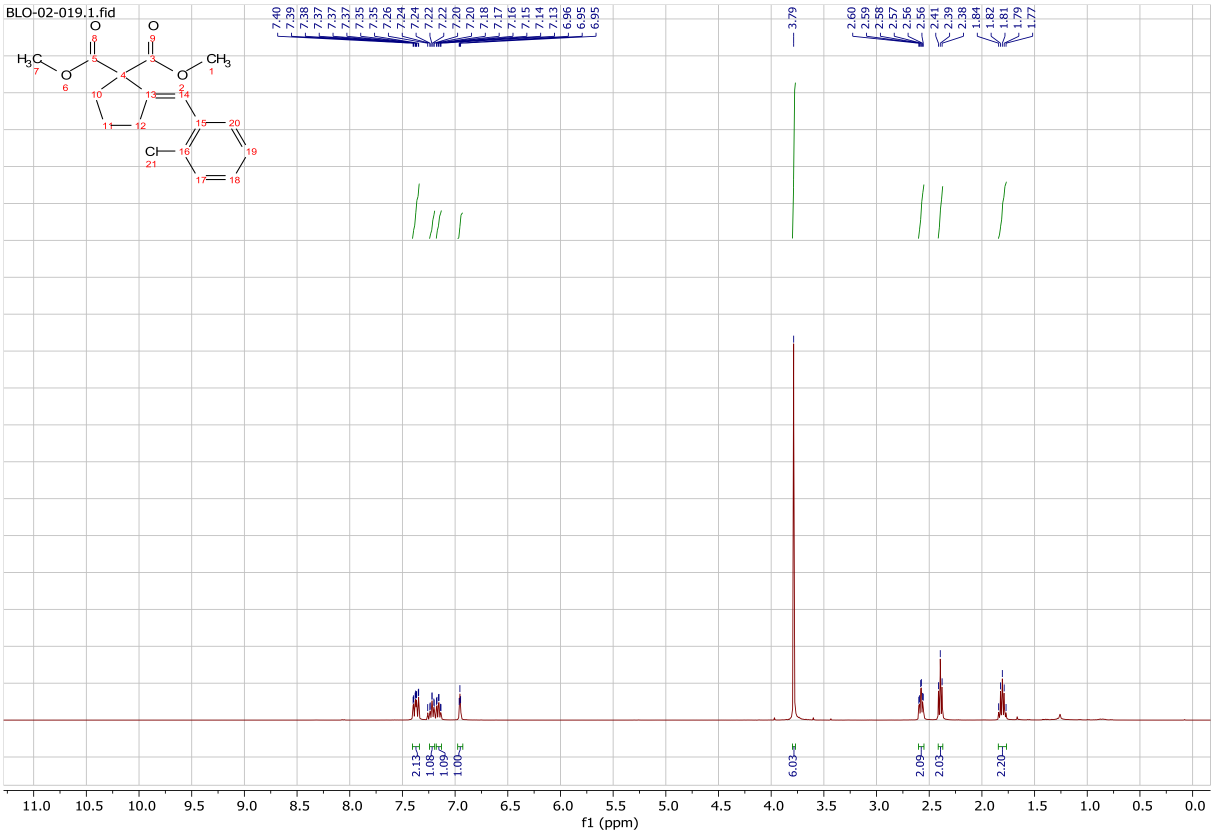

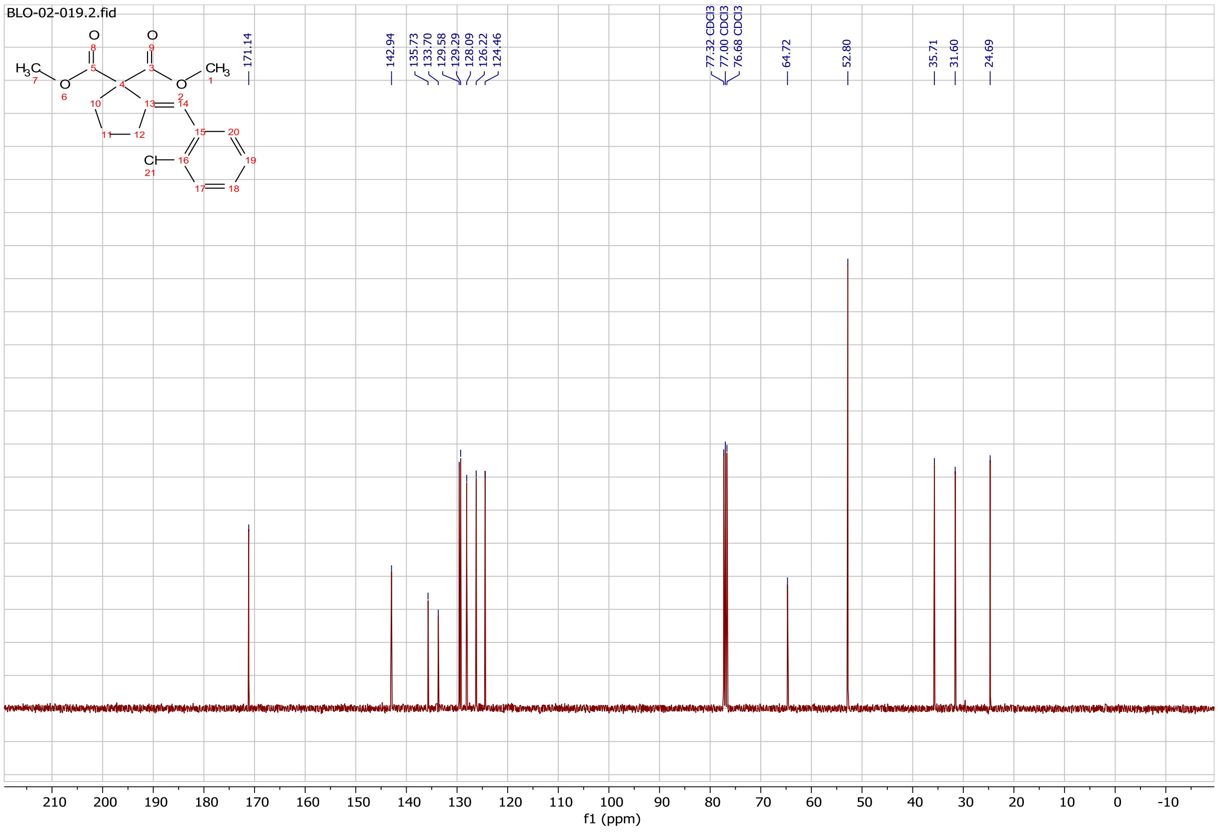

Dimethyl (2*E*)-2-(4-nitrobenzylidene)cyclopentane-1,1-dicarboxylate (25).

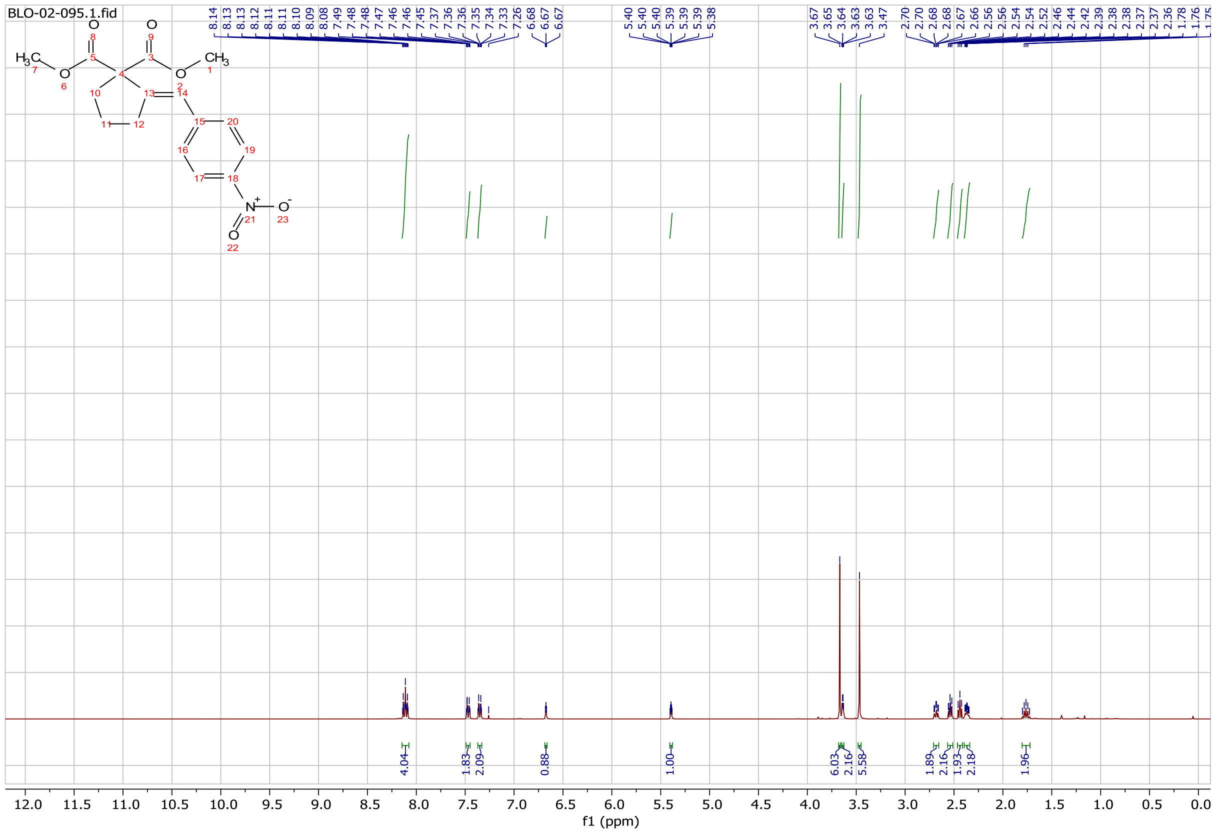

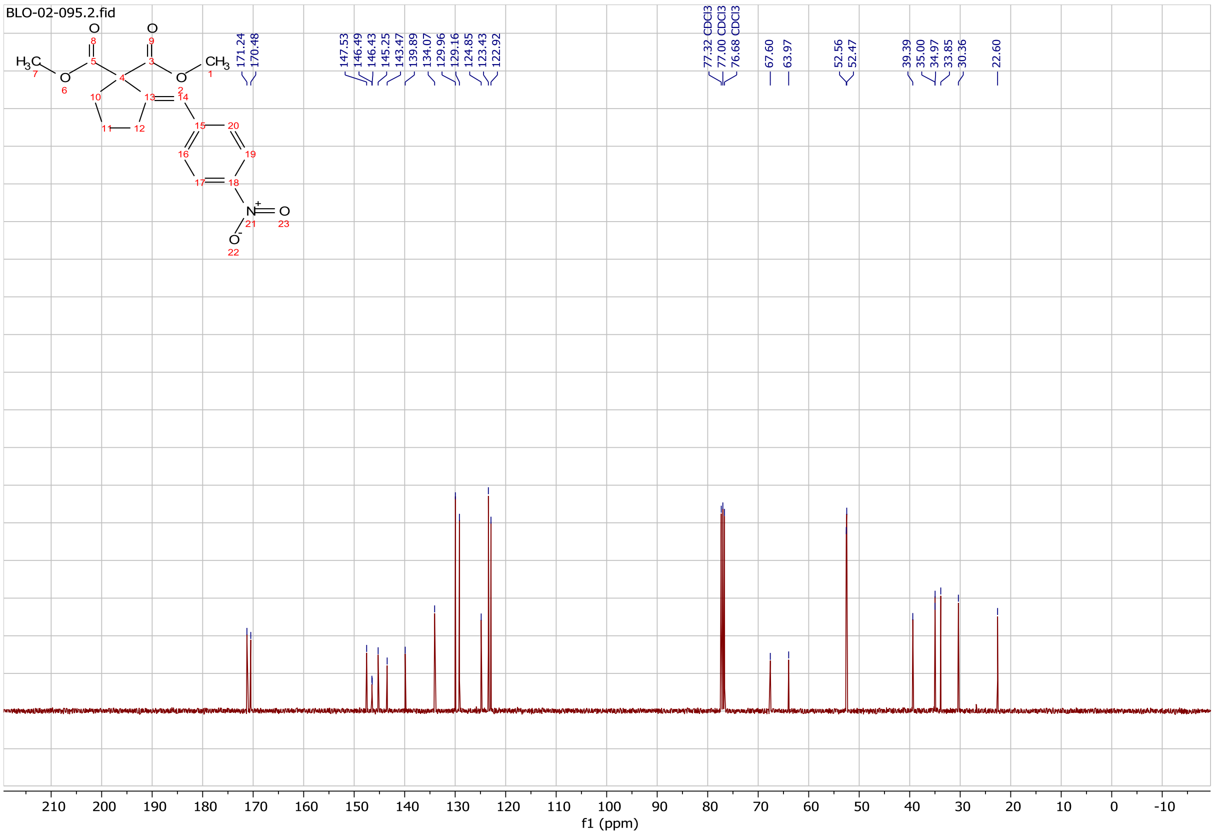

Di(propan-2-yl) (2*E*)-2-benzylidenecyclopentane-1,1-dicarboxylate (26).

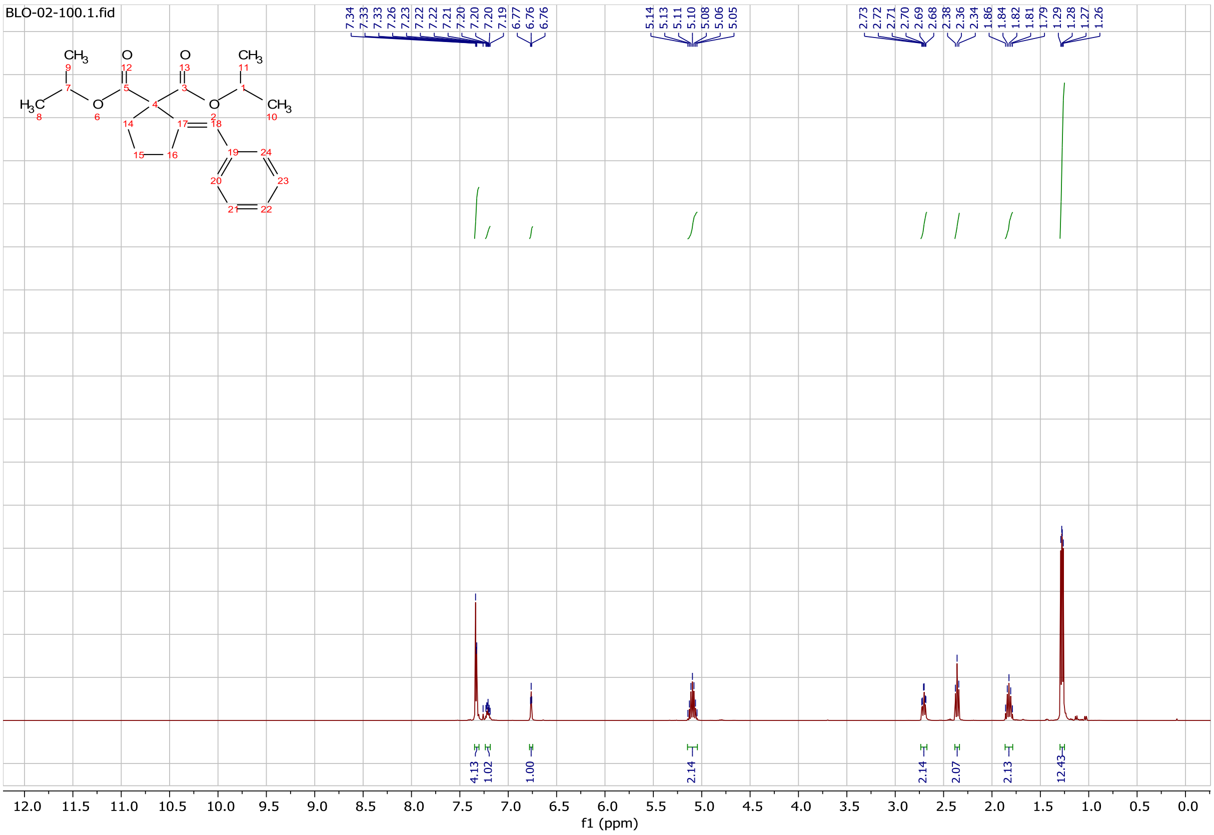

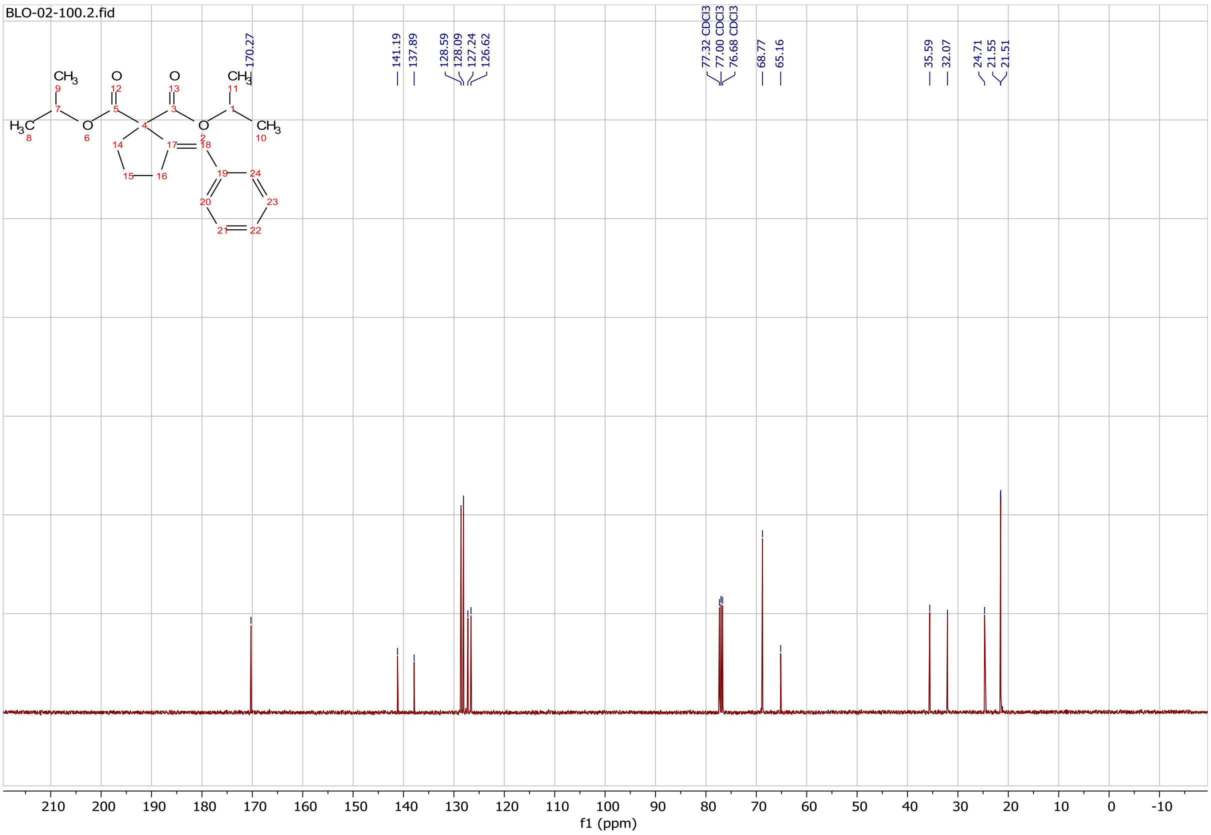

Di(propan-2-yl) (2*E*)-2-(4-methoxybenzylidene)cyclopentane-1,1-dicarboxylate (27).

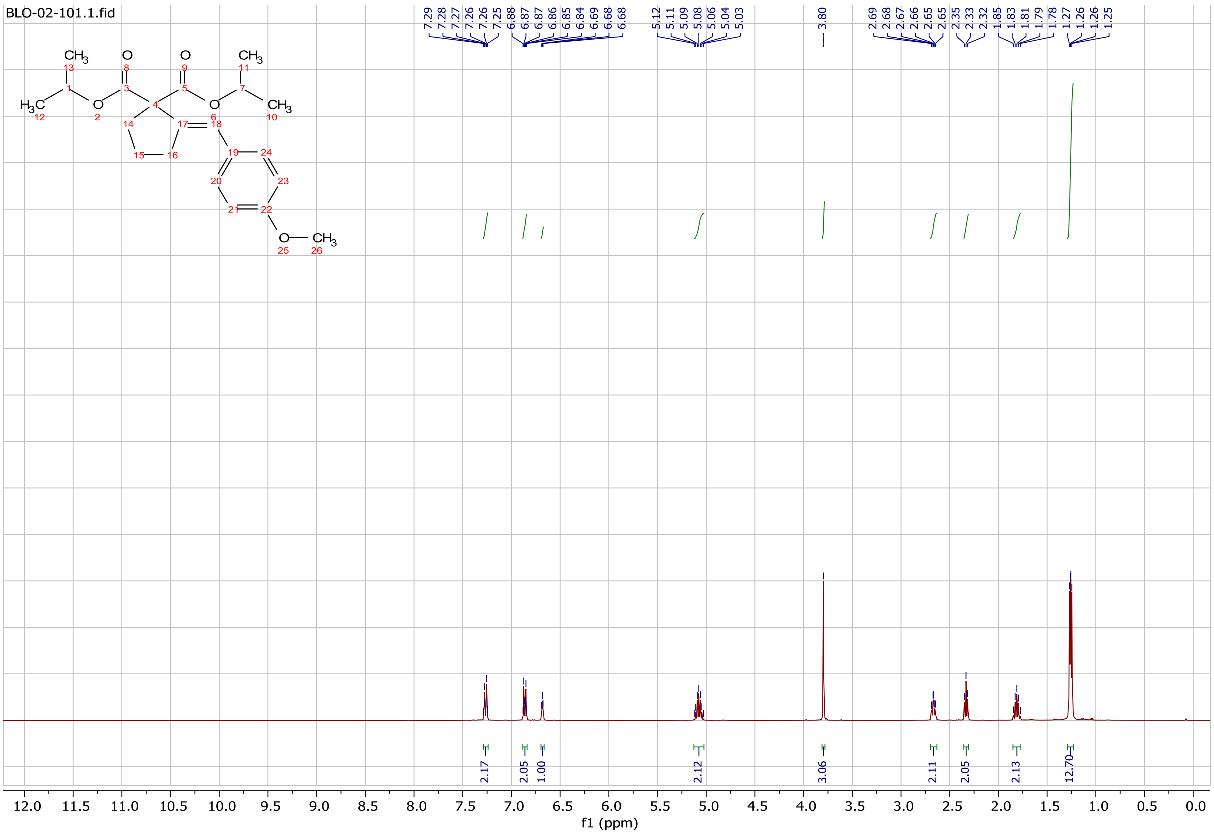

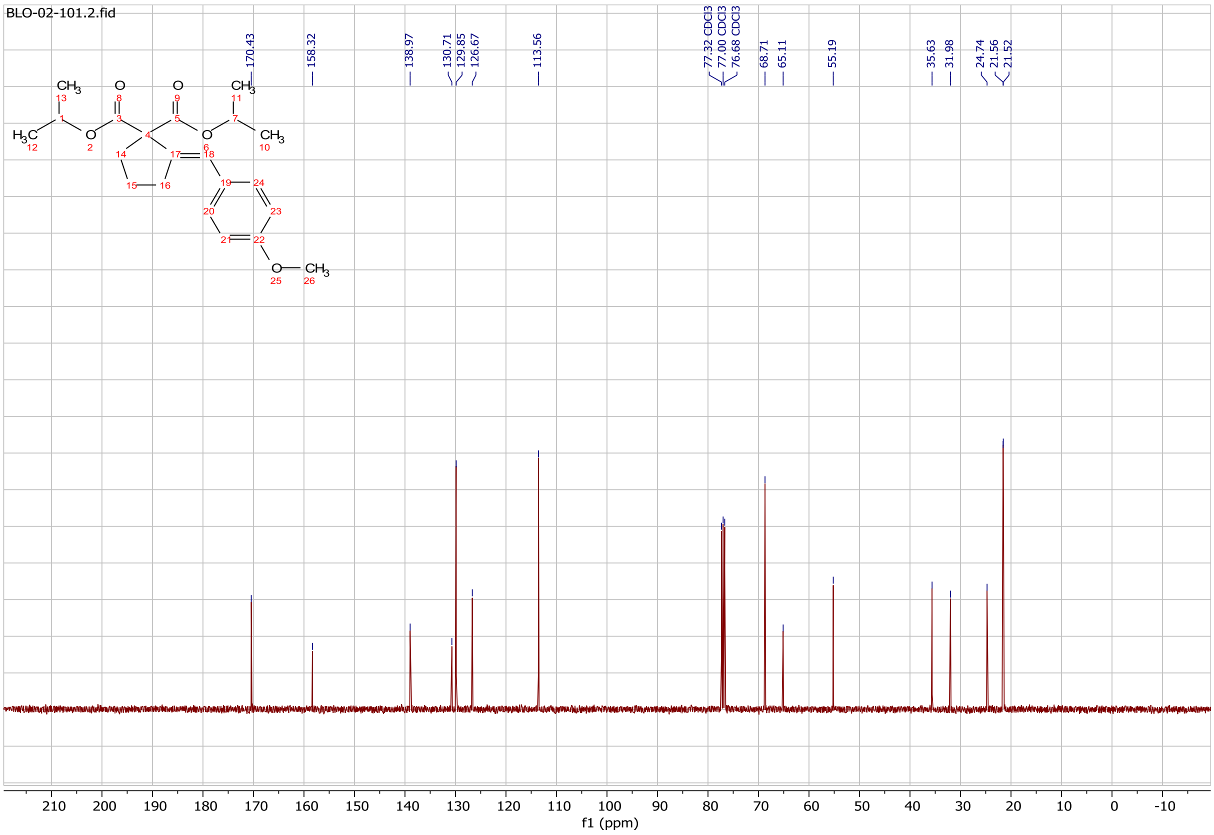

Di(propan-2-yl) (2*E*)-2-(4-cyanobenzylidene)cyclopentane-1,1-dicarboxylate (28).

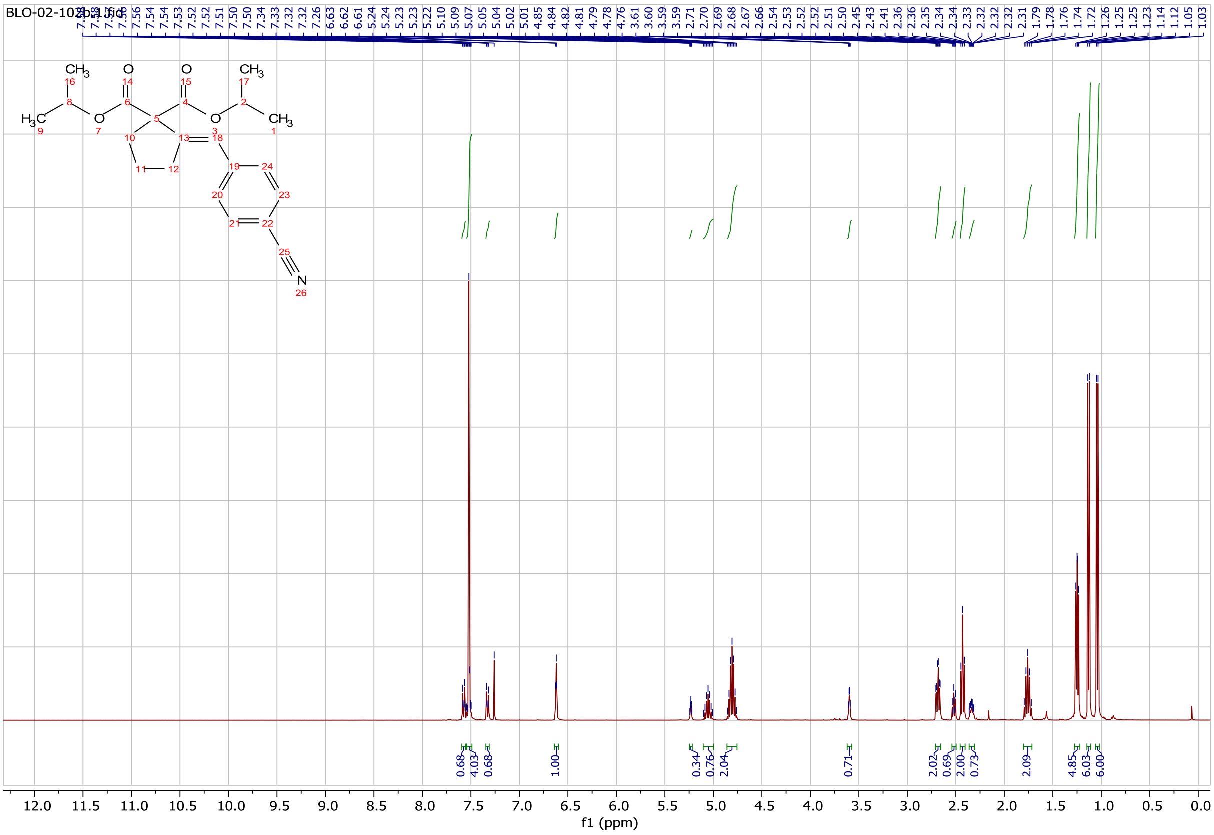

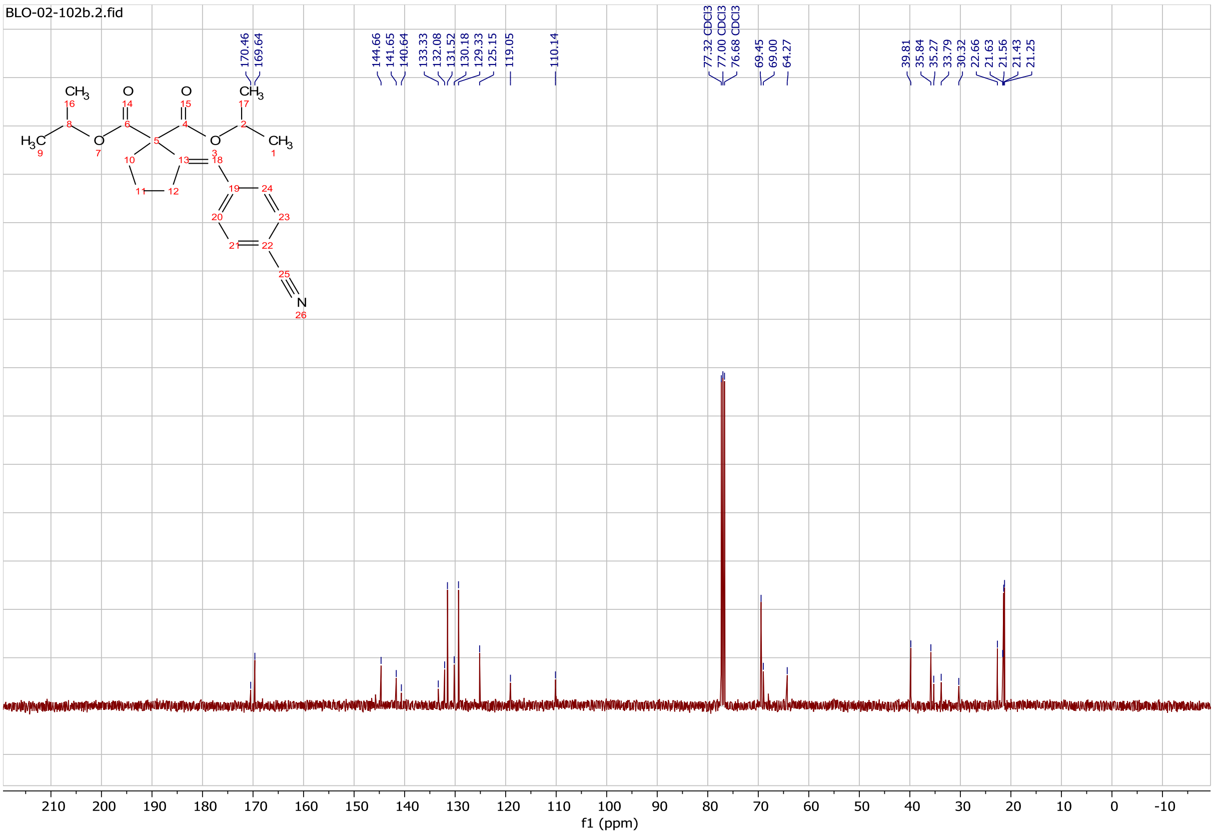

Di-*tert*-butyl (2*E*)-2-benzylidenecyclopentane-1,1-dicarboxylate (29).

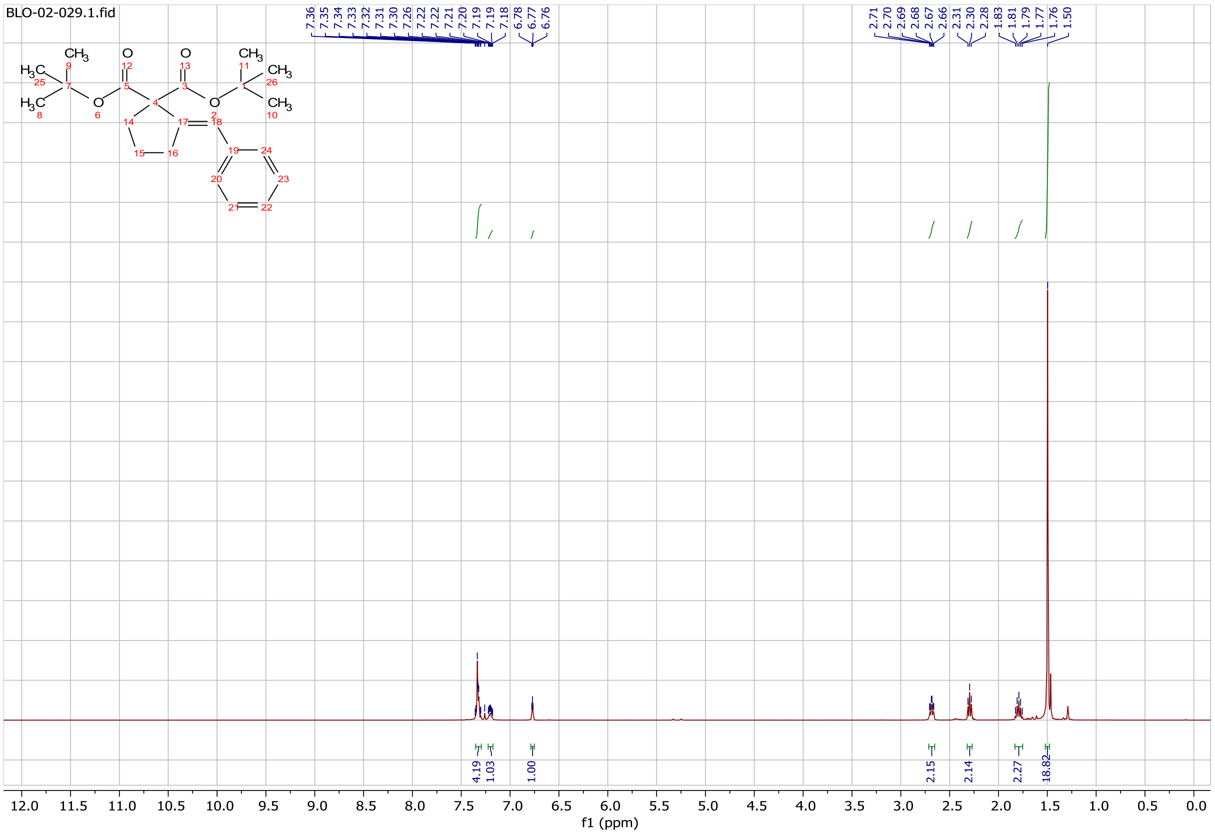

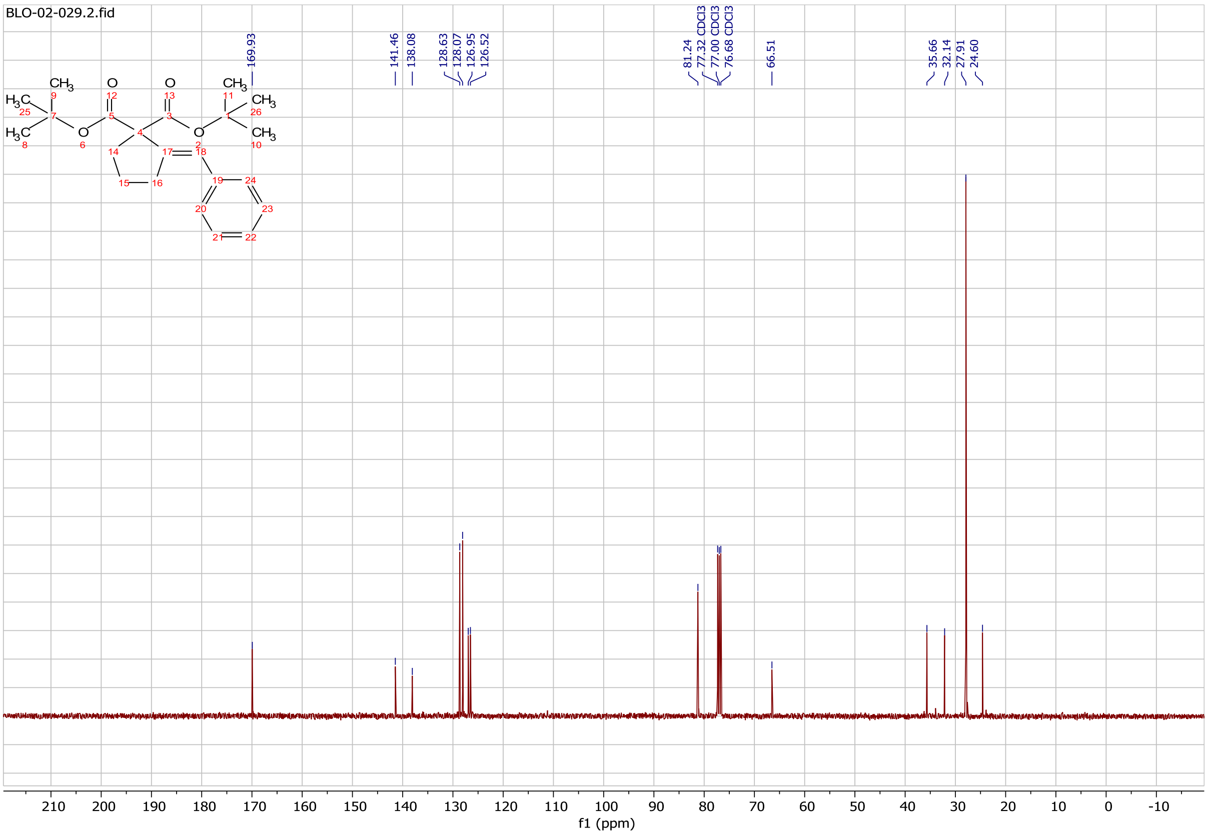

Di-*tert*-butyl (2*E*)-2-(4-methoxybenzylidene)cyclopentane-1,1-dicarboxylate (30).

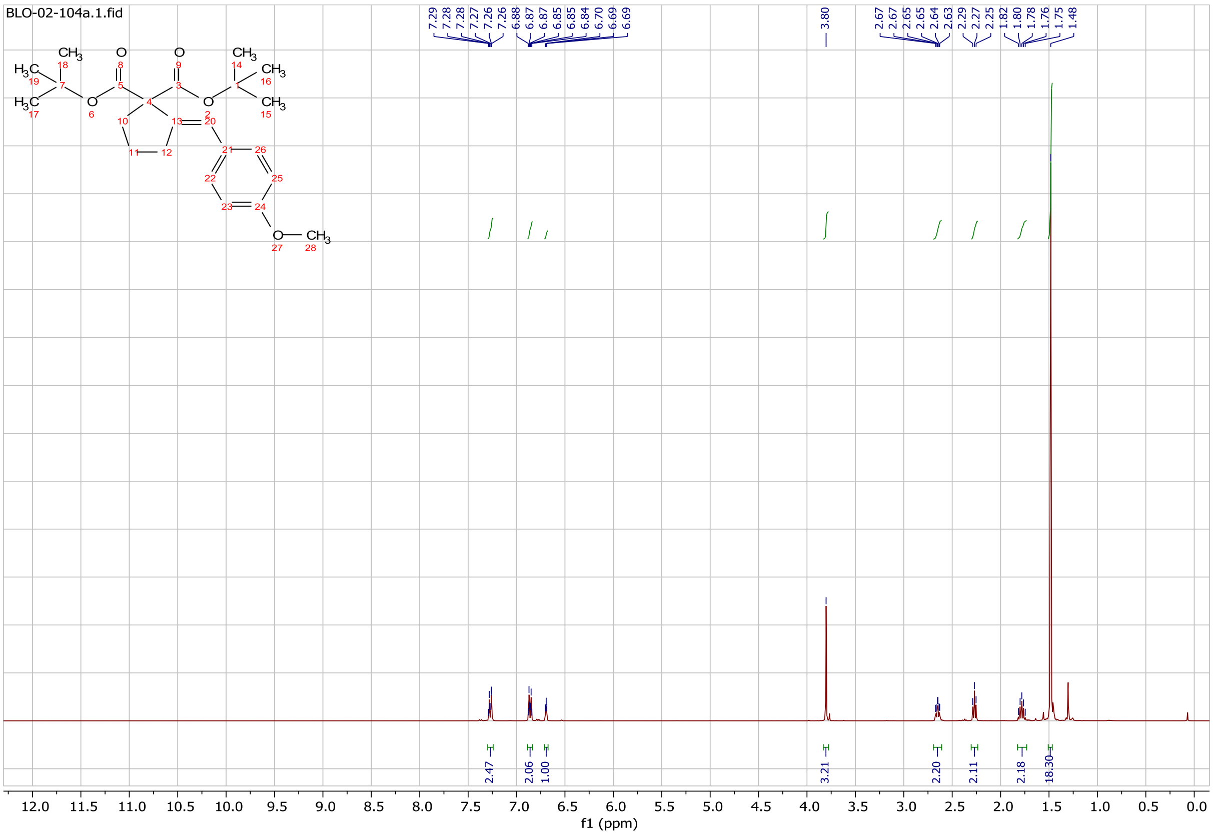

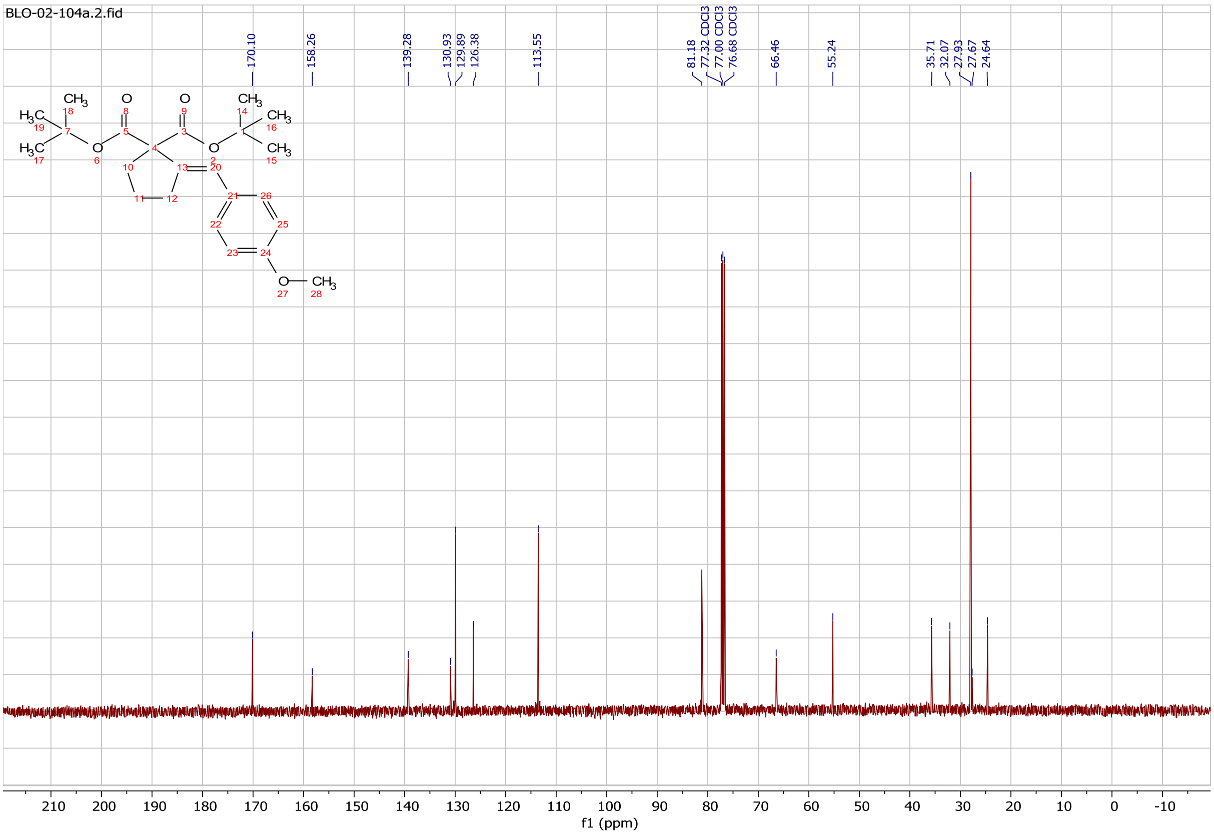

Di-*tert*-butyl (2*E*)-2-(4-cyanobenzylidene)cyclopentane-1,1-dicarboxylate (31).

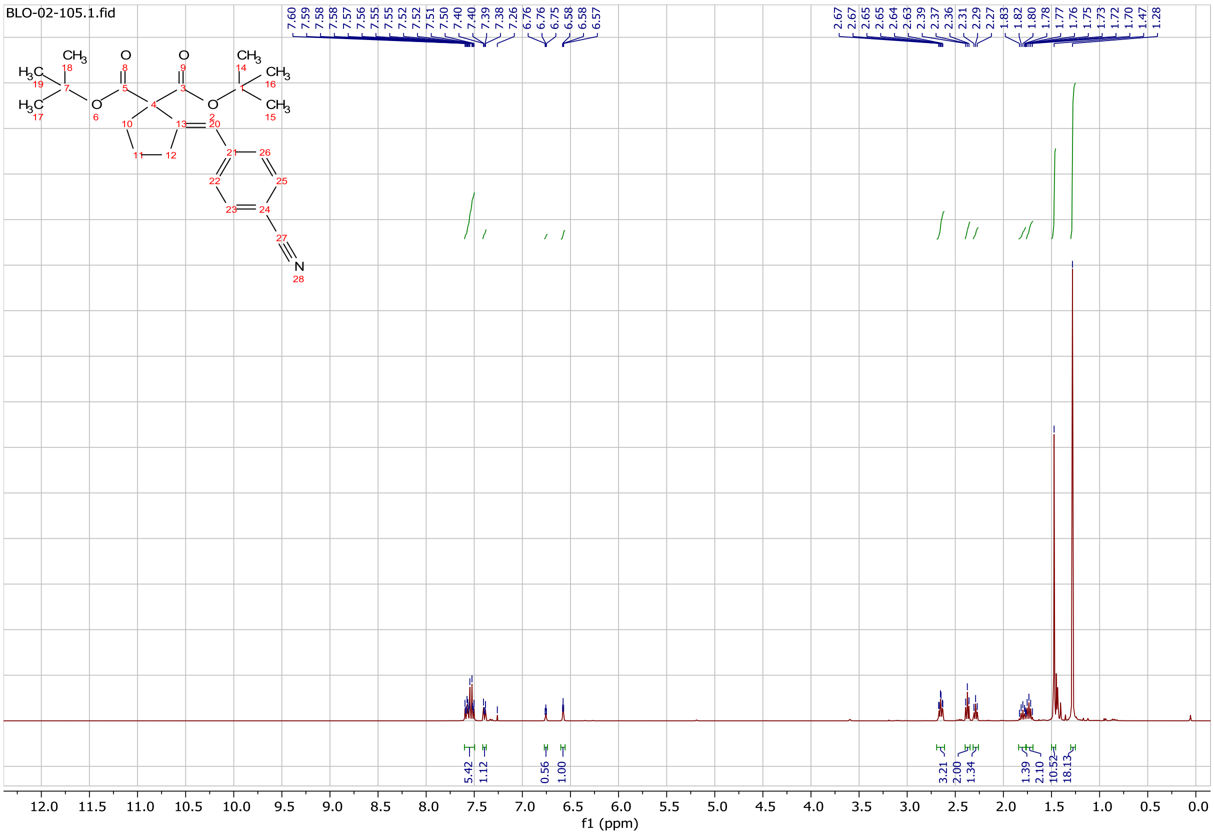

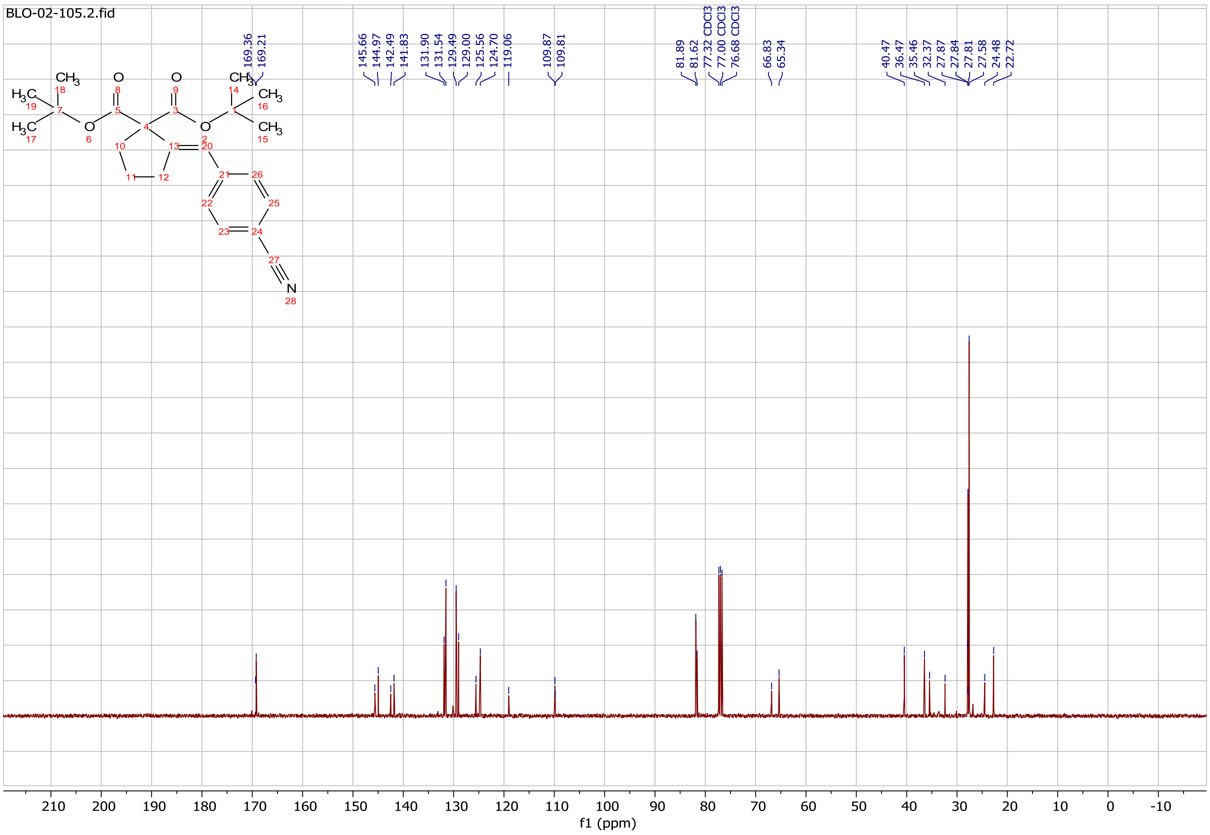

***tert*-butyl (2*E*)-2-benzylidene-1-cyanocyclopentanecarboxylate (32).**

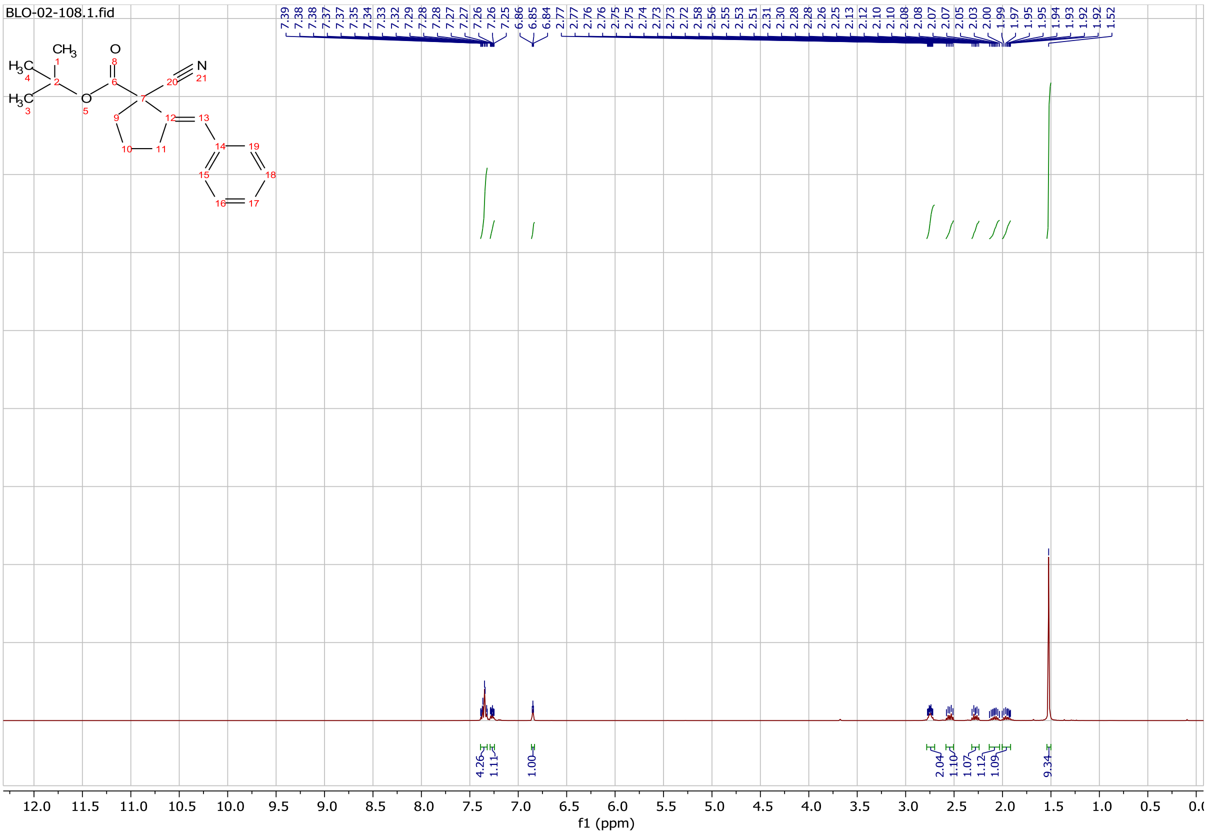

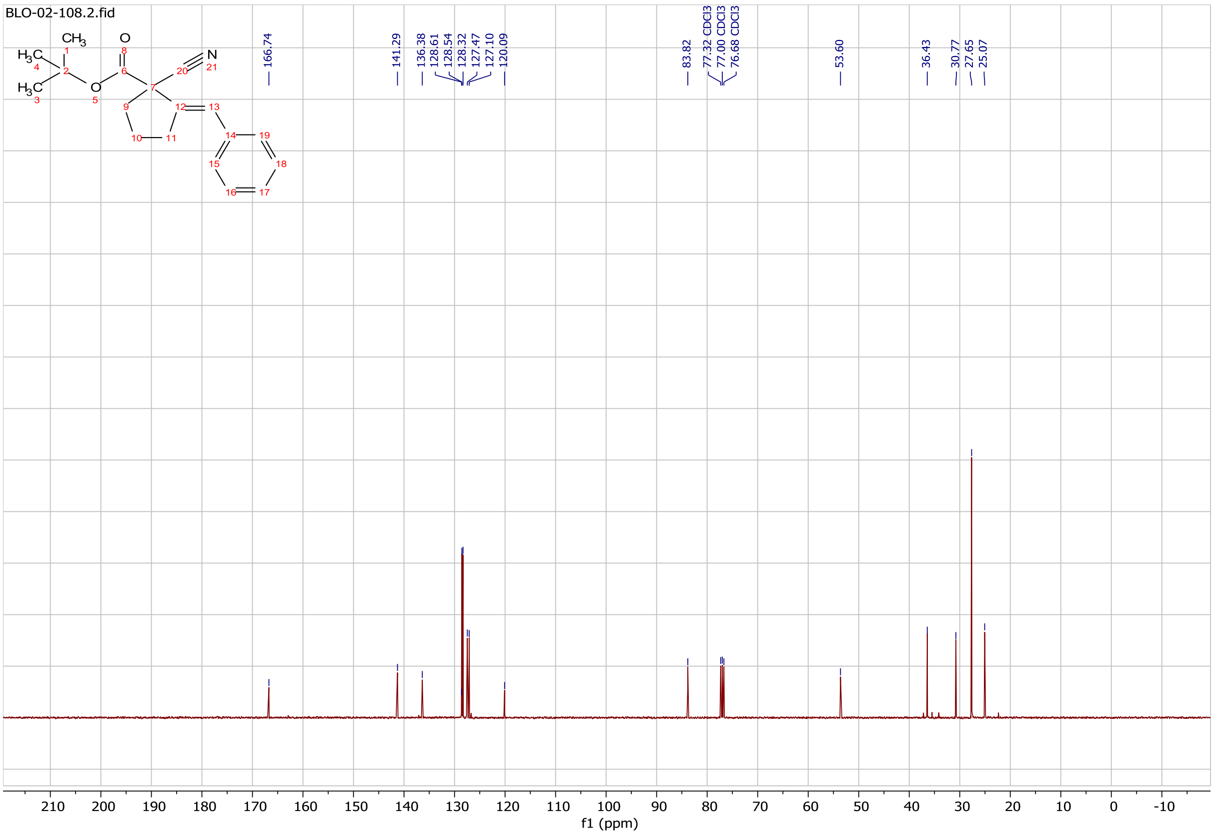

tert-butyl (2_E_)-1-cyano-2-(4-methoxybenzylidene)cyclopentanecarboxylate (33).

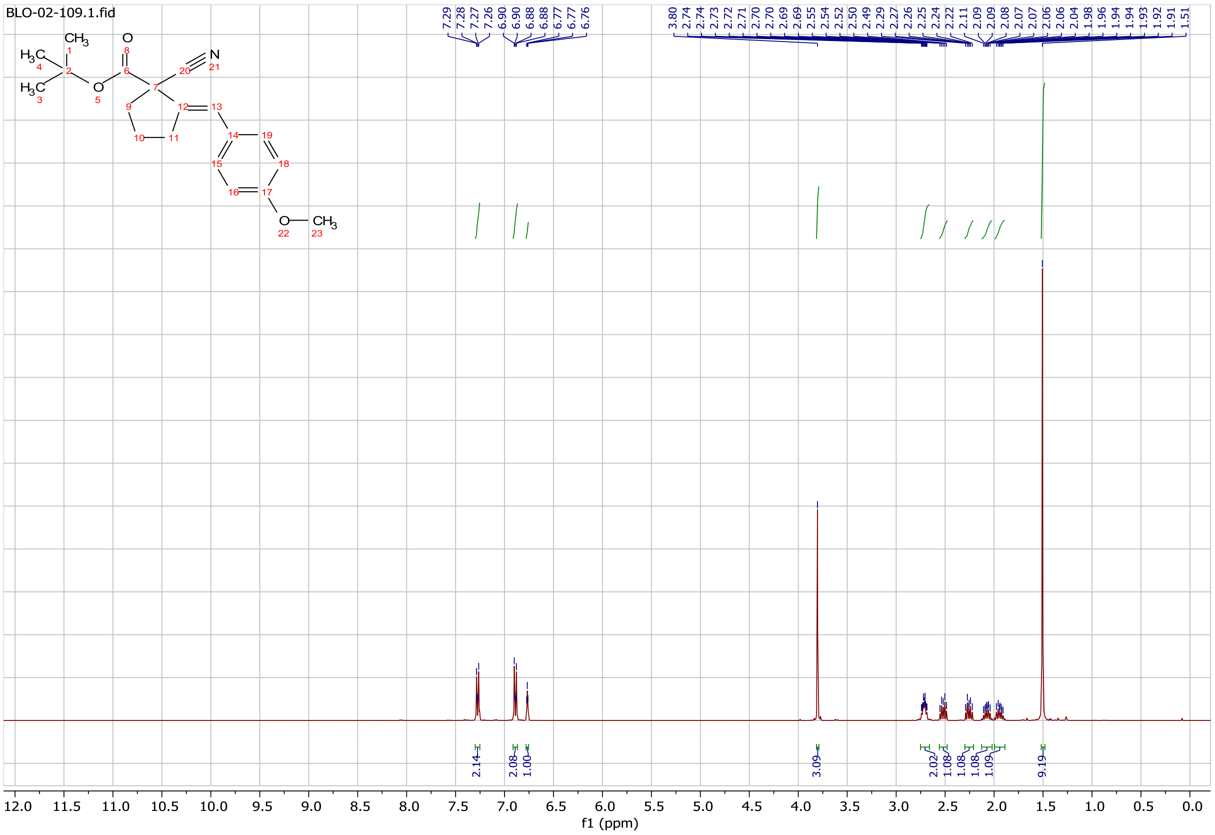

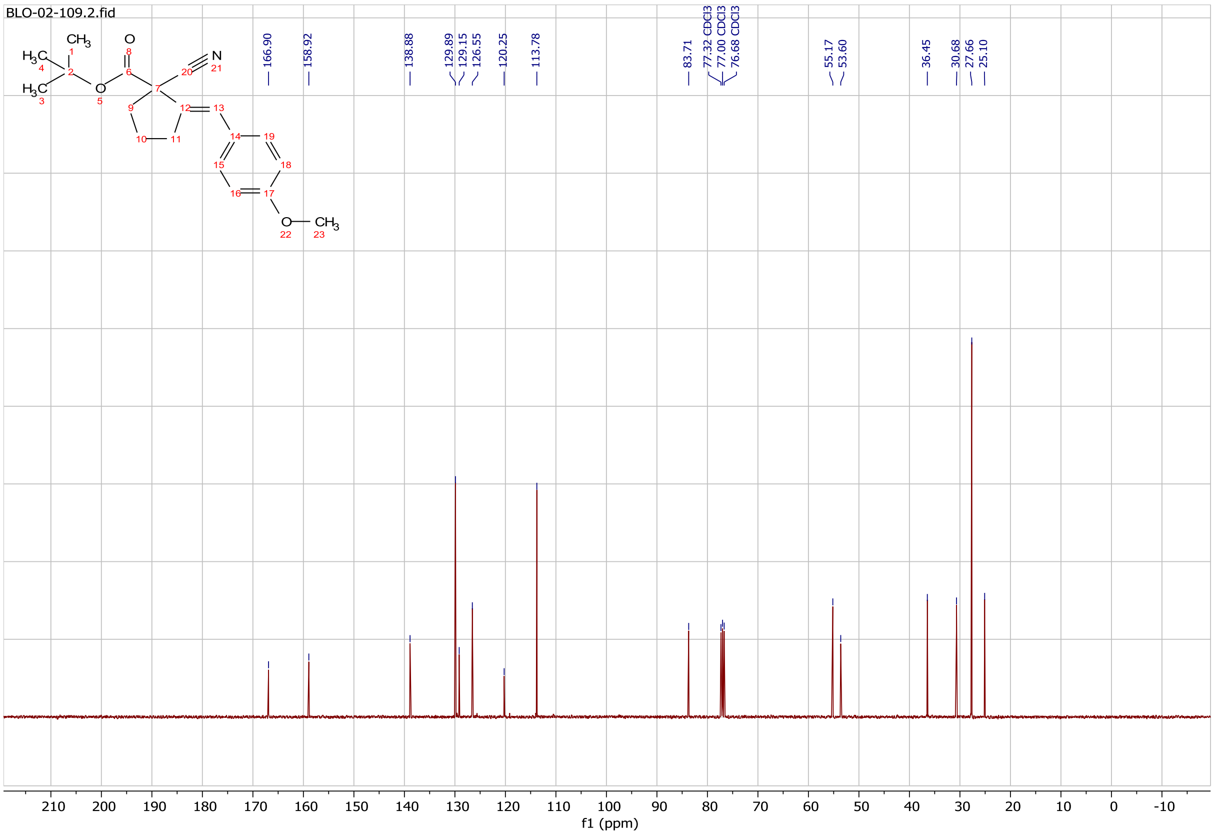

***tert*-butyl (2*E*)-1-cyano-2-(4-cyanobenzylidene)cyclopentanecarboxylate (34).**

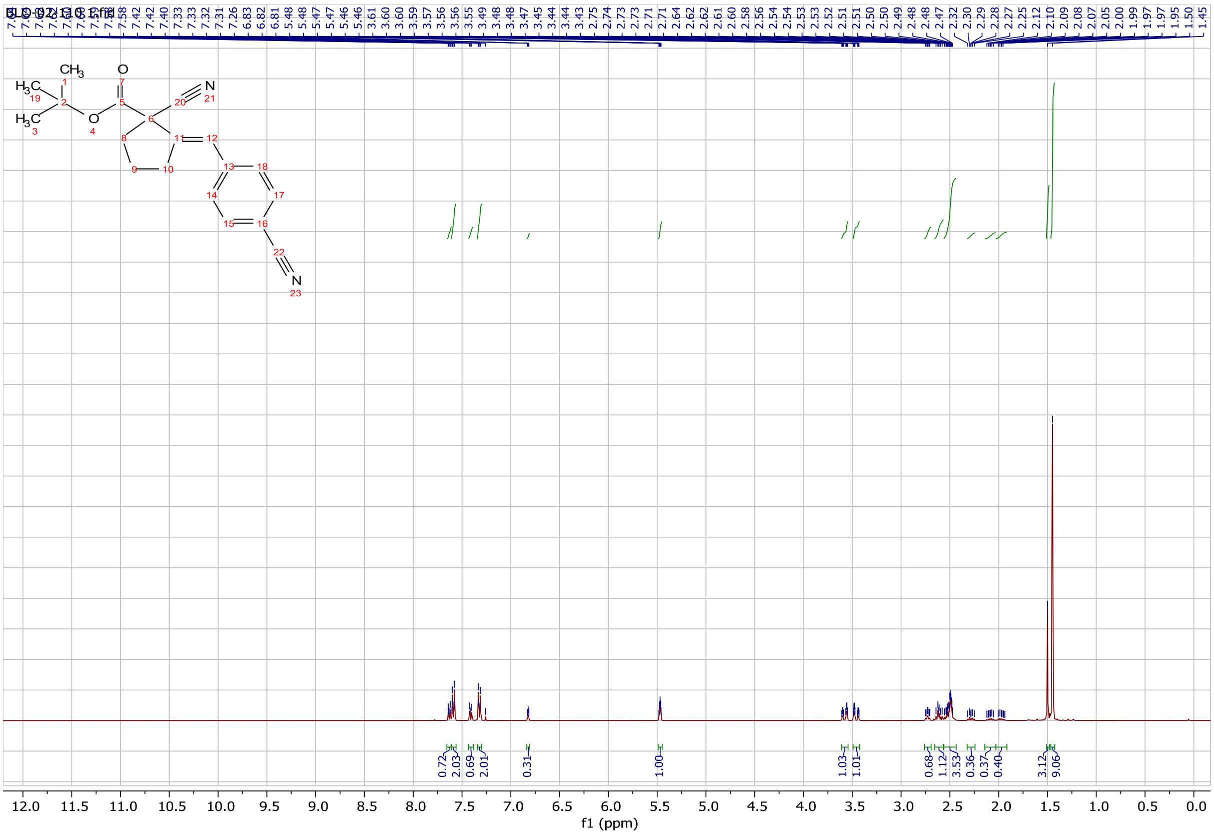

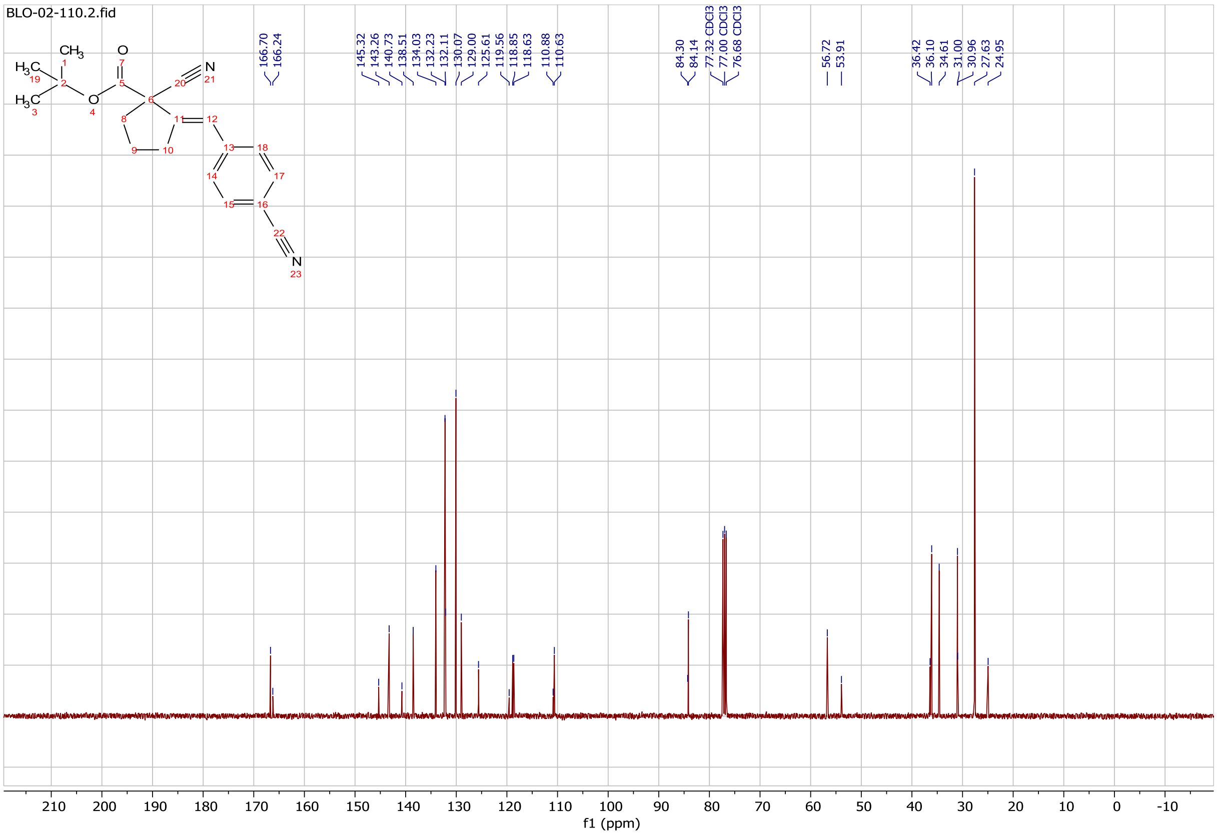

Propan-2-yl (2*E*)-2-benzylidene-1-cyanocyclopentanecarboxylate (35).

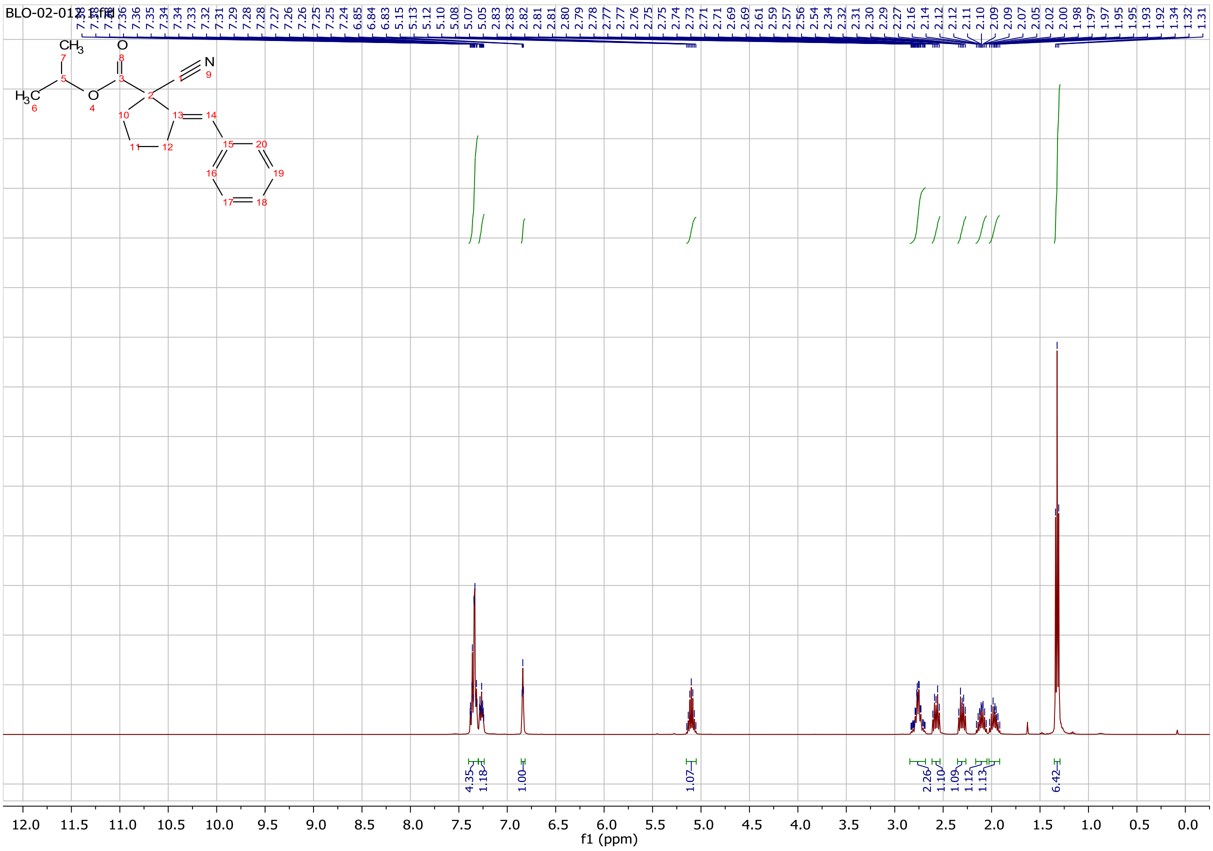

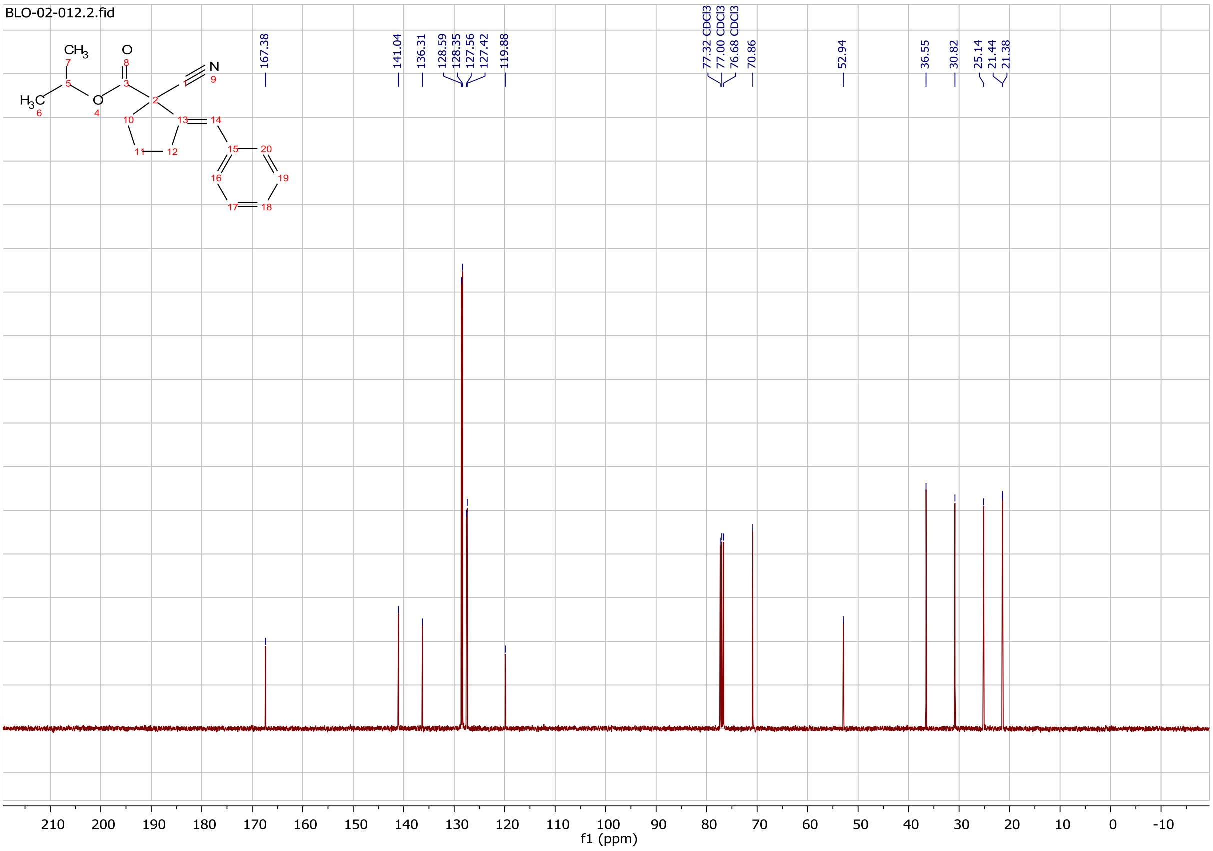

Propan-2-yl (2*E*)-1-cyano-2-(4-methoxybenzylidene) cyclopentanecarboxylate (36).

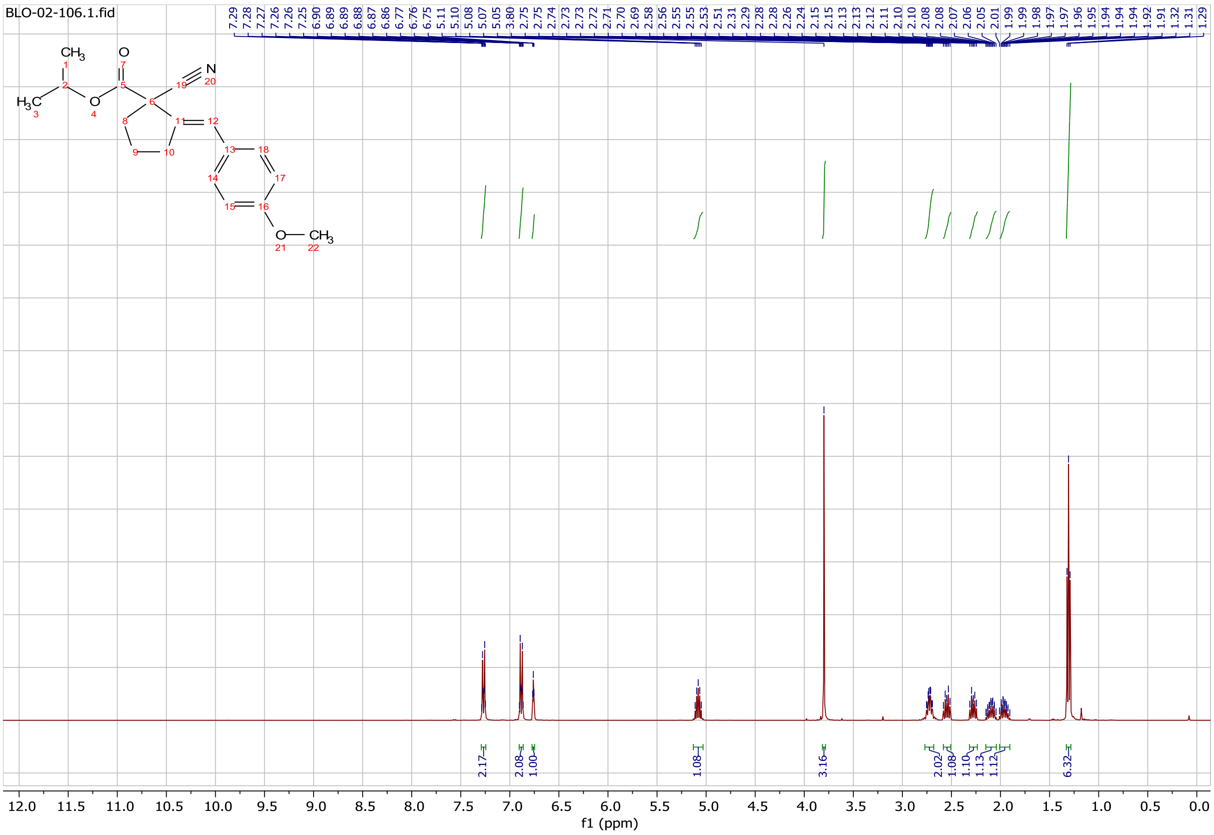

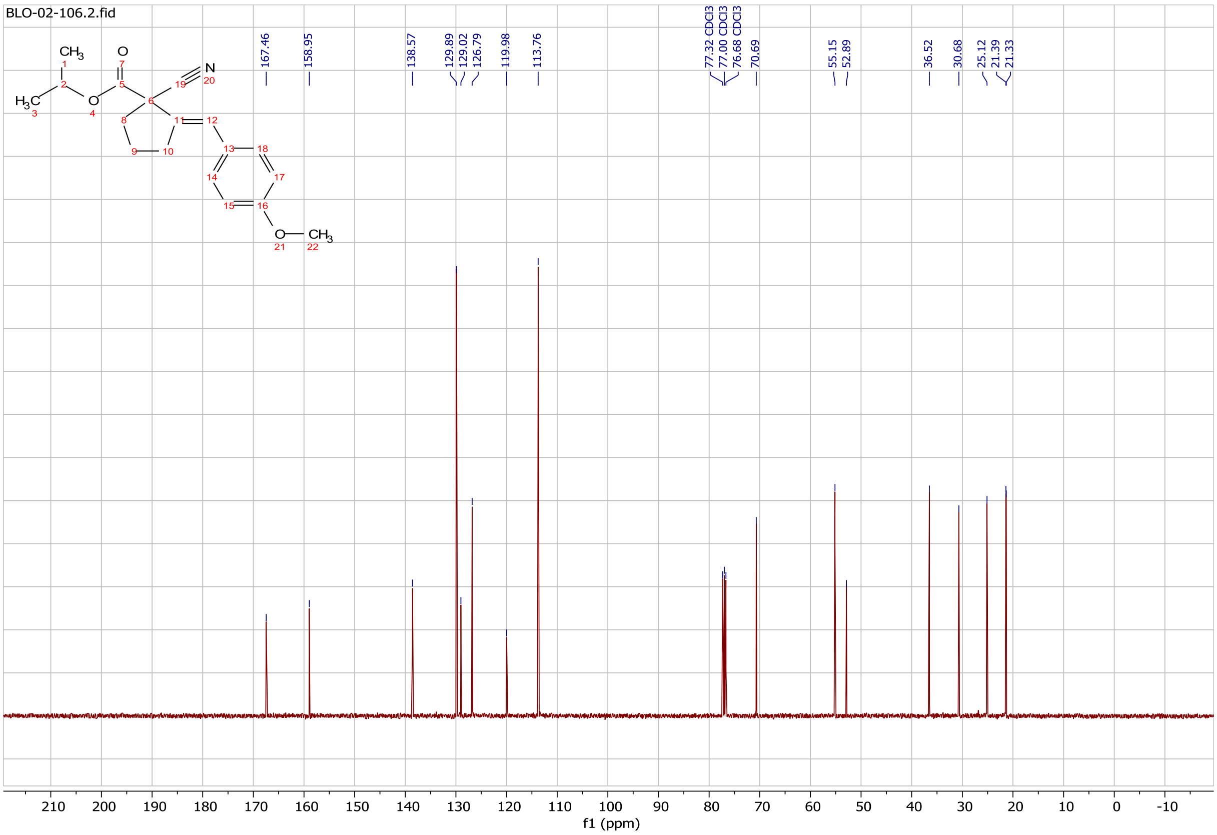

Propan-2-yl (2*E*)-1-cyano-2-(4-cyanobenzylidene)cyclopentanecarboxylate (37).

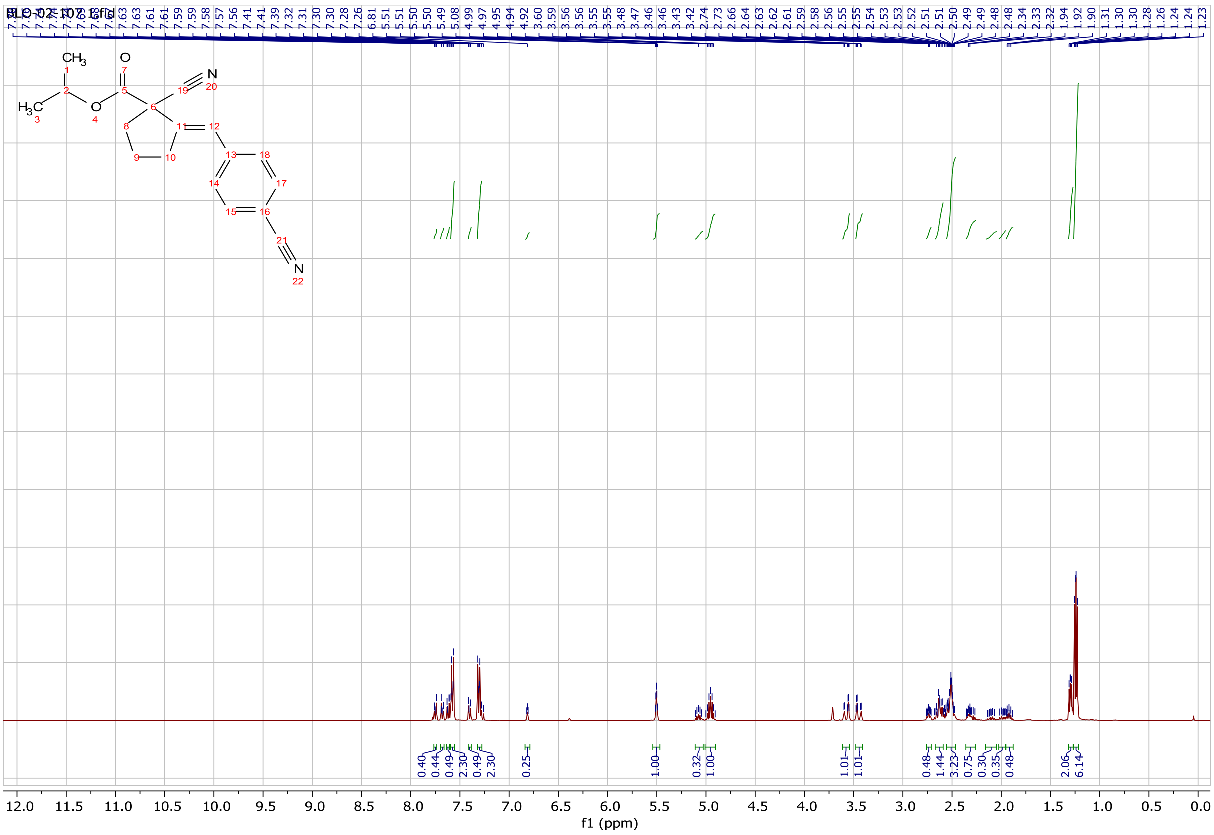

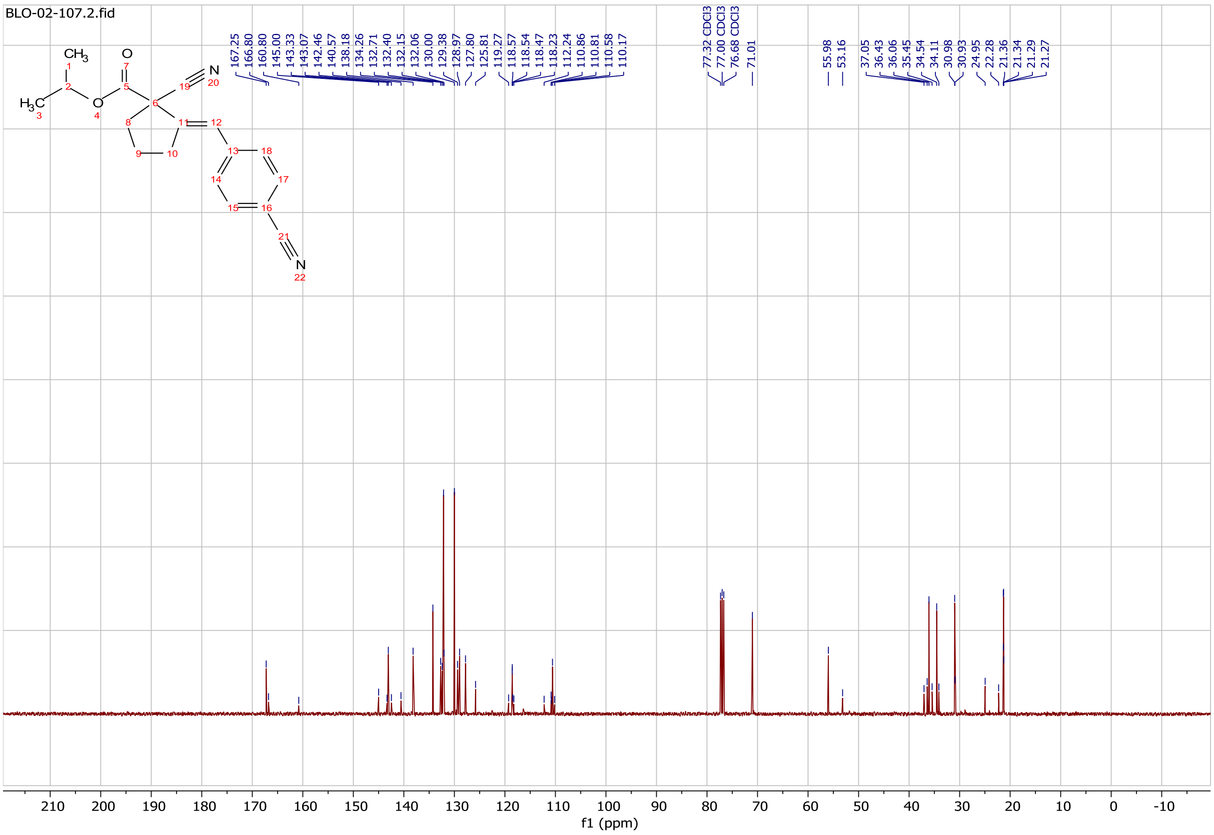

(2*E*)-2-benzylidenecyclopentane-1,1-dicarbonitrile (38).

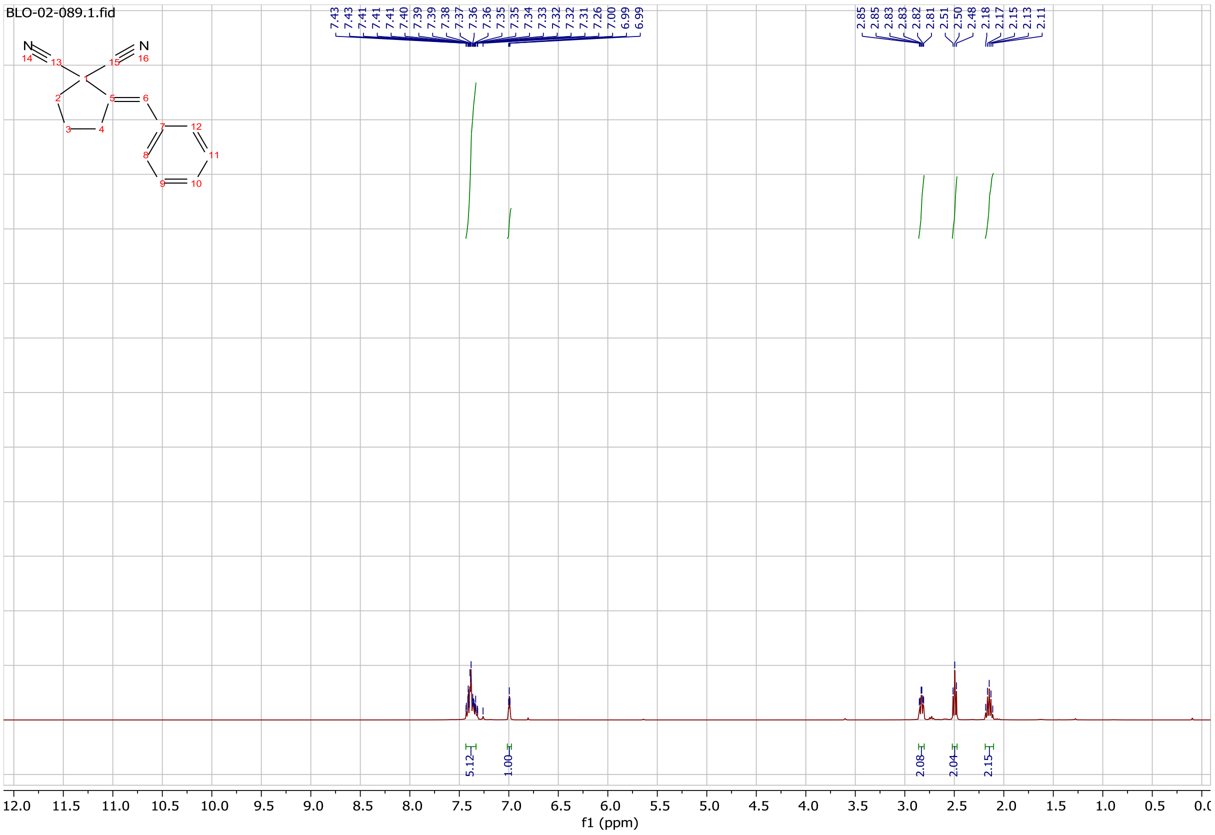

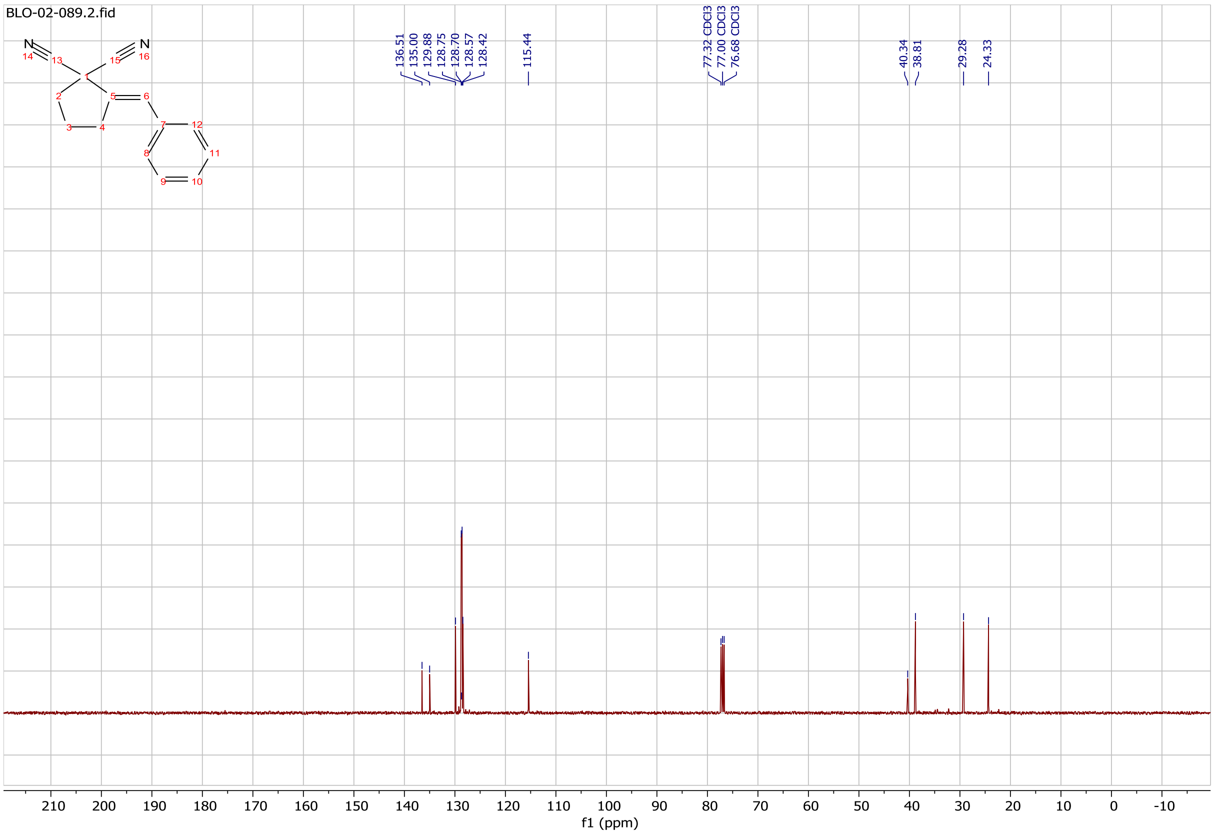

(2*E*)-2-(4-methoxybenzylidene)cyclopentane-1,1-dicarbonitrile (39).

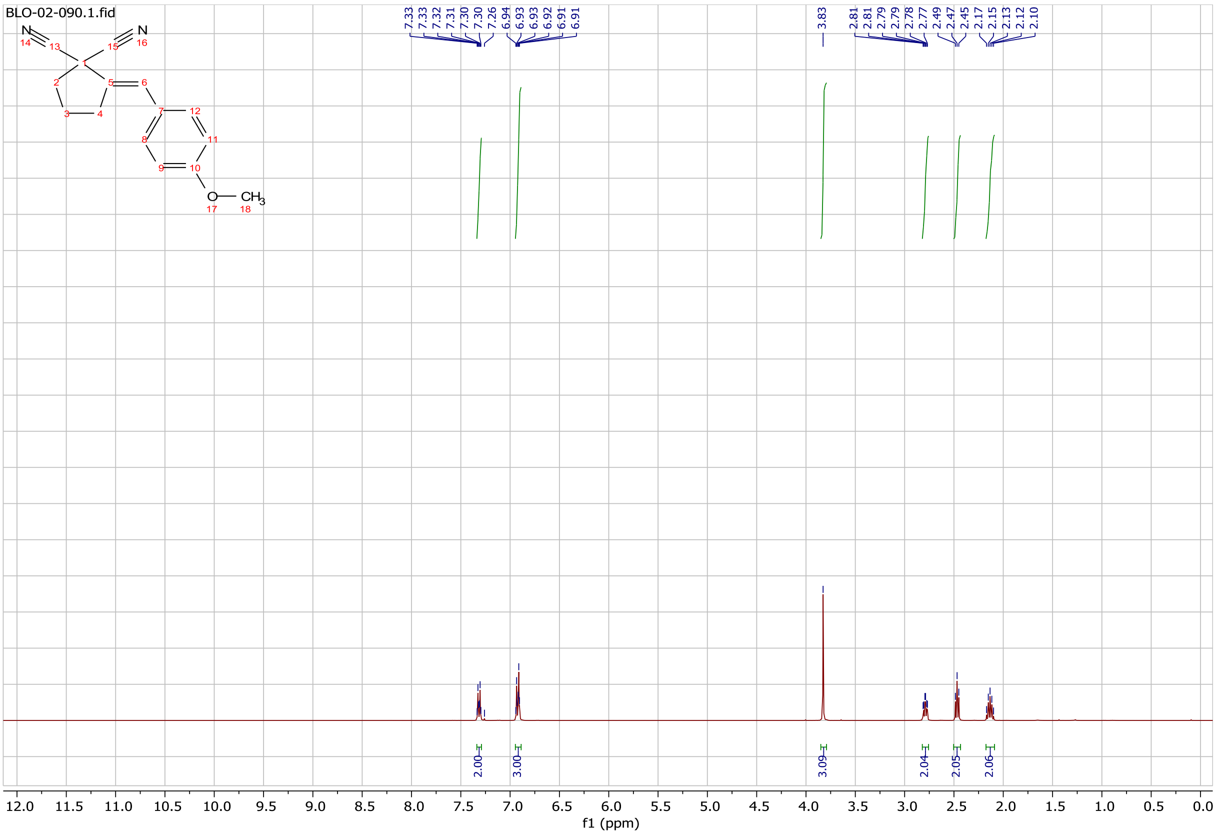

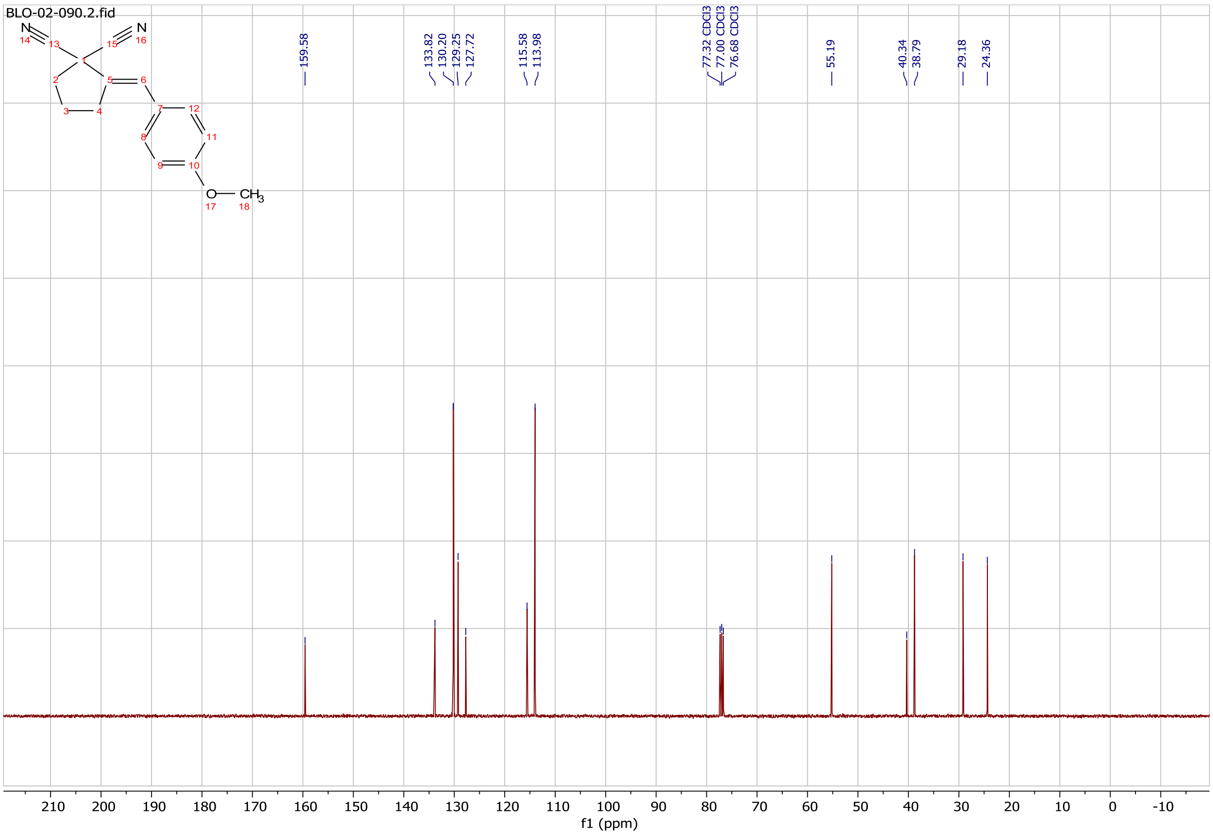

***(E)*-methyl 1-acetyl-2-benzylidenecyclopentanecarboxylate (40).**

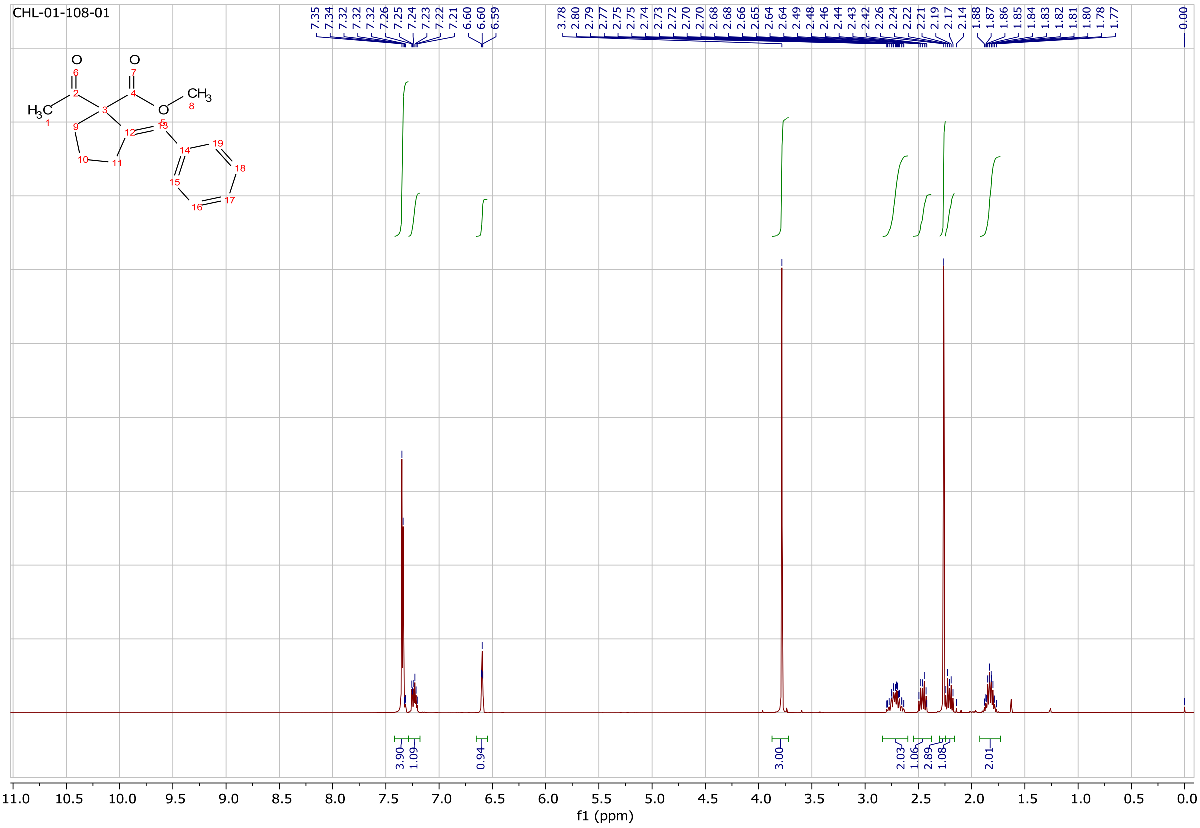

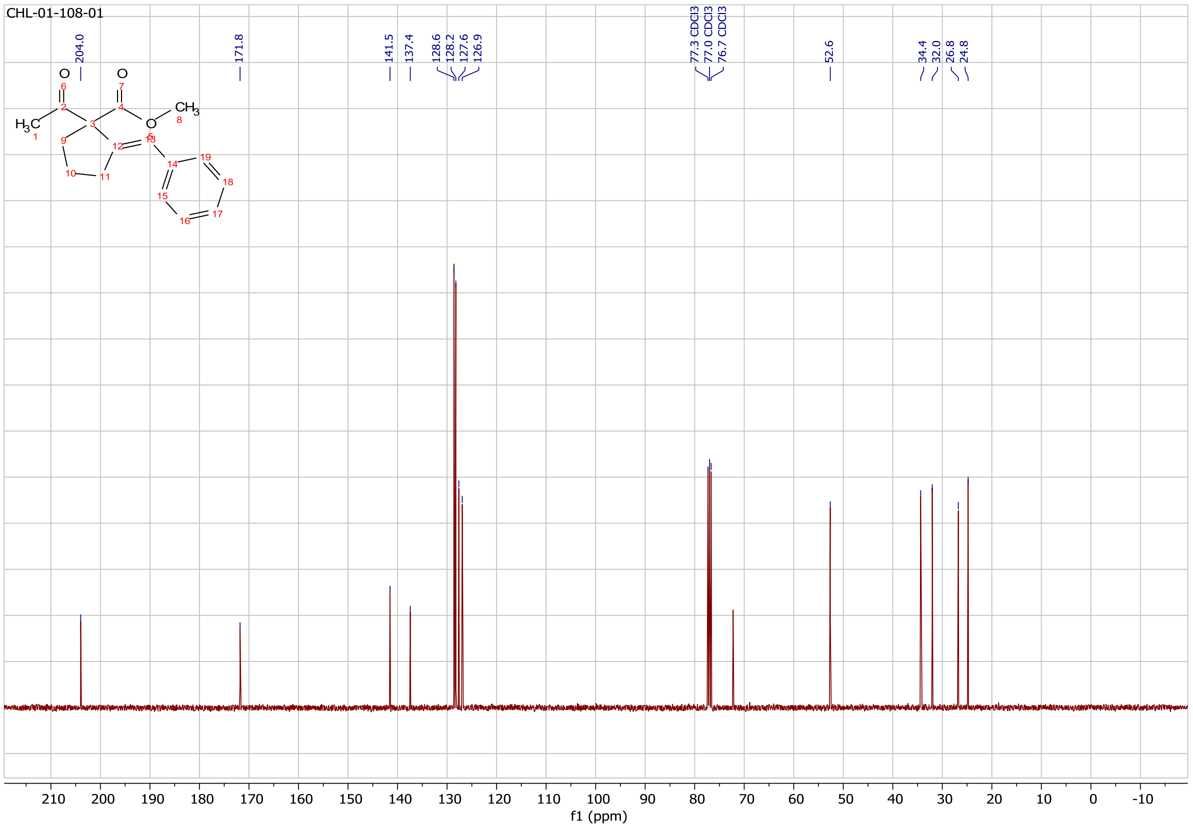

***(E)*-methyl 1-acetyl-2-(4-methoxybenzylidene)cyclopentanecarboxylate (41).**

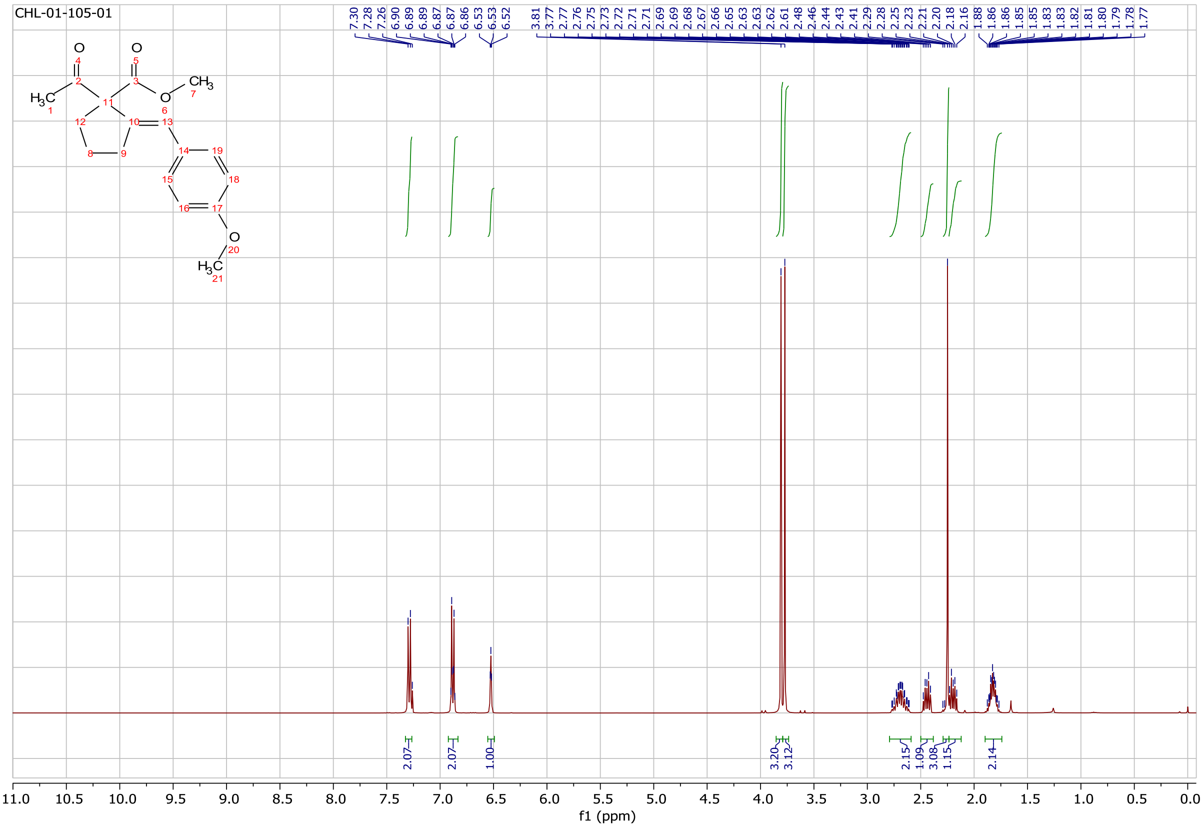

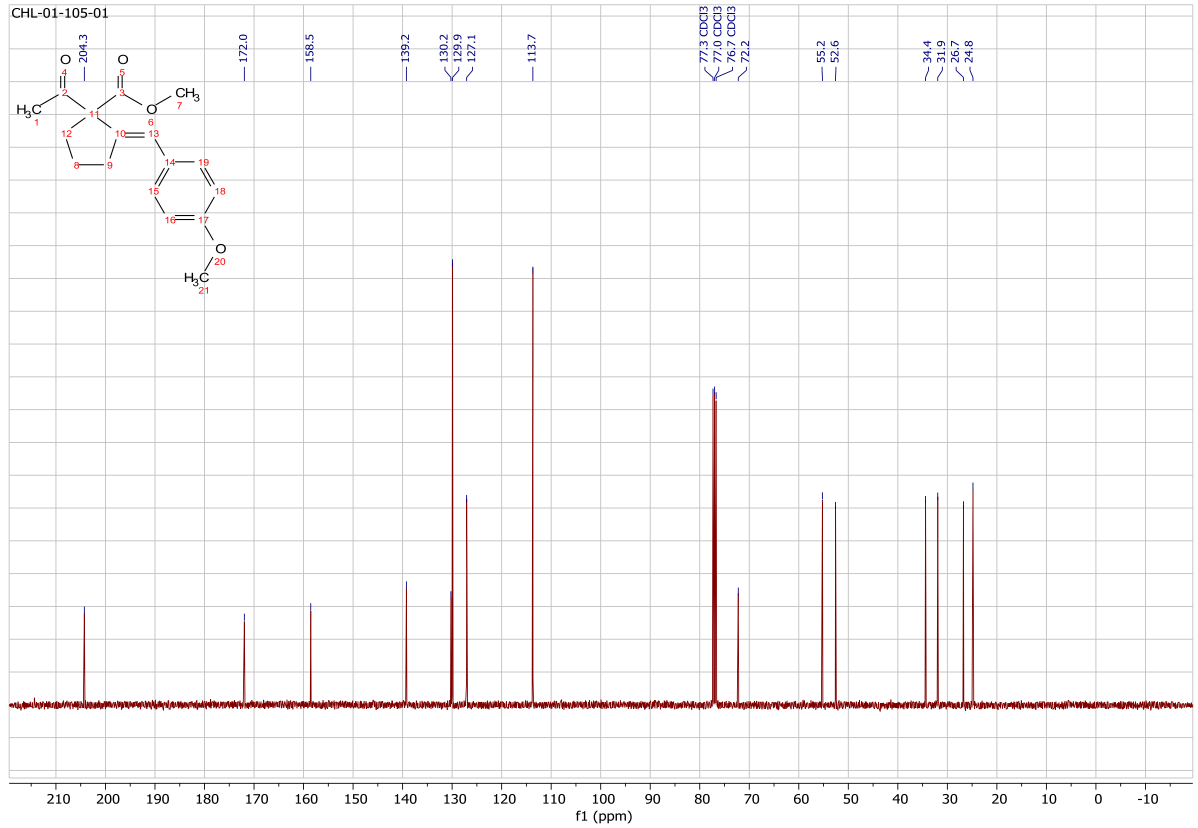

(*E*)-methyl 1-acetyl-2-(4-cyanobenzylidene)cyclopentanecarboxylate (42).

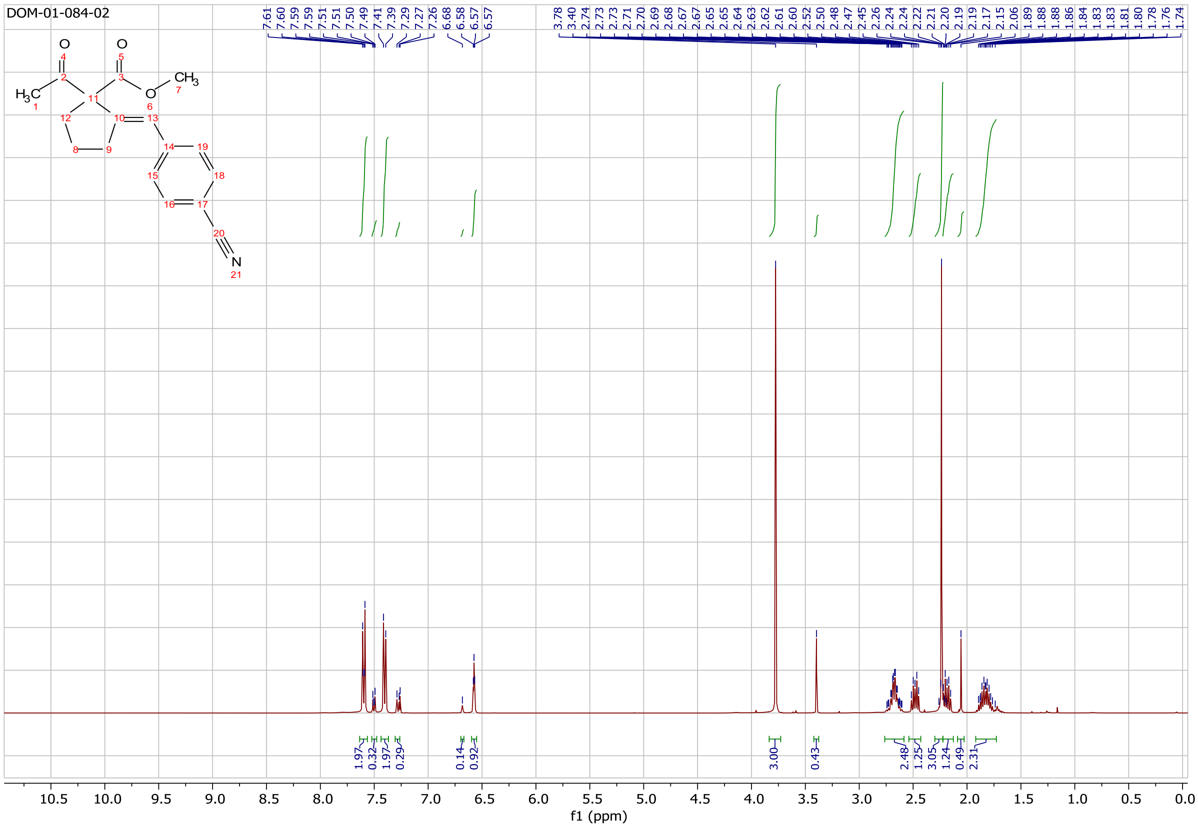

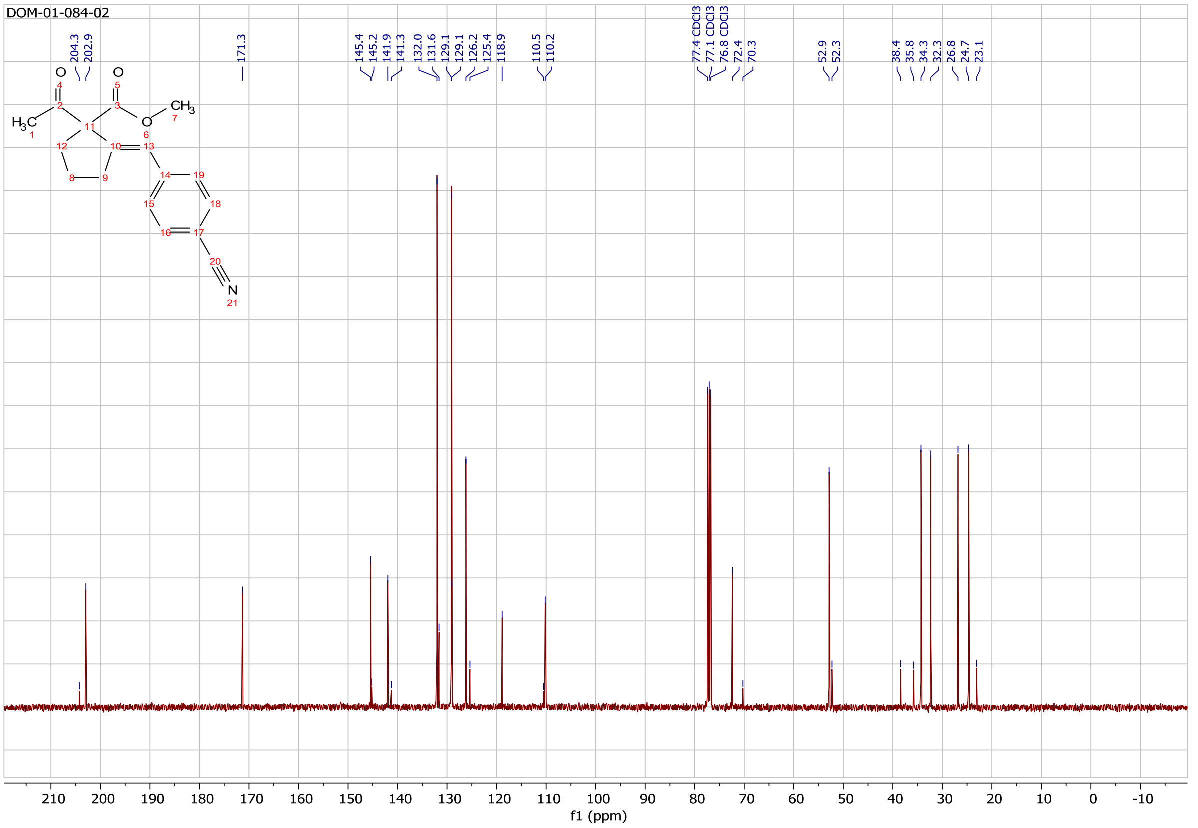

1-((2*E*)-1-benzoyl-2-benzylidenecyclopentyl)ethanone (43).

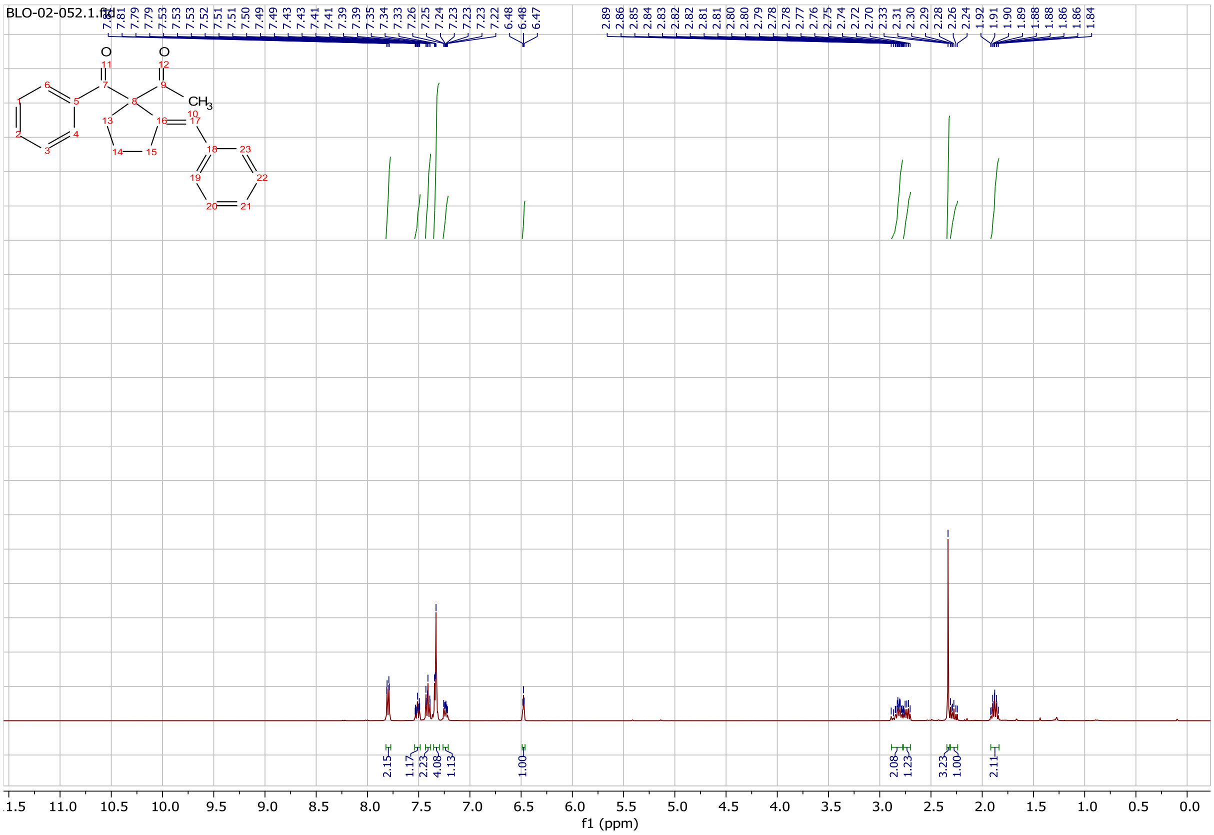

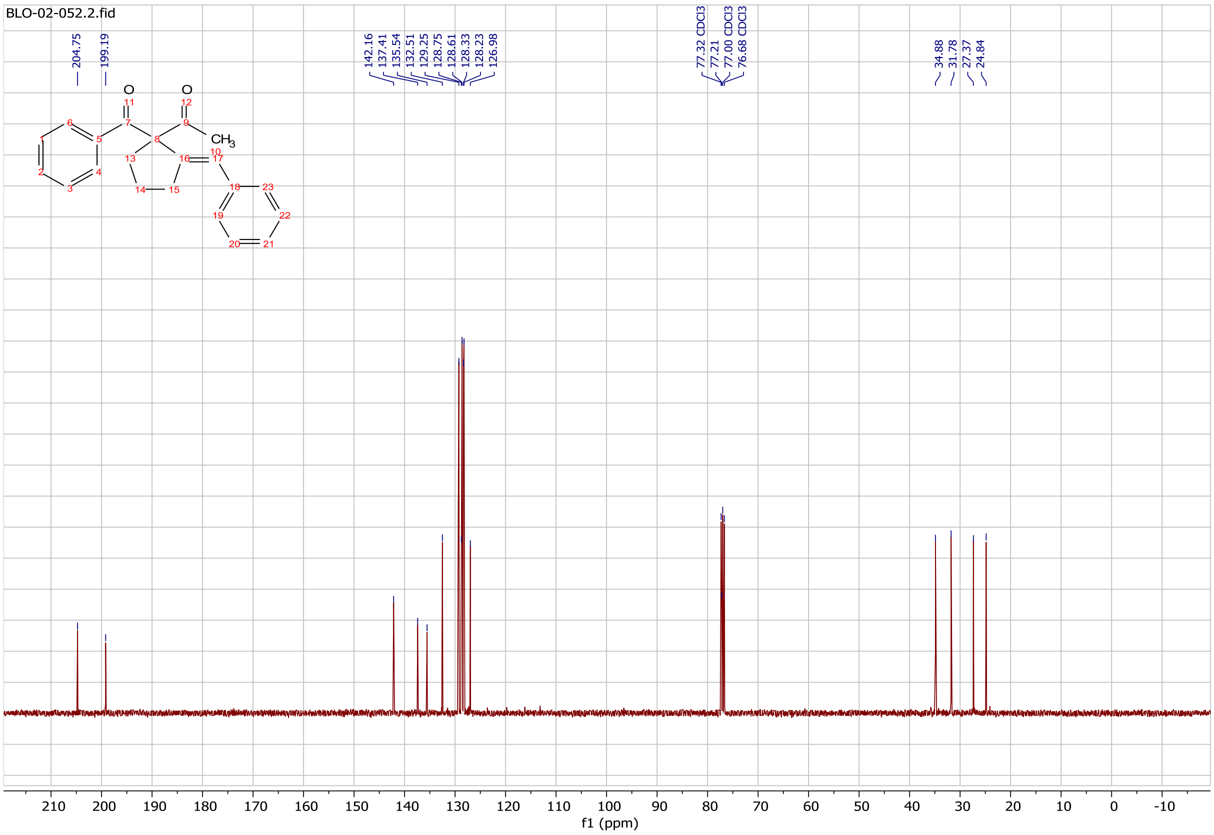

1-((2*E*)-1-benzoyl-2-(4-methoxybenzylidene)cyclopentyl)ethanone (44).

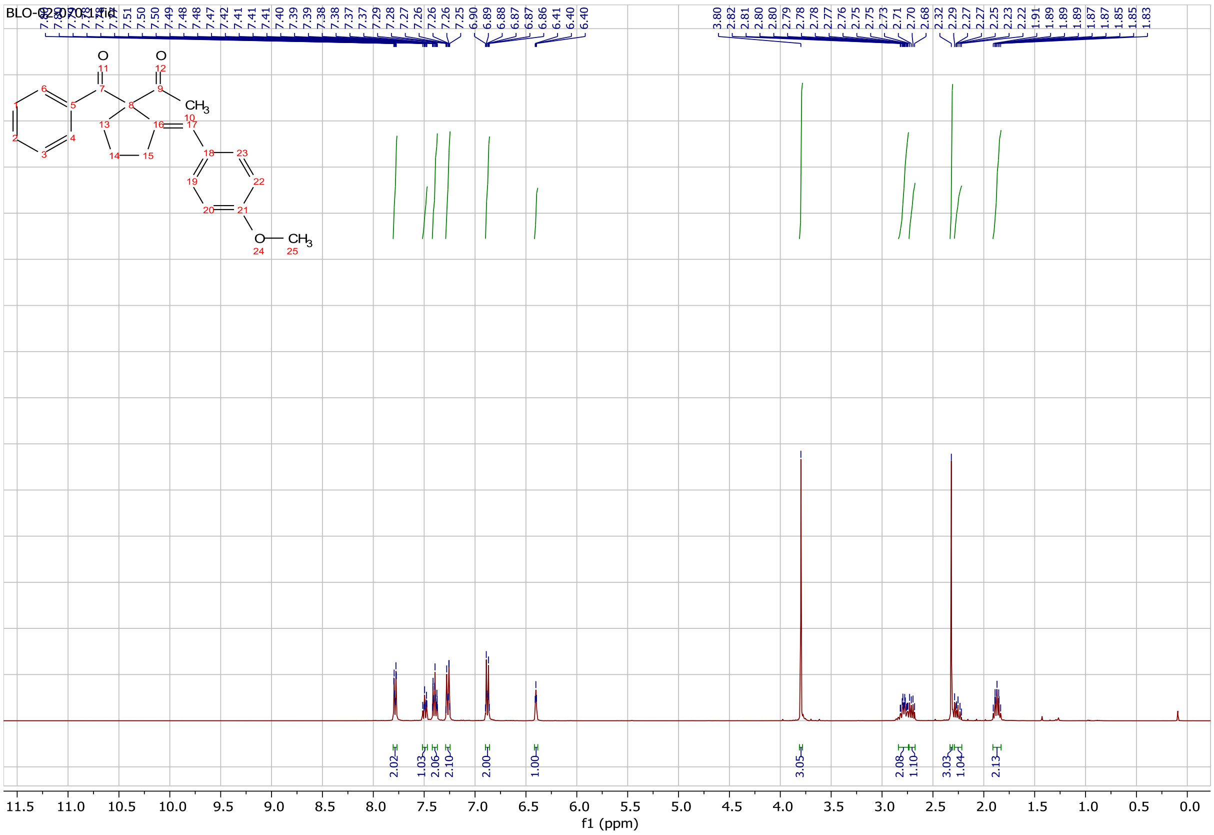

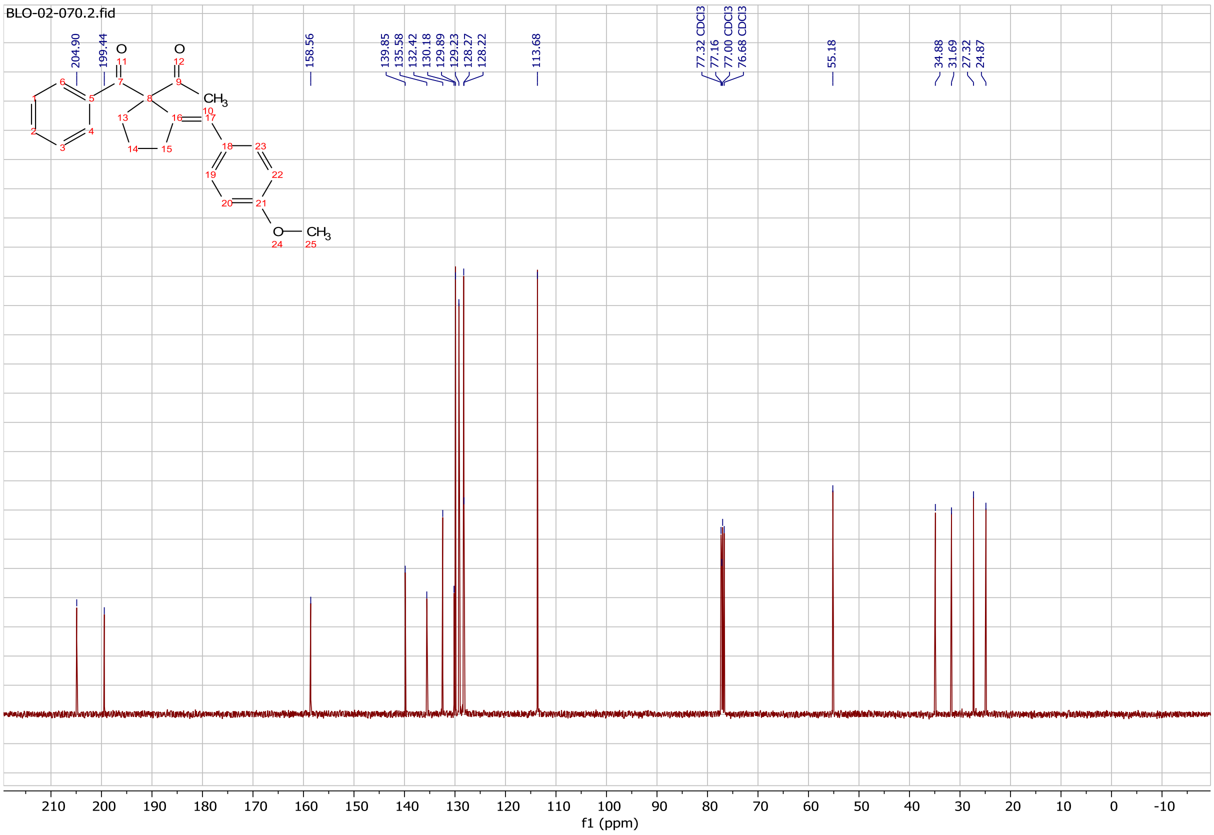

4-(((1*E*)-2-acetyl-2-benzoylcyclopentylidene)methyl)benzonitrile (45).

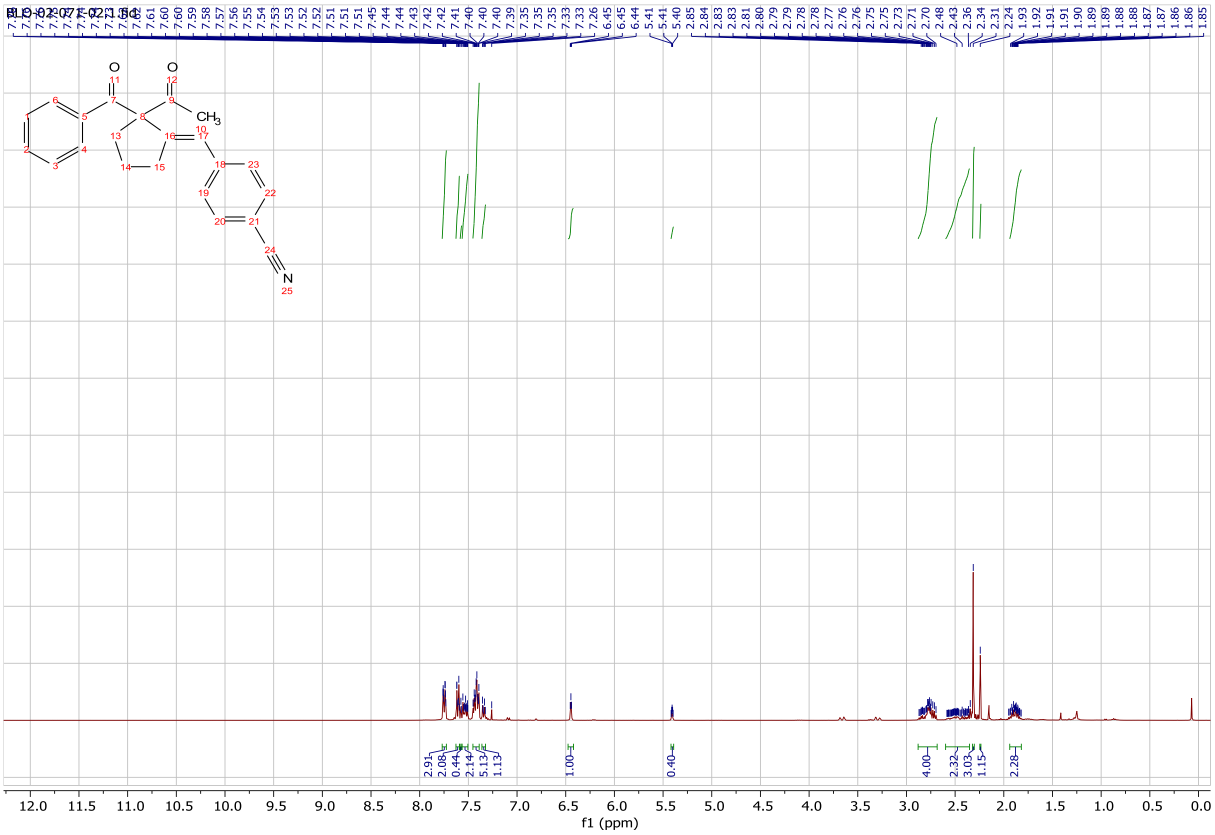

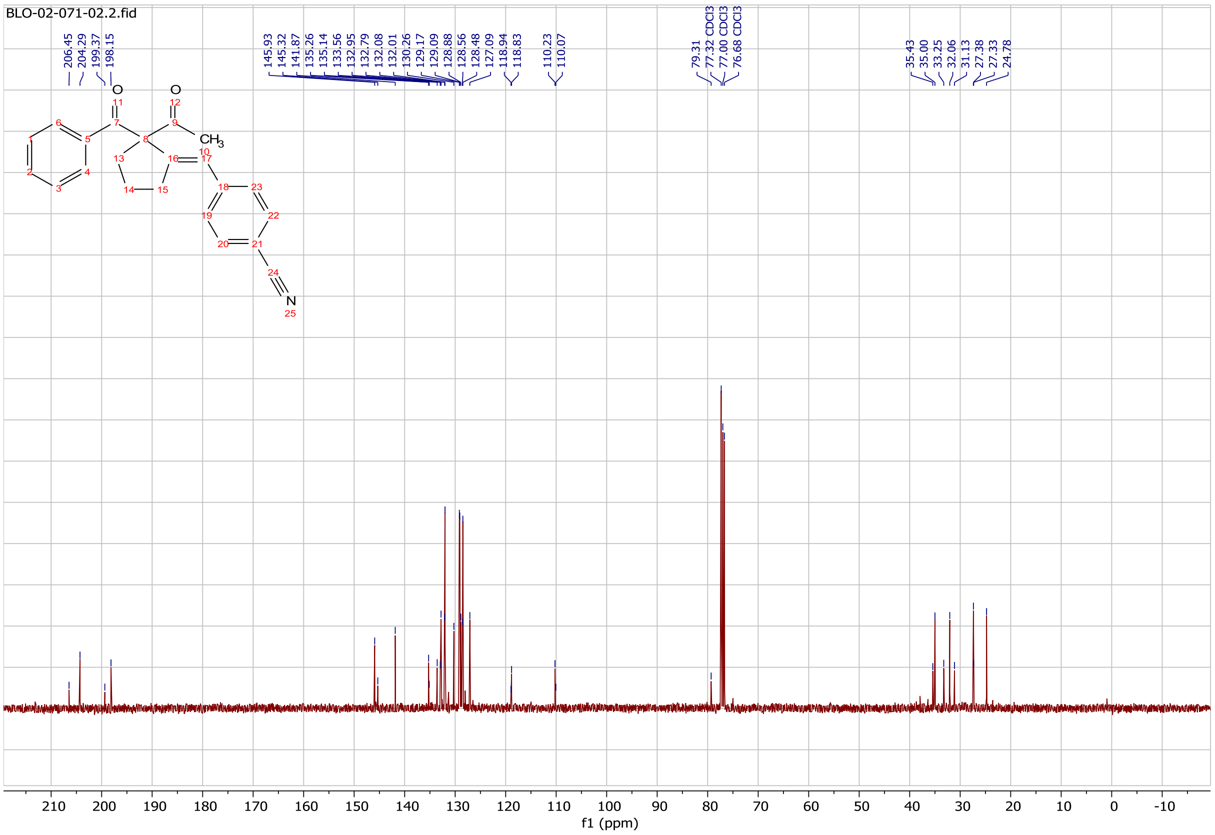

(2*E*)-1-benzoyl-2-benzylidenecyclopentyl)(phenyl)methanone (46).

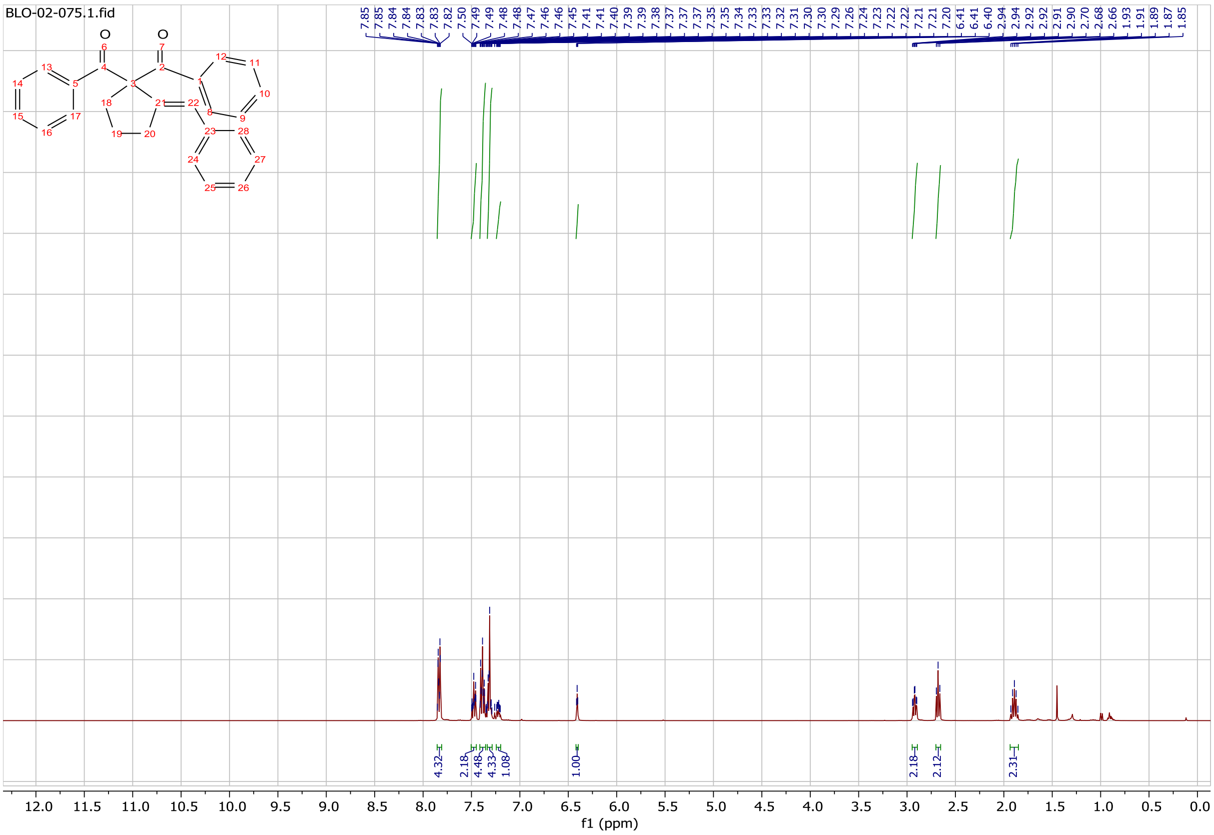

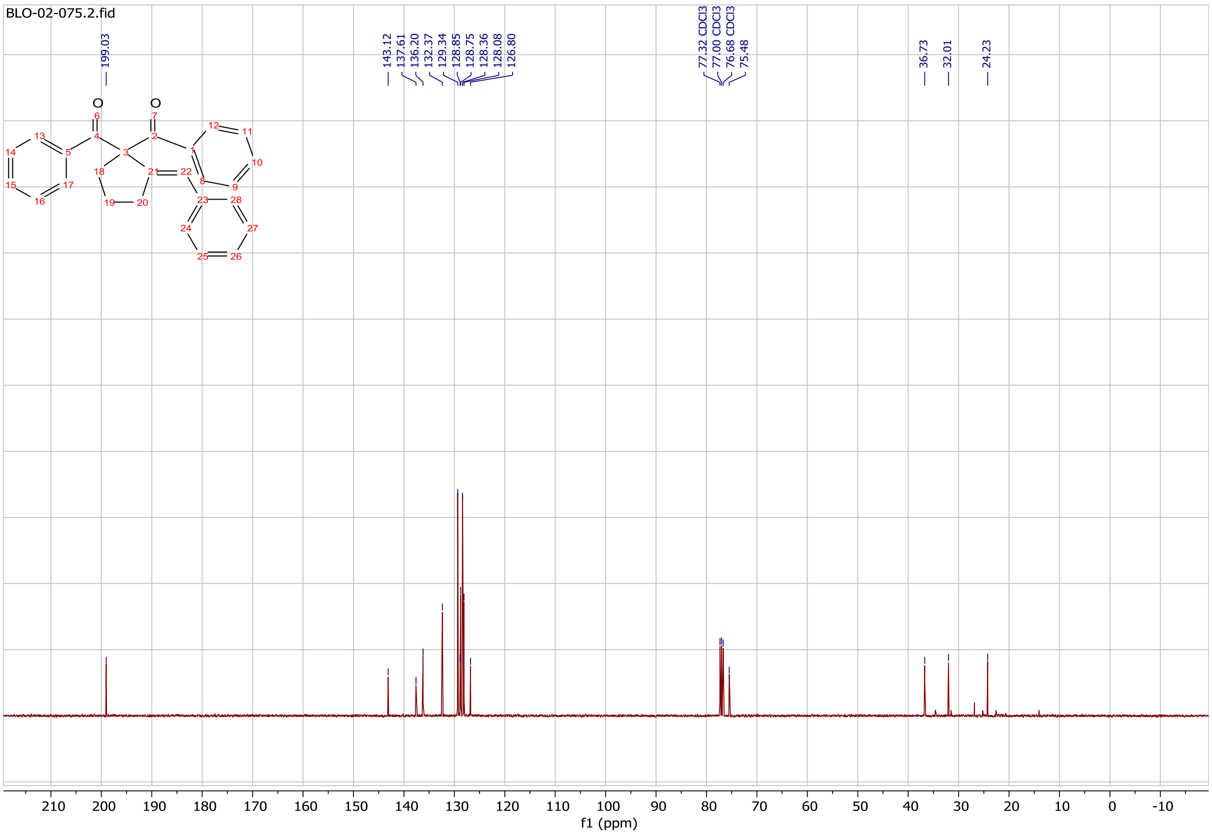

((2*E*)-1-benzoyl-2-(4-methoxybenzylidene)cyclopentyl)(phenyl)methanone (47).

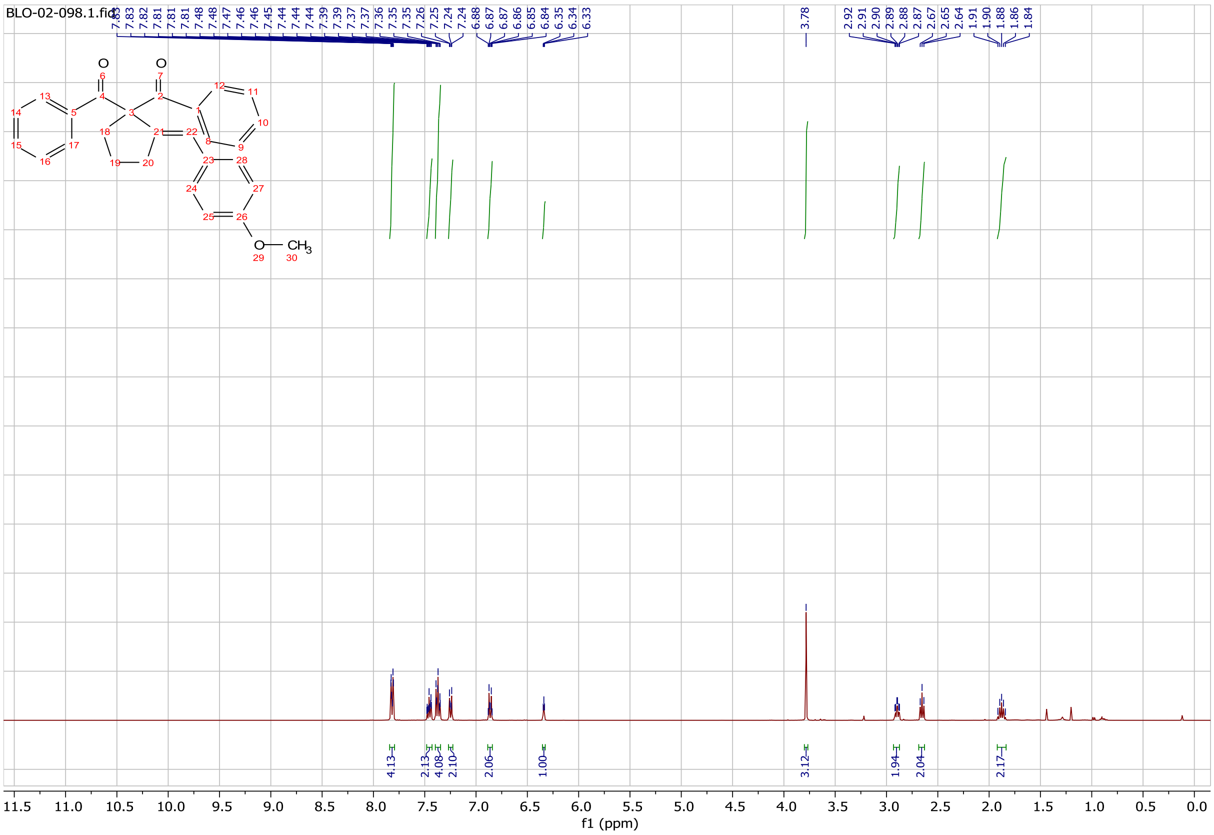

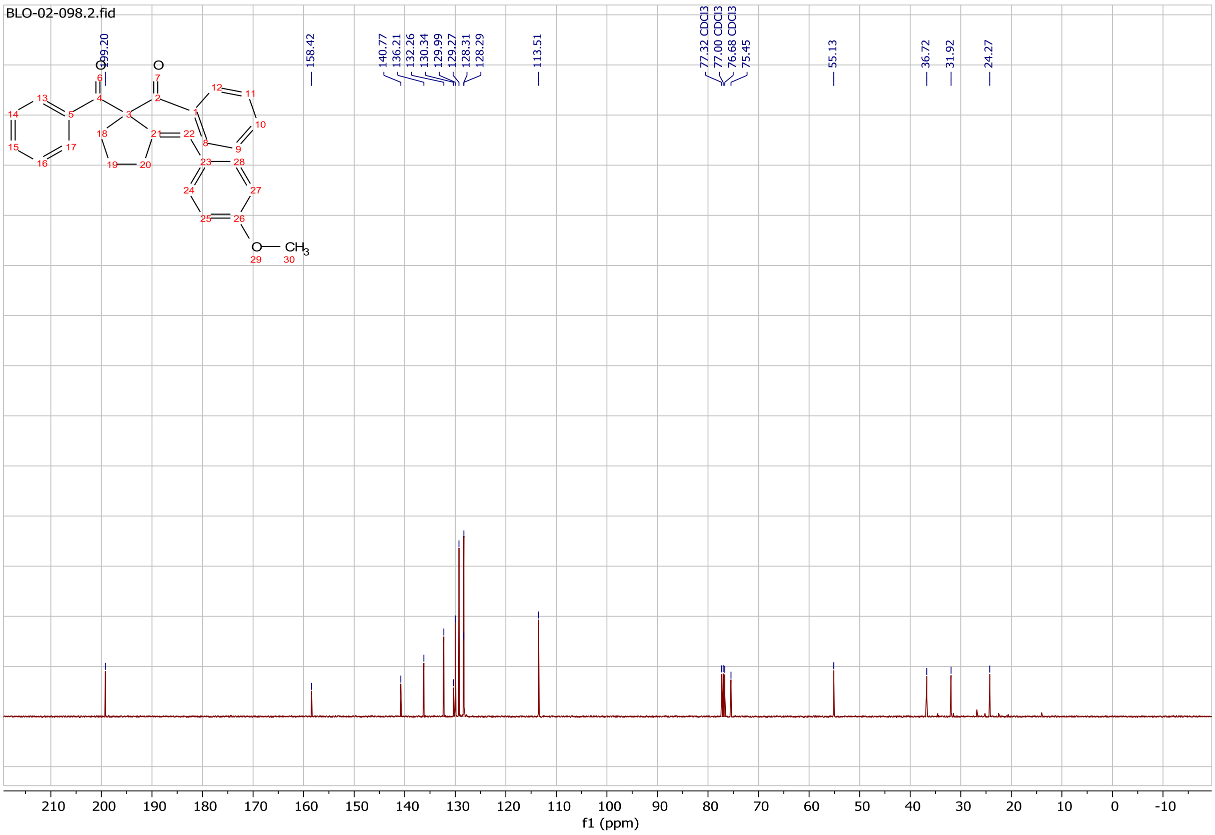

4-(((1*E*)-2,2-dibenzoylcyclopentylidene)methyl)benzonitrile (48).

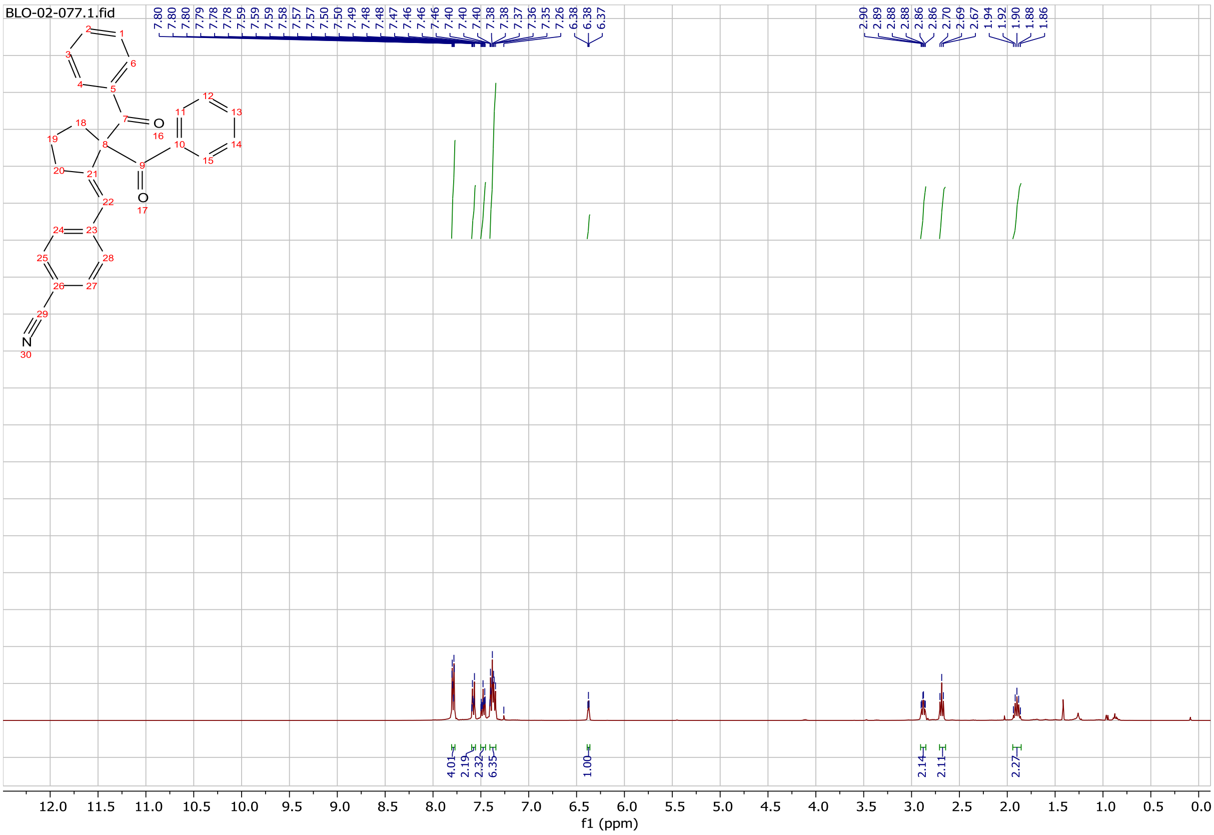

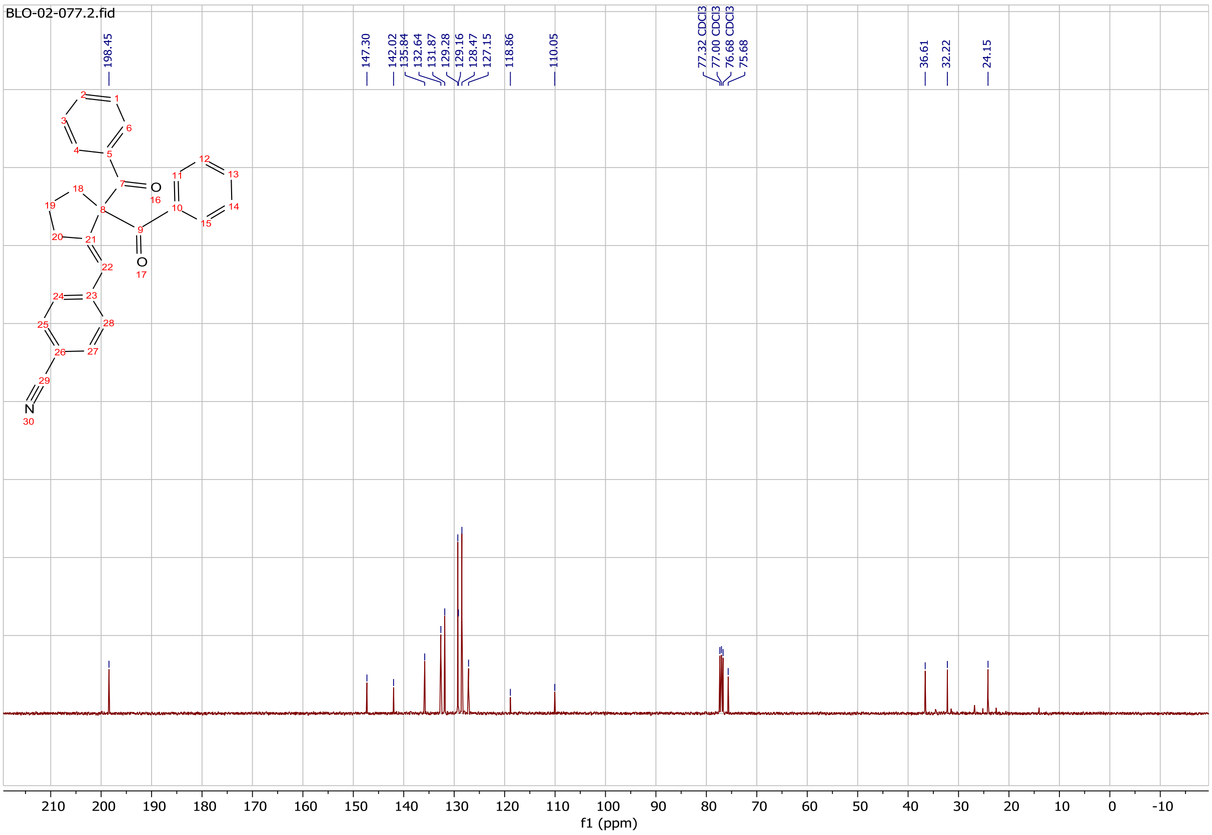

Ethyl (2*E*)-2-benzylidene-1-(diethylphosphono)cyclopentanecarboxylate (49).

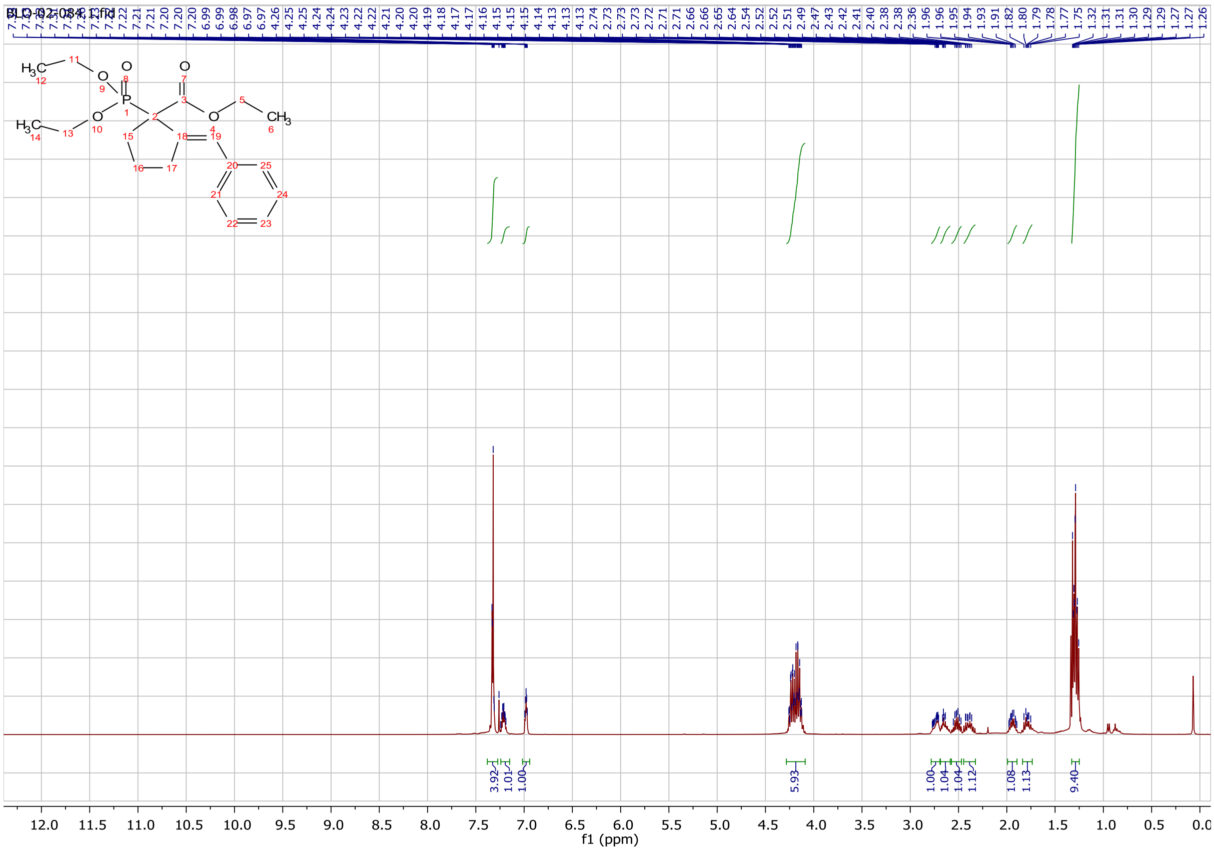

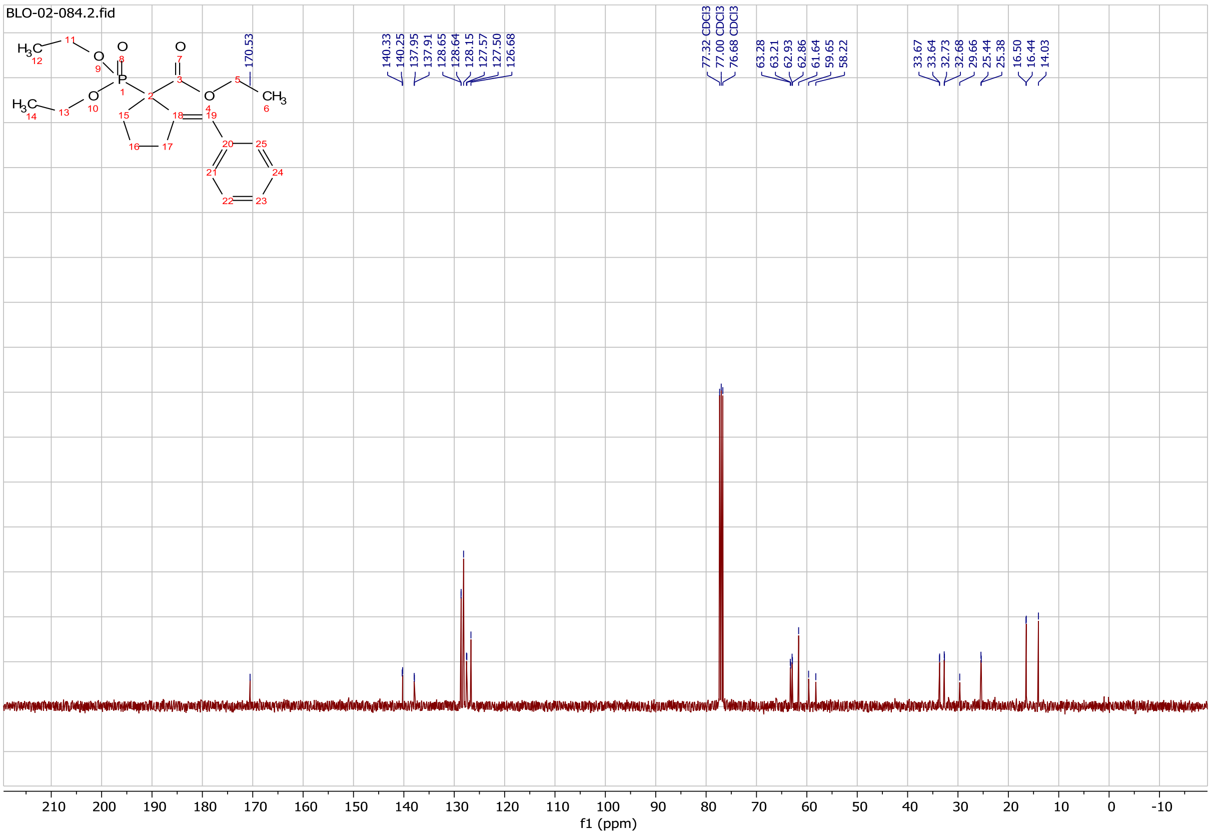

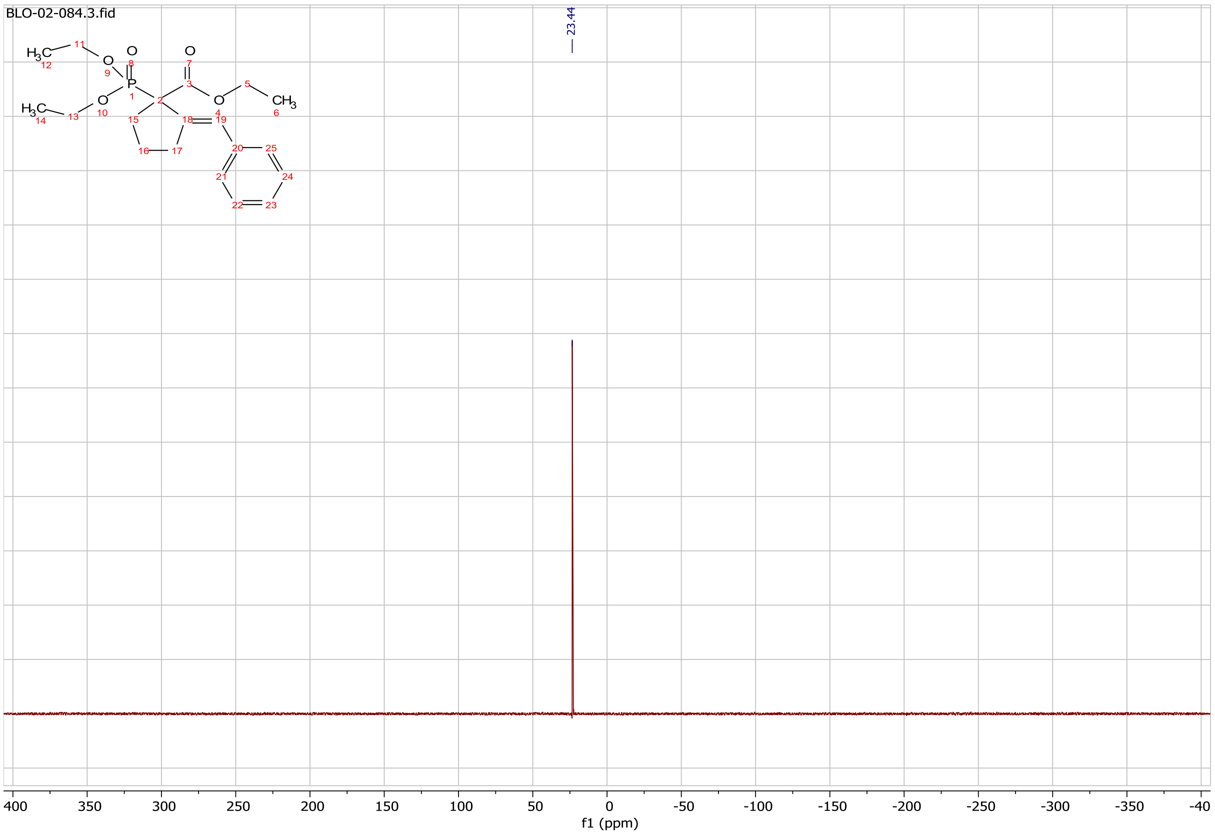
BLO-02-084.3.fid
23.44
f1 (ppm)

Dipropan-2-yl ((2*E*)-2-benzylidene-1-cyanocyclopentyl)phosphonate (50).

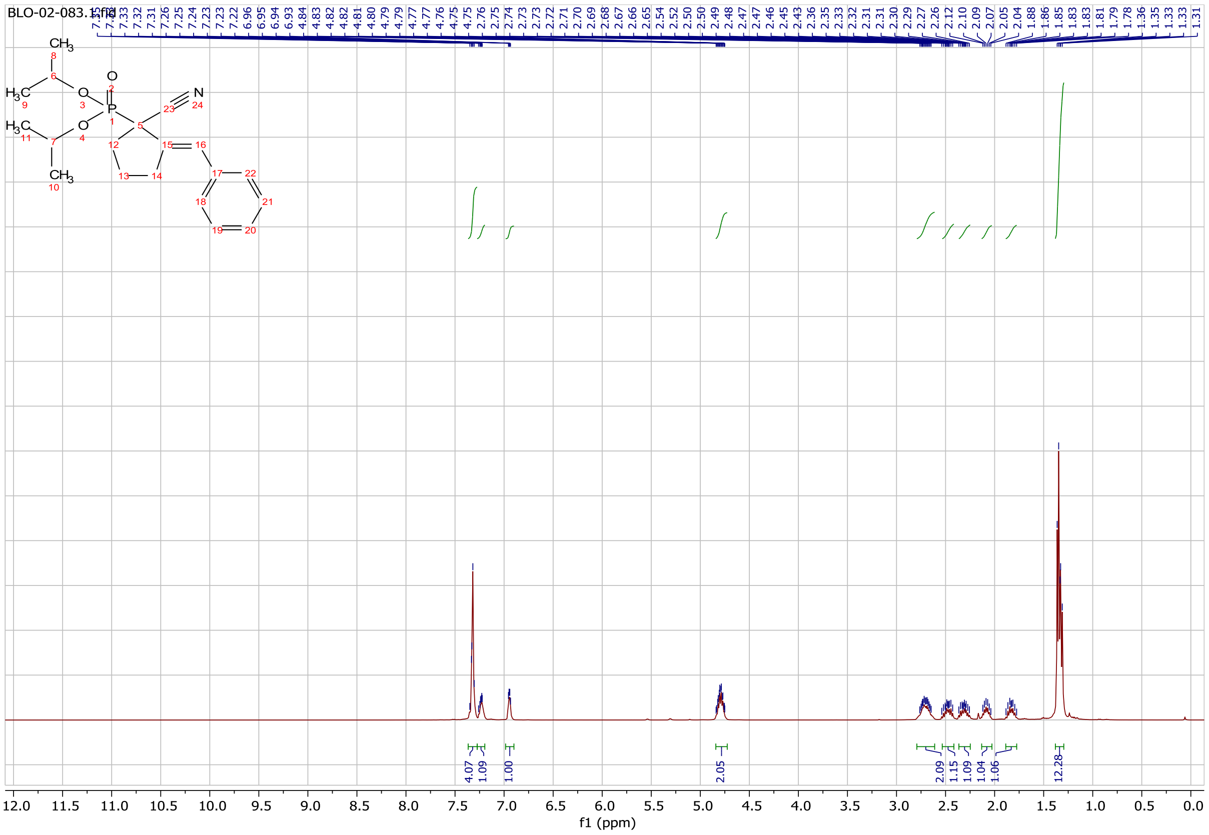

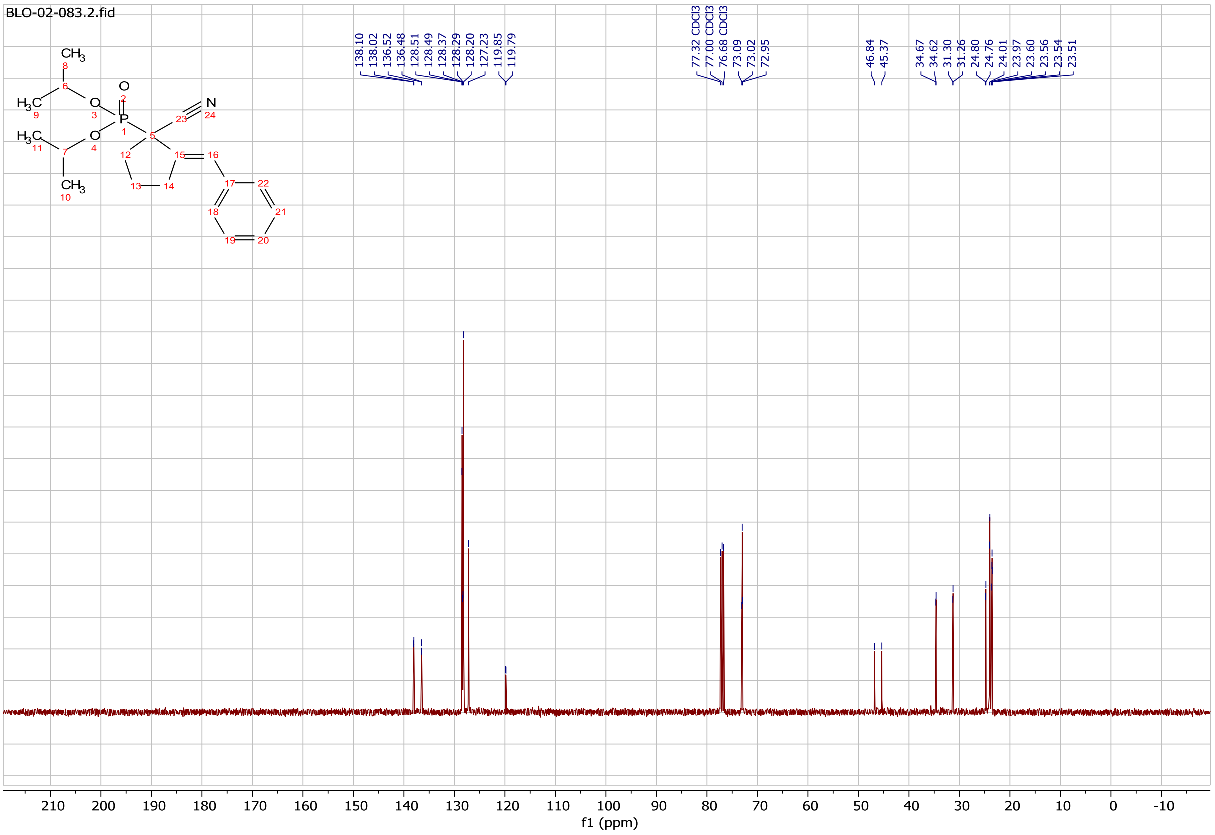

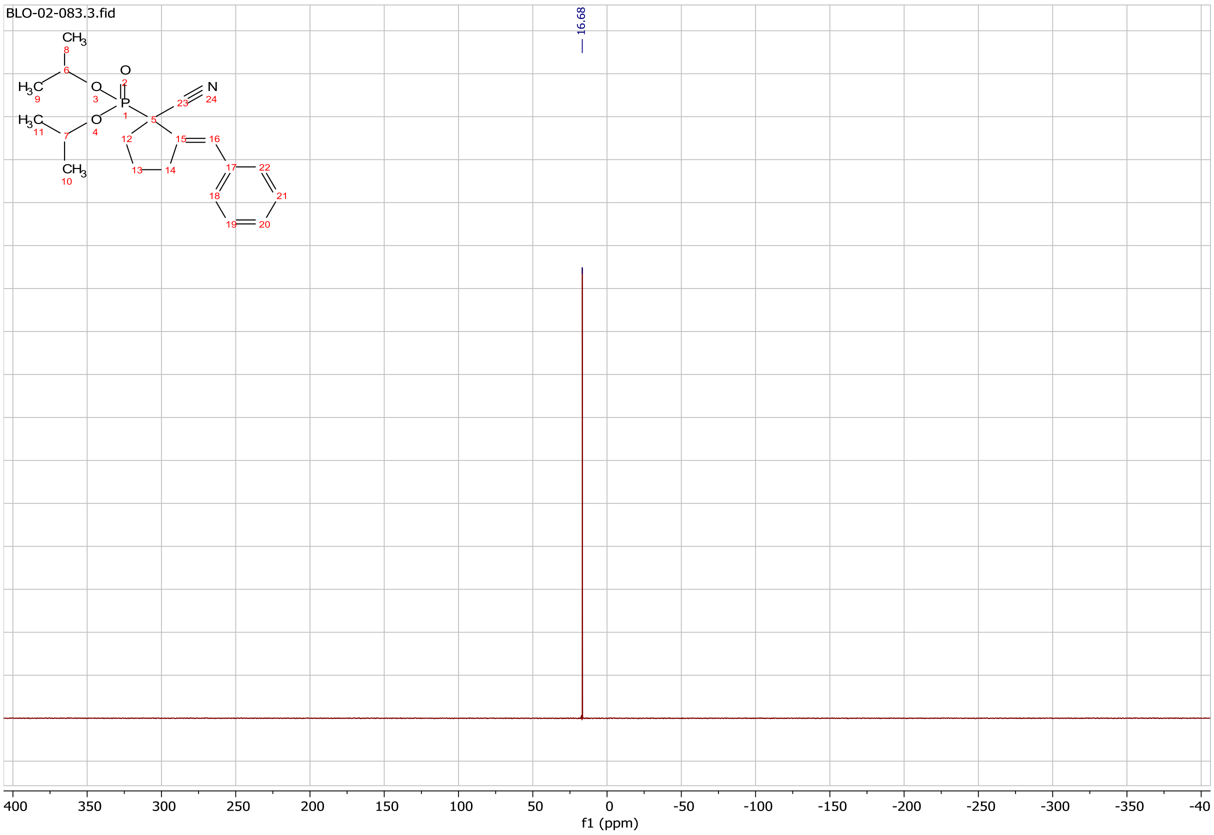
BLO-02-083.3.fid
16.68
400 350 300 250 200 150 100 50 0 -50 -100 -150 -200 -250 -300 -350 -40
f1 (ppm)

Diethyl ((2*E*)-1-acetyl-2-benzylidenecyclopentyl)phosphonate (51).

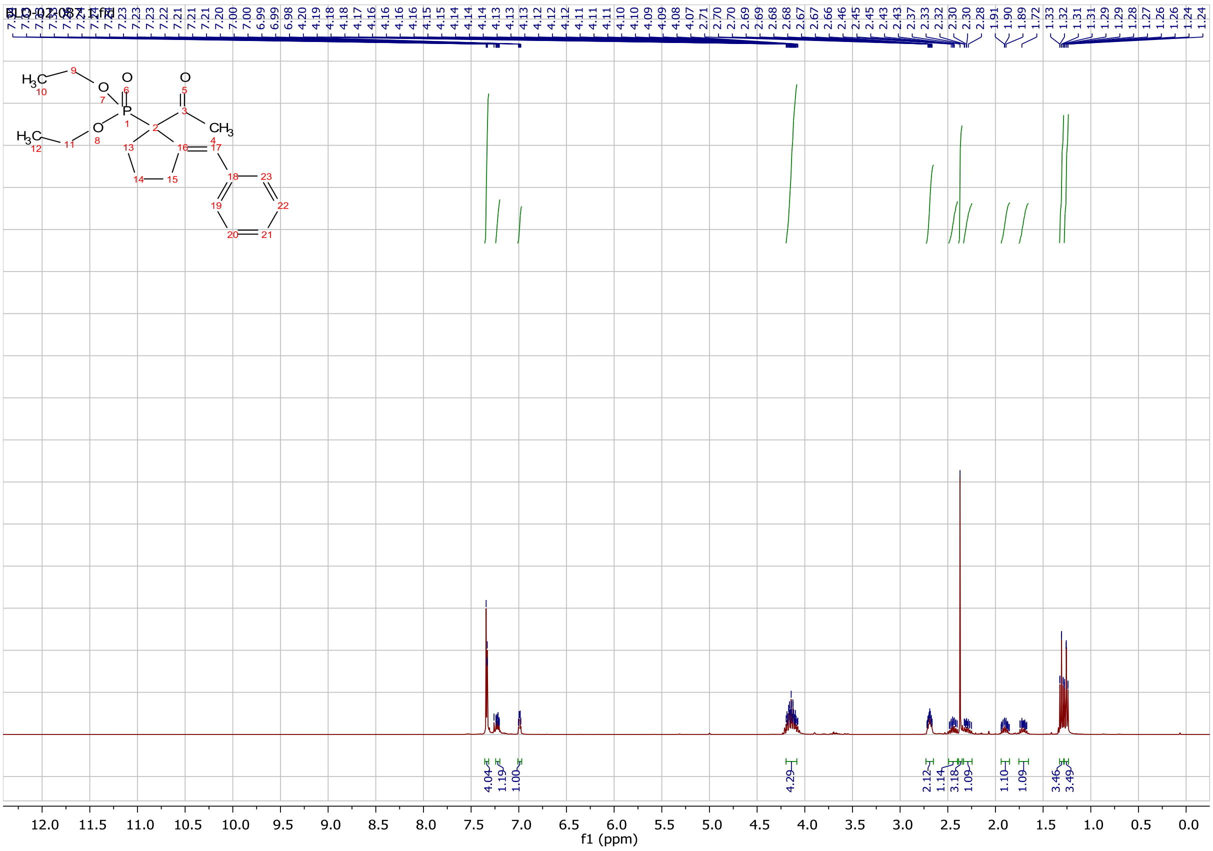

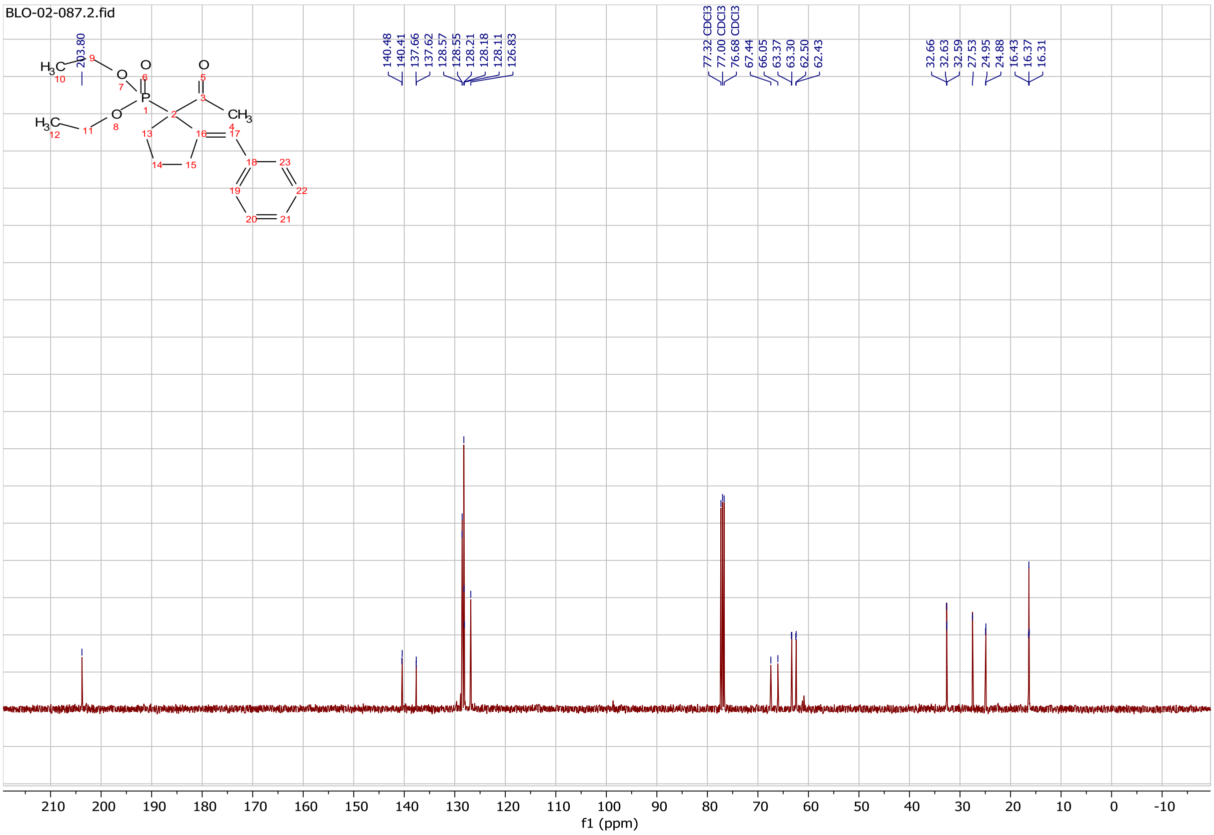

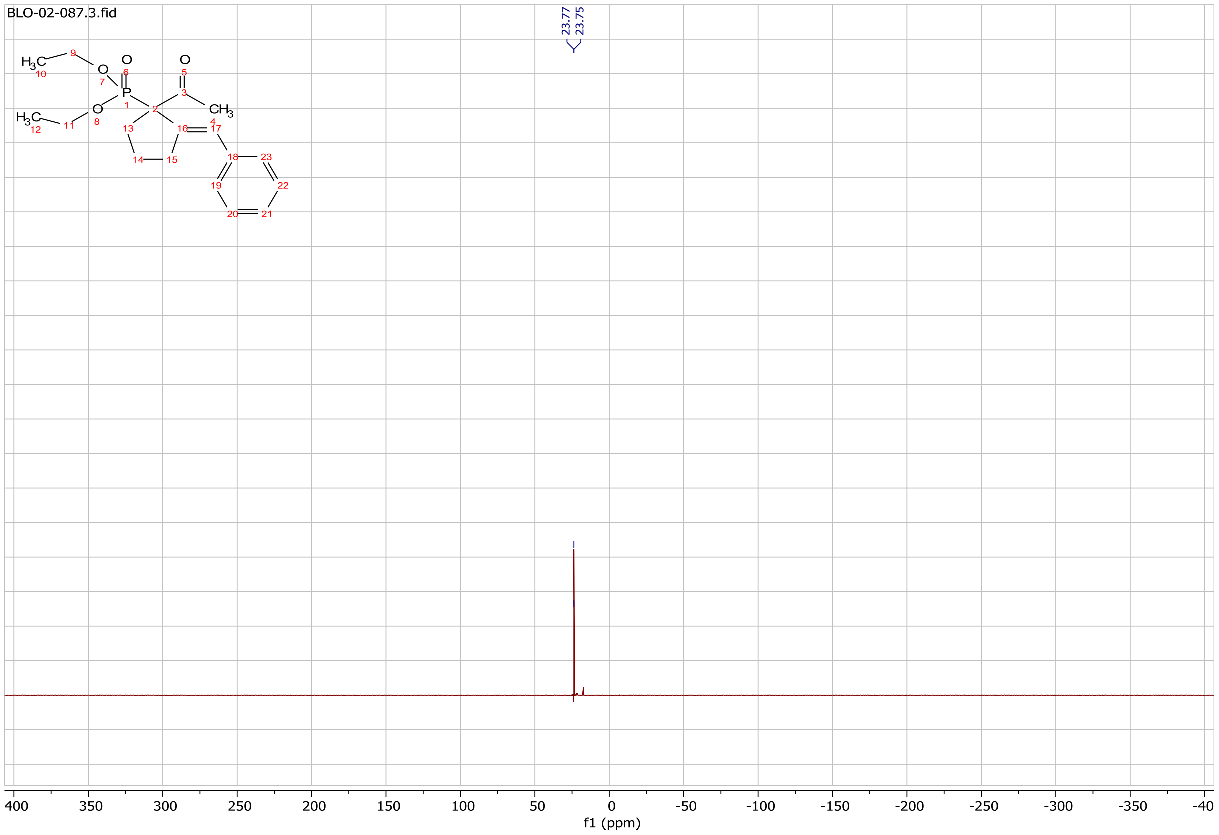
BLO-02-087.3.fid
23.77
23.75
f1 (ppm)

(2*E*)-2-benzylidene-1-(diphenylphosphoryl)cyclopentanecarbonitrile (52).

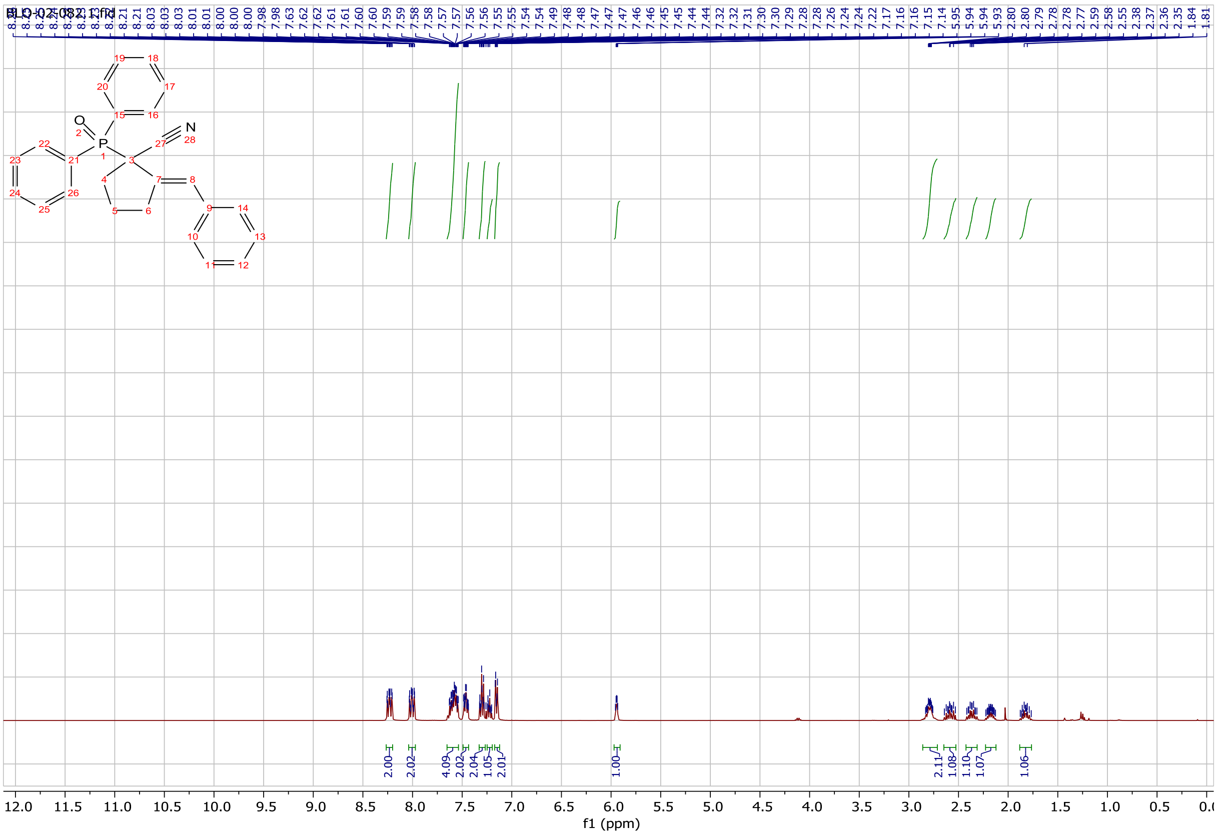

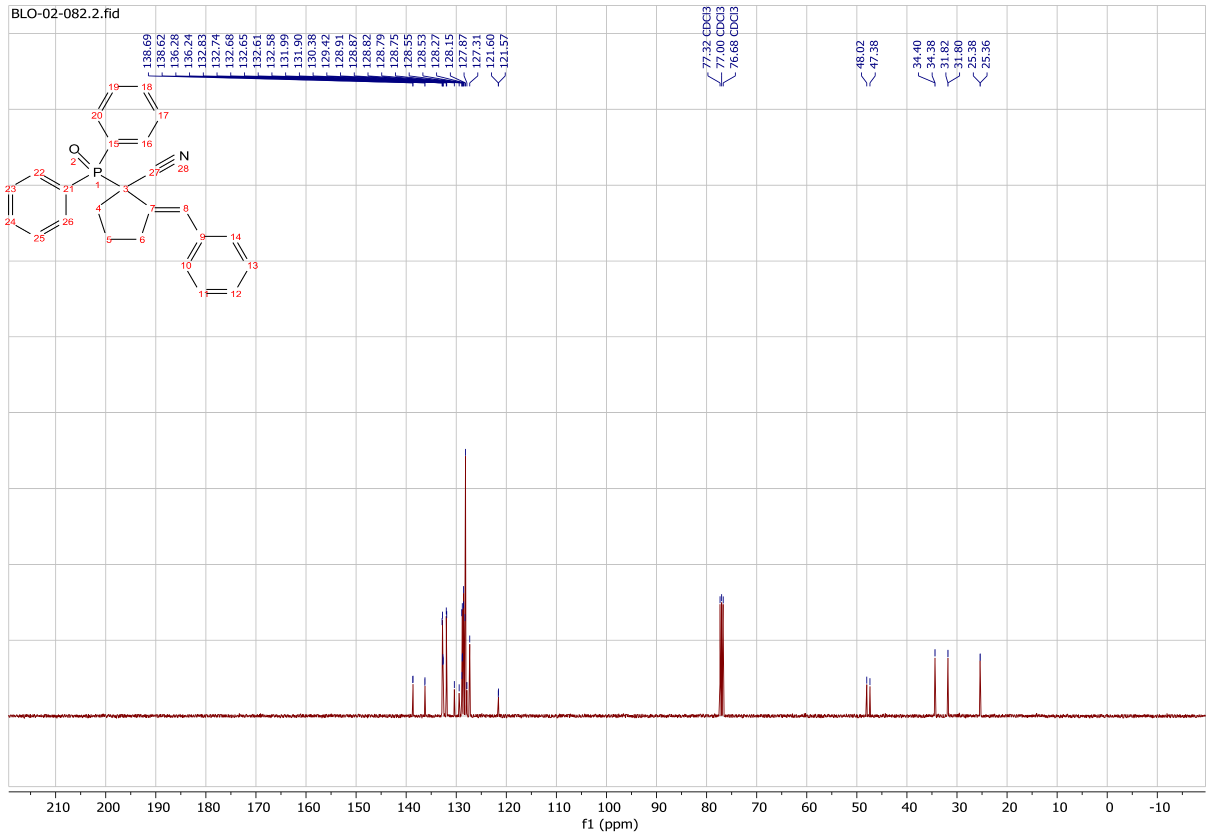
BLO-02-082.2.fid
138.69
138.62
136.28
136.24
132.83
132.74
132.68
132.65
132.61
132.58
131.99
131.90
130.38
129.42
128.91
128.87
128.82
128.79
128.75
128.55
128.53
128.27
128.15
127.87
127.31
121.60
121.57
77.32 CDCl3
77.00 CDCl3
76.68 CDCl3
48.02
47.38
34.40
34.38
31.82
31.80
25.38
25.36
f1 (ppm)

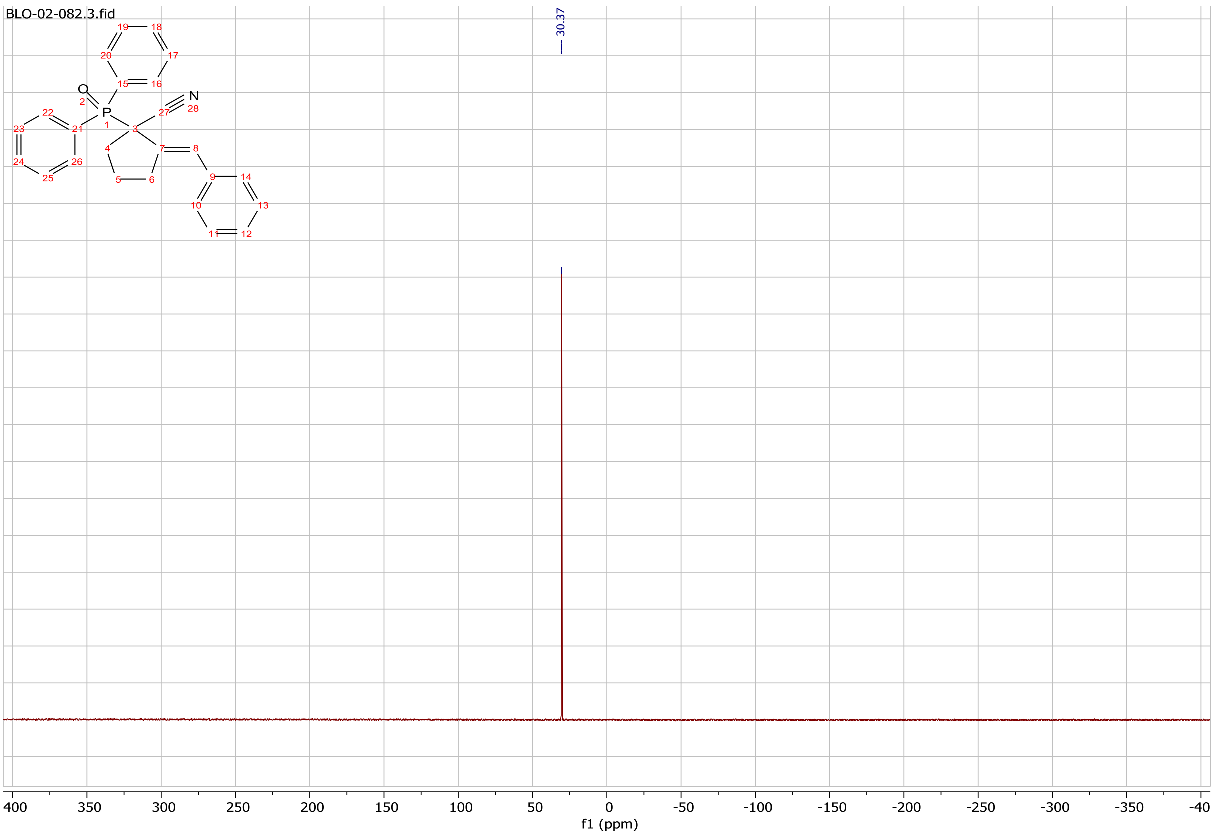
BLO-02-082.3.fid
30.37
f1 (ppm)

Ethyl (2*E*)-2-benzylidene-1-(diphenylphosphoryl)cyclopentanecarboxylate (53).

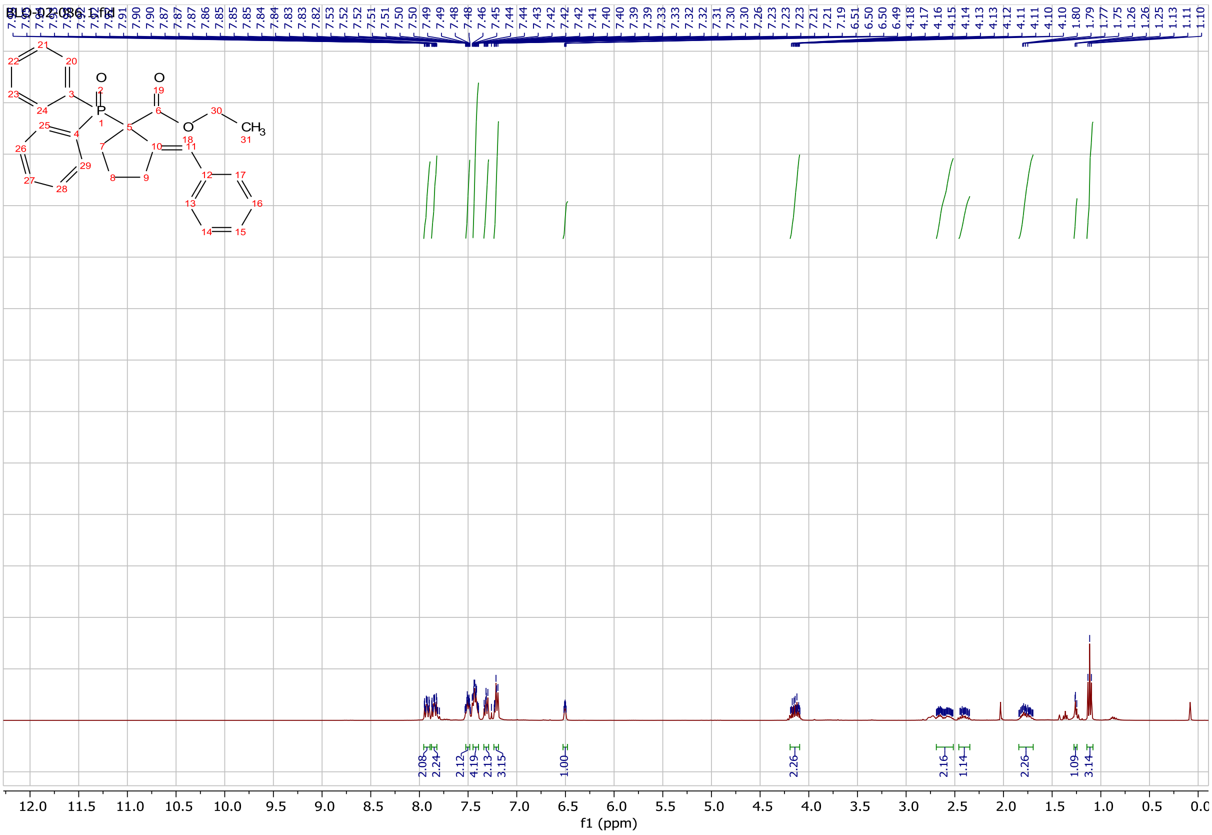

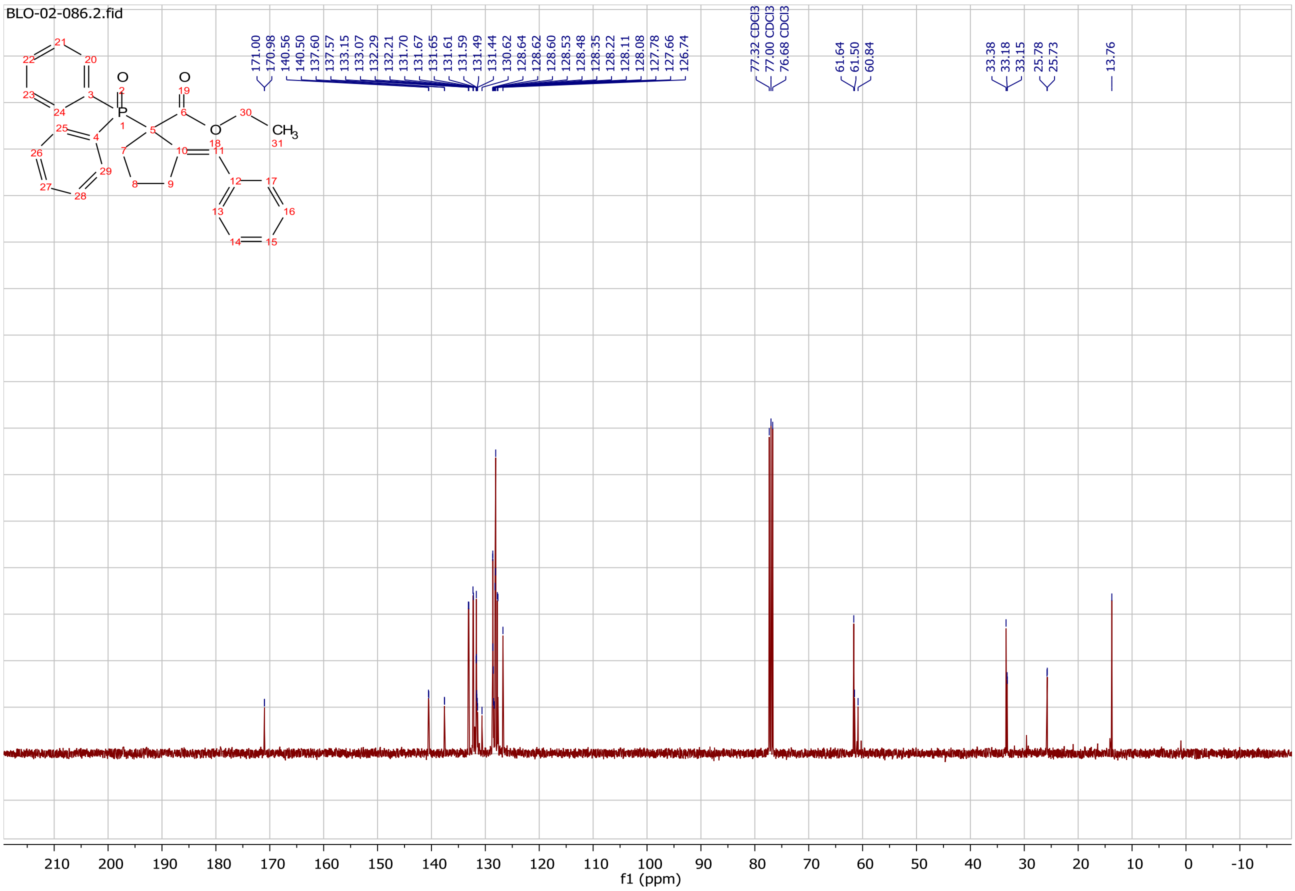

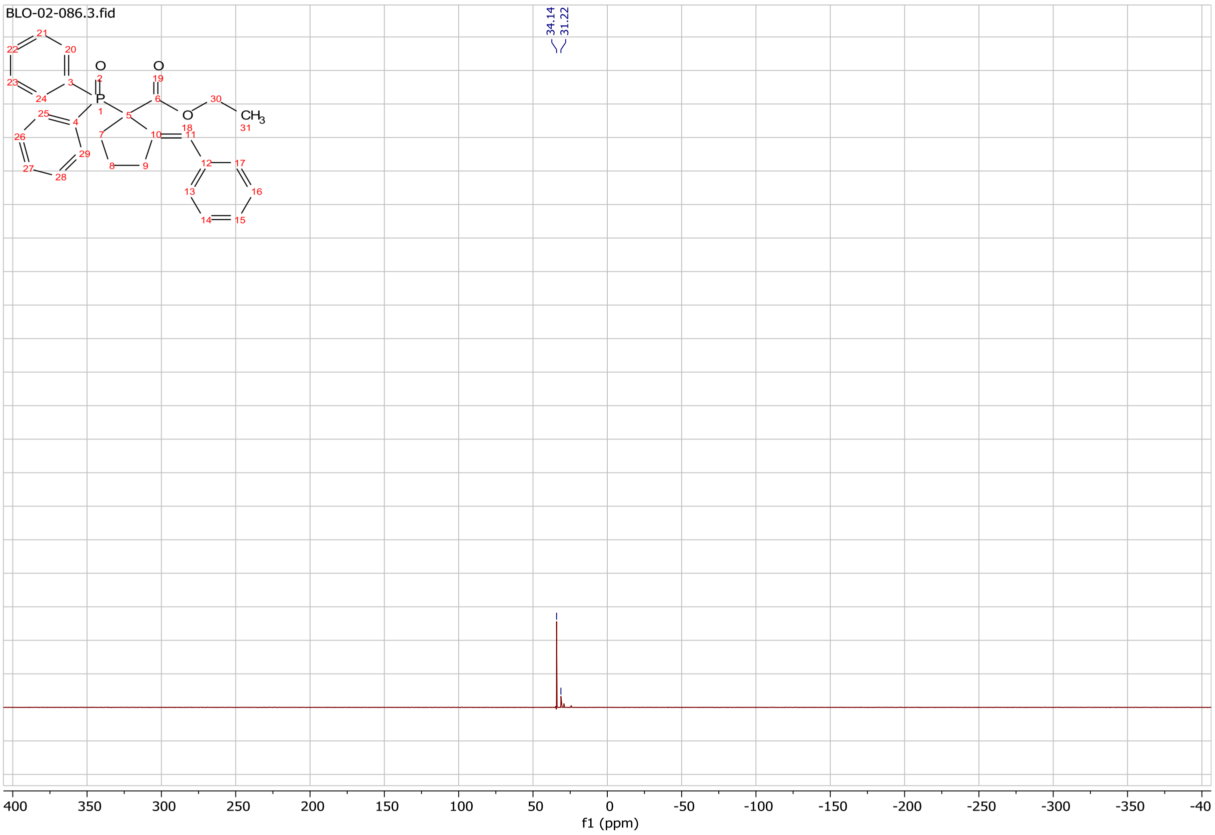
BLO-02-086.3.fid
34.14
31.22
f1 (ppm)

1-((2*E*)-2-benzylidene-1-(diphenylphosphoryl)cyclopentyl)ethanone (54).

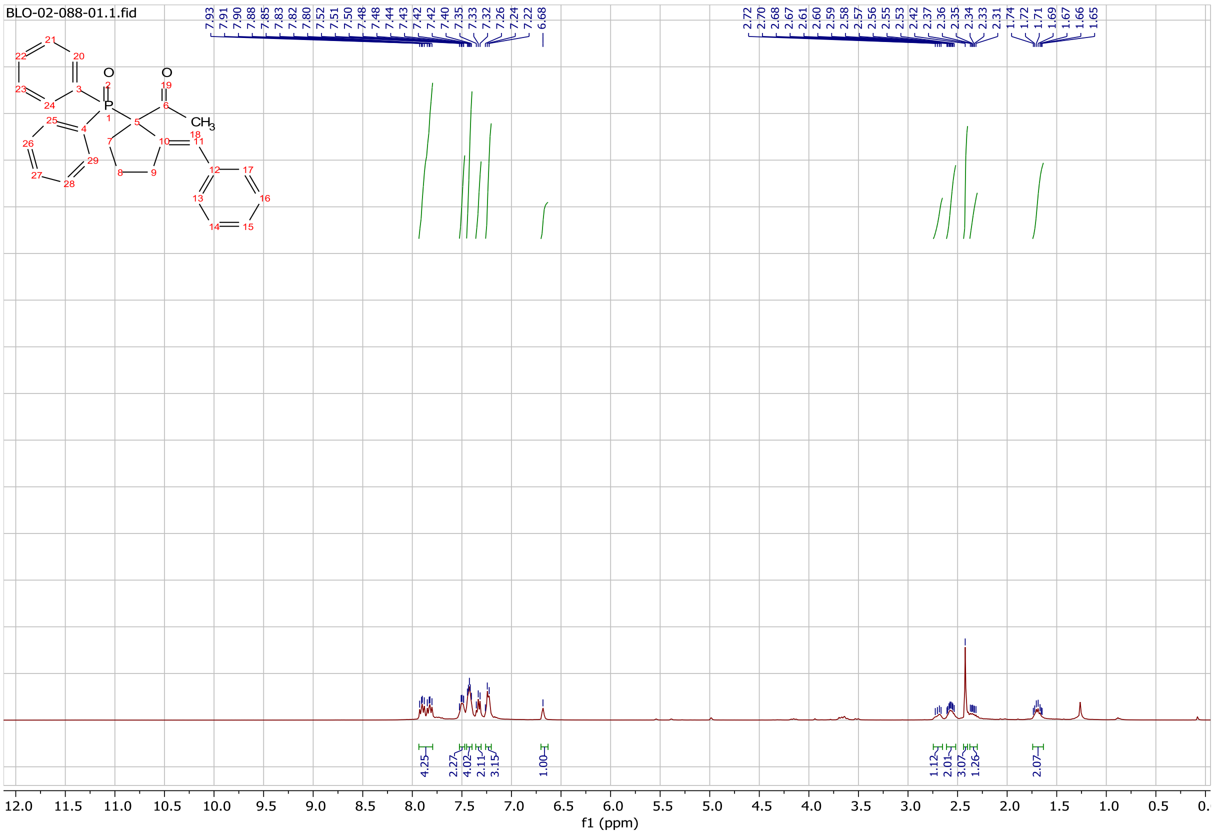

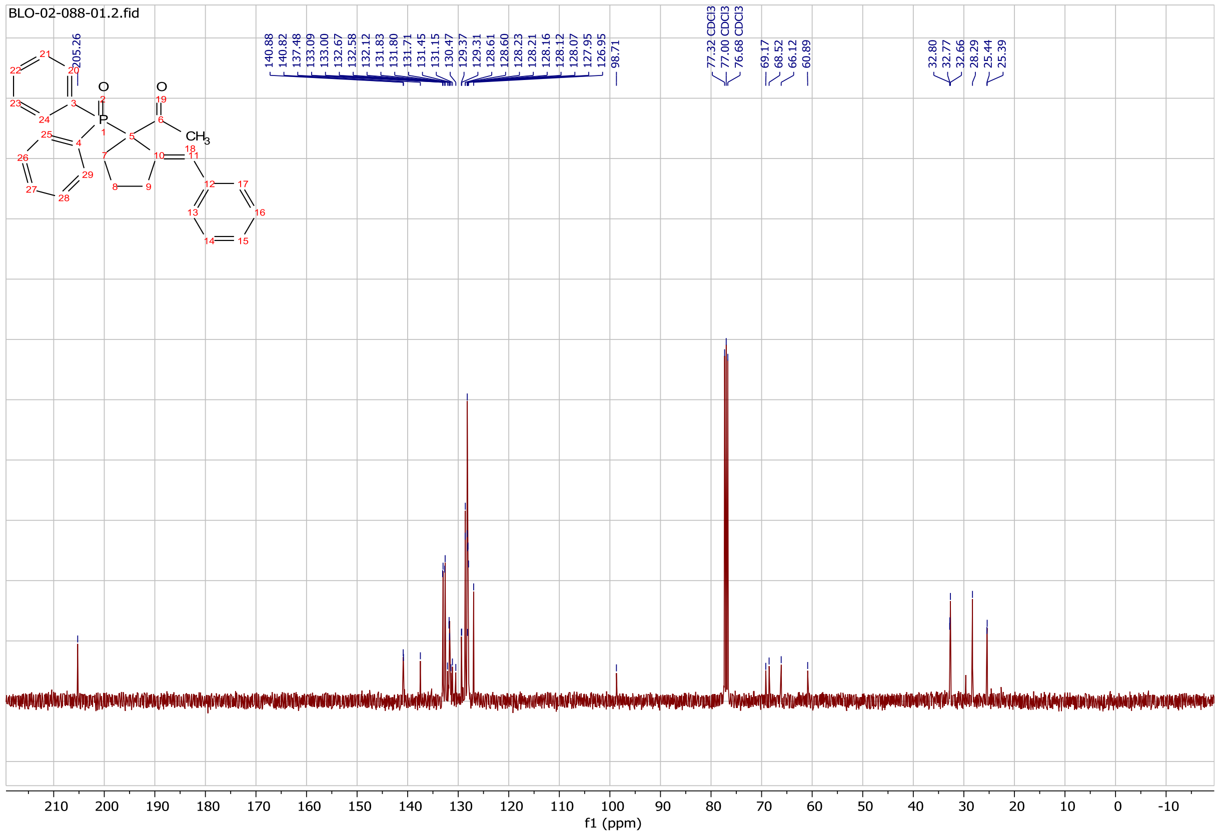

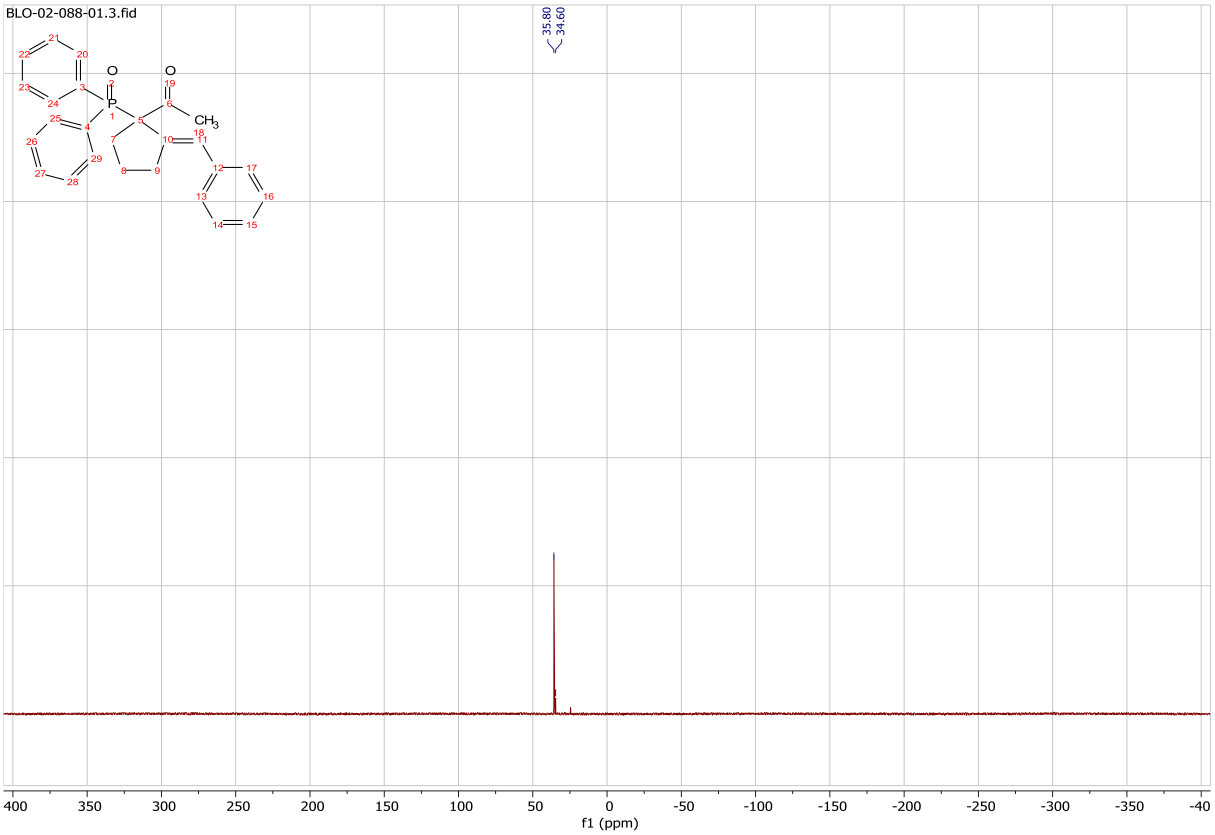

BLO-02-088-01.3.fid
35.80
34.60
f1 (ppm)

Dimethyl (2E)-2-(4-fluorobenzylidene)cyclopentane-1,1-dicarboxylate (55).

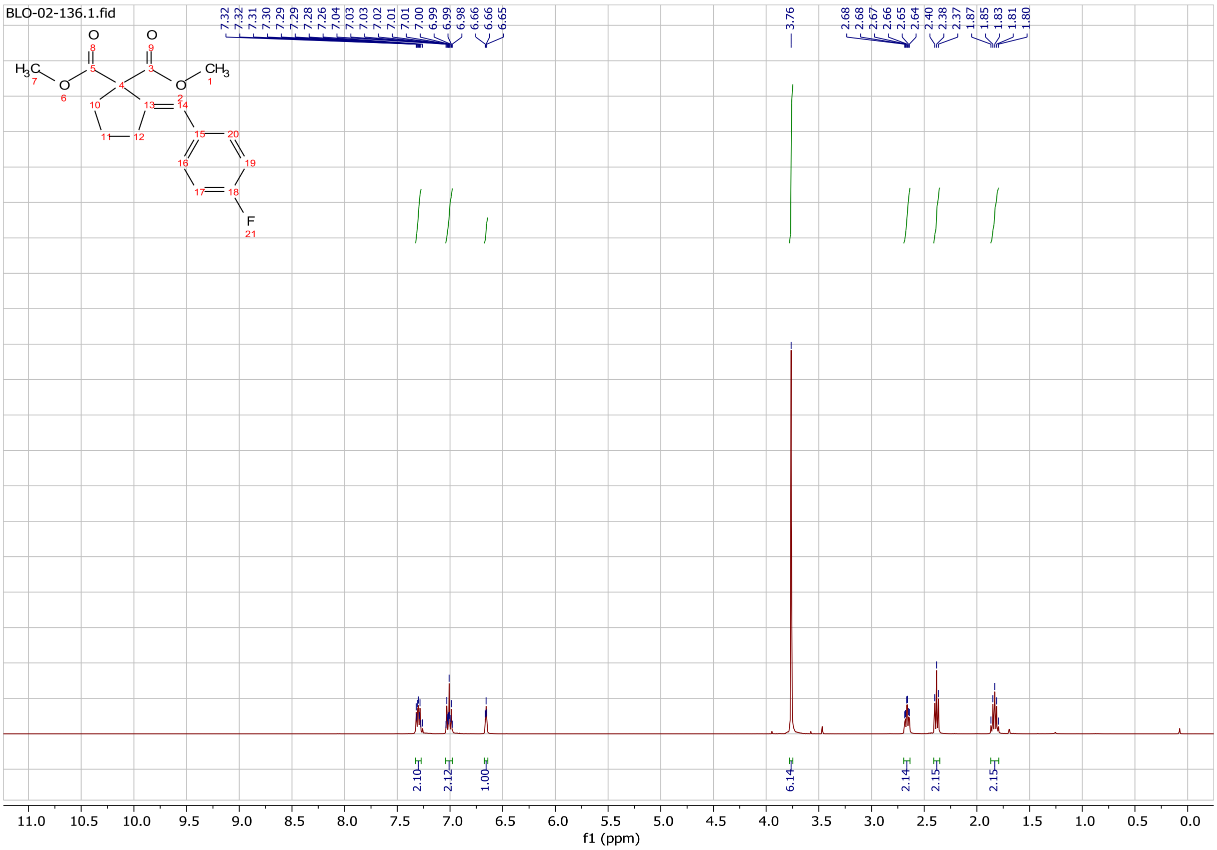

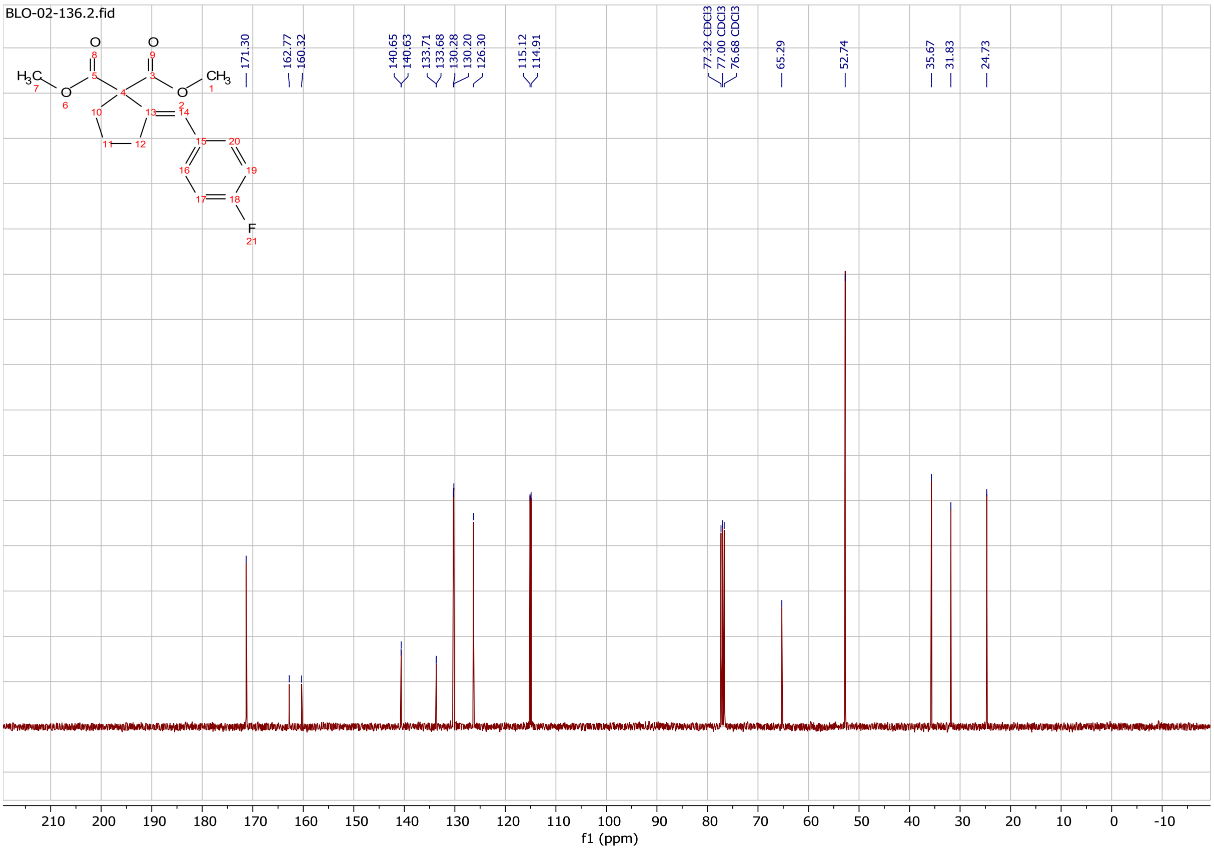

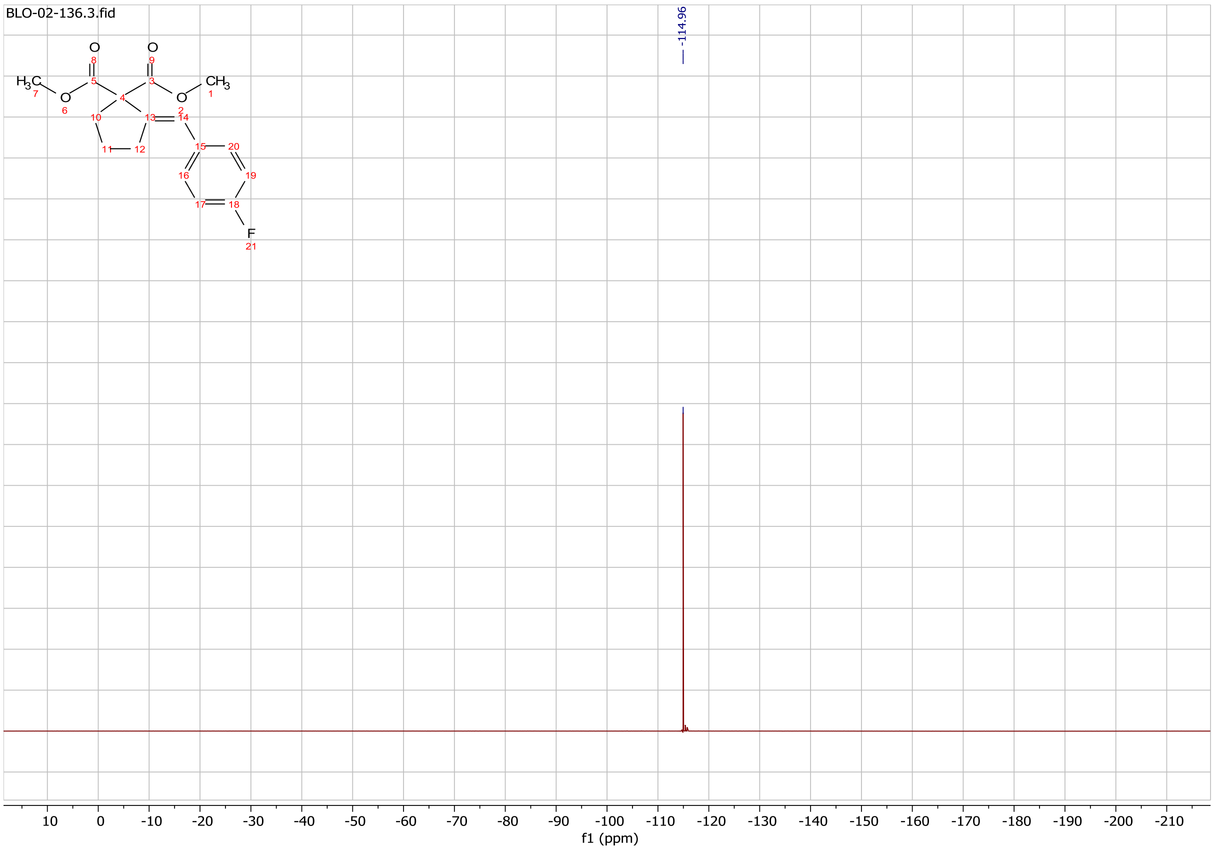
BLO-02-136.3.fid
-114.96
H3C
O
CH3
F
10
0
-10
-20
-30
-40
-50
-60
-70
-80
-90
-100
-110
-120
-130
-140
-150
-160
-170
-180
-190
-200
-210
f1 (ppm)

Dimethyl (2E)-2-(4-(methoxycarbonyl)benzylidene)cyclopentane-1,1-dicarboxylate (56).

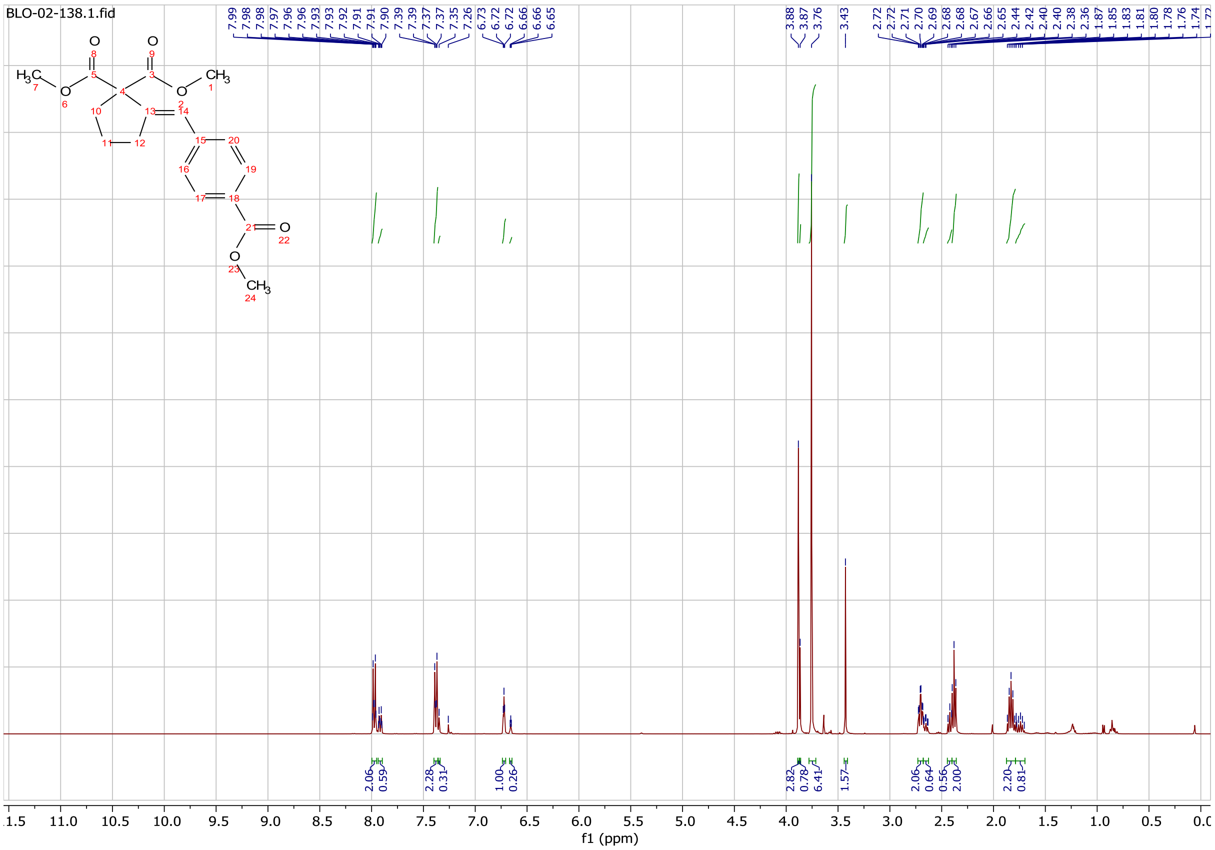

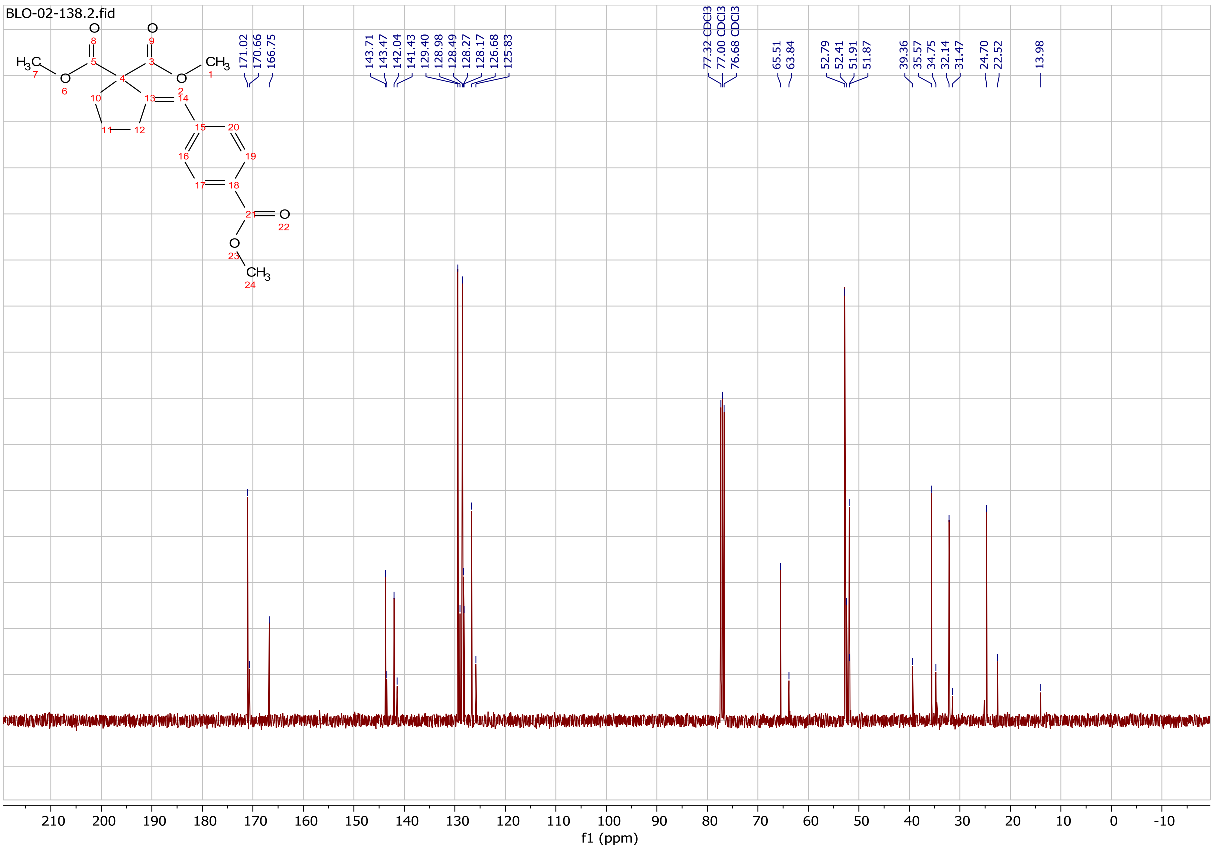

Dipropan-2-yl pent-4-yn-1-ylpropanedioate (S1)

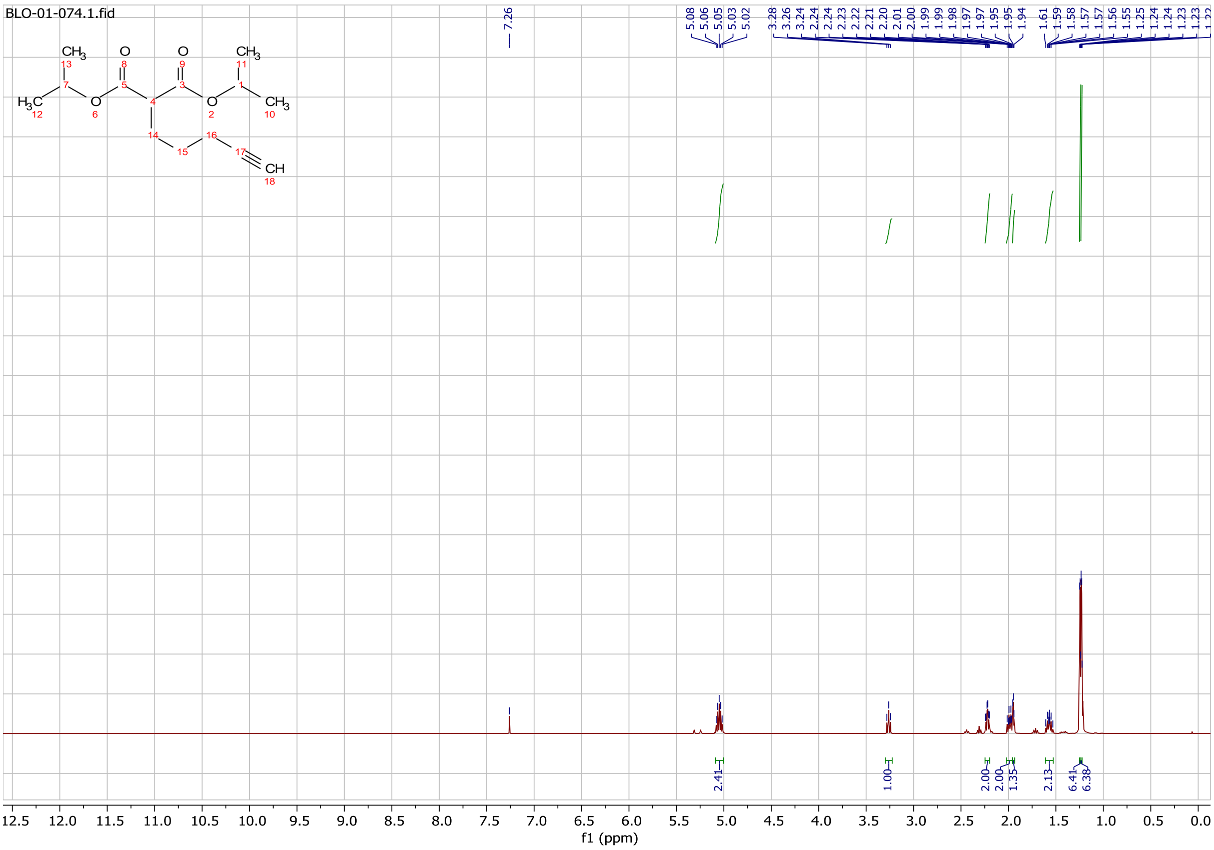

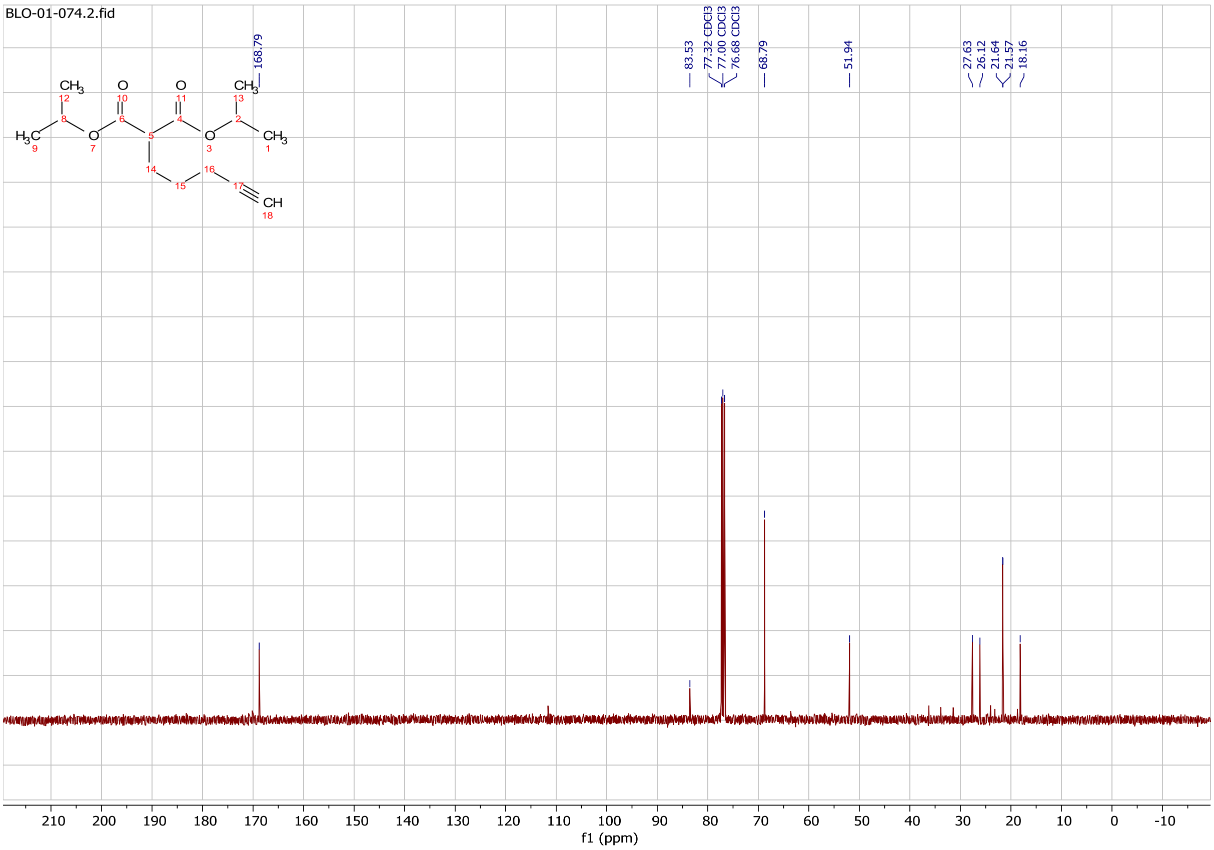

Di-*tert*-butyl pent-4-yn-1-ylpropanedioate (S2)

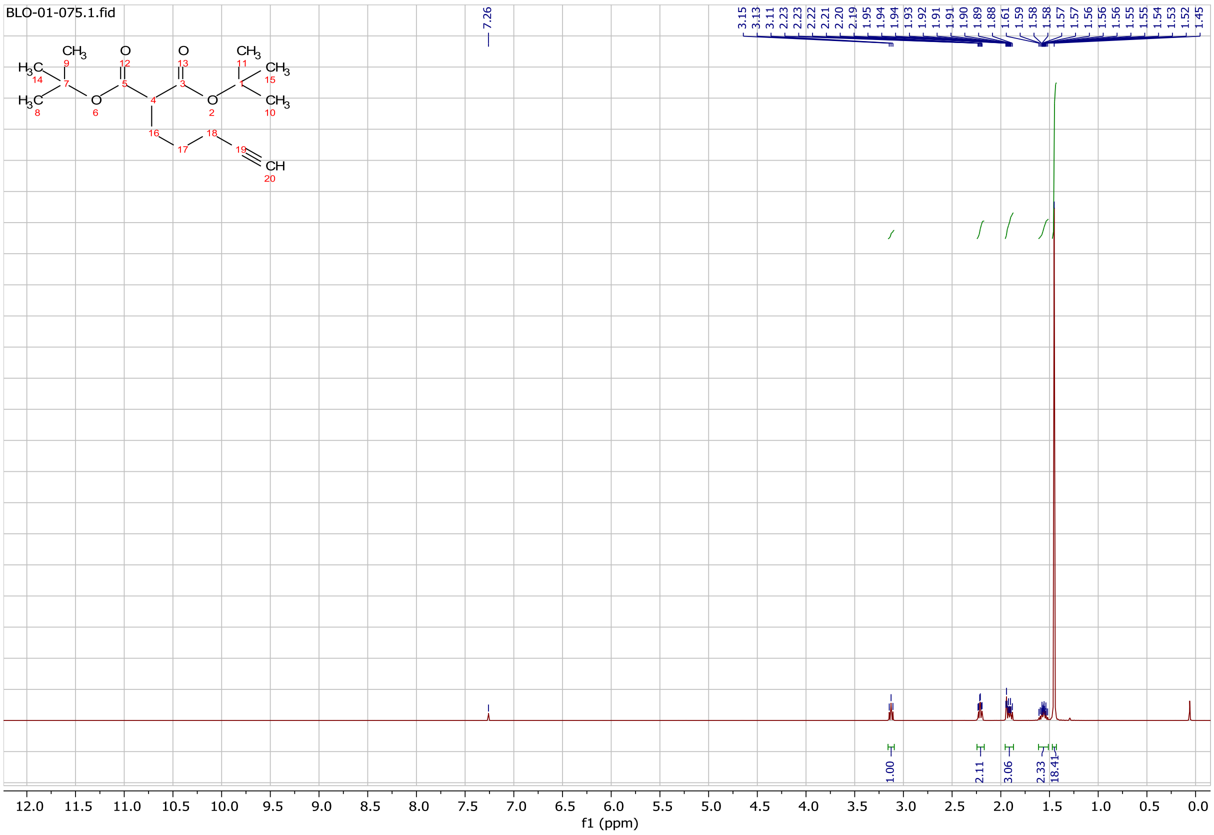

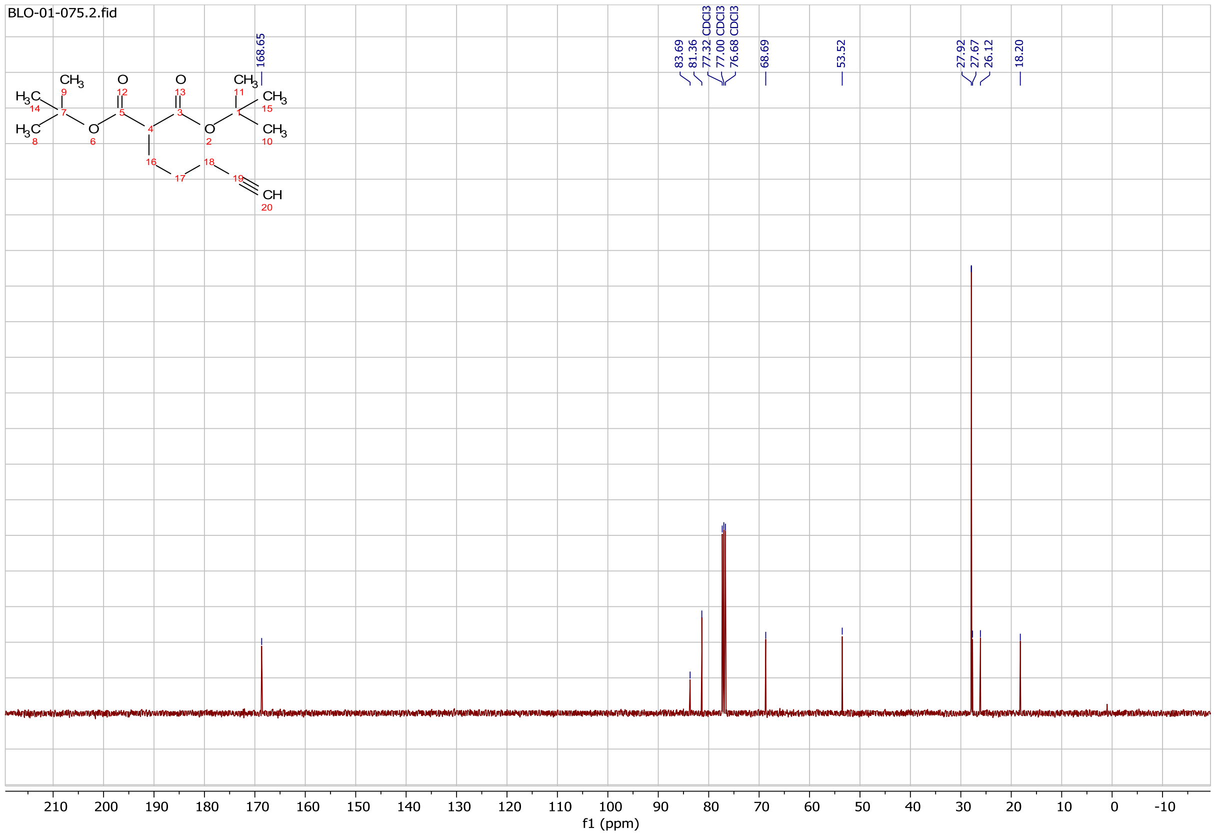

Propan-2-yl 2-cyanohept-6-ynoate (S3)

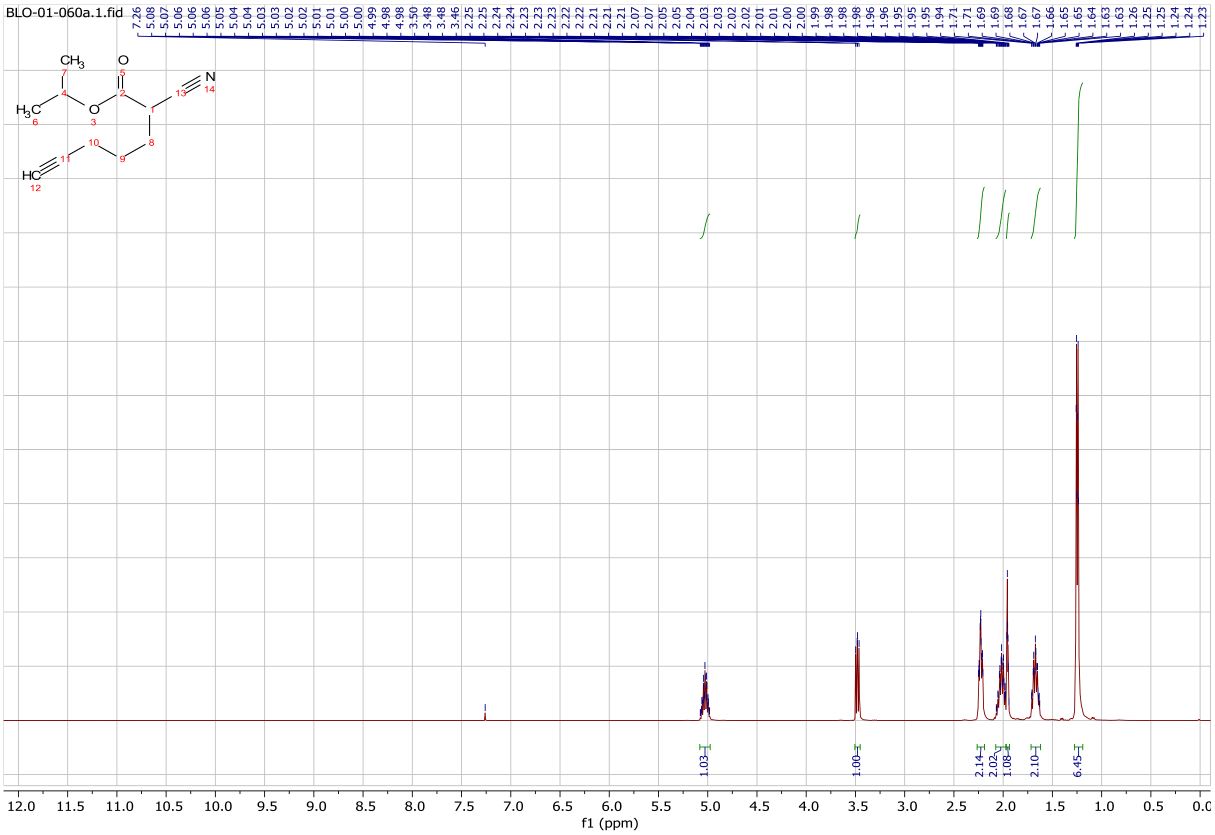

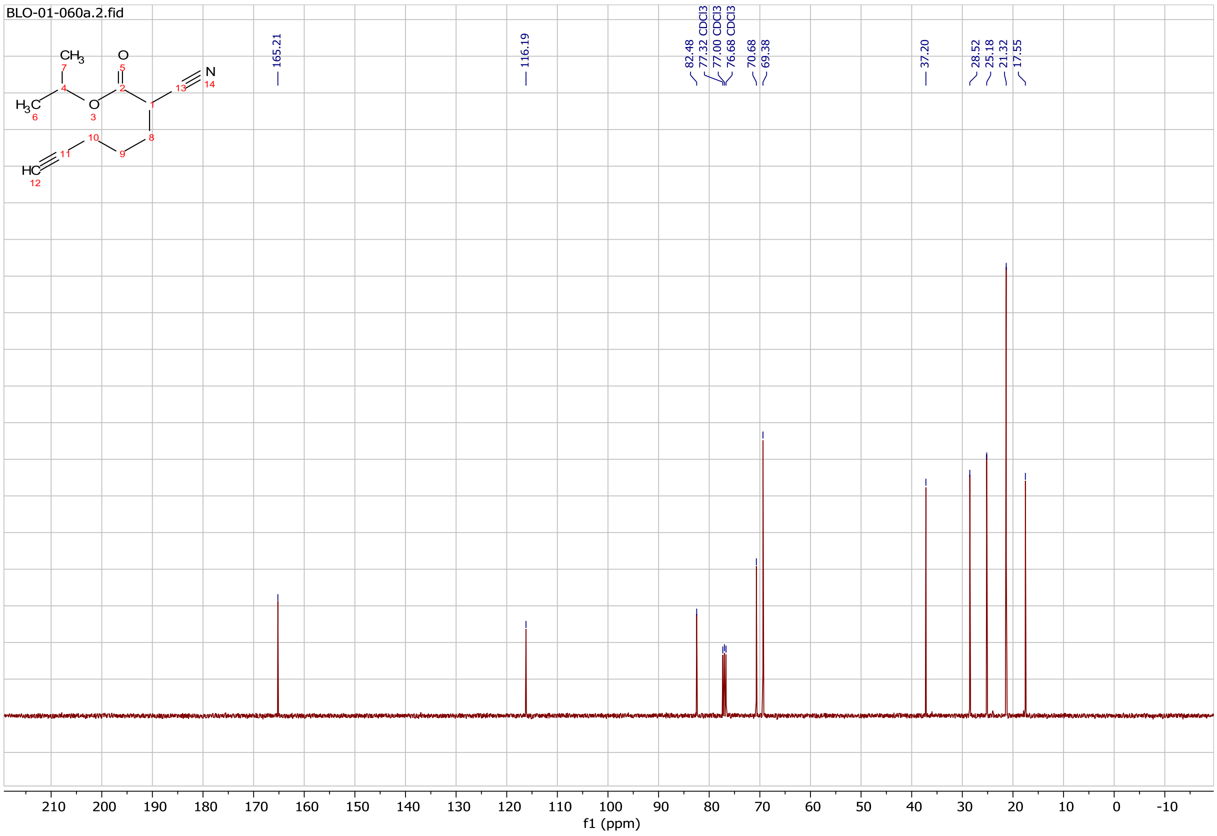

tert-butyl 2-cyanohept-6-ynoate (S4)

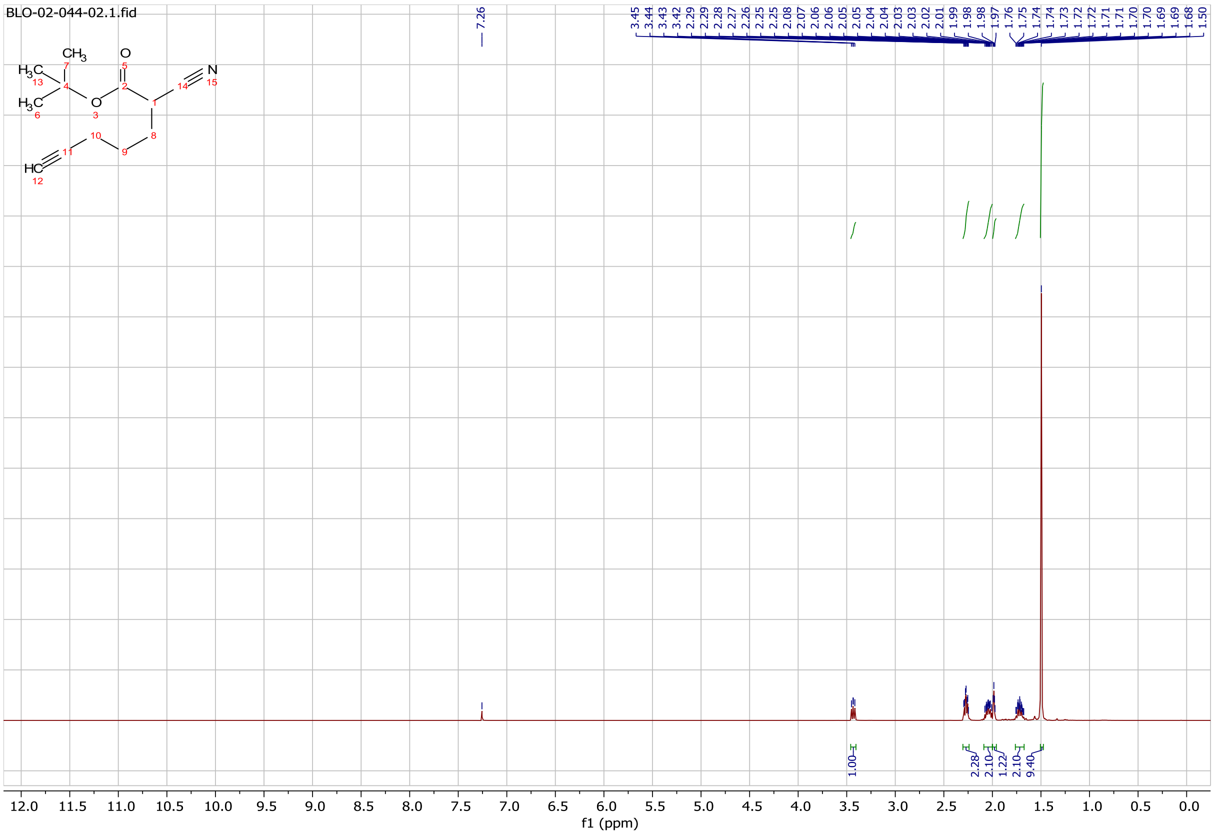

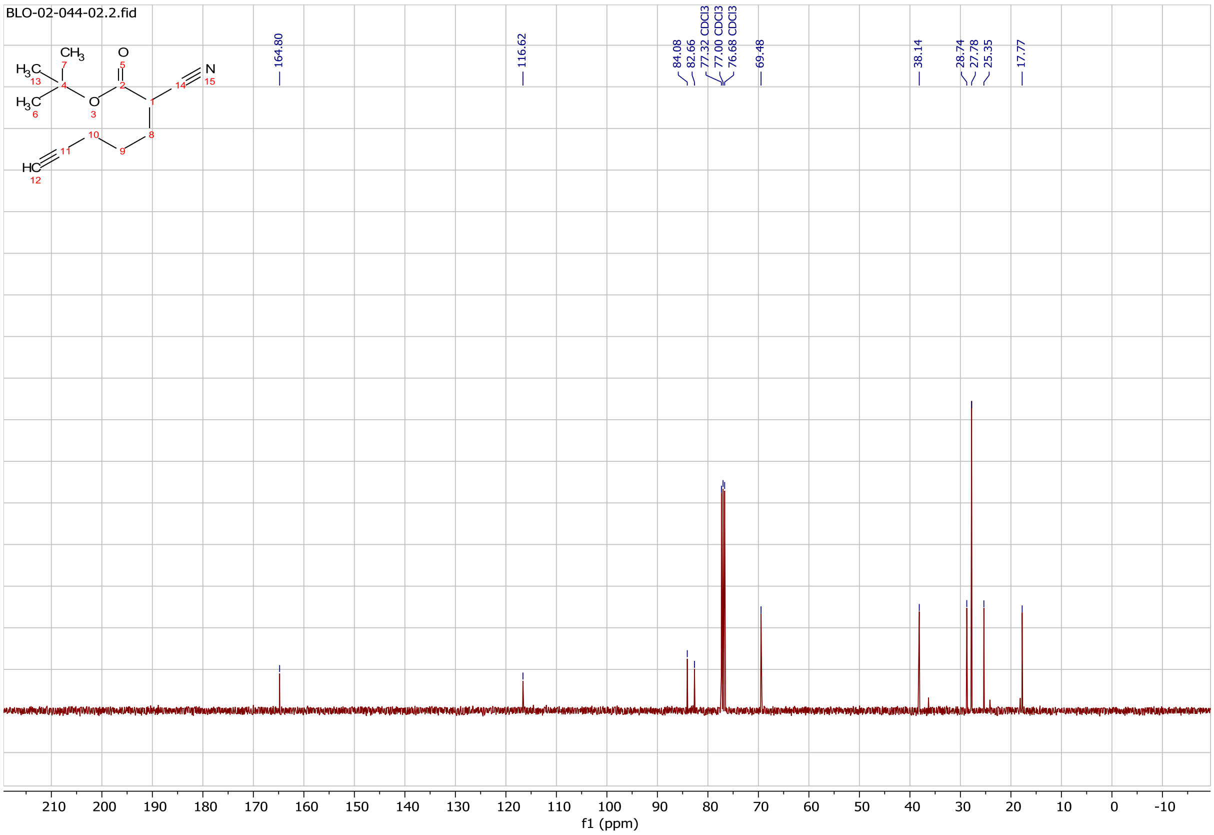

2-(Diphenylphosphoryl)acetonitrile (S13)

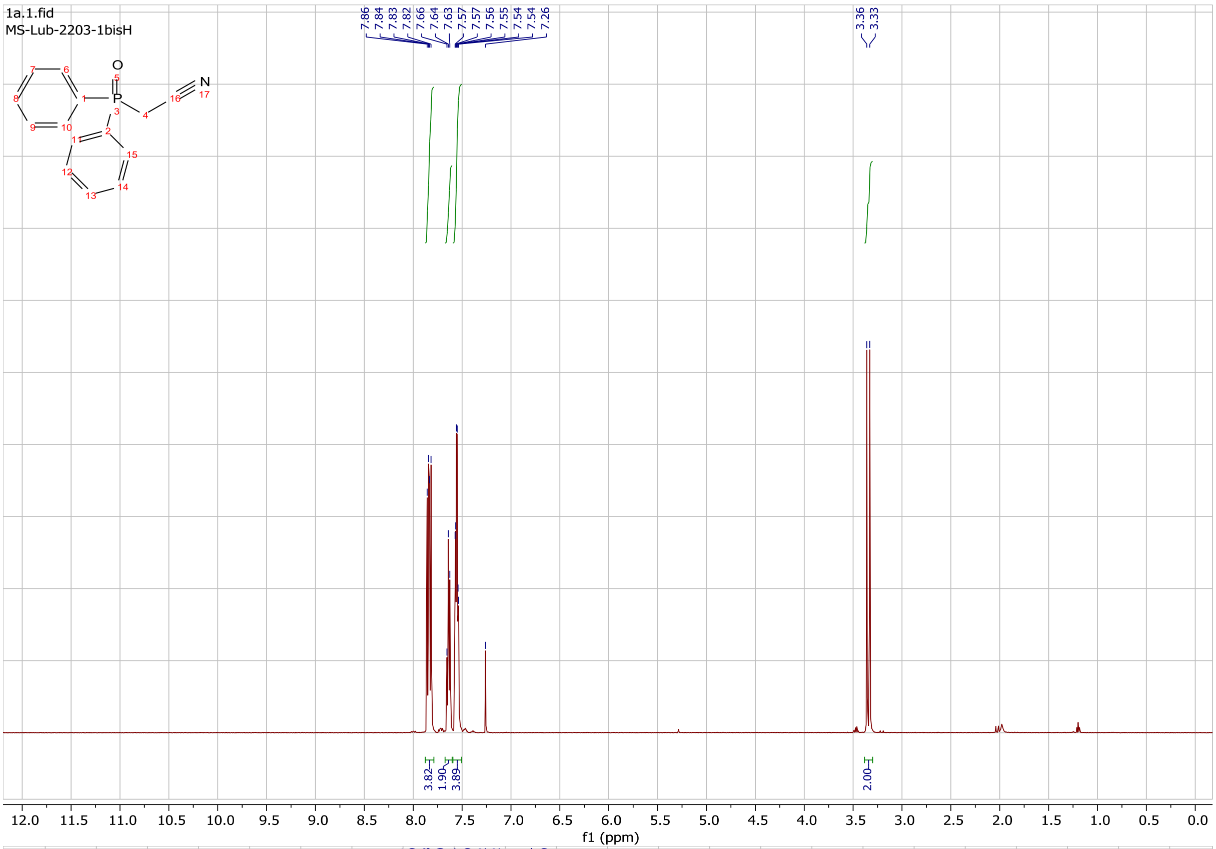

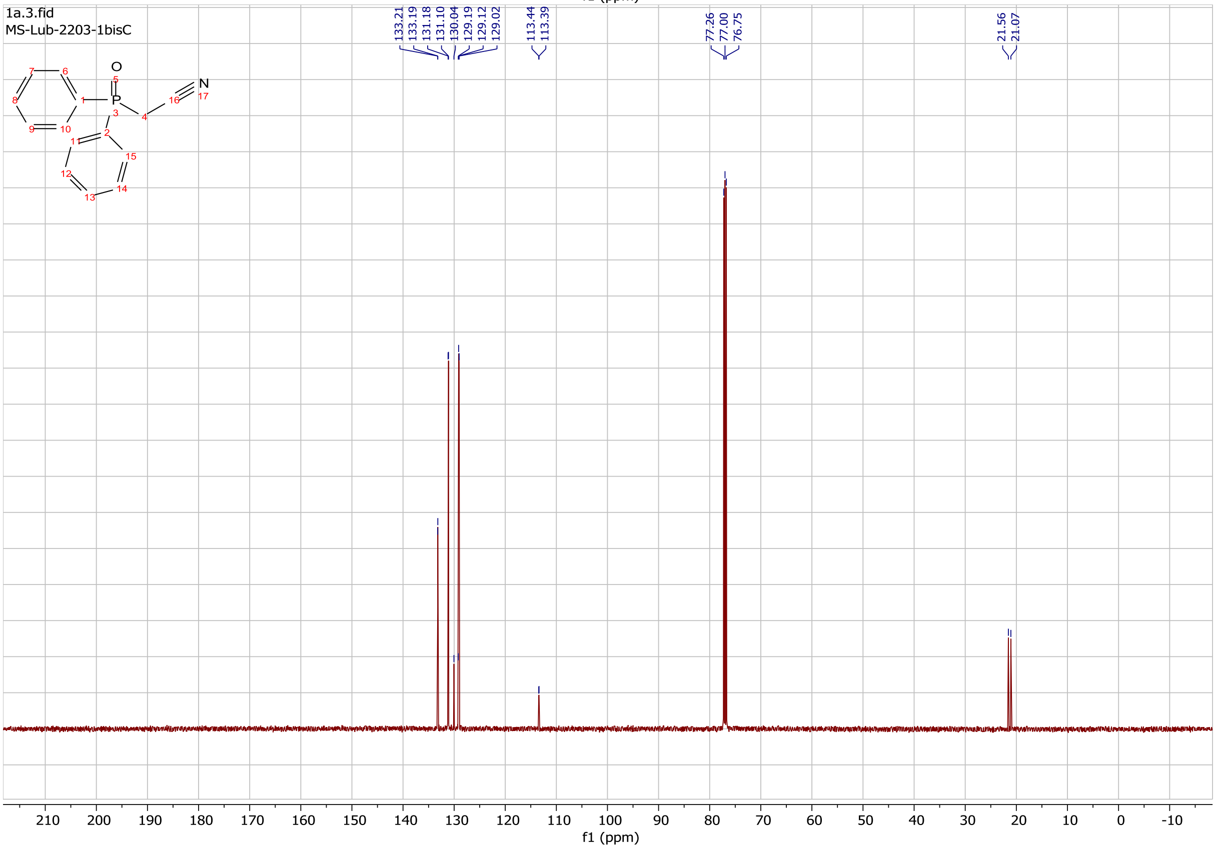

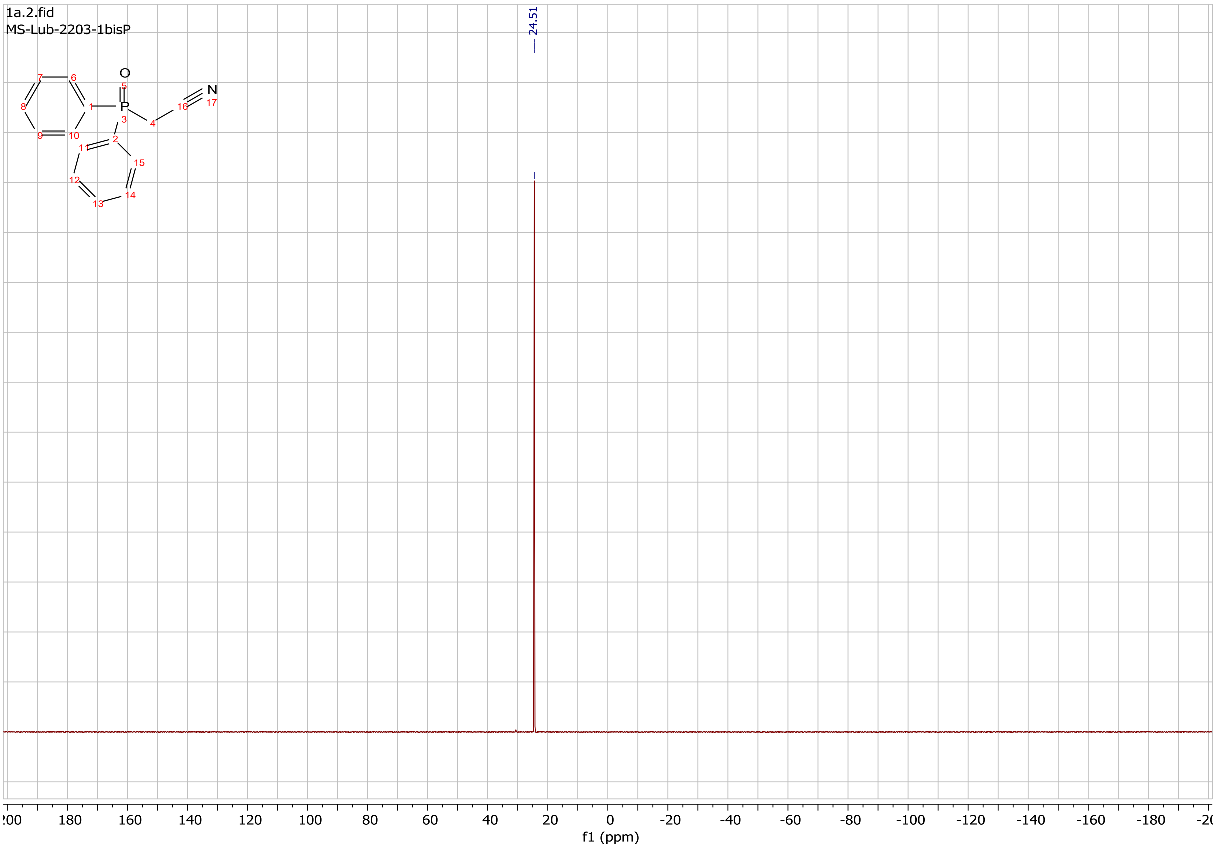
1a.2.fid
MS-Lub-2203-1bisP
24.51
O
P
N
200
180
160
140
120
100
80
60
40
20
0
-20
-40
-60
-80
-100
-120
-140
-160
-180
f1 (ppm)

1-(Diphenylphosphoryl)propan-2-one (S14)

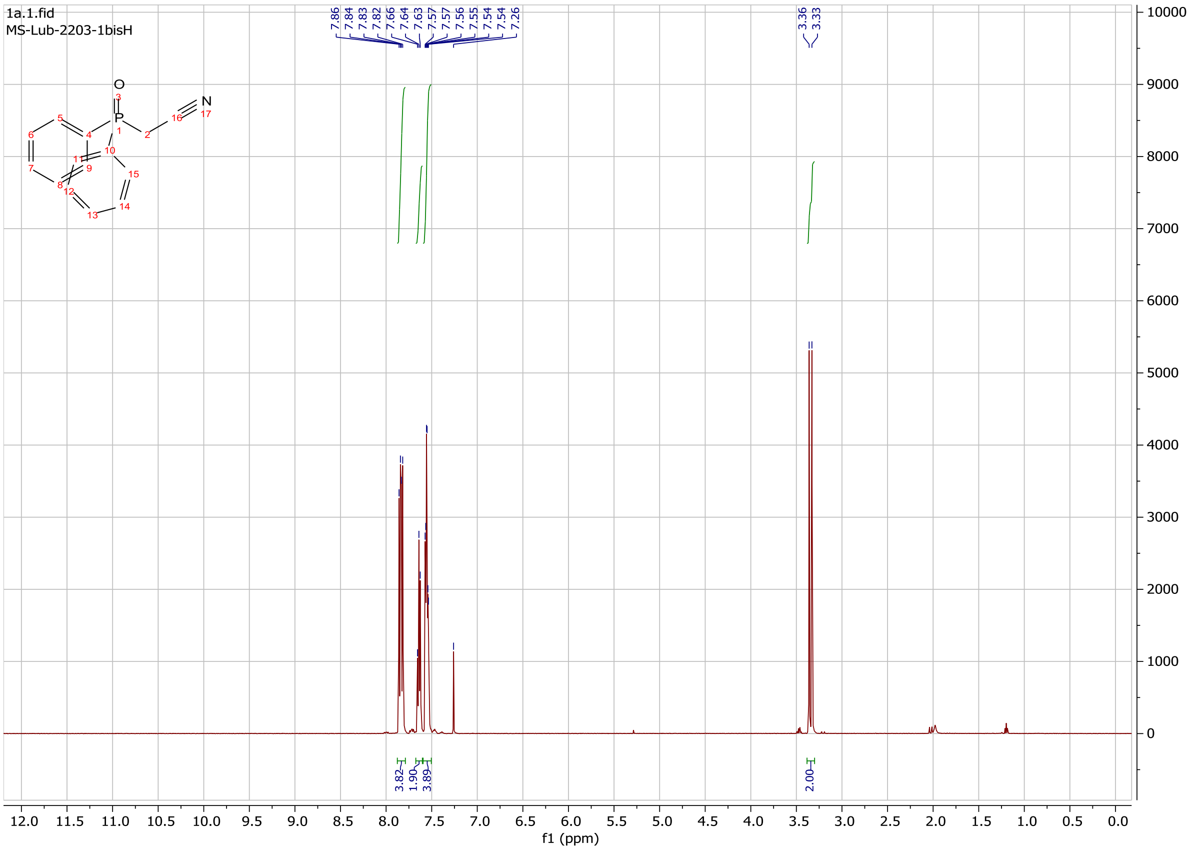

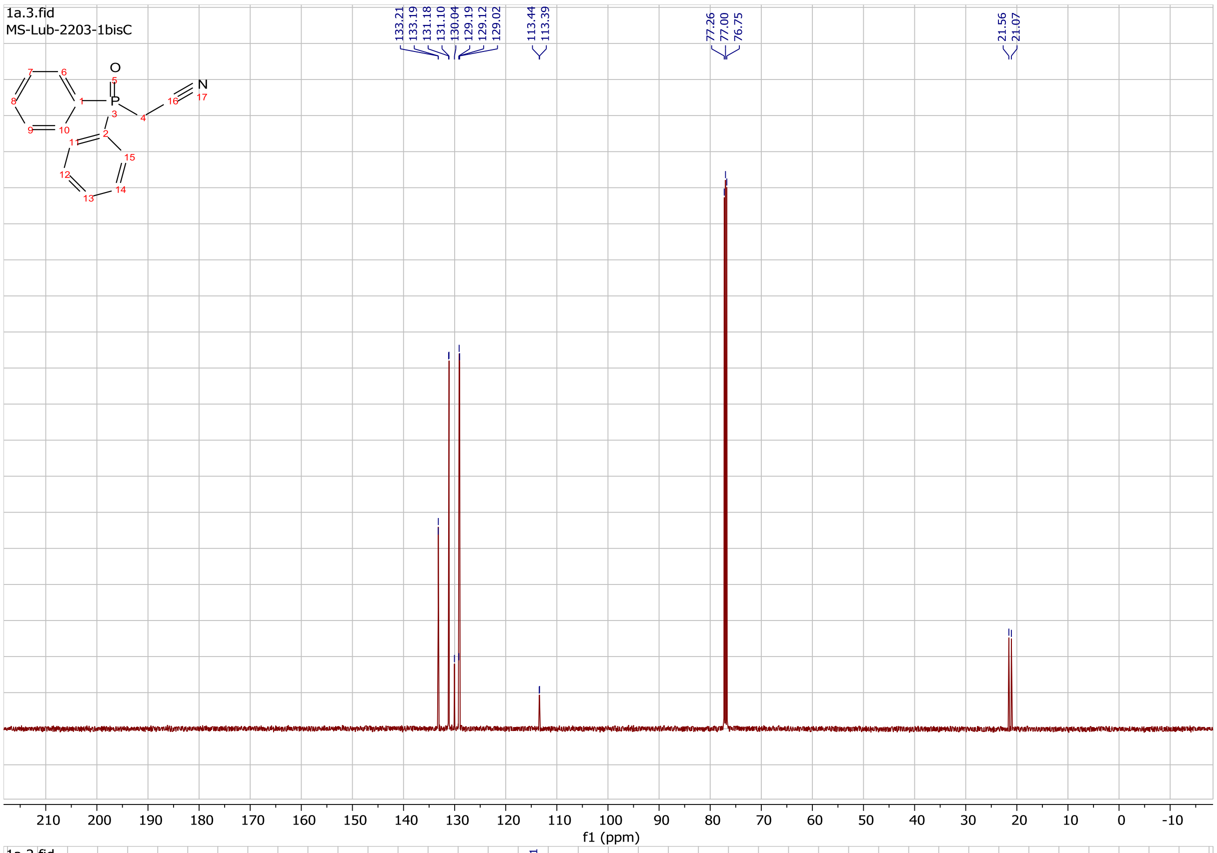
1a.3.fid
MS-Lub-2203-1bisC
133.21
133.19
131.18
131.10
130.04
129.19
129.12
129.02
113.44
113.39
77.26
77.00
76.75
21.56
21.07
f1 (ppm)

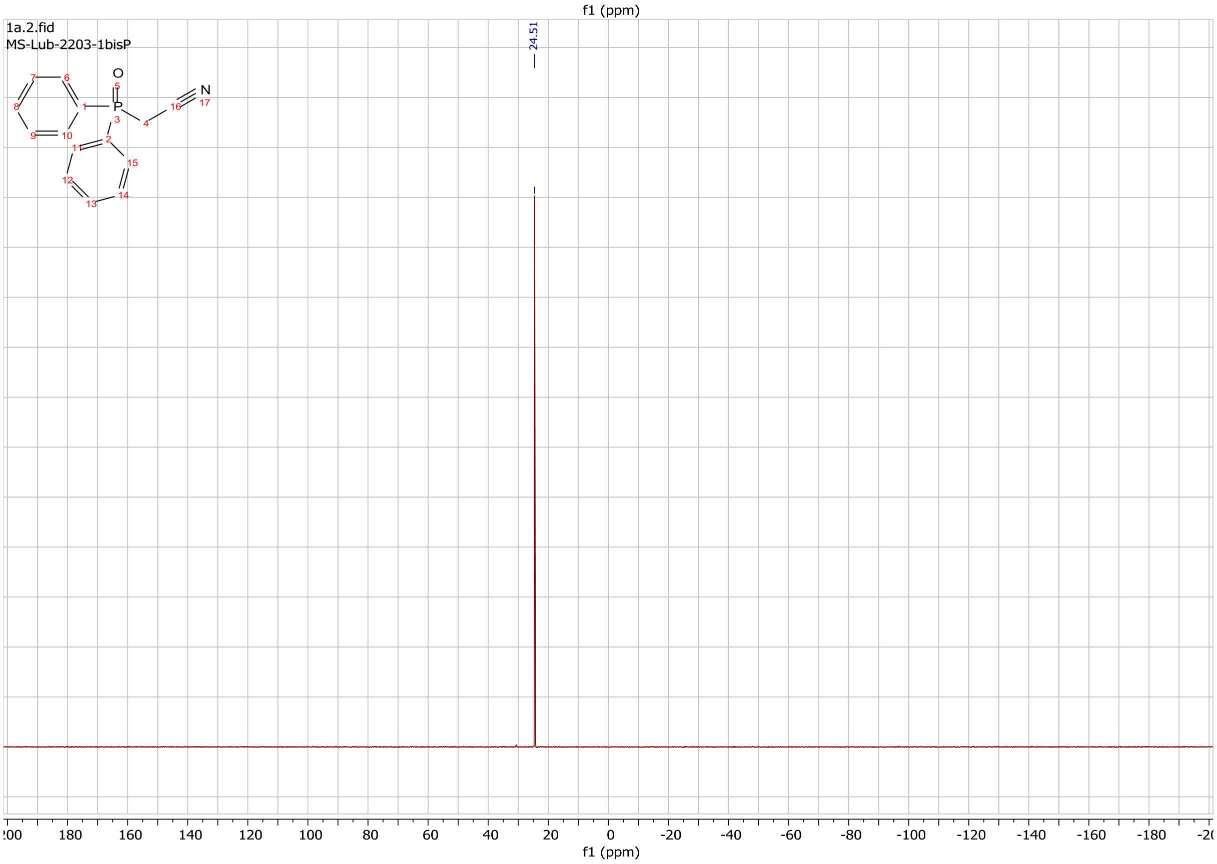
1a.2.fid
MS-Lub-2203-1bisP
24.51
f1 (ppm)

Ethyl 2-(diphenylphosphoryl)acetate (S15)

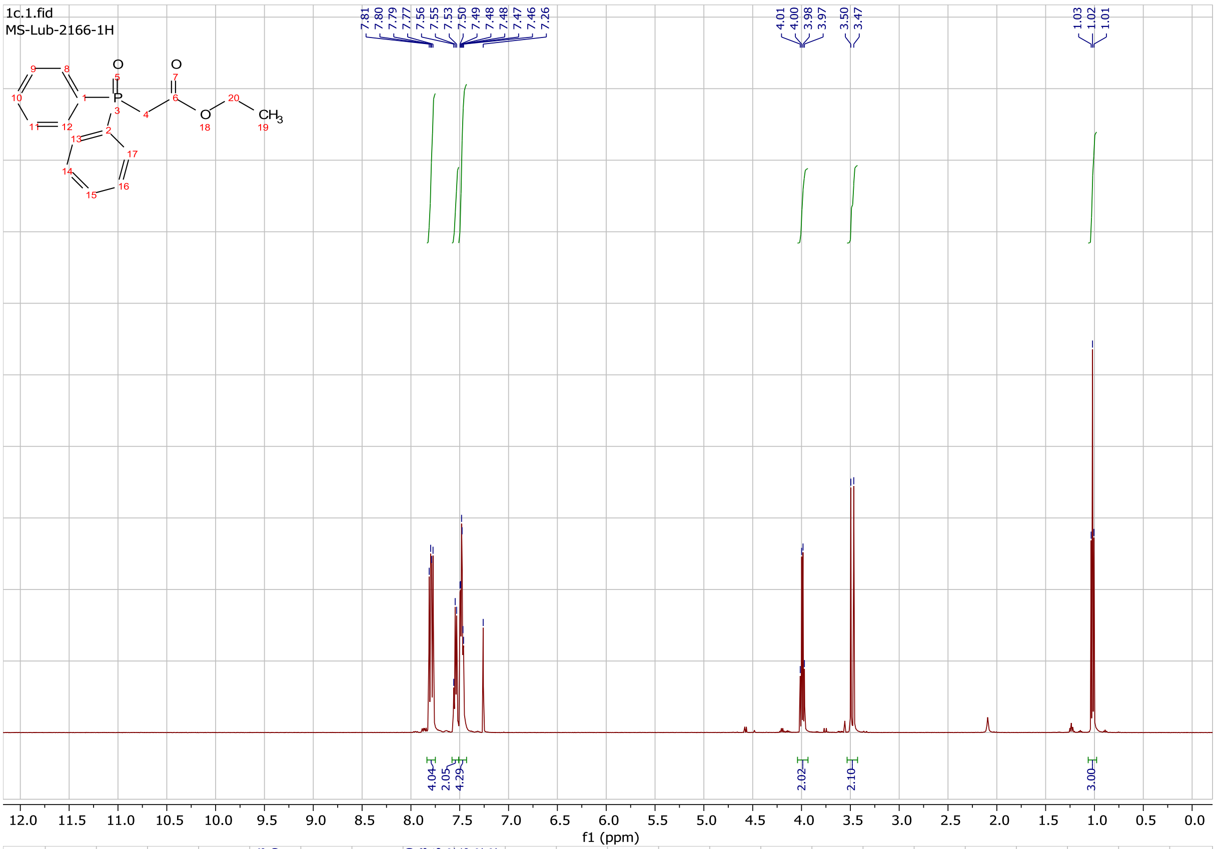

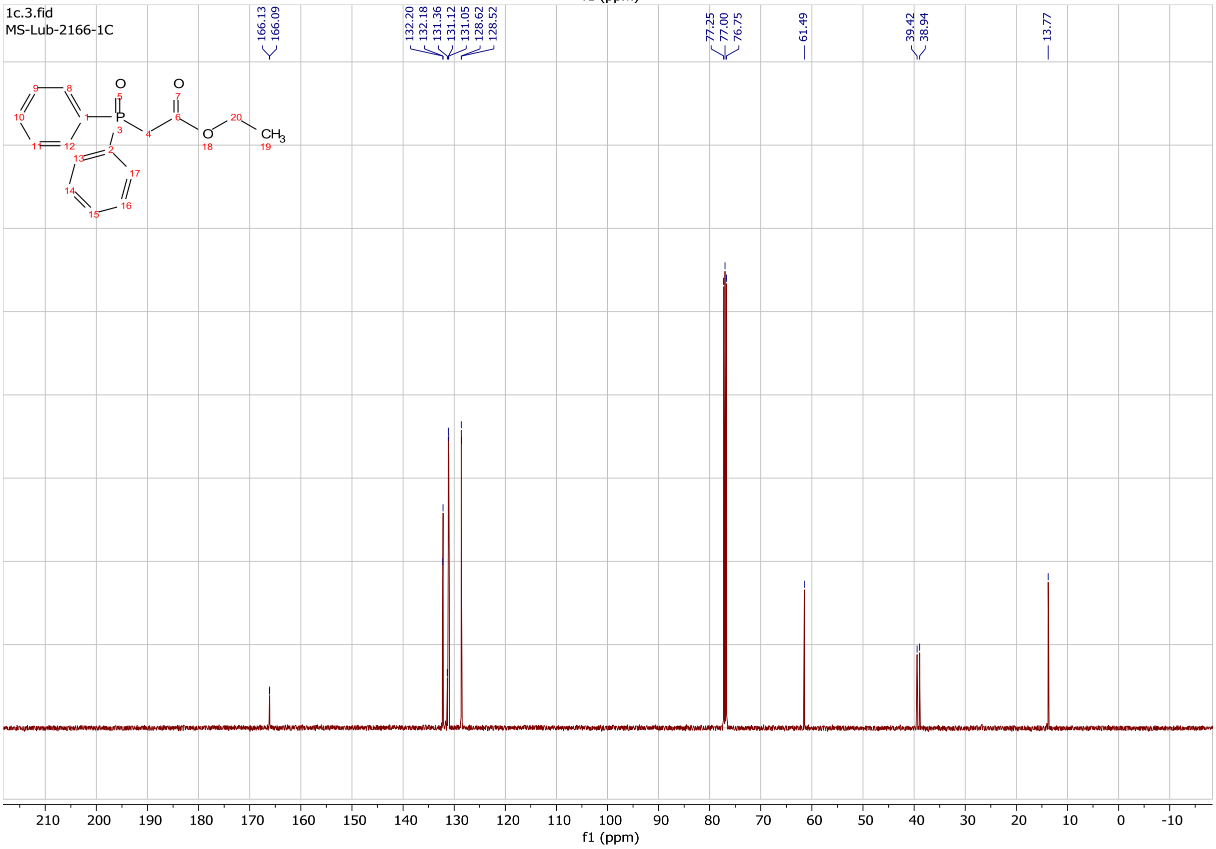

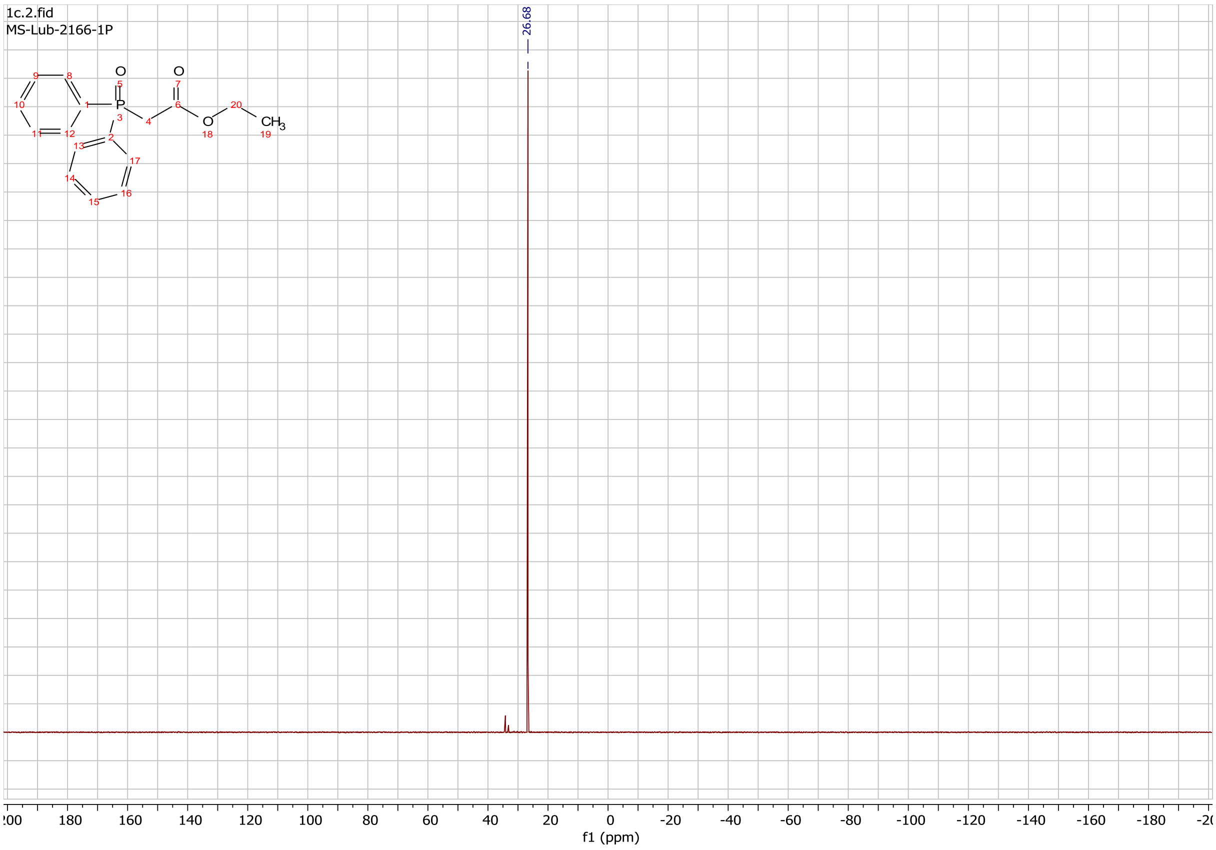
1c.2.fid
MS-Lub-2166-1P
26.68
f1 (ppm)

Diisopropyl (cyanomethyl)phosphonate (S16)

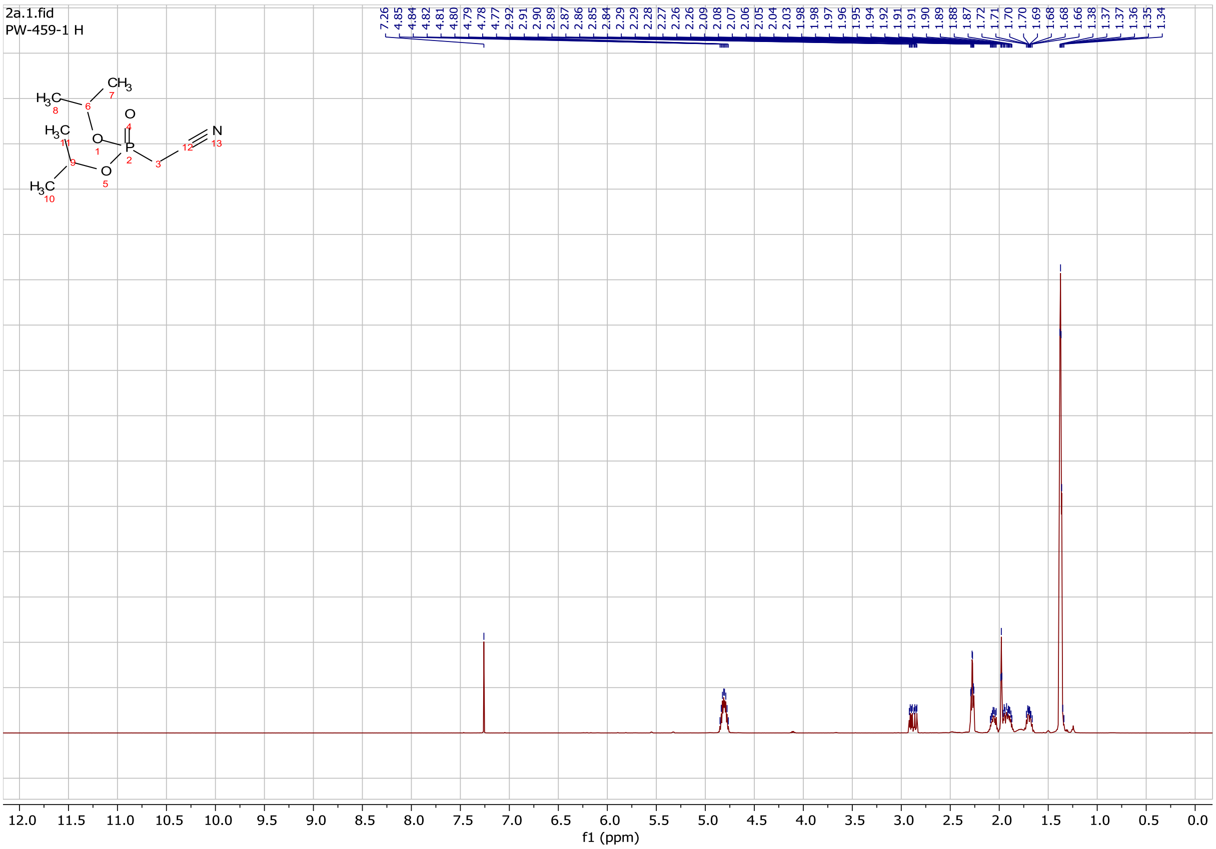

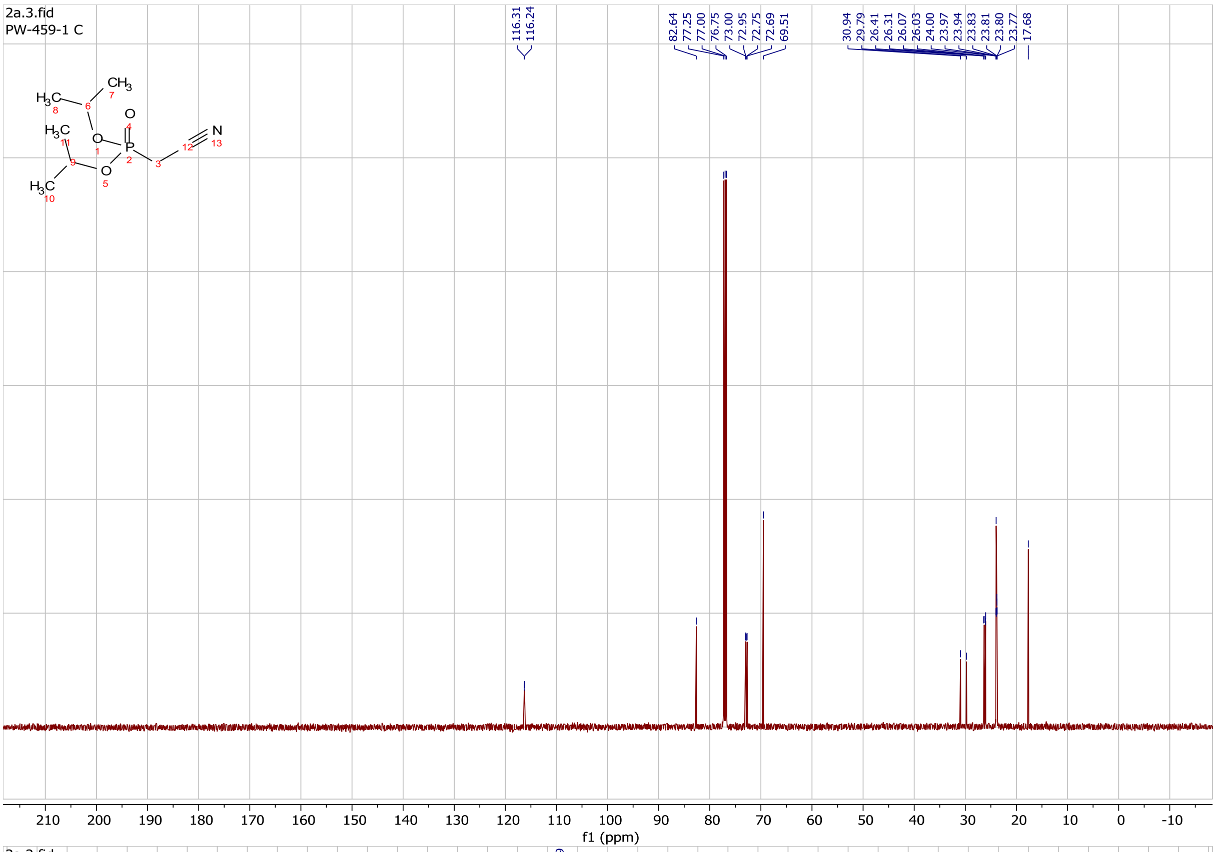
2a.3.fid
PW-459-1 C
116.31
116.24
82.64
77.25
77.00
76.75
73.00
72.95
72.75
72.69
69.51
30.94
29.79
26.41
26.31
26.07
26.03
24.00
23.97
23.94
23.83
23.81
23.80
23.77
17.68
f1 (ppm)

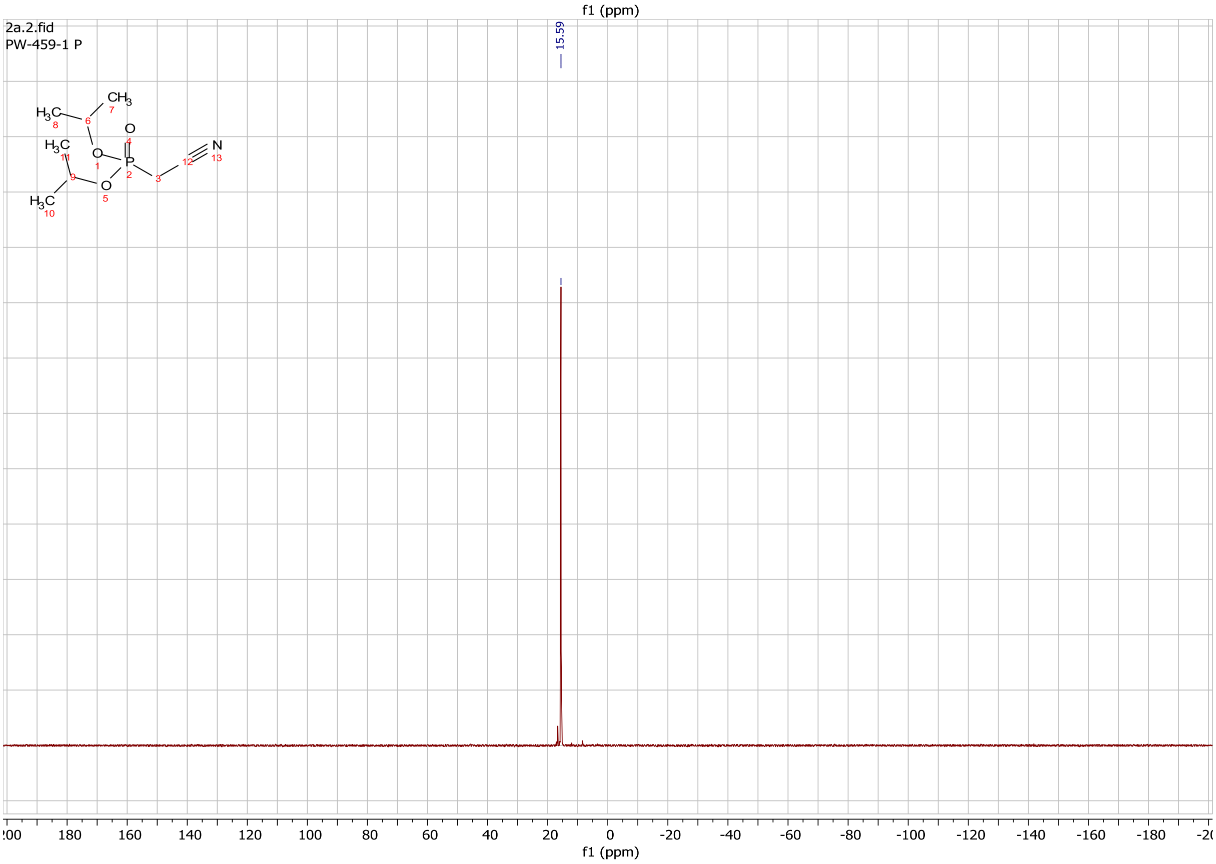
2a.2.fid
PW-459-1 P
15.59
f1 (ppm)

Diethyl (2-oxopropyl)phosphonate (S17).

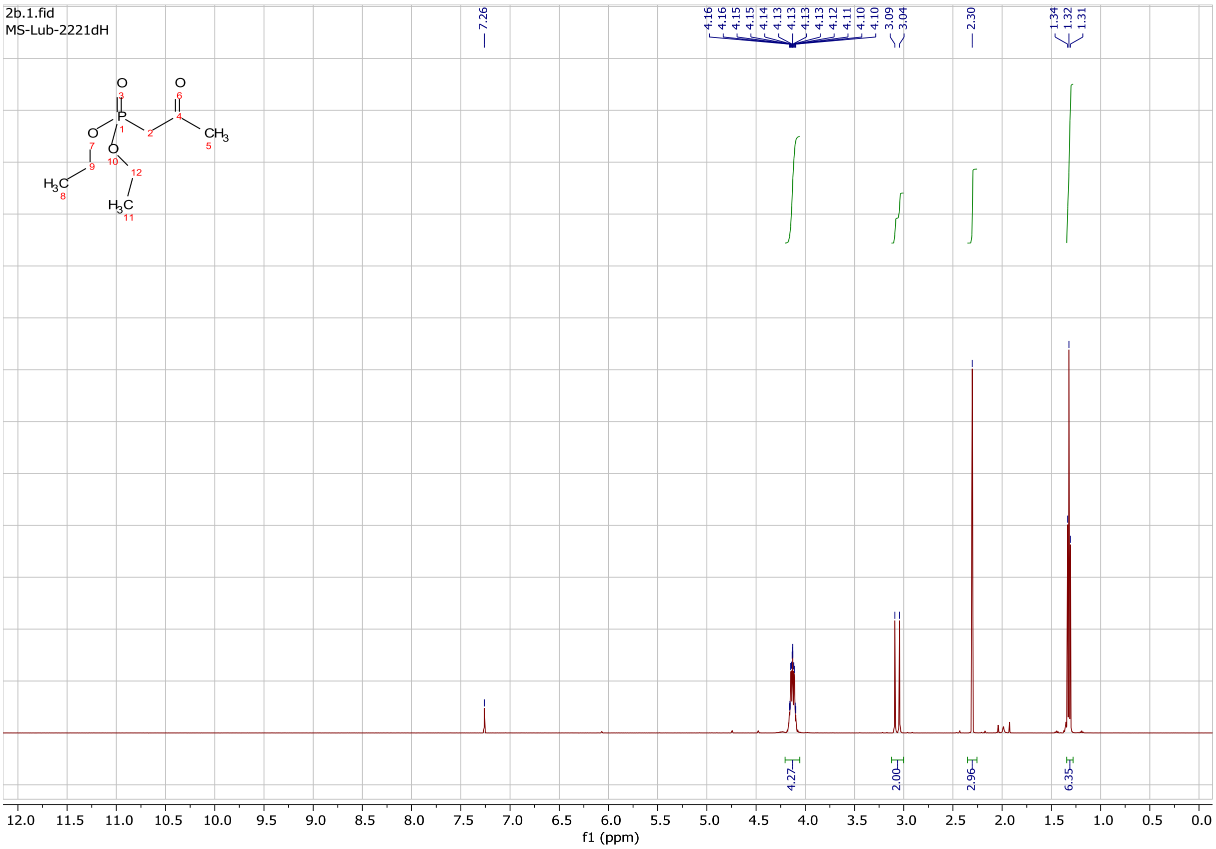

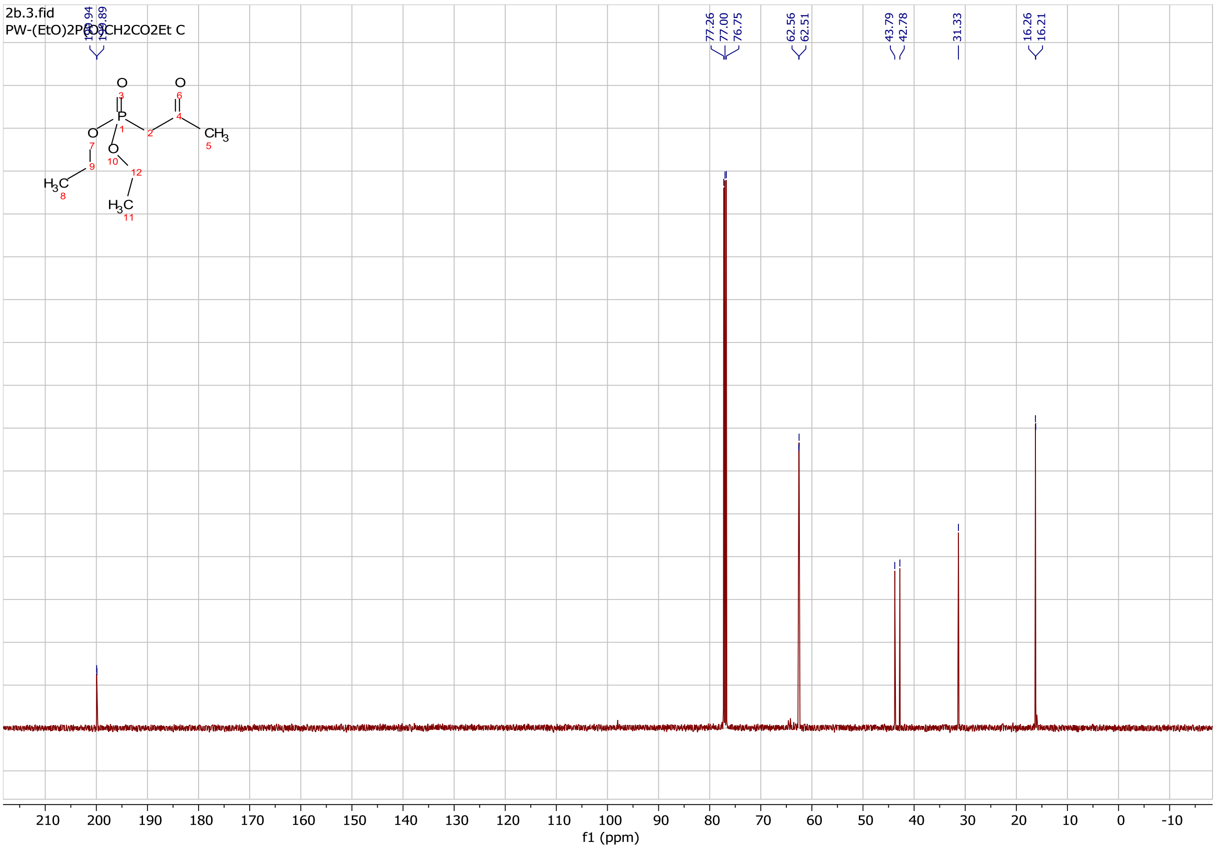

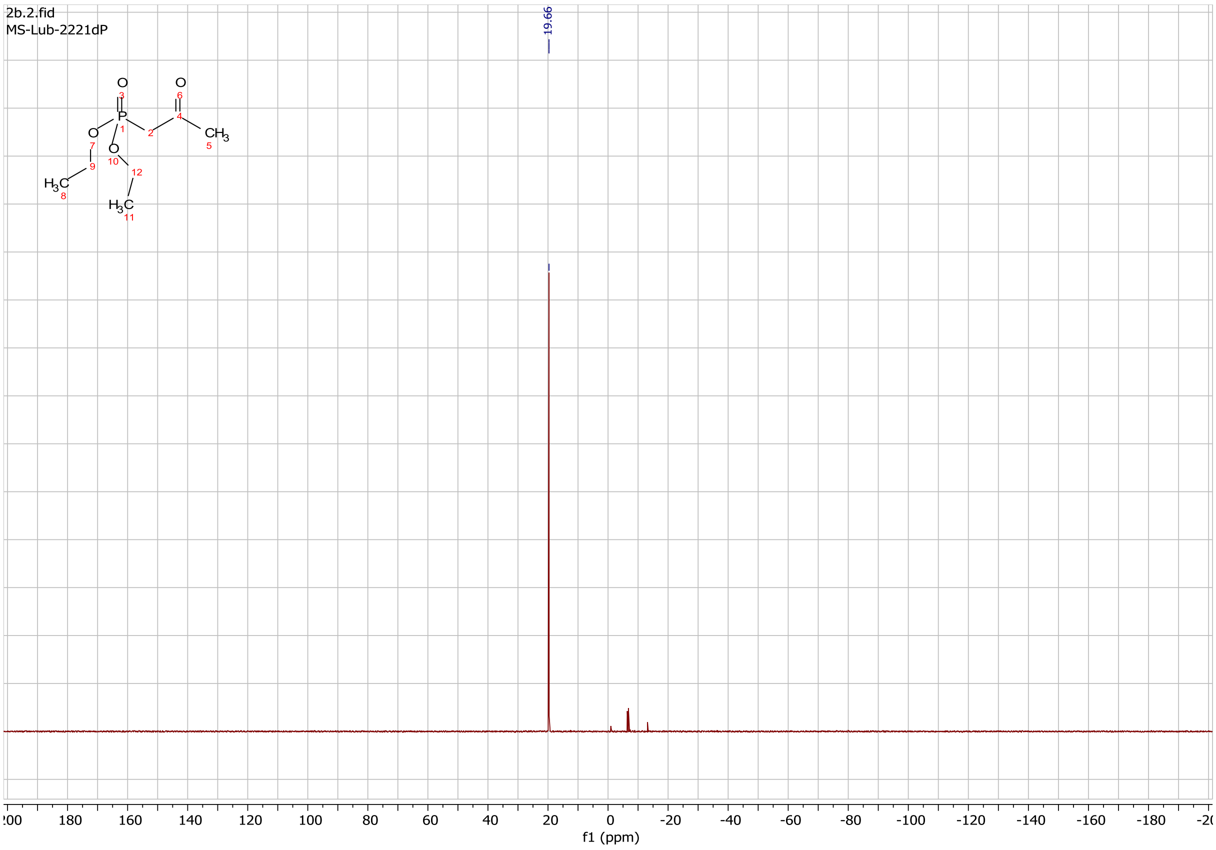
2b.2.fid
MS-Lub-2221dP
19.66
f1 (ppm)

Ethyl 2-(diisopropoxyphosphoryl)acetate (S18)

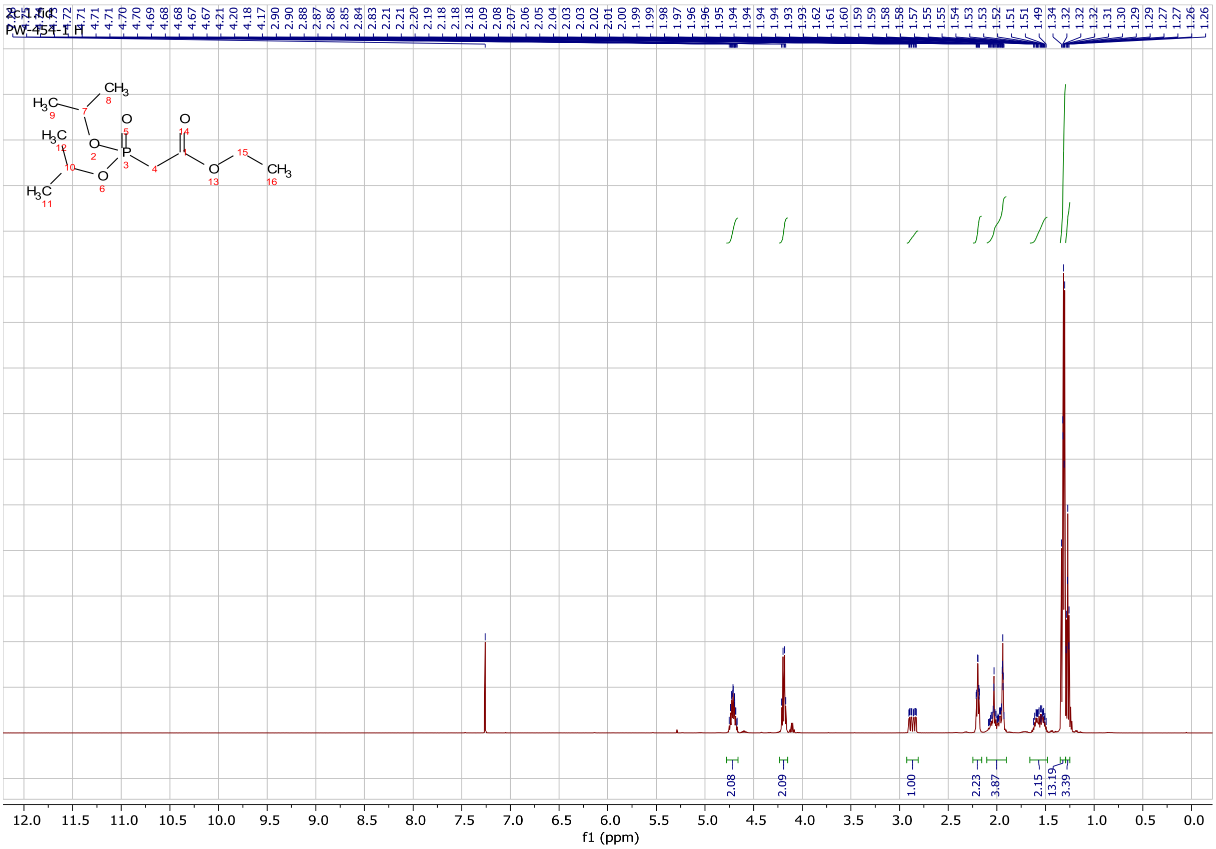

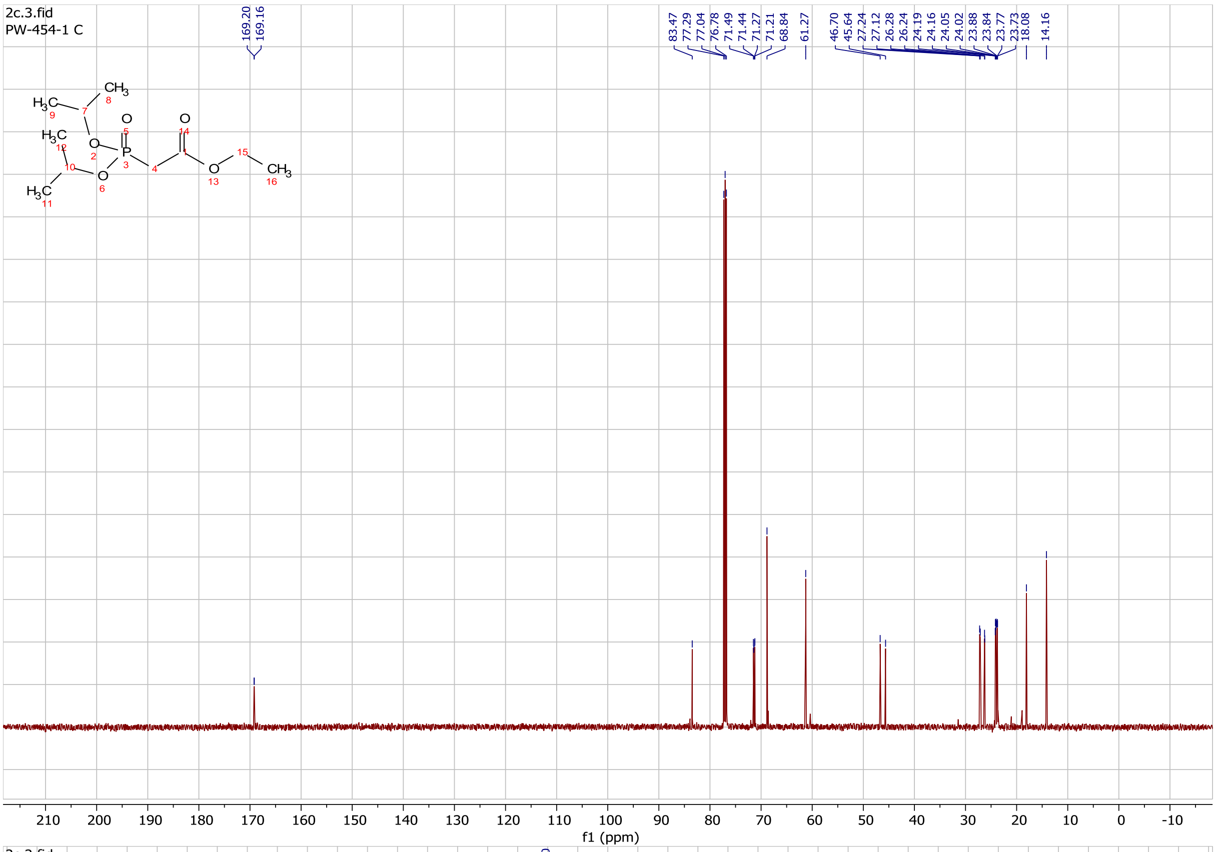
2c.3.fid
PW-454-1 C
169.20
169.16
83.47
77.29
77.04
76.78
71.49
71.44
71.27
71.21
68.84
61.27
46.70
45.64
27.24
27.12
26.28
26.24
24.19
24.16
24.05
24.02
23.88
23.84
23.77
23.73
18.08
14.16
f1 (ppm)

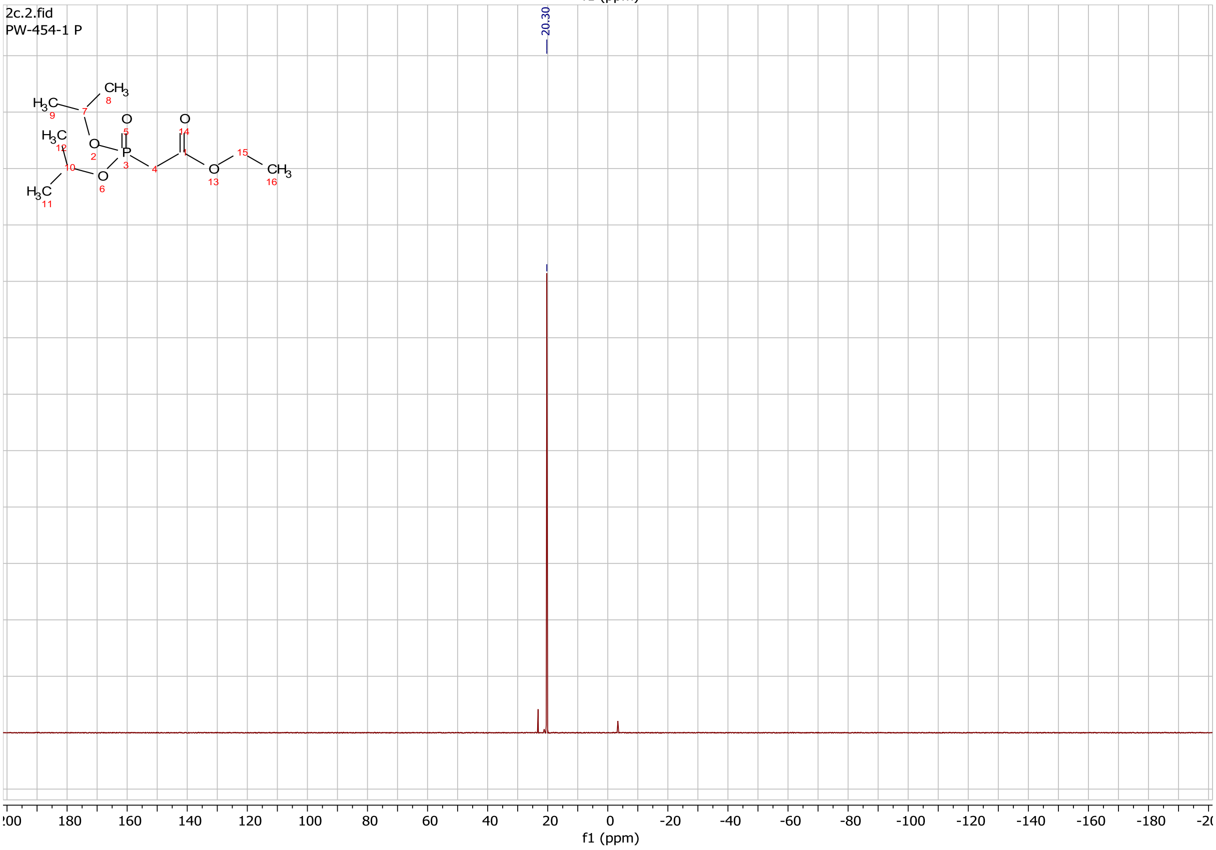
2c.2.fid
PW-454-1 P
20.30
f1 (ppm)

Diisopropyl (1-cyanohex-5-yn-1-yl)phosphonate (S7).

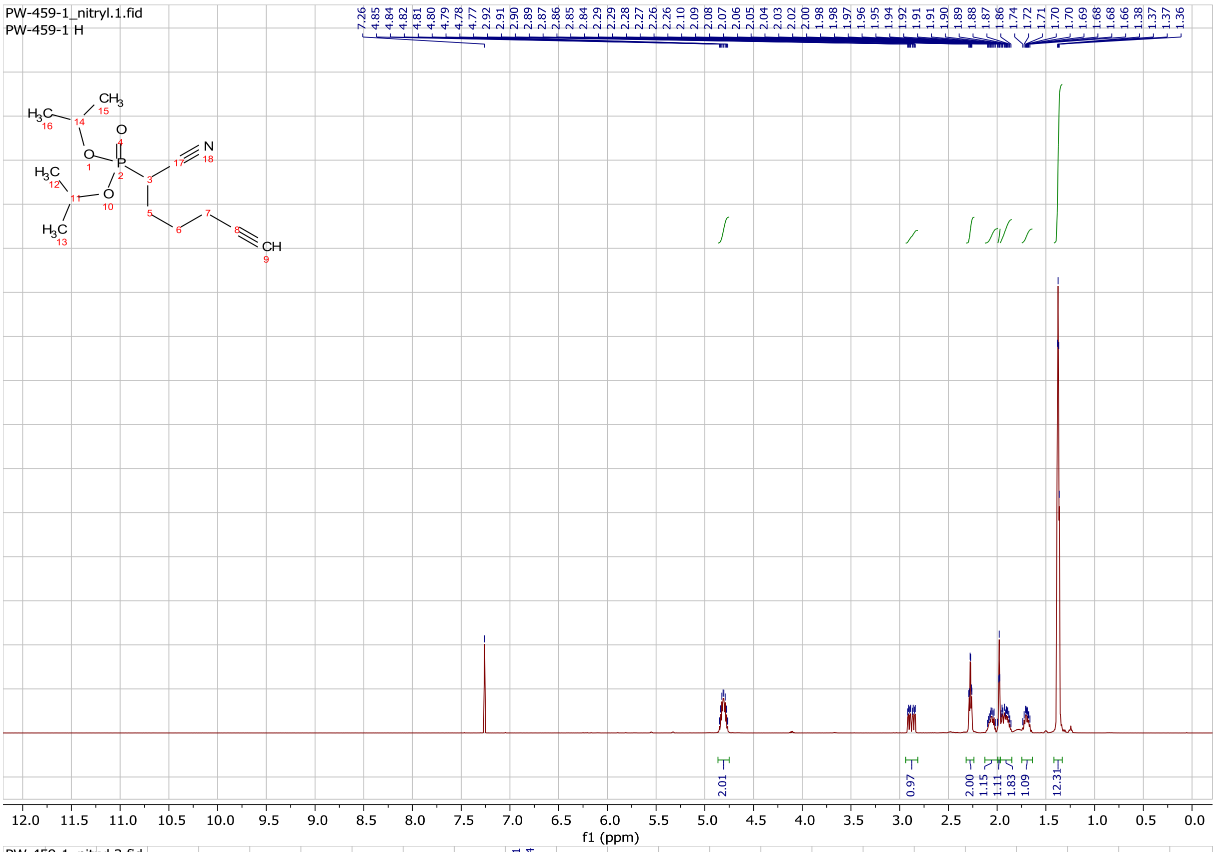

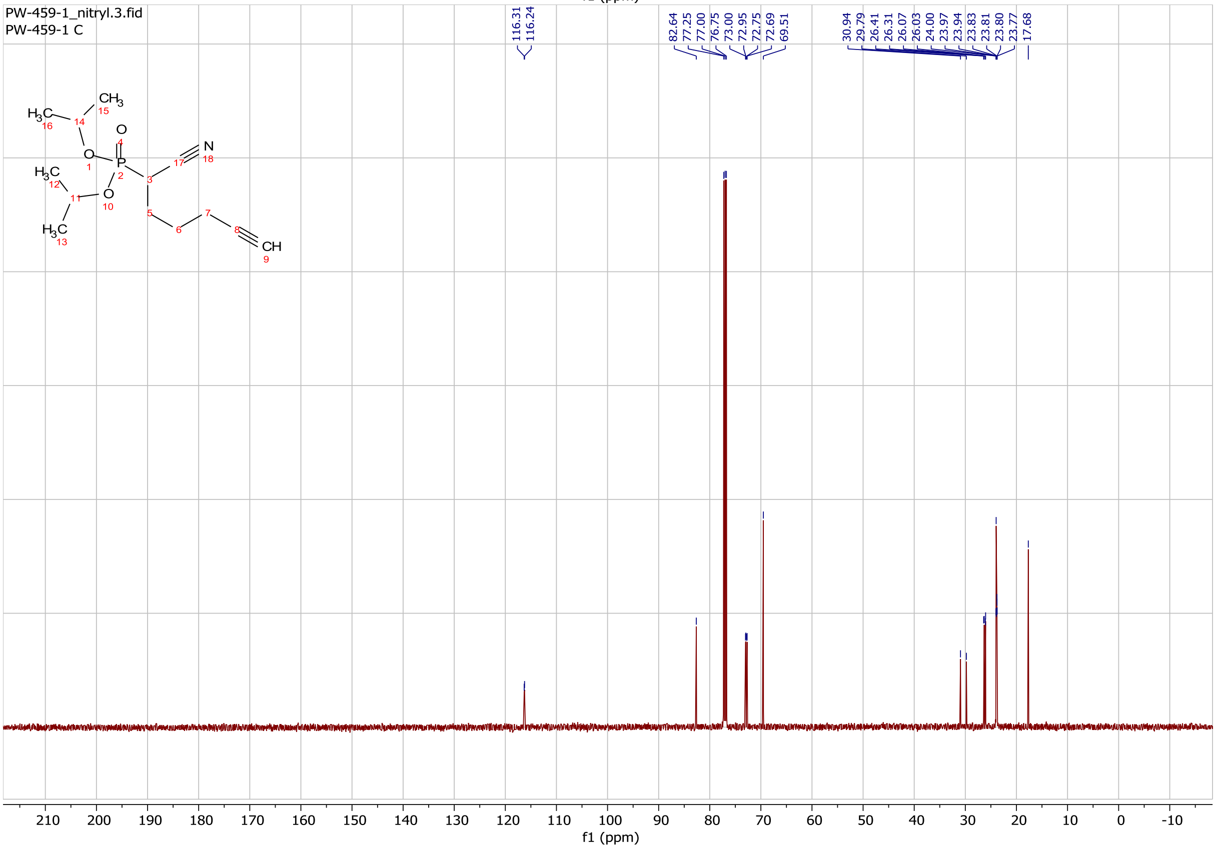

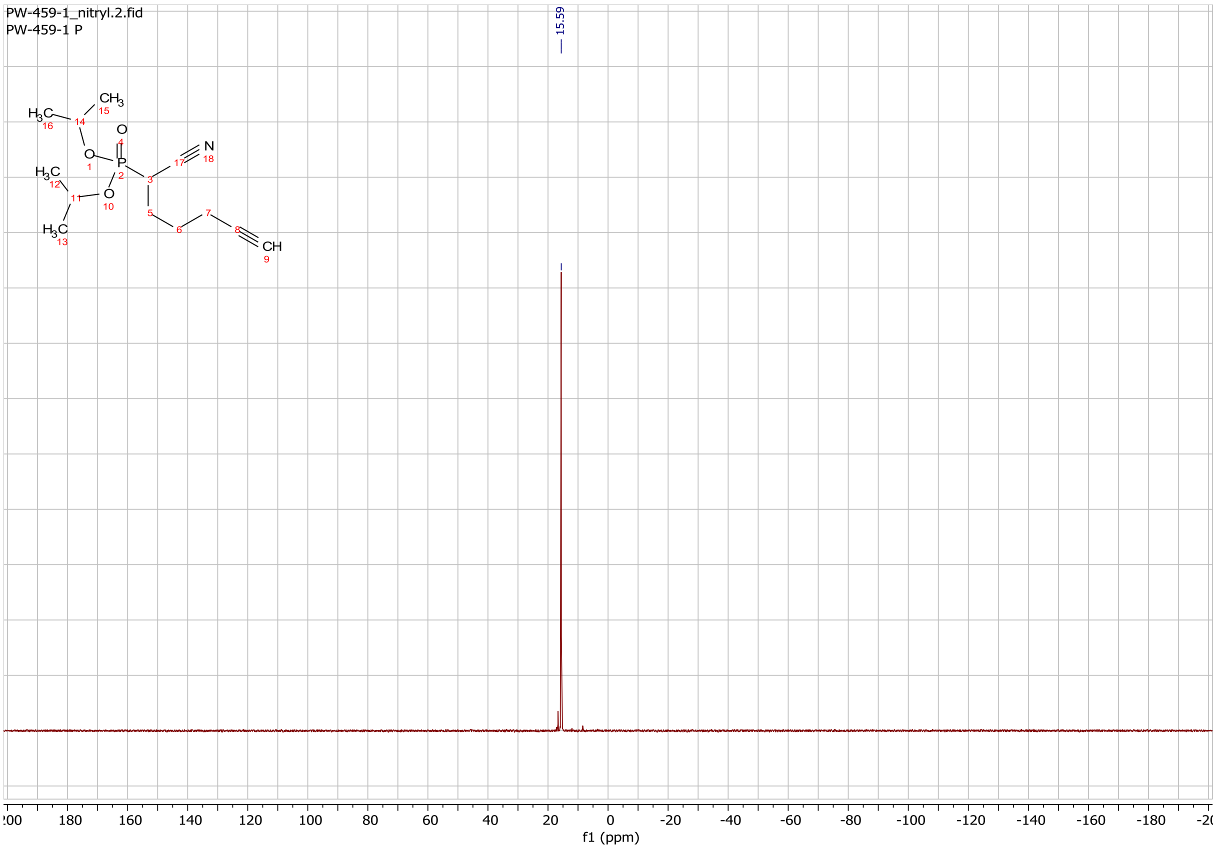
PW-459-1_nitryl.2.fid
PW-459-1 P
15.59
f1 (ppm)

Diethyl (2-oxooct-7-yn-3-yl)phosphonate (S8)

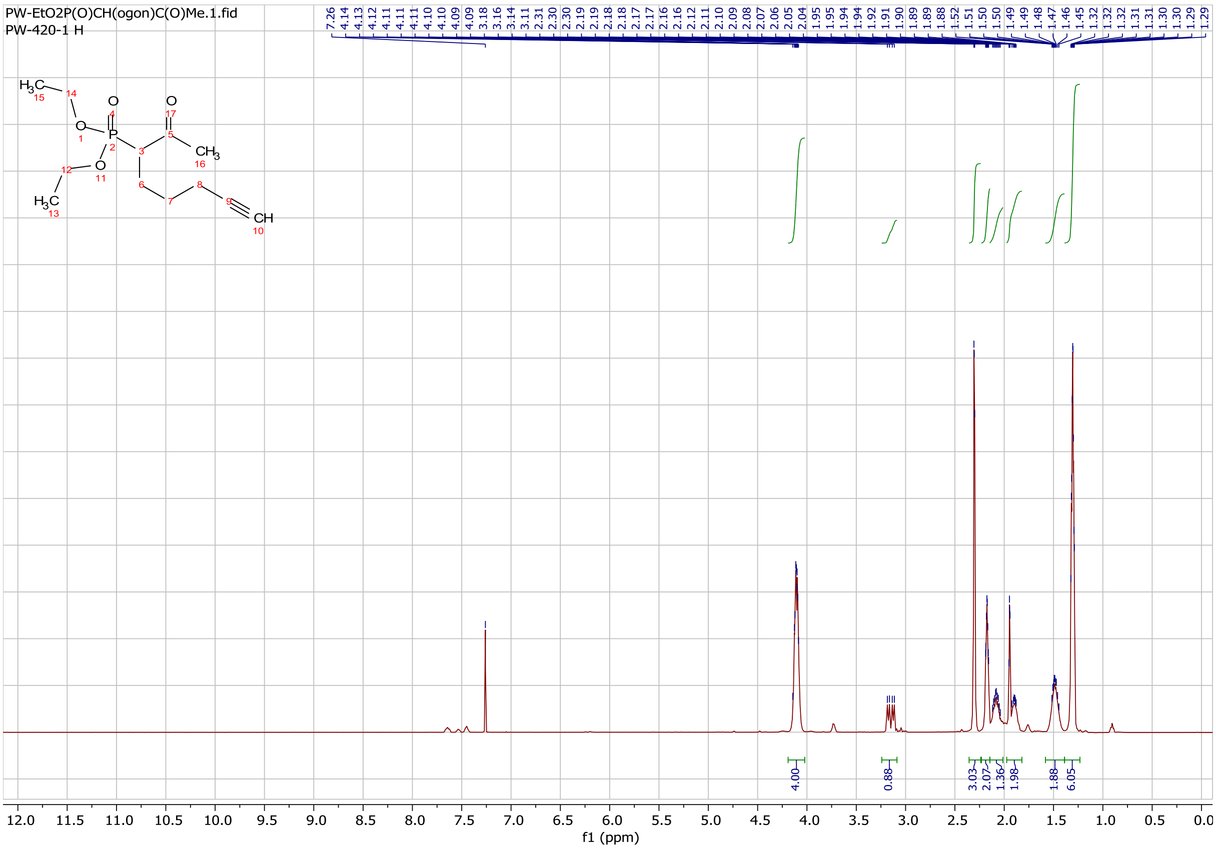

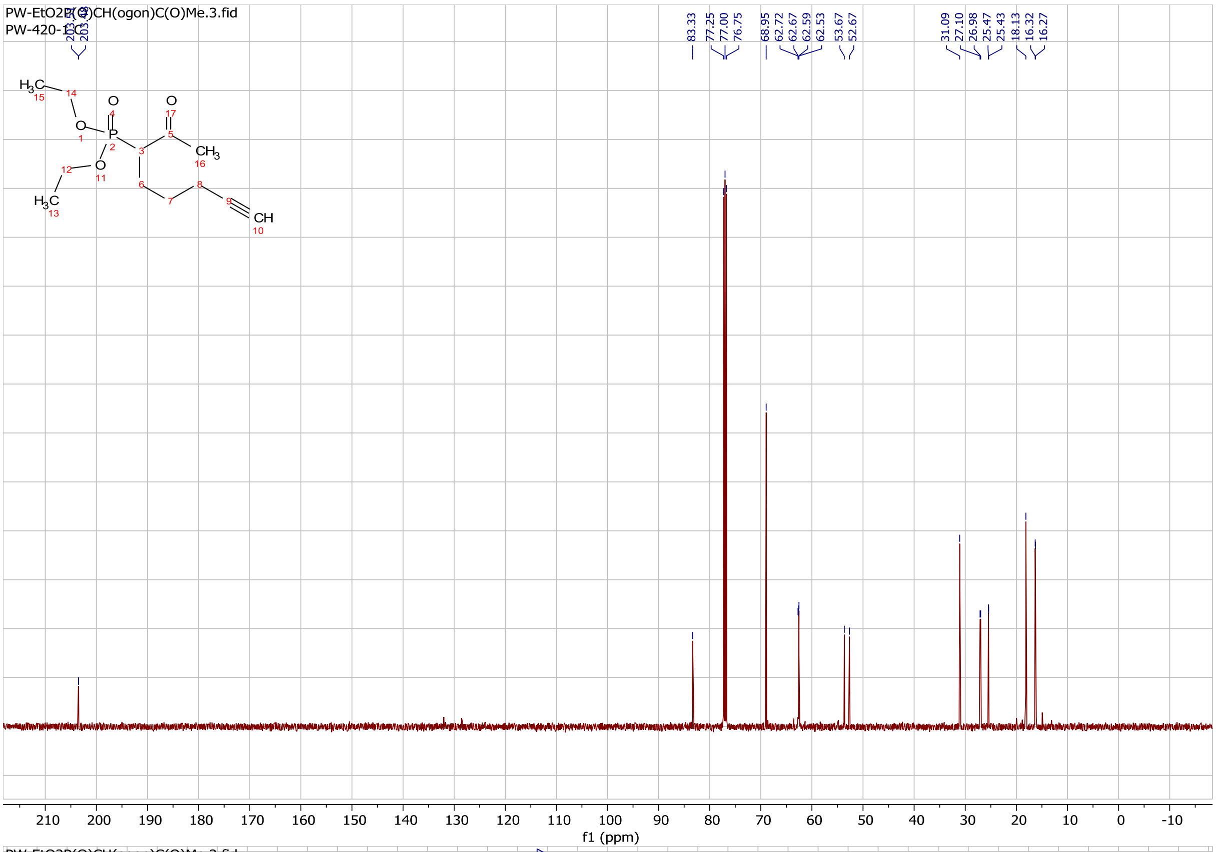
PW-EtO2P(O)CH(ogon)C(O)Me.3.fid
83.33
77.25
77.00
76.75
68.95
62.72
62.67
62.59
62.53
53.67
52.67
31.09
27.10
26.98
25.47
25.43
18.13
16.32
16.27
f1 (ppm)

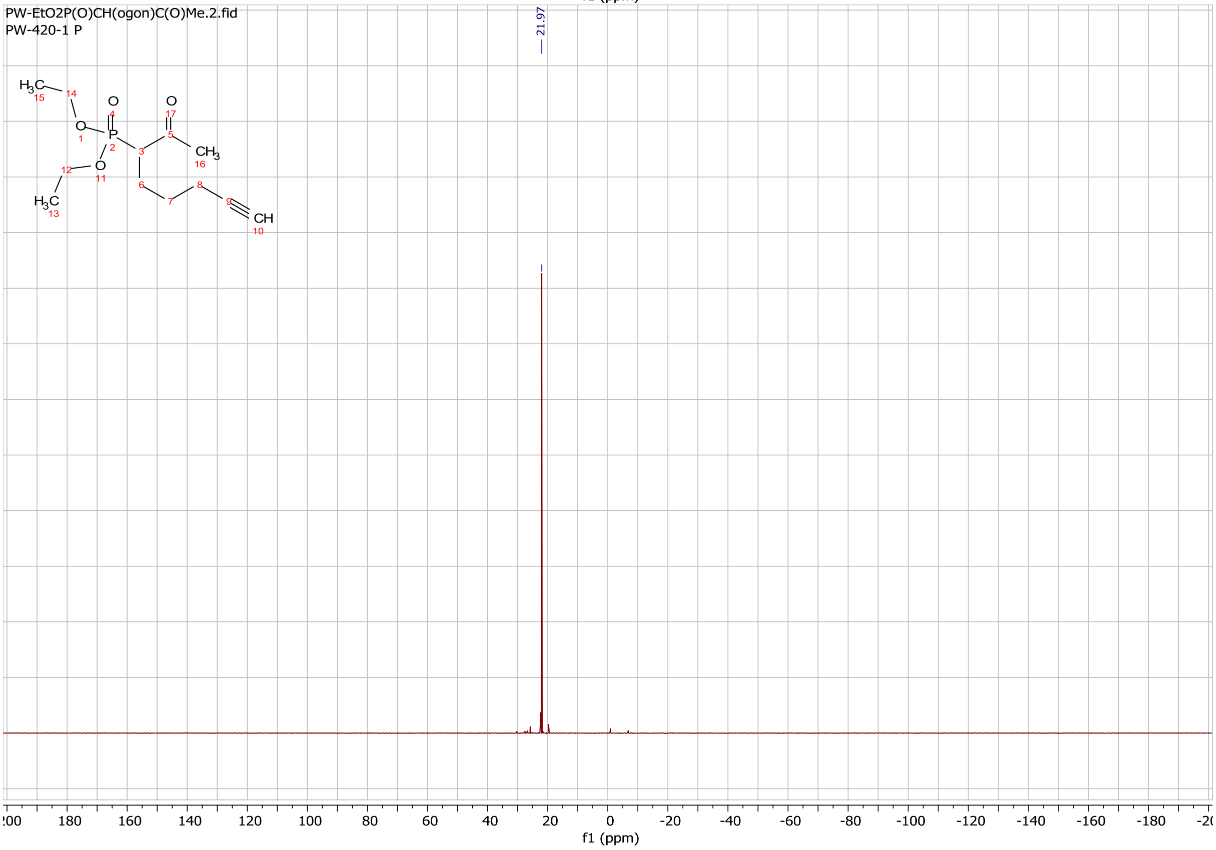
PW-EtO2P(O)CH(ogon)C(O)Me.2.fid
PW-420-1 P
21.97
f1 (ppm)

Ethyl 2-(diisopropoxyphosphoryl)hept-6-ynoate (S9)

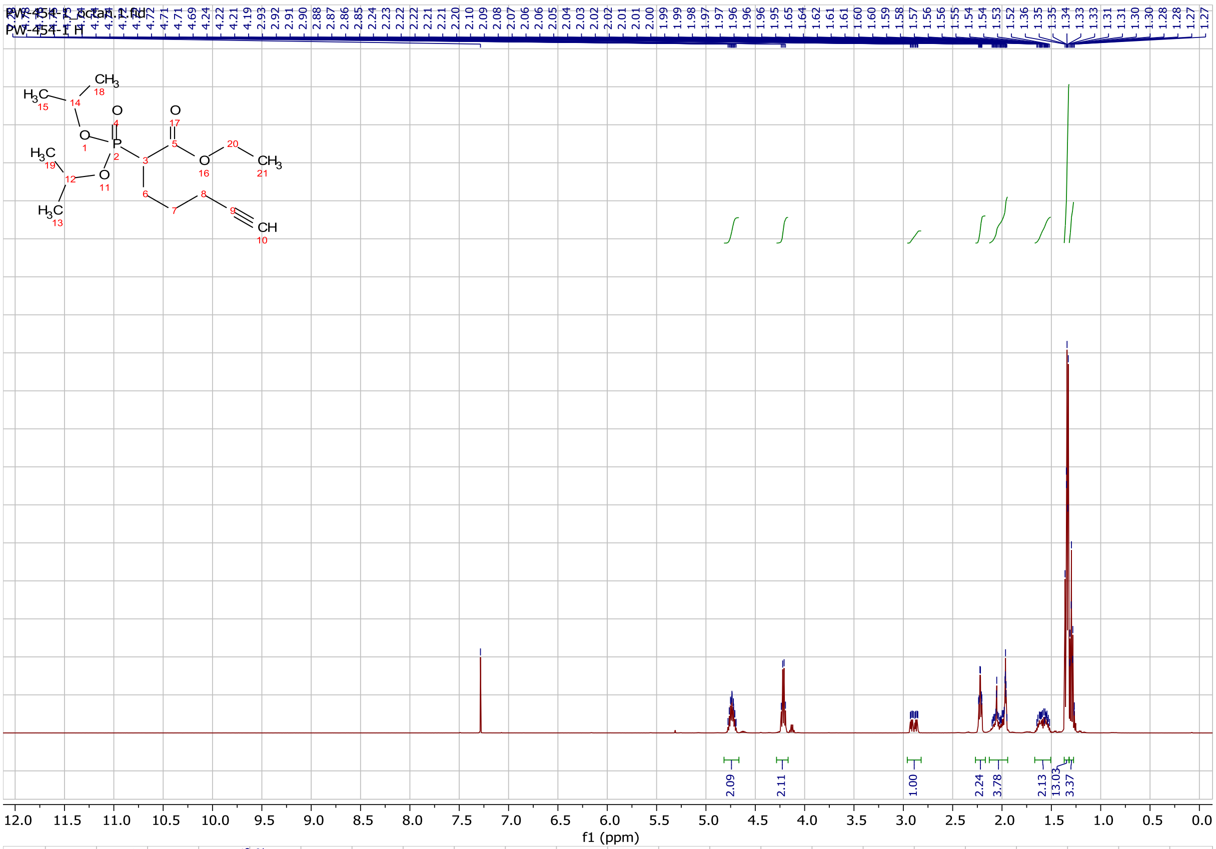

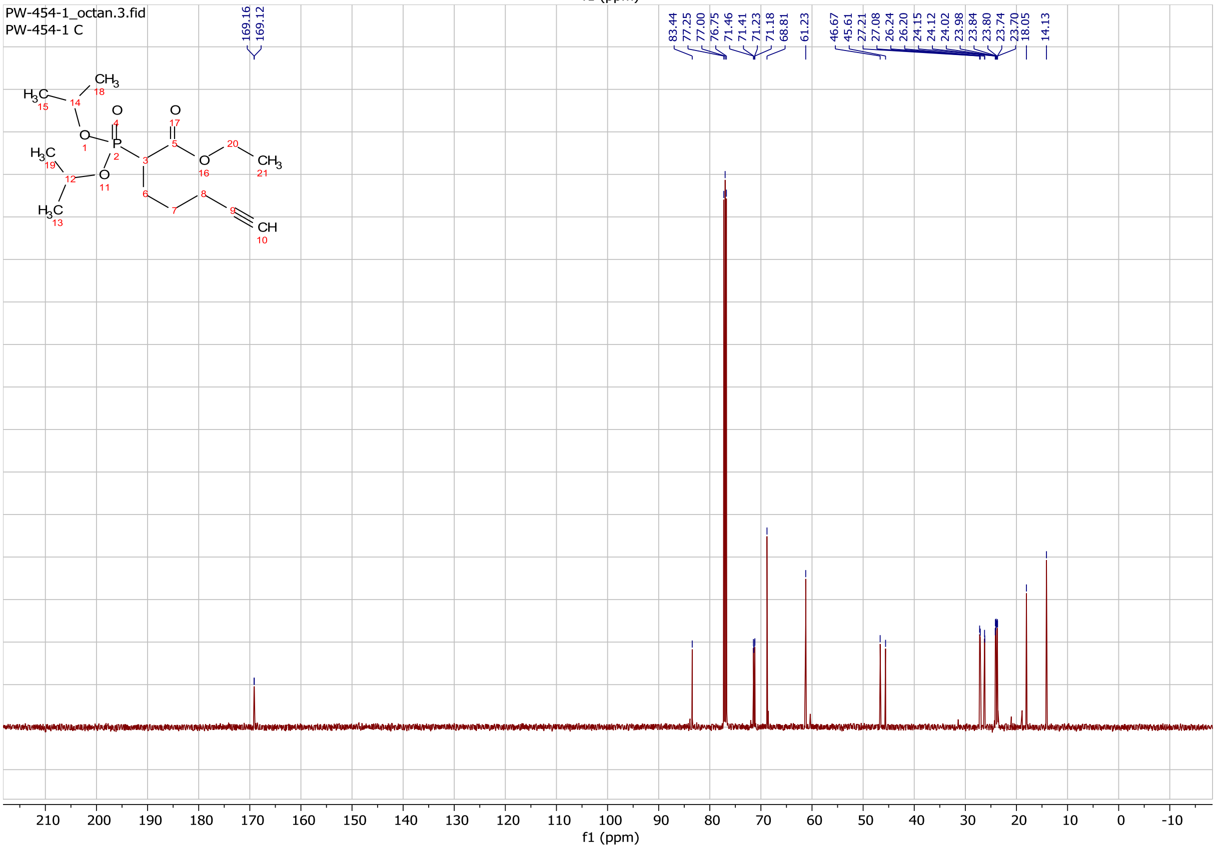

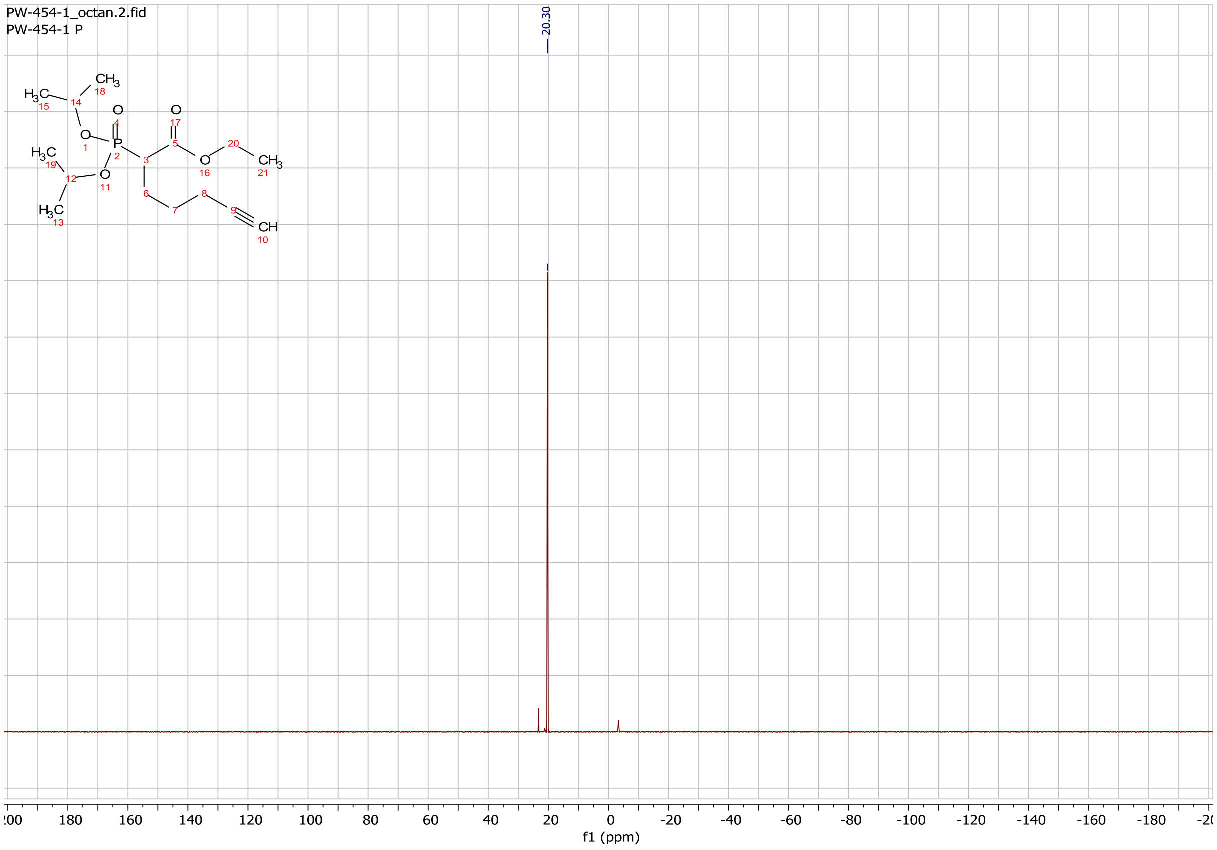
PW-454-1_octan.2.fid
PW-454-1 P
20.30
f1 (ppm)

2-(Diphenylphosphoryl)hept-6-ynenitrile (S10)

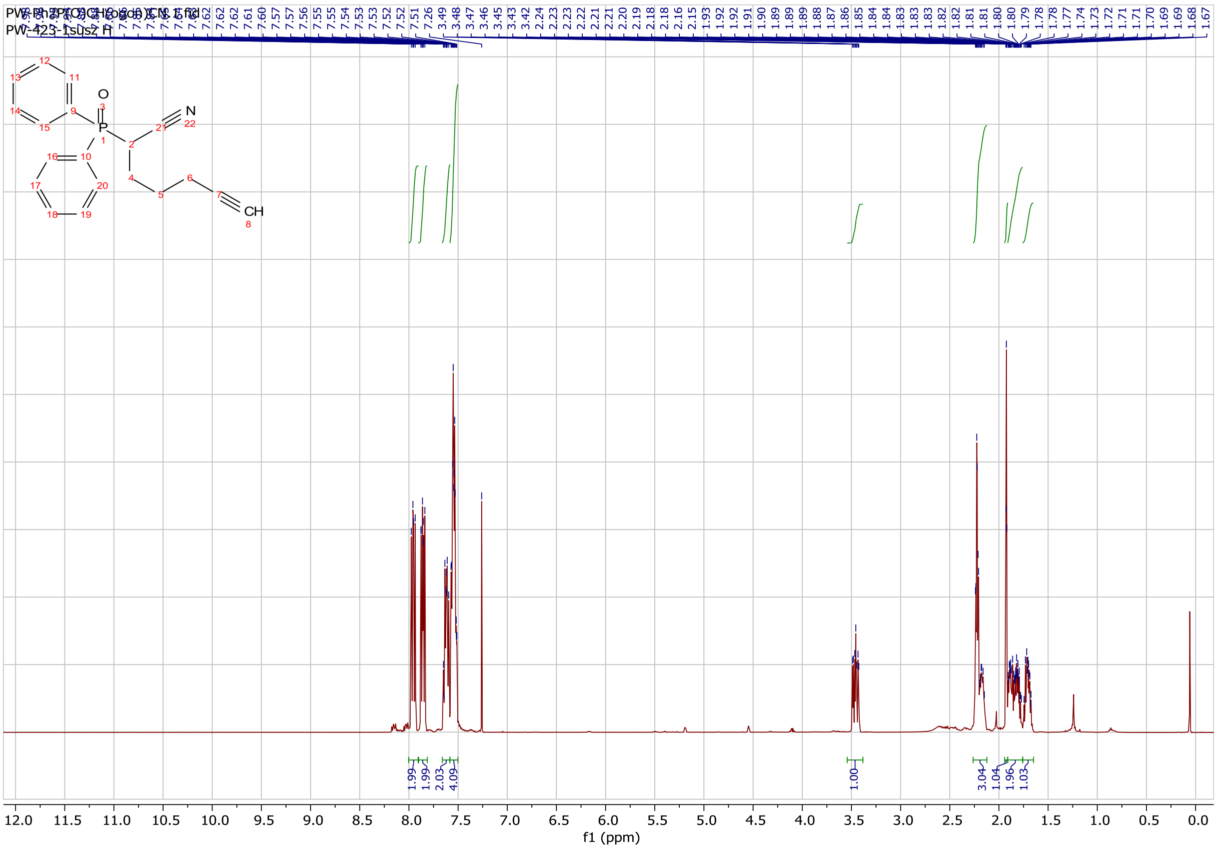

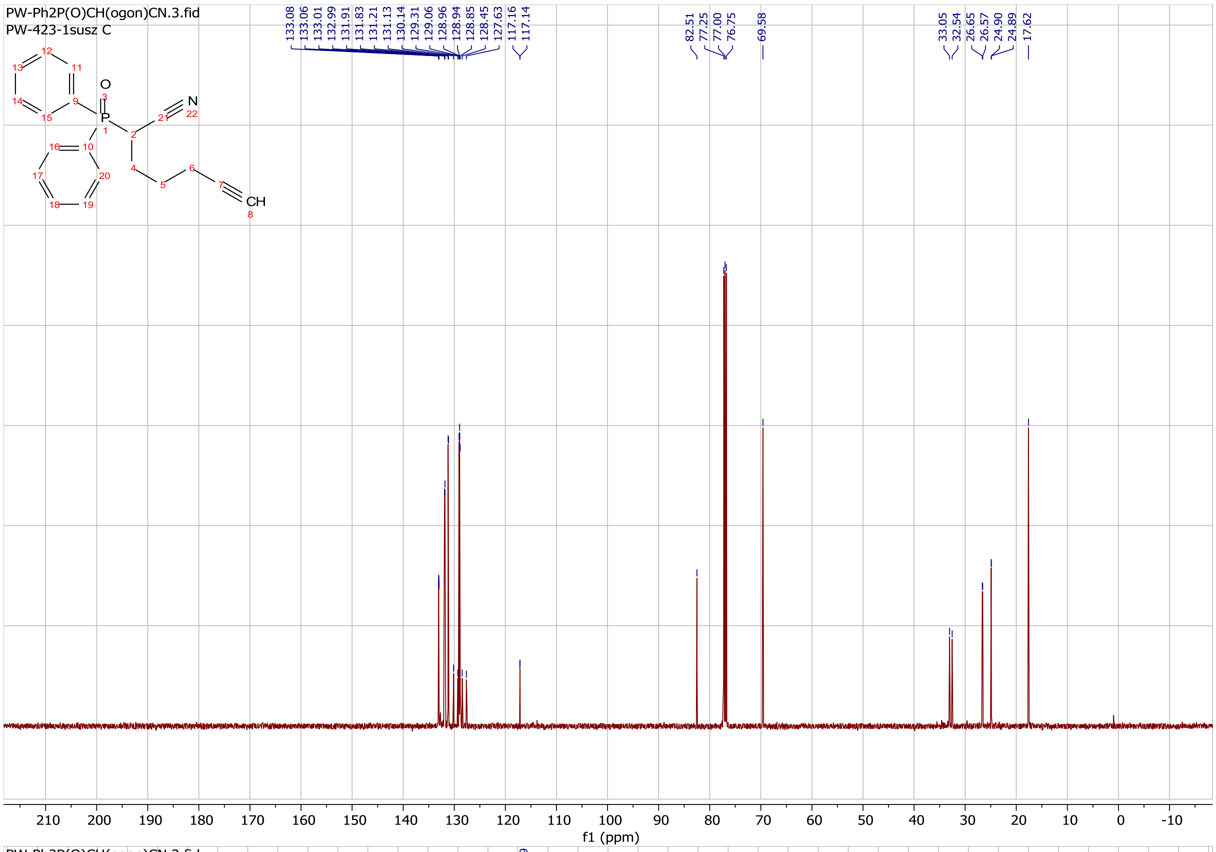
PW-Ph2P(O)CH(ogon)CN.3.fid
PW-423-1susz C
133.08
133.06
133.01
132.99
131.91
131.83
131.21
131.13
130.14
129.31
129.06
128.96
128.94
128.85
128.45
127.63
117.16
117.14
82.51
77.25
77.00
76.75
69.58
33.05
32.54
26.65
26.57
24.90
24.89
17.62
f1 (ppm)

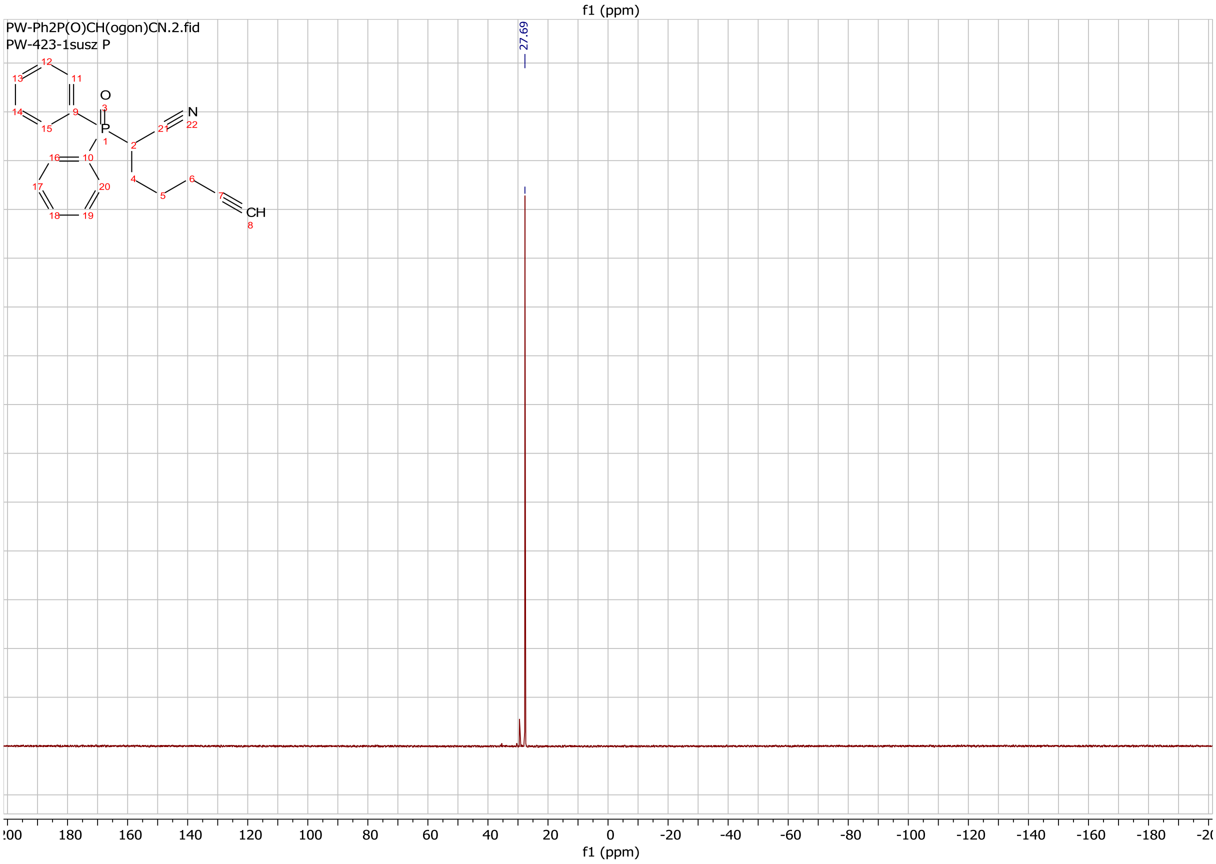
PW-Ph2P(O)CH(ogon)CN.2.fid
PW-423-1susz P
27.69
f1 (ppm)

3-(Diphenylphosphoryl)oct-7-yn-2-one (S11)

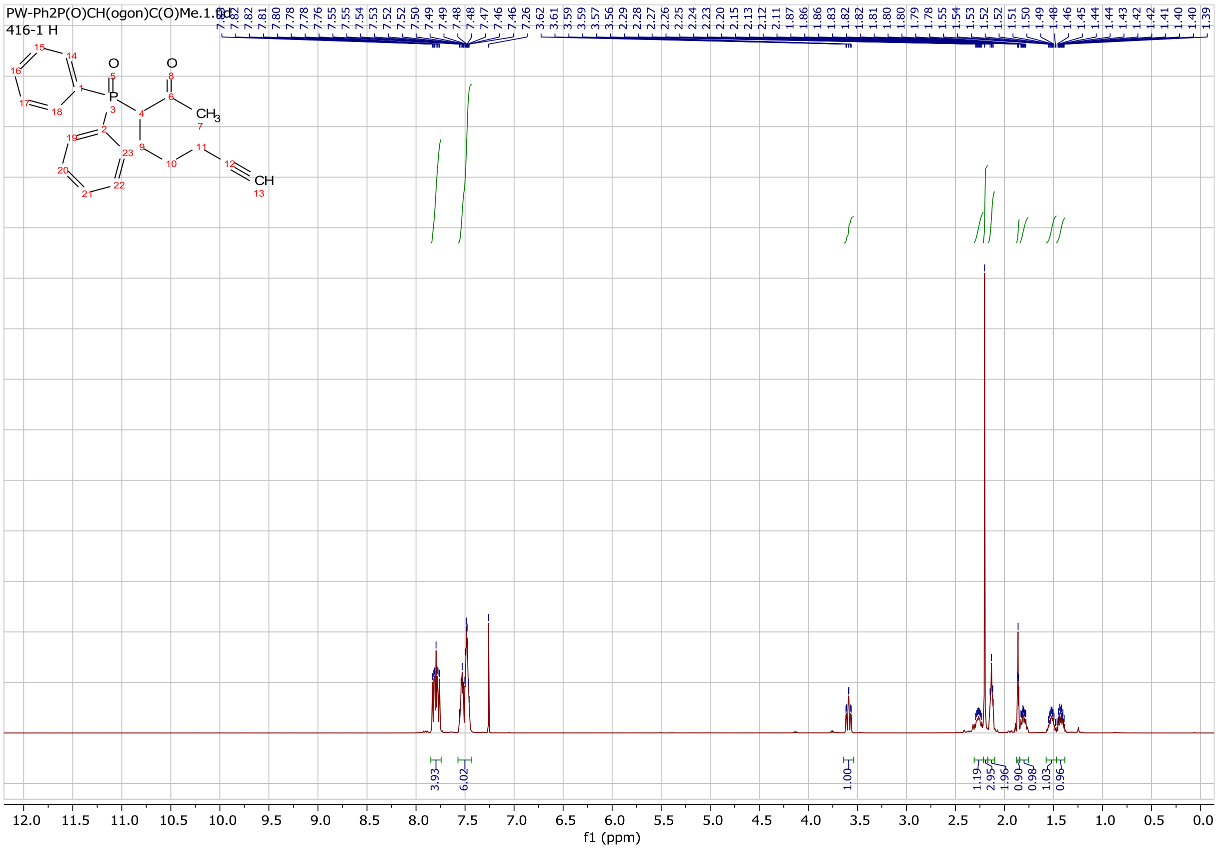

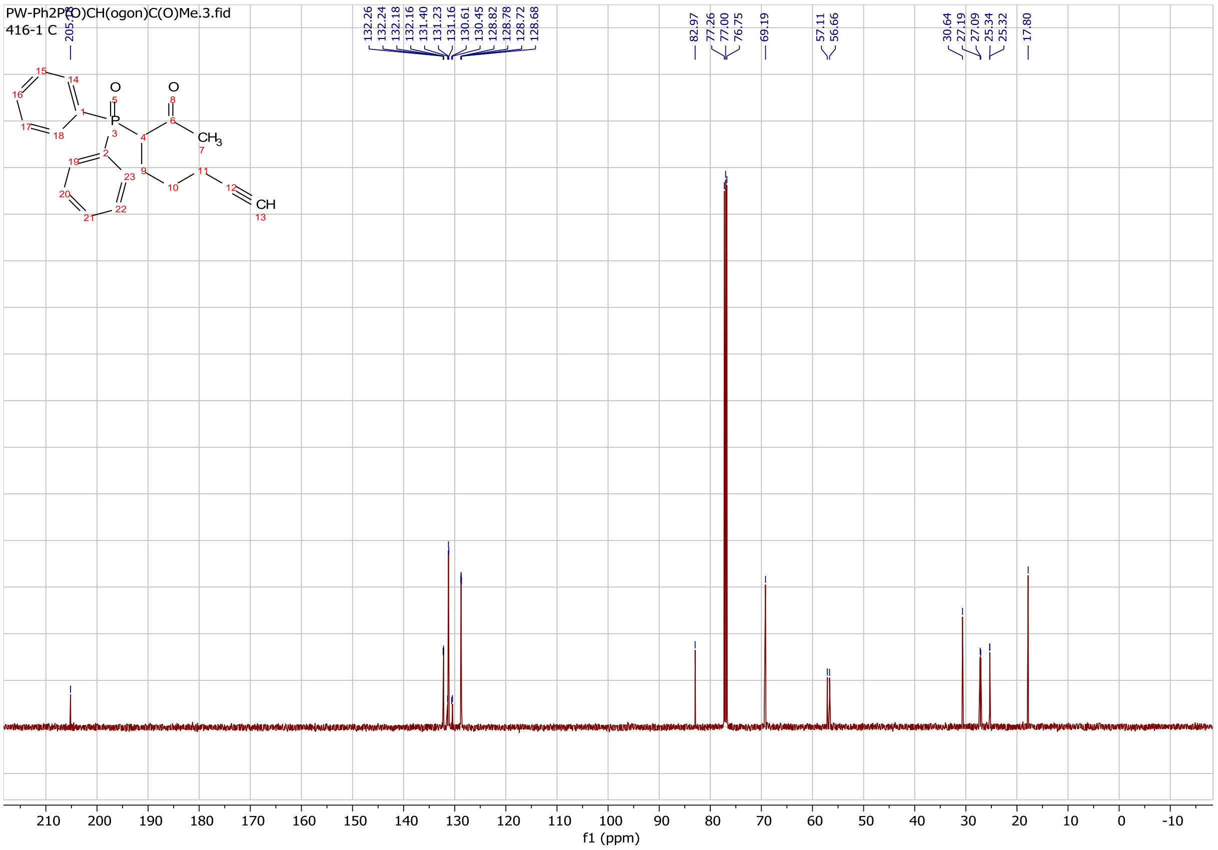

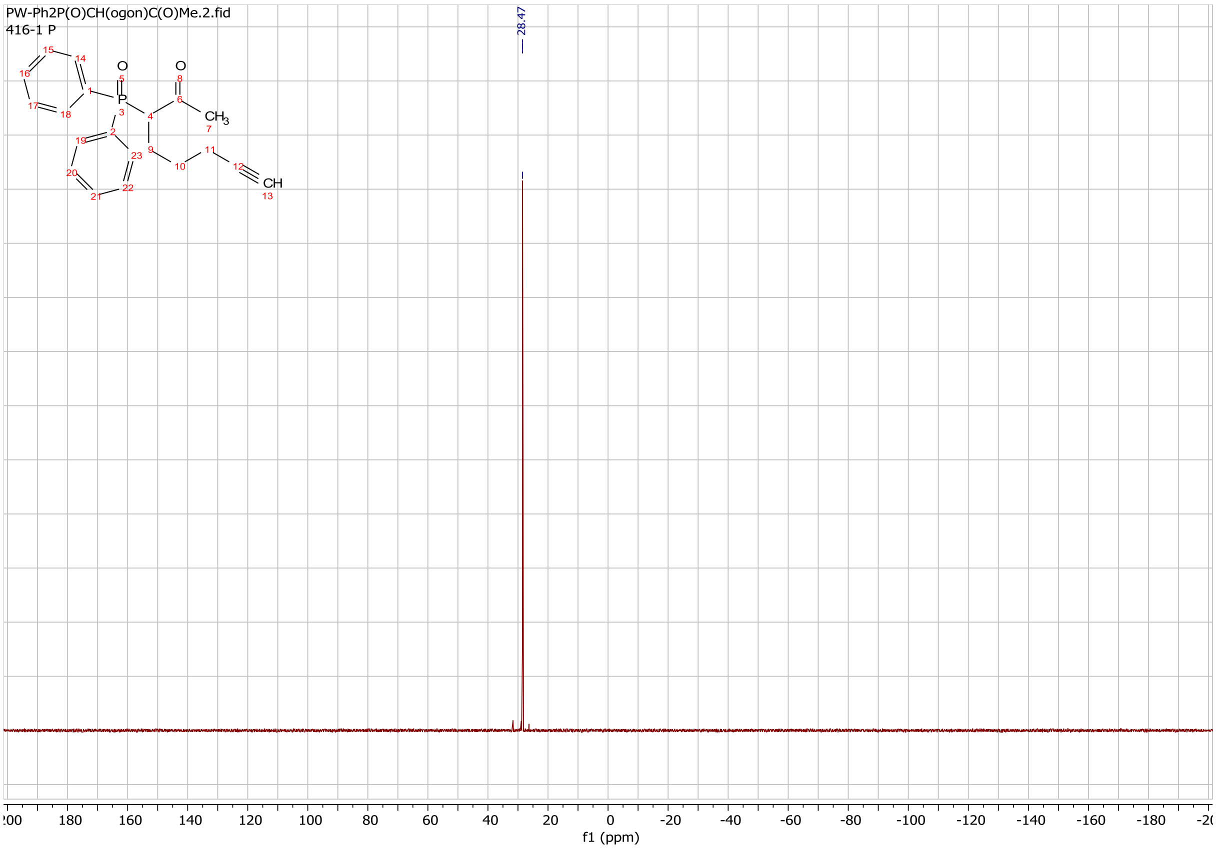
PW-Ph2P(O)CH(ogon)C(O)Me.2.fid
416-1 P
28.47
f1 (ppm)

Ethyl 2-(diphenylphosphoryl)hept-6-ynoate (S12)

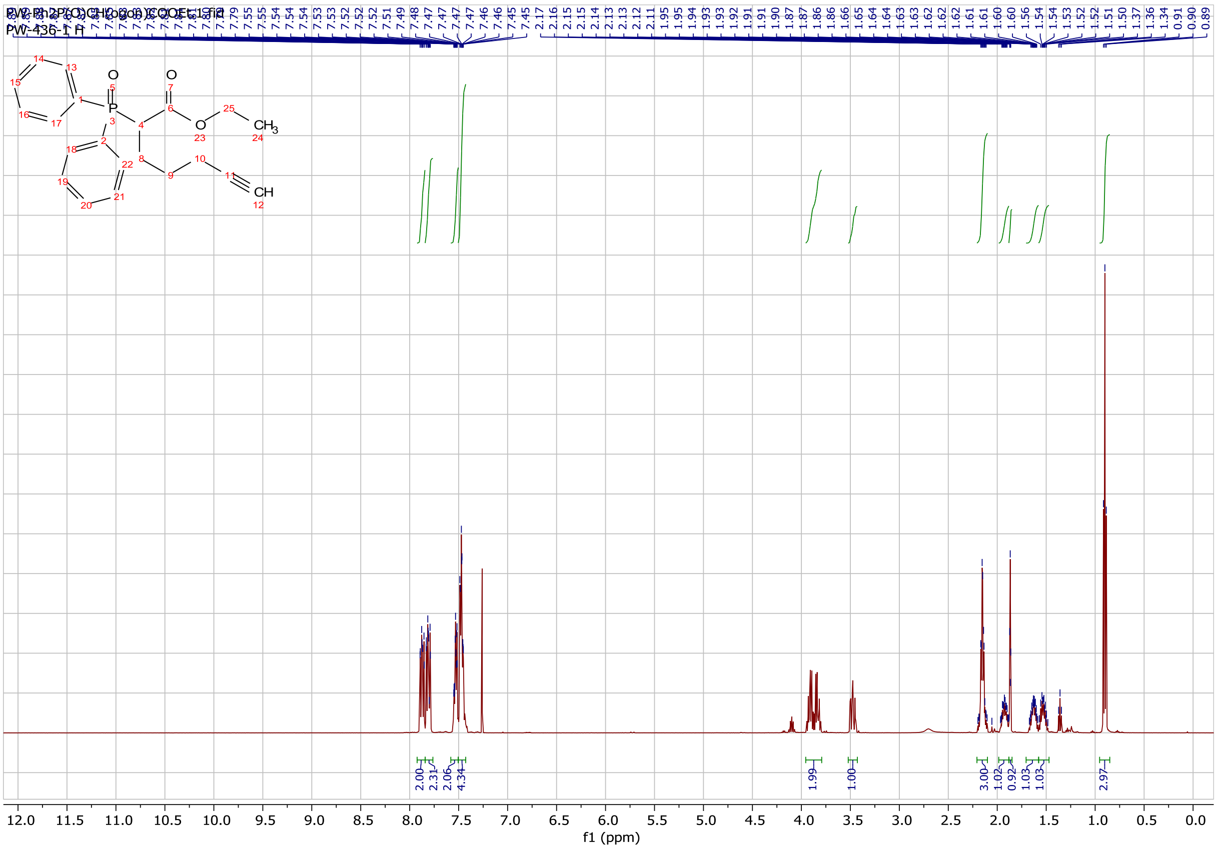

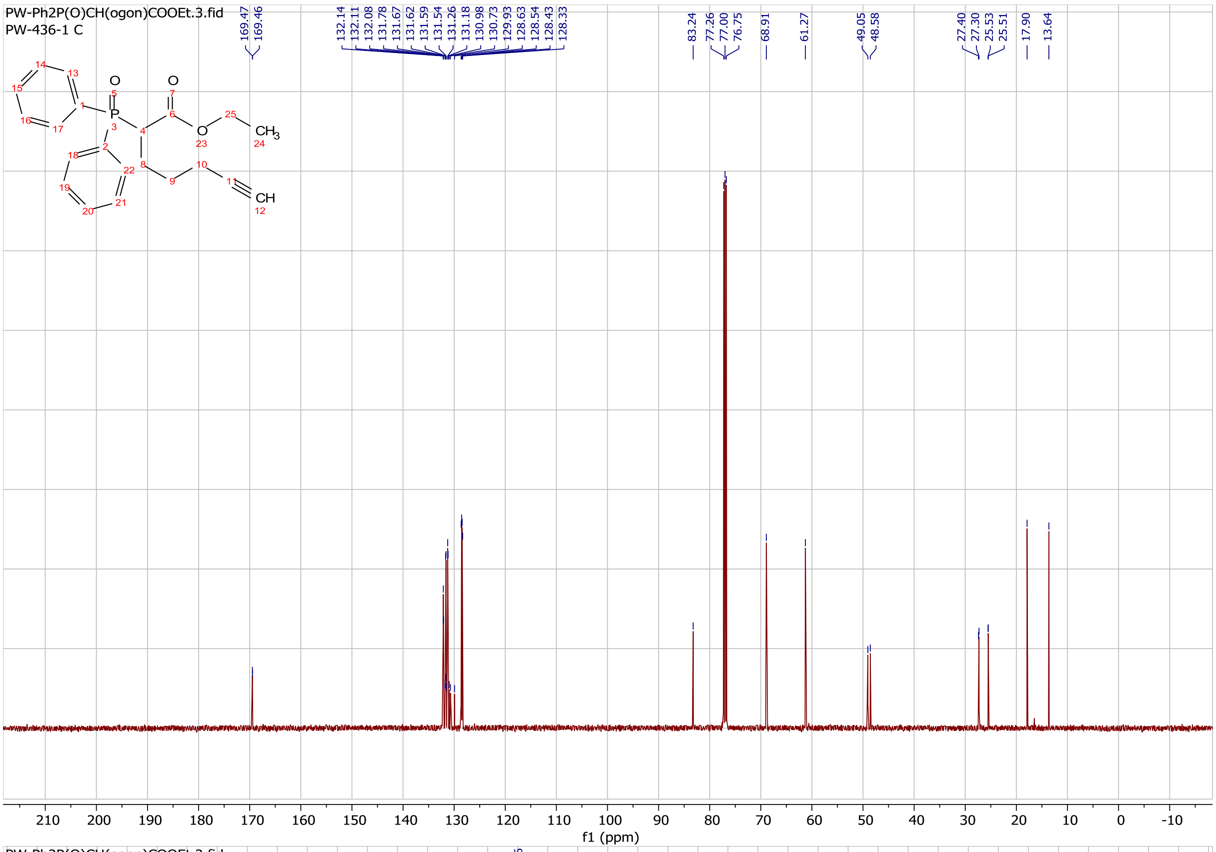

PW-Ph2P(O)CH(ogon)COOEt.3.fid
PW-436-1 C
169.47
169.46
132.14
132.11
132.08
131.78
131.67
131.62
131.59
131.54
131.26
131.18
130.98
130.73
129.93
128.63
128.54
128.43
128.33
83.24
77.26
77.00
76.75
68.91
61.27
49.05
48.58
27.40
27.30
25.53
25.51
17.90
13.64
CH3
CH
f1 (ppm)

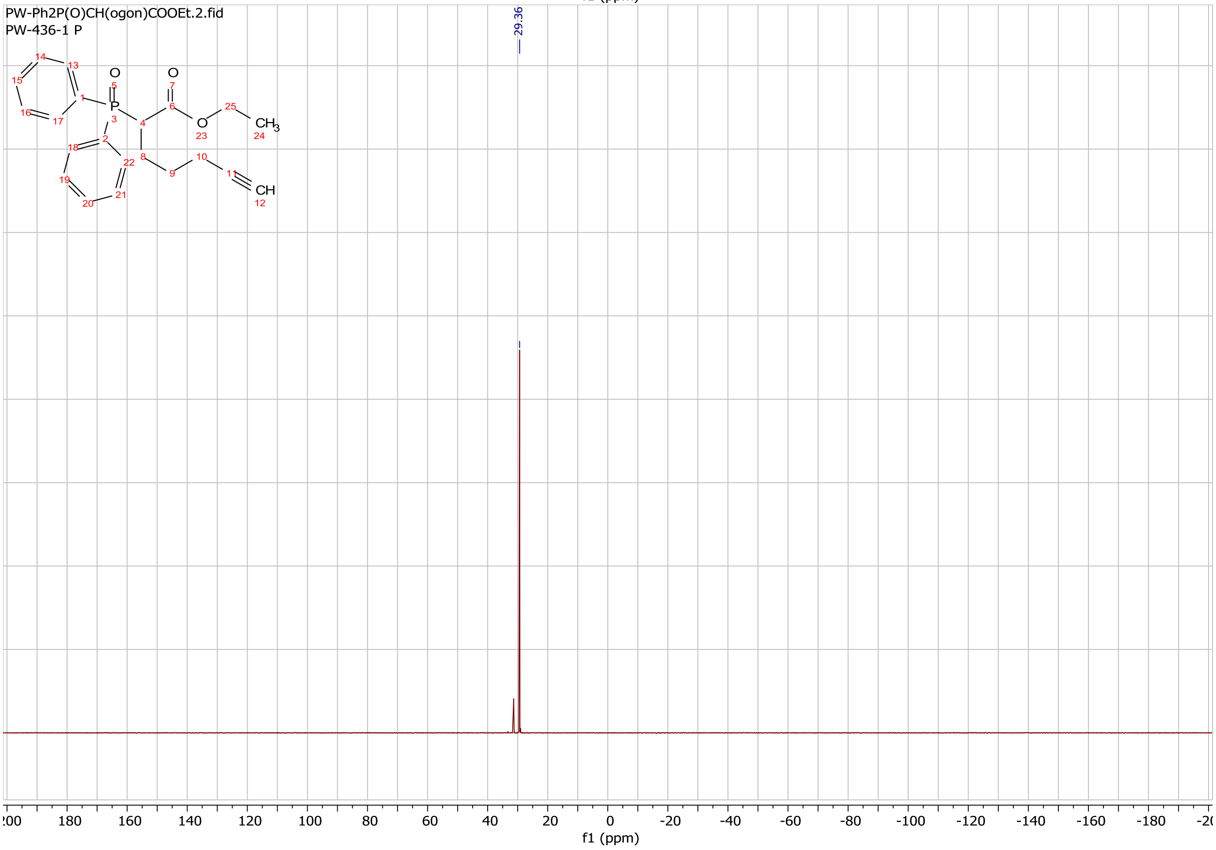

PW-Ph2P(O)CH(ogon)COOEt.2.fid
PW-436-1 P
29.36
CH3
CH
f1 (ppm)

References

1 N. C. Bruno, M. T. Tudge and S. L. Buchwald, *Chem. Sci.*, 2013, **4**, 916–920.
2 L. Huang, L. Ye, X.-H. Li, Z.-L. Li, J.-S. Lin and X.-Y. Liu, *Org. Lett.*, 2016, **18**, 5284–5287.
3 B. M. Trost, A. Breder and B. Kai, *Org. Lett.*, 2012, **14**, 1708–1711.
4 E. C. Taylor, J. E. Macor and L. G. French, *J. Org. Chem.*, 1991, **56**, 1807–1812.
5 N. Santschi and A. Togni, *J. Org. Chem.*, 2011, **76**, 4189–4193.
6 H. C. Fisher, L. Prost and J.-L. Montchamp, *European Journal of Organic Chemistry*, 2013, **2013**, 7973–7978.
7 R. W. Dugger and C. H. Heathcock, *Synthetic Communications*, 1980, **10**, 509–515.
